# Comparison and Validation of Actigraphy Algorithms Using a Large Community Dataset: Algorithm Validation Study

**DOI:** 10.2196/70778

**Published:** 2025-12-11

**Authors:** Darshan Panesar, Aashish Vichare, Jason Goncalves, Robyn Stremler

**Affiliations:** 1 Applied Psychology and Human Development, Ontario Institute for Studies in Education, University of Toronto, 252 Bloor Street West, Toronto, ON, M5S 1V6, Canada, 1 416 934 4503; 2 Independent Researcher, Vancouver, BC, Canada; 3 Independent Researcher, Toronto, ON, Canada; 4 Lawrence Bloomberg Faculty of Nursing, University of Toronto, Toronto, ON, Canada

**Keywords:** accelerometer, actigraphy, algorithm, Multi-Ethnic Study of Atherosclerosis, M.E.S.A., polysomnography, sleep, sleep monitoring, sleep disorder, wake

## Abstract

**Background:**

For decades, the measurement of sleep and wake has relied upon watch-based actigraphy as an alternative to expensive, obtrusive clinical monitoring. At the time of this publication, we have relied upon a handful of algorithms to score actigraphy data as sleep or wake. However, these algorithms have largely been tested and validated with only small samples of young, healthy individuals.

**Objective:**

This study aimed to establish the accuracy and agreement of conventional and traditional actigraphy algorithms against polysomnography, the clinical standard, using the diverse Multi-Ethnic Study of Atherosclerosis (MESA) sleep dataset. As a secondary objective, we examined algorithm and polysomnography agreement for key sleep metrics including total sleep time (TST), sleep efficiency (SE), and wake after sleep onset (WASO).

**Methods:**

We assessed 5 well-established algorithms, including Cole-Kripke, University of California San Diego (UCSD) scoring, Kripke 2010, Philips-Respironics, and Sadeh, with and without rescoring across 1440 individuals (M_age_=mean 69.36, SD 8.97) from the MESA sleep dataset. We conducted epoch-by-epoch comparisons assessing accuracy, confusion matrix analyses, receiver operator characteristic curves (ROC), area under the curve (AUC), and Bland-Altman analyses for agreement.

**Results:**

Primary results indicated all algorithms demonstrated accuracy between 78%‐80% with the highest accuracy by the Kripke 2010 (80%) algorithm followed closely by the Cole-Kripke (80%) and Philips-Respironics (80%‐79%) algorithms. In addition, moderate Cohen κ agreement and moderate positive Matthews correlations were demonstrated by all algorithms. Further, all algorithms demonstrated significant mean difference across sleep metrics.

**Conclusions:**

The findings of this study establish that these traditional actigraphy algorithms can, with high accuracy, detect sleep and wake in large, diverse population samples, including older adults or populations at risk of health conditions. However, these algorithms may carry difficulty for precise assessment of sleep metrics, especially in cases of sleep disorders or irregular sleep.

## Introduction

### Background

Over several decades, actigraphy has been used to objectively examine rest and wake periods in a variety of participants. Actigraphs are accelerometers that measure activity (acceleration of motion) levels of the person wearing the device [1-3]. Given its small footprint and lower cost, actigraphy is often used as a simple alternative to traditional, more invasive sleep monitoring methods. Actigraphy is used to measure basic movement activity patterns of individuals noninvasively to assess when they are asleep (rest) or awake (wake) [1-3]. Movement data collected by actigraphs are analyzed using validated algorithms to determine, for each minute of recording, whether the wearer was asleep or awake. At the time of this publication, the vast majority of sleep-wake analysis of movement data collected by actigraphy continues to be done primarily using one of a handful of popular linear regression models. These include Cole-Kripke, University of California San Diego (UCSD), Sadeh, and Philips-Respironics. Though these algorithms are extensively used, they have largely been tested with only small samples of young and healthy individuals [3-11]. There is little research examining the comparison between and validity of these algorithms with a large, demographically diverse sample or with a sample at risk of health conditions. Given the increasing use of actigraphy for a variety of populations, it is critical to evaluate these algorithms across a large, diverse population to give a better representation of their accuracy. Further, it is important to evaluate the accuracy of these algorithms across older adults, as these populations have higher susceptibility to, and high prevalence of health disorders. Given the wide range of studies using actigraphy, it is important to understand actigraphy’s performance when used with these higher-risk populations for sleep-wake assessment [3,12-17]. In addition, given the comorbidity of sleep issues with other health disorders, the use of actigraphy in sleep studies has become more commonplace [3,12-17]. Therefore, it is highly important to assess the accuracy and reliability of traditional actigraphy algorithms with these populations.

### Multi-Ethnic Study of Atherosclerosis Dataset

There are several significant challenges in collecting a large, diverse dataset. This, in part, is due to the ideal method of validation (for actigraphy), which is polysomnography, the gold standard for sleep assessment. Polysomnography and its analysis are expensive, clinic-based procedures that require extensive equipment and expertise, are highly time-consuming to conduct, and are therefore impractical for measuring sleep across several nights with large samples [8,18,19]. However, an ongoing initiative called the Multi-Ethnic Study of Atherosclerosis (MESA) has conducted a large sleep study (over 2000 participants), including time-synchronized actigraphy and polysomnography data as part of their larger initiative [20,21]. This sleep sample represents a portion of over 6800 participants between the ages of 45 and 84 years, free of cardiovascular disease at baseline, who were monitored longitudinally for subclinical cardiovascular disease [20,21]. This dataset presents a novel opportunity to establish a concrete comparison between conventional algorithms and establish their accuracy across a large population of older adults who are also at risk of health issues. Thus, this paper evaluates traditional actigraphy algorithms using the MESA dataset.

### Current Study

The primary objective of this study was to establish the accuracy of traditional actigraphy algorithms against the gold standard polysomnography using a large established dataset. In doing so, we provide comprehensive foundational benchmarks of the most commonly used traditional actigraphy algorithms. Further, we aimed to also establish performance benchmarks of commonly used actigraphy algorithms across a diverse population with older adults and sleep pathologies. These benchmarks greatly inform research and clinical use of these algorithms. Further, this study provides detailed standards to developers of novel actigraphy technologies and actigraphy analysis methodologies. Based on previous, albeit smaller, sample validation studies, we hypothesized that traditional actigraphy algorithms would detect sleep and wake with high accuracy. In addition, we predicted that additional rescoring of actigraphy would help improve their accuracy over the algorithms alone.

In addition to sleep-wake, researchers and clinicians often examine sleep metrics to evaluate patterns or disruption of sleep patterns. As our secondary goal, we examined each algorithm’s agreement and bias with polysomnography on several commonly studied sleep metrics, including total sleep time (TST), sleep efficiency (SE), and wake after sleep onset (WASO). TST is the total number of minutes an individual sleeps during the night from the first onset of sleep to sleep offset. On the other hand, WASO is the total duration of wake between the first onset of sleep and the sleep offset. Finally, SE is the percentage of time asleep (TST) during the total time spent in bed. As these are key sleep metrics, this study’s results provide detailed information for current research and clinical practice as well as benchmarks for future improvements to current and novel actigraphy methods. We hypothesized that overall, sleep metrics derived from actigraphy algorithm-processed activity would have high agreement with polysomnography [22].

As an exploratory analysis, we looked at both accuracy and sleep metrics of each algorithm and evaluated possible points of failure and variables that impact the performance of actigraphy analyses. Specifically, we examined if accuracy results and sleep metrics would be poorer for subgroups of participants who had sleep disorders. To our knowledge, this is the first study to provide detailed actigraphy performance metrics across large key samples of individuals with sleep problems. These metrics provide nuanced information that will critically facilitate current and future practice. We discuss the implications of these variables, making recommendations for future directions of research.

## Methods

### Sample

The dataset sample for this study was derived from the MESA and acquired through the National Sleep Research Resource (NSRR) [20,21]. MESA is a large prospective, community-based study designed to examine the risk factors, prevalence, and progression of cardiovascular disease [20,21]. The MESA study was conducted from 2000 to 2002, with 6814 ethnically diverse men and women aged 45‐84 years who were free of overt cardiovascular disease and recruited from 6 US sites [20,21]. MESA participants underwent periodic core physical assessments at enrollment and 10 years following initial recruitment (2010‐2012) [20,21]. A subset of participants was invited to participate in the MESA ancillary sleep assessment study. This study entailed a home visit (1 night) in which home polysomnography was conducted along with concurrent actigraphy. Polysomnography data were analyzed by one of 3 polysomnologists using standard guidelines, while 2 scorers completed actigraphy analysis by first marking the sleep period and then using automatic scoring software. Scorers completed a MESA-rule–based training and certification before scoring, and postscoring both interscorer reliability was assessed at 2 timepoints for sleep stages (which pertained to the sleep data used for the study). At time point 1 for sleep stage scoring, the interscorer intraclass correlation coefficients (ICC) across n=27 from 9 participants were as follows: Stage 1 was 0.86, Stage 2 was 0.63, Stage 3‐4 was 0.81, and REM was 0.96 [20,21]. At time point 2, interscorer ICC across n=38 from 19 participants were as follows: Stage 1 was 0.74, Stage 2 was 0.81, Stage 3‐4 was 0.79, and REM was 0.93 [20,21]. For this study, we used raw actigraphy activity counts for analysis. For ground truth, we used the matching polysomnography sleep-wake data that was collapsed to wake (coded wake stage) versus all sleep stages collapsed to one “sleep” variable. Only one night of polysomnography data were collected by the original study which was matched and synchronized with the respective actigraphy data (cropped to match).

The data were filtered based on several criteria. The initial dataset contained 2159 samples for actigraphy and 2056 samples for polysomnography. We first excluded samples that did not have concurrent polysomnography data (103 removed). The actigraphy and polysomnography samples were then matched based on a synchronization document provided by NSRR and MESA. Those samples that did not have a corresponding synchronization or were reported as having data issues by NSRR (2 individuals) were excluded, resulting in a sample of 1831 individuals (225 removed). Next, we filtered the dataset based on polysomnography data quality as indicated by the dataset description. We included individuals for whom the quality of polysomnography data was rated as very good or better, as this rating indicated an appropriate number of channels present for accurate sleep-wake evaluation. An additional 14 participants with complete, matched data of fair and good quality were also included. This resulted in a total of 1484 samples (347 removed).

Outlier filtering, or filtering based on actigraphy, was not conducted, as we wished to retain all actigraphy data for a true representation of how actigraphy data are traditionally processed using these algorithms. Given the absence of outlier filtering, we conducted sensitivity analysis to evaluate the effects of these outliers on our overall sample results. We identified outliers in the cropped 30-second epoch synchronized (1-night) actigraphy raw activity data using a 2-step process. The raw 30-second data were used as this was the data used for each algorithm (the binary algorithm results could not be used to identify outliers). Each participant’s individual iIQR, mean, and SD of activity were calculated. For the first level, participants’ IQR was compared to the full sample IQR. Any participants whose range fell outside of 1.5 times below the first quartile and above the first quartile were identified as outliers. For the second step, the *z* score for each participant was calculated relative to the sample mean and SD. Any participants with a *z* score of 2 or greater were identified as outliers. A total of 56 participants were identified as outliers using this 2-tier approach. Once these outlier individuals were identified, they were omitted, and Cohen κ, Matthews correlation coefficient (MCC), and confusion matrix metrics were recalculated. The relative change between the full dataset and outlier-omitted results was calculated to be less than 5% across all metrics and algorithms except for the Philips 40 rescored algorithm sensitivity (5.55%) and specificity (7%). However, the relative change was for only the rescored algorithm, close to the 5% threshold, and all other metrics were within the 5% range; therefore, we determined that the outliers did not pose a significant issue. For full comparison metrics, see Multimedia Appendix 1.

Further, polysomnography data were in binary format, and the actigraphy data, once processed through an algorithm, would also be in binary data point format. This would limit the breadth of fluctuation in the main analyses and would allow for direct 1:1 comparison of epochs. In addition to the main analyses, rescoring was also applied, which is the standard method of correcting issues with actigraphy data. In addition, we retained all participants for subsequent analysis of sleep metrics, as we aimed to evaluate the entire spread of sleep metric results for normal sleep, sleep problems, and extreme cases of sleep. As a secondary measure, we added analyses for participants with sleep problems to evaluate these extreme cases separately.

**Figure 1.**
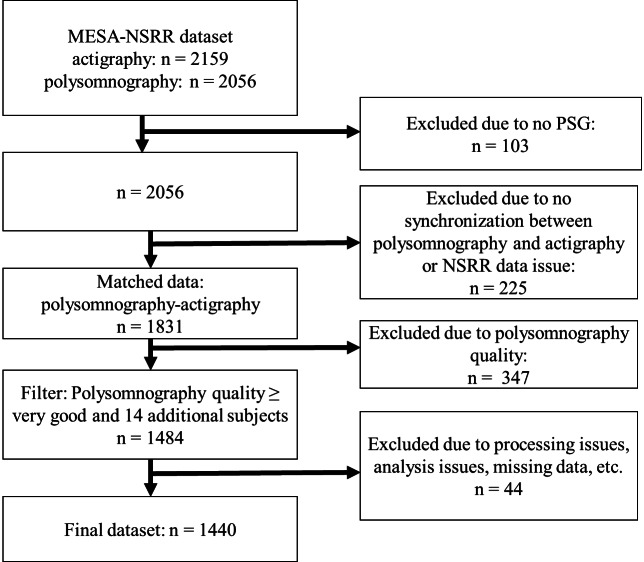
Data filtering and selection flowchart reflecting the data sample selection and filtering from the source National Sleep Research Resource–Multi-Ethnic Study of Atherosclerosis dataset. NSRR-MESA: National Sleep Research Resource–Multi-Ethnic Study of Atherosclerosis;

Finally, during data processing, an additional 44 individuals were excluded due to issues synchronizing actigraphy and polysomnography time points, missing data, and issues with processing. The final sample size analyzed was N=1440 (M_age_=69.3, SD 9.0 years; n_male_=663) individuals (Figure 1). The distribution of included participants is denoted in Table 1.

**Table 1.** Baseline characteristics of the selected sample subset from National Sleep Research Resource–Multi-Ethnic Study of Atherosclerosis sleep dataset.

Characteristic	Values
Age at study (years), mean (SD)	69.3 (9)
Age (years), range	54‐94
Sex, n (%)	
Female	817 (55.7)
Male	663 (46)
Race, n (%)	
White	565 (39.2)
Black/African American	396 (27.5)
Hispanic	351 (24.4)
Asian	168 (11.7)
Sleep problems, n (%)	
Sleep apnea	104 (7.2)
Insomnia	84 (5.8)
Restless legs syndrome	66 (4.6)
Use of CPAP^a^ machine for sleep	62 (4.3)
Snoring ≥3‐5 times per week	584 (40.6)

a CPAP: continuous positive airway pressure.

In addition to the final sample, for exploratory analyses, subsamples for the populations with sleep problems were also selected. Four sleep problem groups, including apnea, individuals who used continuous positive airway pressure (CPAP), insomnia, and restless leg syndrome (RLS), were examined using the same detailed analyses and methods as the full sample (Table 1).

### Data Processing

Data processing was conducted using Python 3 with a wide range of libraries and platforms. Before the final analyses, both actigraphy data and polysomnography data were transformed from 30-second epochs to 1 minute to standardize, since many algorithms and previous studies typically present minute-by-minute epochs for actigraphy for easier data analysis and interpretation [22,23]. For the actigraphy data, transformation was done after algorithm results were obtained. For the actigraphy results, once each respective algorithm had processed the data, the actigraphy values from the algorithms were summed to collapse the data to 1-minute epochs. The binarized polysomnography data were also transformed to 1-minute epochs (from 30-second epochs) using the following set of rules. First, if 2 adjacent 30-second epochs were both coded as sleep, the resultant minute would be sleep. Second, if 2 adjacent 30-second epochs were both coded as wake, the resultant minute would be wake. Third, if one of 2 adjacent 30-second epochs were wake, the resultant minute would be wake. Polysomnography transformation to collapse epochs to 1-minute intervals is a common practice for actigraphy comparison [24-27]. With respect to the wake-wins, evaluation of the distribution of pairs was conducted for the polysomnography epochs across all participants. A total of 55.13% of pairs were both asleep, 38.33% were both awake, and only 6.54% were mixed (wake-sleep or sleep-wake). Given the low percentage of mixed pairs, we expected the effect of data transformation to be limited with respect to the wake-wins condition.

All data transformations were again done through Python 3, using a wide range of libraries with coding assistance provided by ChatGPT (OpenAI) to generate Python code for existing Python libraries [28]. ChatGPT was used to generate Python code for file data file preparation and processing (eg, data transformation). Manual data checks in addition to code-based error reporting were conducted to verify the code was working as desired and data were correctly processed. In addition, ChatGPT was used to generate Python code for running statistical analyses. All statistical formulae and analyses were verified within the code. Once code was generated, it was checked by at least one or more of the study authors before use. No machine learning code was generated via ChatGPT, and any actigraphy algorithm code was checked and specified in the Python code exactly as defined by the original algorithm sources. Data were synchronized based on the NSRR and the MESA-provided synchronization guide, which highlighted polysomnography start times relative to actigraphy.

#### Actigraphy Algorithms

For the application of actigraphy algorithms on the actigraphy data, we used Python (Python 3)-based platform pipelines. For this study, we uploaded raw actigraphy data. This platform allows data processing by simply selecting some basic parameters, that is, the specific algorithm for processing and the files. All files were processed through each algorithm, respectively. Once the algorithm value was calculated, raw results were collapsed to 1-minute epochs by summing the raw result values. We then applied each algorithm’s respective classification threshold to determine whether each epoch was sleep or wake. These thresholds were prespecified by the respective algorithm authors in the original articles.

Algorithms were selected on the basis that they were well established and commonly used throughout the literature for actigraphy analyses across all populations, including with older adults and in populations with sleep disorders [29-36]. However, concrete performance benchmarks have yet to be established. The most used algorithms at the time of this publication are Cole-Kripke (cited 2413 times in Google Scholar) and Sadeh (cited 1795 times in Google Scholar) algorithms. The Cole-Kripke has been used across older adult and sleep disorder populations in several studies [31,34]. The Philips algorithm was selected as the actigraphs used for the MESA actigraphy data collection were Philips Actiwatch, which are also among a commonly used clinical actigraphs [21,22]. Additional algorithms were selected based on the notation that they were adapted from the Cole-Kripke algorithm to be used in various sleep populations and cases with varying parameters. Further, all algorithms had variants that could handle 30-second epoch data or 1-minute epoch data, allowing the use of our dataset and for comparison between algorithms. Generally, the selected algorithms all follow a similar regression-based approach to determine sleep and wake, allowing for comparability and comprising a comprehensive spread of traditionally used actigraphy algorithms. The selected algorithms noted below were evaluated in this study [22].

#### Cole-Kripke (CK)

Cole et al [6] developed an actigraphy analysis, and to date, this algorithm has been heavily used throughout actigraphy research analysis. The CK algorithm was developed for a variety of epochs, including 30-second and 1-minute epochs [6]. The algorithm is regression based, using a 7-epoch window to compute whether a participant is awake or asleep. The original Cole et al [6], 30-second version of the algorithm used for this study, uses the optimal parameters for a maximum 30-second nonoverlapping epoch of activity per minute.

D=0.0001(50A_-4_+30A_-3_+14A_-2_+28A_-1_+12lA_0_+8A_+1_+50A_+2_)

Here, if D<1, the epoch is scored as sleep, while if D>=1, the epoch is scored as wake. The 0.001 represents the scale factor for the entire equation, while the numerical values assigned to each epoch represent the weighting factors for the present, previous, and following epochs. A represents an epoch, where A_-4_ to A_-1_ represent the four preceding epochs to the current epoch (A0) and A_+1_ to A_+2_ represent the following 2 epochs.

#### University of California, San Diego Scoring Algorithm (UCSD)

Developed by Jean-Louis et al [9], this actigraphy algorithm is similar to the Cole-Kripke algorithm, with the only difference being the weights on the epoch. The UCSD algorithm was also designed for minute-by-minute epochs. However, the authors noted that two 30-second epochs were counted as 1 minute to match their polysomnography data recording rates. In our case, we gave this algorithm the original 30-second epoch data, treating each epoch in the formula as is. The UCSD algorithm used in this study was as follows:

D=0.05(0.010A_-4_+0.015A_-3_+0.028A_-2_+0.031A_-1_+0.085A_0_+0.015A_+1_+.010A_+2_)

where, if D<1, the epoch is scored as sleep, while if D≥1, the epoch is scored as wake. According to previous analyses of Actillume data for healthy young adults, the optimal scaling factor, *P,* was .05 [9]. The numerical values assigned to each epoch represent the weighting factors for the present, previous, and following epochs. A represents an epoch, where A_-4_ to A_-1_ represent the four preceding epochs to the current epoch (A_0_) and A_+1_ to A_+2_ represent the following two epochs.

#### Kripke 2010 (K2010)

Developed by Kripke et al and applied through Microsoft Excel Visual Basic macro, this algorithm aimed to set optimal parameters for a sleep, wake scoring algorithm to score each epoch. This algorithm is similar to the aforementioned algorithms [10]. However, the optimal algorithm accounts for the activity counts of 13 30-second epochs; the 10 epochs preceding and 2 epochs proceeding the epoch being scored (X0) and differs in the weights assigned to each epoch. The optimal algorithm by Kripke et al [10] used in this study was as follows:

D=0.30(0.0064X_-10_+0.0074X_-9_+0.0112X_-8_+0.0112X_-7_+0.0118X_-6_+0.0118X_-5_+0.0128X_-4_+0.0188X_-3_+0.0280X_-2_+0.0664X_-1_+0.0300X_0_+0.0112X_+1_+.100X_+2_)

where D was the scaled polynomial sum of activity scores for 13 30-second epochs. The 0.30 represents the optimal overall scaling parameter. The numerical value attached to each epoch (X) represents a scaling parameter for each respective corresponding epoch. When D<1, the epoch being scored (X_0_) is scored as sleep, while if D≥1 according to this algorithm.

#### Philips-Respironics (Philips)

This algorithm is designed for Philips-Respironics and previously Mini-Mitter Co. Inc. devices known as Actiwatch [37,38]. This algorithm is designed to handle data from the Actiwatch monitors, which measure activity levels in several epoch lengths of 15 seconds, 30 seconds, 1 minute, or 2 minutes. In our study, we used the 30-second version of this algorithm. Similar to the aforementioned algorithms, this also applies weights to 5 epochs, the 2 preceding and 2 proceeding epochs, and the epoch being scored. The algorithm used in this study is as follows:

A=0.04E_-4_+0.04E_-3_+0.2E_-2_+0.2E_-1_+2E_0_+0.2E_+1_+.2E_+2_+.04E_+3_+.04E_+2_

Based on activity counts measured through the device, a total activity value (A) is generated for each epoch. The total activity value is then evaluated against the wake threshold value (20 [low], 40 [medium], 80 [high]), which, in the Actiwatch software, is automatically generated based on the activity data of individual cases or a custom value selected by the user [39]. If the total activity value is less than or equal to the wake threshold value, the epoch is scored as sleep. That is, if A>T, the epoch is scored as wake; otherwise, if A ≤ T, the epoch is scored as sleep. The En represented the activity counts of the previous, successive, or scored epoch. For our purposes, we evaluated each threshold after processing.

#### Sadeh

Sadeh et al [11] developed an algorithm based on concurrent polysomnography and a wrist actigraph (Ambulatory Monitoring, Ardsley). The algorithm features a discriminant function using 5 calculated variables on an 11-minute window (the 5 preceding, 5 proceeding, and the scored epochs), centered on the epoch being scored [11]. Any missing epochs are considered 0 to avoid infinity problems. This happens if the current epoch is at the beginning or end of a dataset. The Sadeh algorithm uses the y-axis epoch data. If any of the epoch counts are over 300, it reduces them to 300. The original formula for this algorithm uses a 1-minute epoch window; in this study, we gave this algorithm the 30-second epoch raw actigraphy data. Each 30-second epoch was evaluated as a 1-minute epoch. This would preserve the comparison between algorithms, as all of them processed the 30-second raw actigraphy data. The original Sadeh algorithm used in this study is as follows:

PS = (7.601 - [0.065 * AVG] - [1.08 * NATS] - [0.056 * SD] - [0.703 * LG])

where AVG is the arithmetic mean (average) of the activity counts for the window, NATS is the number of epochs that have counts ≥50 and <100, SD is the standard deviation for the first 6 epochs of the window, and LG is the natural (base e) logarithm of the current epoch. Post scoring of the epoch, if the resultant value, referred to as probability of sleep (PS), is ≥ 0, the epoch is scored as sleep. Based on the original paper, typically, if the result is > −4, then the current epoch is considered asleep.

#### Webster’s Rescoring Rules (RS)

Notably, traditional actigraphy algorithms often incorrectly score periods of wake as sleep or rest. To counter this issue, Webster et al [40] developed a set of rescoring rules to apply after initial classification using a scoring algorithm [6,22,40]. These results have been well cited in the literature and given their relevance in increasing algorithm accuracy, they were an important consideration to evaluate within this study [22]. These were applied in unison with the algorithms and compared with nonrescored results. Once the data were processed with each respective algorithm, the raw activity scores were binarized, reflecting sleep (1) or wake (0) using optimal thresholds (see data analysis), and rescoring was sequentially applied to the binarized data. Nonrescored results were denoted as NRS, and rescored results were as RS. The rescoring rules were as follows:

1. After at least 4 minutes scored as wake, the next 1 minute scored as sleep is rescored as wake
2. After at least 10 minutes scored as wake, the next 3 minutes scored as sleep are rescored as wake
3. After at least 15 minutes scored as wake, the next 4 minutes scored as sleep are rescored as wake
4. If 6 minutes or less are scored as sleep surrounded by at least 10 minutes (before and after) scored as wake are rescored as wake
5. If 10 minutes or less are scored as sleep surrounded by at least 20 minutes (before and after) scored as wake, they are rescored as wake

### Statistical Analysis

#### Epoch-by-Epoch Comparison

Once the data were processed with each respective algorithm, the raw activity scores were examined. We conducted an epoch-by-epoch comparison of sleep-wake for each algorithm against the corresponding ground truth polysomnography measurements. In our analysis, the polysomnography classification was considered the actual class, while the actigraphy classification was considered the predicted class (with wake=0 and sleep=1). We used confusion matrix analyses to assess key metrics accuracy, sensitivity, specificity, precision, and *F*_1_-score (a measure of an algorithm’s predictive performance of sleep and wake). A complete definition of metrics is provided in Multimedia Appendix 2.

Epoch-by-epoch comparison was conducted for each algorithm based on the original sleep-wake thresholds defined in the respective papers (see Actigraphy Algorithm Section). The Philips-Respironics algorithm has 3 suggested threshold values (20 [low], 40 [medium], 80 [high]), all of which were examined [39]. In addition, weighted mean and SD results were generated for each algorithm both with and without rescoring.

#### Cohen κ and Matthews Correlation Coefficient

As measures of comparison between sleep algorithms and polysomnography, Cohen κ and MCC were calculated for each algorithm. As with the confusion matrix statistics, the sleep algorithm results (predicted) were evaluated in comparison to the ground truth polysomnography (actual). Cohen κ is a measure of agreement between raters for nominal or categorical data with adjustments for chance agreement [41,42]. For this study, the raters were individual sleep algorithms and polysomnography. MCC was also used to evaluate the correlation (performance) between binary classification, such as in our case between sleep algorithms and polysomnography [43,44]. A complete definition of metrics is provided in Multimedia Appendix 2.

#### Repeated Measures ANOVA With Post Hoc Examination

As the same sample of participants was assessed by each algorithm, repeated measures ANOVA was conducted to evaluate whether there were respective differences between sleep algorithm results for Cohen κ, MCC, and confusion matrix. The assumption of sphericity was violated across all results based on significant Mauchly tests and low epsilon values. Therefore, Greenhouse-Geisser corrections were applied, and adjusted degrees of freedom and *P* values were evaluated. For significant ANOVA results, subsequent post hoc analyses were conducted with Bonferroni correction (controlling for type 1 error) to evaluate which algorithm pairs demonstrated significant differences and their effect sizes (Hedges *g*) [45,46]. For Hedges *g* interpretation, the range of 0.2, 0.5, 0.8 for small, medium, and large effect sizes was considered.

#### Receiver Operating Characteristic Curve and Area Under the Curve (ROC; AUC)

To comparably evaluate the ability of each algorithm to classify sleep-wake, we evaluated the area under the receiver operating characteristic curves (AUC and ROC), respectively. AUC provides information about algorithm performance (ie, how much is each algorithm capable of distinguishing sleep vs wake) as a measure of global accuracy and robustness. An ROC curve is generated by plotting the sensitivity (also known as the true positive rate; TPR) against 1 - specificity (also known as the false positive rate; FPR) at various cut-off thresholds. We used raw values for actigraphy and binary ground truth (polysomnography) to generate the ROC curve. Using the s*cikit-learn* Python library, an automatic extensive range of thresholds was covered for the ROC curve. Both ROC curves and AUC were generated for each algorithm. Given that the original algorithms reported a fixed, predetermined threshold for sleep-wake, the optimal cut-off point was not examined in the ROC curves. However, the sensitivity and specificity for each algorithm relative to their sleep-wake threshold were reported in the epoch-by-epoch results. A complete definition of metrics is provided in Multimedia Appendix 2.

#### Rescored Algorithm Analysis

To evaluate whether applying Webster et al [40] rescoring rules improved algorithm performance in contrast to nonrescored actigraphy algorithms, we applied the rescoring rules to each algorithm [40]. To do so, we first binarized (sleep [1], wake [0]) the raw activity values generated by each algorithm using the optimal thresholds determined in the ROC analyses. We then applied the rescoring rules to the binary data. Finally, we calculated the Cohen κ, MCC, and all confusion matrix results for the rescored algorithms, respectively. These results were compared to the nonrescored results.

#### Sleep Metrics Comparison

We examined each algorithm’s agreement with polysomnography on several commonly studied sleep metrics, including TST, SE, and WASO. For the polysomnography, we evaluated the MESA and NSRR-provided data for each sleep metric, while for the actigraphy data, once each dataset was processed by each algorithm and binarized, we calculated each sleep metric for both the nonrescored and rescored data. We used the MESA and NSRR definitions to calculate the respective sleep metrics. To do so, we applied the following calculations:

1. TST=the total number of minutes of sleep from the first sleep onset time to sleep offset time.
2. SE=TST/in-bed (results presented as %).
  - In-bed=synchronized to polysomnography in-bed start time, the interval between lights out and in-bed time versus lights off, out-bed, or wake time.
3. WASO=the total duration of wake between first sleep onset time and sleep offset time.

These sleep metrics were calculated for each participant and each algorithm, respectively. For comparison of agreement, we used Bland-Altman distributions [47]. Bland-Altman distributions are used to visualize the difference between actigraphy and polysomnography. We evaluated the average difference between corresponding measurements (actigraphy and polysomnography) for each respective sleep metric. Through these plots, we examined the distribution of differences between actigraphy and polysomnography, that is, the consistency between the 2. In addition, we examined the mean difference (actigraphy-polysomnography), bias, limits of agreement (LoA), and proportional bias (across values; regression) between actigraphy and polysomnography. The Bland-Altman distribution analysis was also conducted in Python using the pyCompare library with code assistance provided by ChatGPT [28].

### Ethical Considerations

The current study was conducted on a pre-established MESA dataset on a secondary use basis [20,21]. The original MESA study obtained institutional review board ethics approval for each study site of data collection and obtained written consent from all participants [20]. The NSRR resource maintains a detailed approval procedure for dataset acquisition and use [21]. The study dataset was acquired through a data access and use agreement with the NSRR [21]. All data were predeidentified for privacy by NSRR. Ethics approval was acquired from the University of Toronto (REB protocol # 35344) for this study.

## Results

The sample demographics results are presented in Table 1. Notably, 12.3% (n=177, includes Apnea, Insomnia RLS, and CPAP subgroup) of sample participants reported some form of sleep problem. Additionally, measures of hypertension, diabetes, and other disorders were not reported in the NSRR version of the MESA dataset. However, previous studies note the prevalence of health issues or health risks within this study sample [20,48,49].

**Figure 2.**
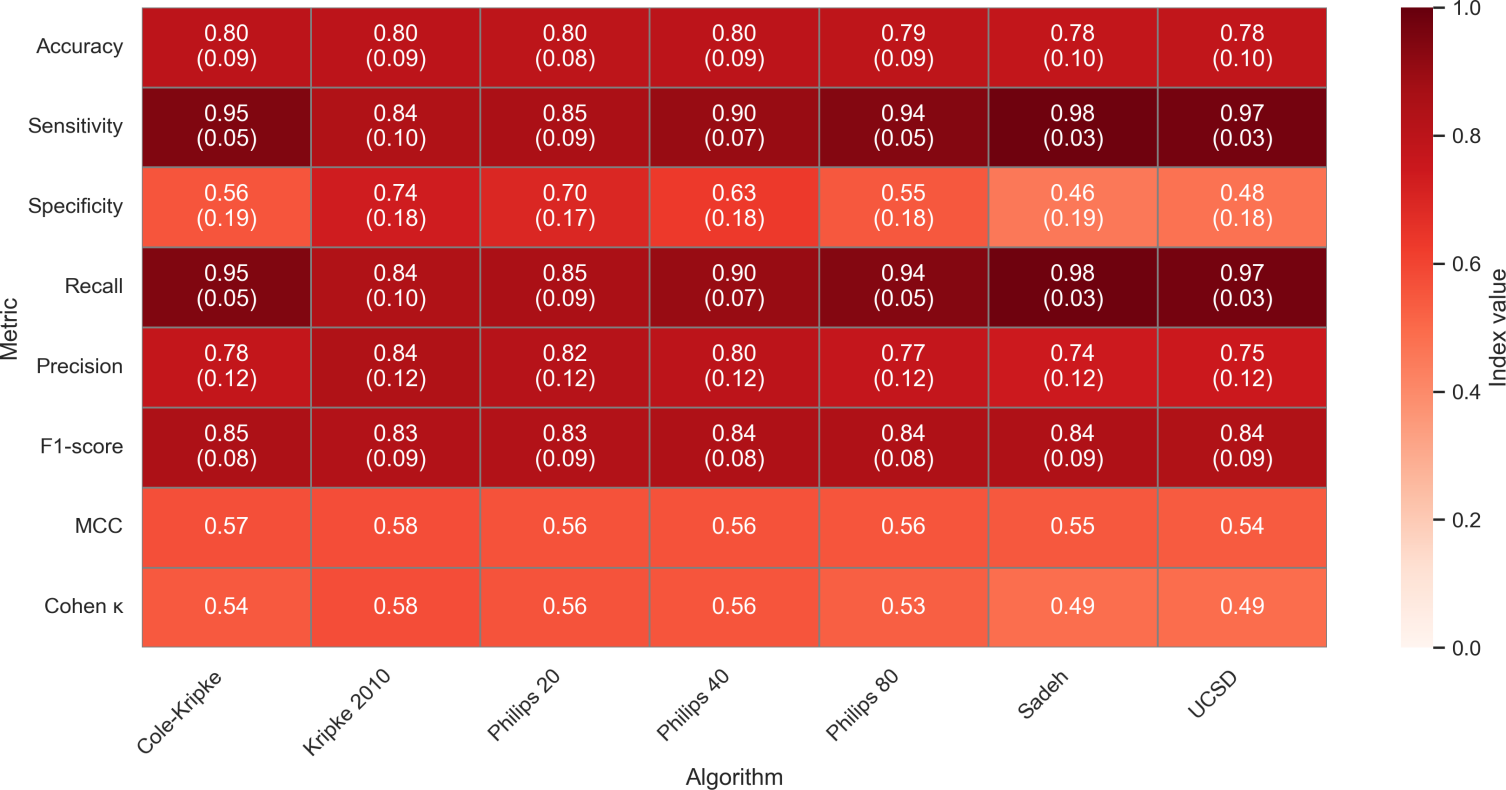
Cohen κ, Matthews correlation coefficient, and confusion matrix heatmap results for nonrescored algorithms. Accuracy, sensitivity, specificity, precision, *F*_1_-score: mean (SD). Color tone represents index value. MCC: Matthews correlation coefficient; UCSD: University of California San Diego.

The epoch-by-epoch comparison for nonrescored algorithms demonstrated that the Kripke 2010 algorithm (mean 0.80, SD 0.09) had the highest accuracy, followed by Cole-Kripke (mean 0.80, SD 0.09), Philips-Respironics (mean 0.80, SD 0.08; mean 0.80, SD 0.09; and mean 0.79, SD 0.09; for 20, 40, and 80 thresholds), UCSD (mean 0.78, SD 0.10), and Sadeh (mean 0.78, SD 0.10) respectively (Figure 2). An important note is that there were not any large differences between traditional algorithms for accuracy, ranging only 0.01‐0.02. *F*_1_-scores for the nonrescored algorithm results were all >0.80. Results for Cohen κ demonstrated moderate agreement (0.4‐0.6) between actigraphy algorithms and polysomnography. Similarly, MCC results showed moderate positive correlations (range 0.49‐0.58) between actigraphy algorithms and polysomnography for all algorithms. Complete results for Cohen κ, MCC, accuracy, sensitivity, specificity, precision, and *F*_1_-scores are presented in Figure 2.

**Figure 3.**
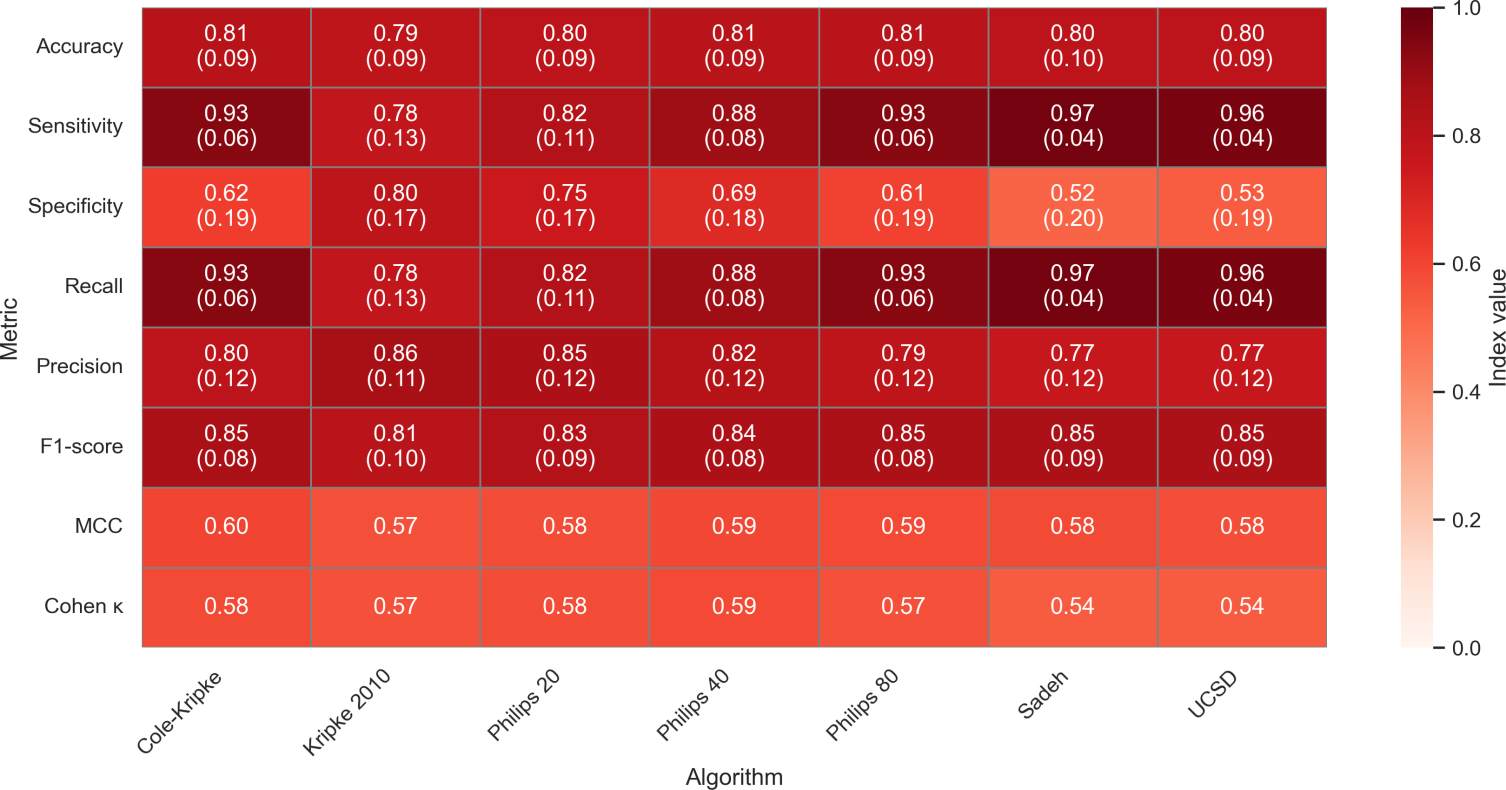
Cohen κ, Matthews correlation coefficient, and confusion matrix heatmap results for rescored algorithms. Accuracy, sensitivity, specificity, precision, *F*_1_-score: mean (SD). Color tone represents index value. MCC: Matthews correlation coefficient; UCSD: University of California San Diego.

Examination of rescored algorithm results revealed marginal changes observed in accuracy. Rescored results demonstrated the Cole-Kripke (mean 0.81, SD 0.09) had the highest accuracy followed by Philips-Respironics (mean 0.80, SD 0.09; mean 0.81, SD 0.09; and mean 0.81 SD 0.09; for 20, 40, and 80 thresholds), Sadeh (mean 0.80, SD 0.10), UCSD (mean 0.80, SD 0.09); and Kripke 2010 (mean 0.79 SD 0.09) respectively (Figure 3). Again, there were no large differences between traditional algorithms for ACC, ranging only 0.01‐0.03. Rescored results for *F*_1_-scores either demonstrated no change or marginally changed by 0.01‐0.02 for some algorithms, with all still >0.80. Results for Cohen κ again demonstrated minor changes remaining within the moderate agreement range between actigraphy algorithms and polysomnography. Similarly, MCC results only showed minor changes again, showcasing moderate positive correlations (range 0.54‐0.60) between actigraphy algorithms and polysomnography for all algorithms. Complete rescored results for Cohen κ, MCC, accuracy, sensitivity, specificity, precision, and *F*_1_-scores are presented in Figure 3. Complete confusion matrix statistical results for both nonrescored and rescored algorithms are provided in Multimedia Appendix 3.

Repeated measures ANOVA testing revealed significant differences between algorithms across all metrics (κ, MCC, accuracy, sensitivity, specificity, precision, and *F*_1_-score). Complete ANOVA results are presented in Tables2  3 for nonrescored and rescored algorithms, respectively. Subsequent post hoc analyses also revealed significant differences between algorithm pairs across all metrics (Tables4  5).

**Table 2.** Repeated measures ANOVA for confusion matrix across nonrescored algorithms.

Metric	SS^a^	MS^b^	*F* test	*df*	η²g^c^	Eps (ε)	*P* value (uncorr.)	df^d^ (adjusted)	*P* value (adjusted)
Accuracy			78.14		0.01	0.19	<.001		<.001
Accuracy_algorithm_	0.50	0.08		6				1.15	
Accuracy_error_	9.22	0.00		8628				1656.37	
Sensitivity			3754.96		0.39	0.20	<.001		<.001
Sensitivity_algorithm_	27.60	4.60		6				1.19	
Sensitivity_error_	10.55	0.00		8616				1711.96	
Specificity			8112.40		0.23	0.22	<.001		<.001
Specificity_algorithm_	99.06	16.51		6				1.34	
Specificity_error_	17.56	0.00		8628				1920.73	
Precision			3435.68		0.07	0.20	<.001		<.001
Precision_algorithm_	10.55	1.76		6				1.22	
Precision_error_	4.42	0.00		8628				1751.47	
*F*_1_-score			93.31		0.00	0.20	<.001		<.001
*F*_1_-score_algorithm_	0.36	0.06		6				1.17	
*F*_1_-score_error_	5.58	0.00		8628				1688.29	

a SS: sum of squares.

b MS: mean squares.

c η²g: general eta squared

d Adjusted values for *df* and *P* values refer to respective Greenhouse-Geisser corrections.

**Table 3.** Repeated measures ANOVA for confusion matrix across nonrescored algorithms.

Metric	SS^a^	MS^b^	*F* test	*df*	η²g^c^	Eps (ε)	*P*_uncorr._ value	df_adjusted^d^_	*P*_adjusted_ value
Accuracy			64.62		0.01	0.20	<.001		<.001
Accuracy_algorithm_	0.56	0.09		6				1.22	
Accuracy_error_	12.56	0.00		8628				1754	
Sensitivity			3906.50		0.40	0.21	<.001		<.001
Sensitivity_algorithm_	44.24	7.37		6				1.24	
Sensitivity_error_	16.26	0.00		8616				1782.96	
Specificity			5961.01		0.22	0.26	<.001		<.001
Specificity_algorithm_	97.56	16.26		6				1.55	
Specificity_error_	23.54	0.00		8628				2227.53	
Precision			2706.59		0.08	0.23	<.001		<.001
Precision_algorithm_	11.65	1.94		6				1.37	
Precision_error_	6.19	0.00		8628				1974.34	
*F*_1_-score			341.85		0.03	0.20	<.001		<.001
*F*_1_-score_algorithm_	2.20	0.37		6				1.22	
*F*_1_-score_error_	9.26	0.00		8628				1759.31	

a SS: sum of squares.

b MS: mean squares.

c η²g: general eta squared.

d Adjusted values for df and *P* values refer to respective Greenhouse-Geisser corrections.

**Table 4.** Repeated measures ANOVA for Matthews correlation coefficient and Cohen κ across nonrescored algorithms.

Metric	SS^a^	MS^b^	*F* test	*df*	η²g^c^	Eps (ε)	*P*_uncorr._ value	*df*_adjusted^d^_	*P*_adjusted^d^_ value
MCC			138.47		0.01	0.23	<.001		<.001
MCC_algorithm_	2.01	0.34		6				1.38	
MCC_error_	20.91	0.00		8628				1988.02	
K			450.81		0.02	0.21	<.001		<.001
K_algorithm_	9.26	1.54		6				1.27	
K_error_	29.53	0.00		8628				1828.56	

a SS : sum of squares.

b MS: mean squares.

c η²g: general eta squared.

d Adjusted values for df and *P* values refer to respective Greenhouse-Geisser corrections.

**Table 5.** Repeated measures ANOVA for Matthews correlation coefficient and Cohen κ across nonrescored algorithms.

Metric	SS^a^	MS^b^	*F* test	*df*	η²g^c^	Eps (ε)	*P*_uncorr._ value	*df*_adjusted^d^_	*P*_adjusted^d^_ value
MCC			37.17		0.00	0.25	<.001		<.001
MCC_algorithm_	0.72	0.12		6				1.53	
MCC_error_	27.85	0.00		8628				2196.75	
K			112.84		0.01	0.23	*P*<.001		<.001
K_algorithm_	3.21	0.53		6				1.38	
K_error_	40.86	0.00		8628				1977.95	

a SS: sum of squares.

b MS: mean squares.

c η²g: general eta squared.

d Adjusted values for df and *P* values refer to respective Greenhouse-Geisser corrections.

Post hoc analyses for MCC were significant; however, all effect sizes were very small to small (all Hedges *g*<0.3). Post hoc results for Cohen κ demonstrated small to medium effects with effect sizes ranging from 0 to <0.5. Complete post hoc analysis results are presented in Tables 4 and 5. Notably, modest differences were observed between K2010 and UCSD (Hedges *g*=0.42) and K2010 and Sadeh (Hedges *g*=0.44), indicating better predictive performance of the K2010 algorithm, respectively. Similarly, significant post hoc results for rescored algorithms for both MCC and Cohen κ showed very small to small effect sizes across all algorithm contrasts (all Hedges *g*<0.25). Detailed results are provided in Multimedia Appendix 4.

Post hoc analyses for nonrescored algorithms' confusion matrix metrics, particularly accuracy and *F*_1_-scores, demonstrated both nonsignificant and many significant contrasts (Multimedia Appendix 5). Significant pairs, however, had very small to small effect sizes (Hedges *g*) ranging between 0‐0.3 for both accuracy and *F*_1_-scores (Multimedia Appendix 5). Post hoc results for sensitivity, specificity, and precision varied greatly, ranging from very small to large effect between contrasts, revealing nuanced performance differences (Multimedia Appendix 5). For detailed post hoc results, see Multimedia Appendix 5.

After rescoring, post hoc results for confusion matrix metrics demonstrated similar very small to small effect sizes for accuracy. However, there were some medium effect sizes for *F*_1_-scores. Notably, modest differences were observed across K2010 predictive performance, which was worse than the CK (Hedges *g*=−0.46), Sadeh (Hedges *g*=−0.42), UCSD (Hedges *g*=−0.40), and Philips algorithm with a threshold of 80 (Hedges *g*=−0.42). Similarly, post hoc results for sensitivity, specificity, and precision varied greatly, ranging from very small to large effects between contrasts, revealing nuanced performance differences (Multimedia Appendix 5). For full post hoc results, see Multimedia Appendix 5.

**Figure 4.**
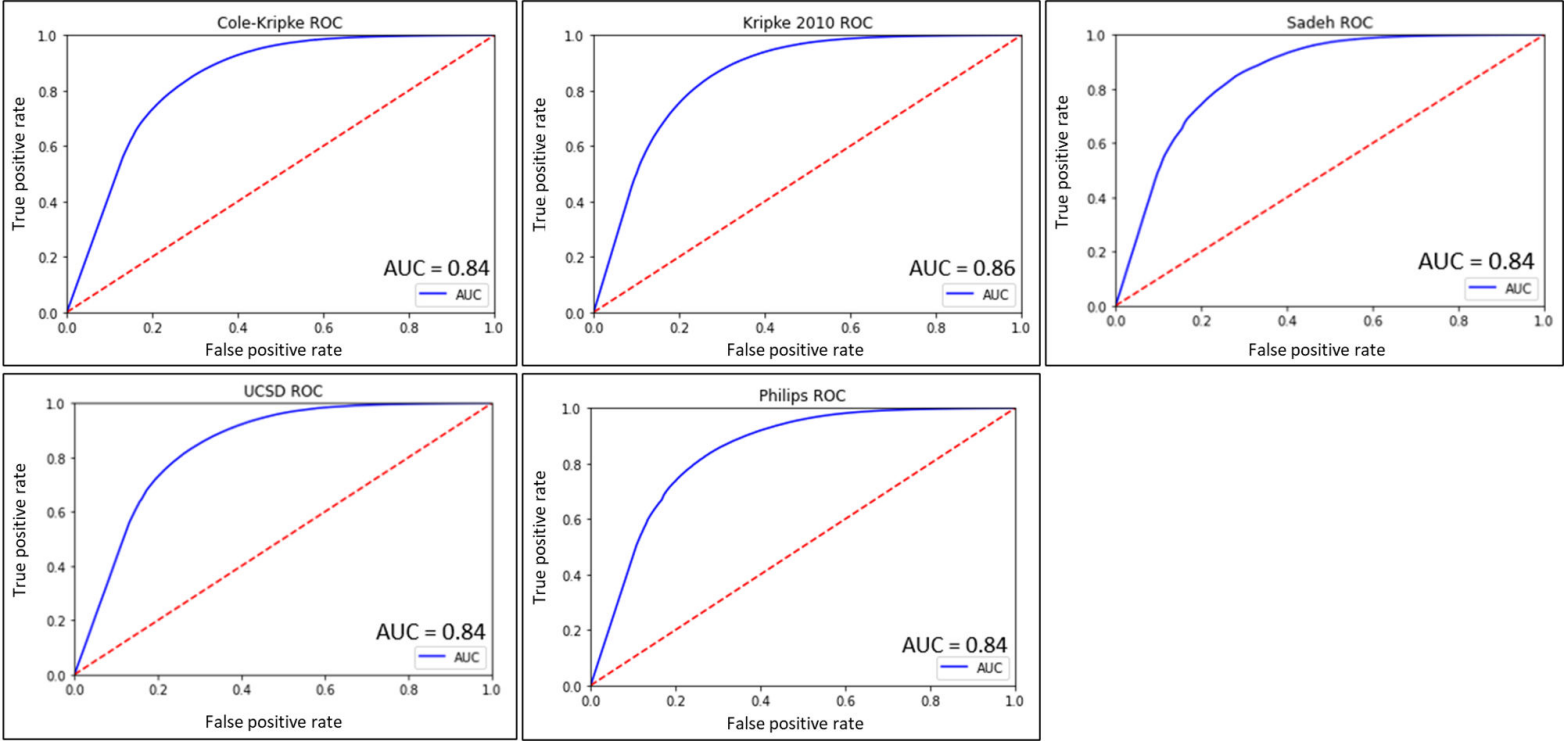
Algorithm receiver operating characteristic curves and area under the curve. Receiver operating characteristics curves and respective area under the curve listed for each algorithm. AUC: area under the curve; ROC: receiver operating characteristics curve;

The ROC analysis revealed that the Kripke 2010 had the highest AUC (0.86) followed by Sadeh (0.85), Philips-Respironics (0.84), Cole-Kripke (0.84), and UCSD (0.84). Across the board, all algorithms demonstrated excellent ability to discriminate sleep-wake (excellent performance). The ROC curves are presented with their respective AUC in Figure 4.

**Figure 5.**
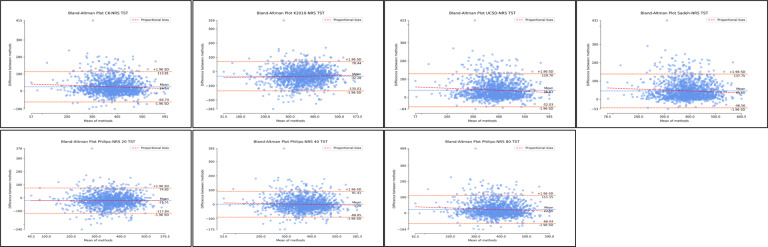
Bland-Altman distributions total sleep time for nonrescored algorithms. Plots represent the Bland-Altman distributions for nonrescored algorithms of actigraphy in comparison to polysomnography (ground truth) for total sleep time. TST: total sleep time.

Examination of agreement for TST through Bland-Altman distributions demonstrated significant levels of mean difference and systematic bias across all algorithms (Figure 5; Multimedia Appendix 6; Table 1). This may be due to the sample or due to clear outliers. These outliers may also have caused discrepancies within the distribution and mean difference. In addition, supplementary regression analyses for proportional bias were significant across nonrescored Cole-Kripke, UCSD, Sadeh, and Philips threshold 80 algorithms. Similarly, rescored Kripke 2010, UCSD, Sadeh, Philips threshold 20, and Philips threshold 80 algorithms also demonstrated significant proportional bias. Although regression tests were significant, both the regression slopes and *R*^2^ values were extremely small, ranging from −0.06 to 0.10 for slope and *R*^2^ values all ≤0.01. Although proportional bias regression analyses were significant for a number of algorithms for TST, both slopes and *R*^2^ were extremely small, and plot distributions were relatively evenly spread. Therefore, effects of proportional bias may be minimal or significant due to sample size. Detailed proportional bias regression statistics are reported in Multimedia Appendix 6, Table 1.

**Figure 6.**
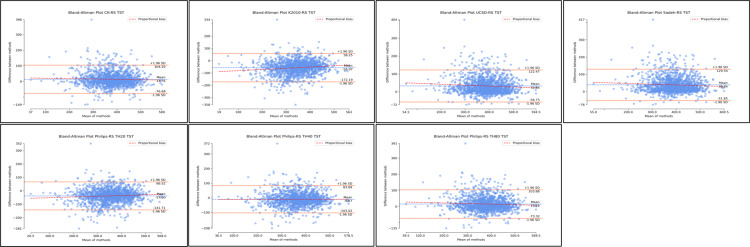
Bland-Altman distributions total sleep time for rescored algorithms. Plots represent the Bland-Altman distributions for rescored algorithms of actigraphy in comparison to polysomnography (ground truth) for total sleep time. TST: total sleep time.

The best results for TST agreement with polysomnography across all algorithms were obtained with the Philips algorithm with the threshold 40 (nonrescored: mean difference [MD] 1.28, SD −46.00; 95% LoA −88.88 to 91.44; Figure 5; Multimedia Appendix 6; Table 1). These results were considerably decreased with rescoring; however, the Philips algorithm with the threshold 40 retained the best agreement (rescored: MD −9.67, SD –47.64; 95% LoA −103.05 to 83.71; Figure 5; Multimedia Appendix 6; Table 1). For TST, the Cole-Kripke, UCSD, and Sadeh algorithms displayed higher mean differences in TST measurements by actigraphy (Figure 5; Multimedia Appendix 6; Table 1). This overestimation of TST was reduced with rescoring (Figure 6; Multimedia Appendix 6; Table 1). Conversely, the Kripke 2010 algorithm underestimated the TST, and the magnitude of underestimation increased with rescoring. For the Philips algorithm, the results varied based on which threshold was used. The Philips algorithm with an 80 threshold overestimated TST when compared to polysomnography; however, this improved with rescoring. Finally, the Philips algorithm with 20 thresholds demonstrated considerable underestimation for TST, which further worsened with rescoring.

**Figure 7.**
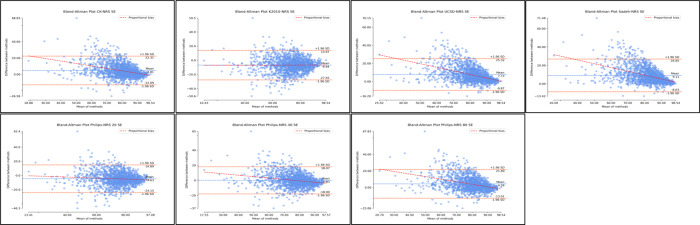
Bland-Altman distributions sleep efficiency for nonrescored algorithms. Plots represent the Bland-Altman distributions for nonrescored algorithms of actigraphy in comparison to polysomnography (ground truth) for sleep efficiency. SE: sleep efficiency.

With respect to SE, all algorithms demonstrated similar distributions where estimates of SE were worse at lower levels of SE (Figure 7; Multimedia Appendix 6; Table 2). Considerable improvements in agreement were observed as SE levels increased. Based on the Bland-Altman plots, the distributions demonstrate some systematic bias and heteroscedasticity (Figure 7; Multimedia Appendix 6; Table 2). Supplementary regression analyses for proportional bias were significant across all nonrescored and rescored algorithms with the exception of nonrescored Kripke 2010 (*P*=.86). Regression slopes covered a wide range and were all negative with the exception of rescored Kripke 2010 (slope=0.15). Therefore, indicating a directional bias as SE levels change (in particular, increased). However, *R*^2^ values were relatively small, with all values ≤0.21. Detailed proportional bias regression statistics are reported in Multimedia Appendix 6; Table 2.

**Figure 8.**
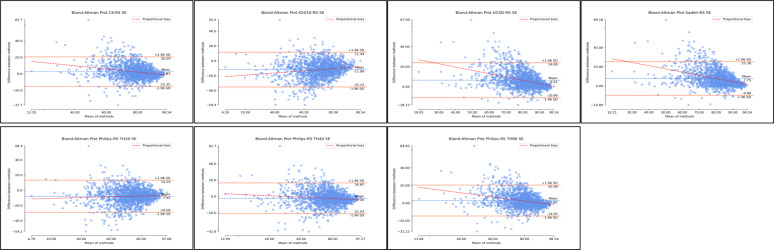
Bland-Altman distributions sleep for rescored algorithms. Plots represent the Bland-Altman distributions for rescored algorithms of actigraphy in comparison to polysomnography (ground truth) for sleep efficiency. SE: sleep efficiency.

The Philips algorithm with a threshold at 40 demonstrated the best agreement with polysomnography (nonrescored: MD 0.03, SD −9.20; 95% LoA −18 to 18.07). Rescoring of this resulted in underestimation of SE (rescored: MD −2.20, SD −9.61; 95% LoA −21.03 to 16.63). The Cole-Kripke, UCSD, Sadeh, and Philips threshold 80 algorithms all demonstrated overestimation of SE, with some improvement with rescoring (Figure 8; Multimedia Appendix 6; Table 2). While the Kripke 2010 and Philips threshold 20 algorithms both demonstrated underestimation of SE, with rescoring further increasing the MD, that is, the magnitude of underestimation (Figure 8; Multimedia Appendix 6; Table 2).

**Figure 9.**
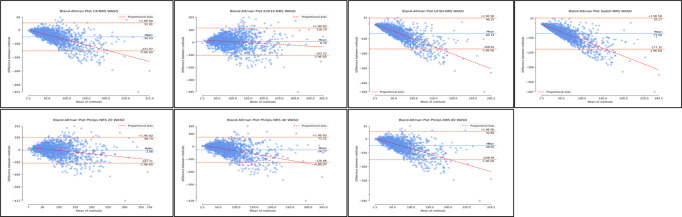
Bland-Altman distributions wake after sleep onset for nonrescored algorithms. Plots represent the Bland-Altman distributions for nonrescored algorithms of actigraphy in comparison to polysomnography (ground truth) for wake after sleep onset. WASO: wake after sleep onset.

Finally, the Bland-Altman distributions for WASO demonstrated considerable underestimation. The distributions demonstrate considerable bias and heteroscedasticity, where WASO estimates are better at lower WASO averages and become considerably worse as WASO increases (Figure 9; Multimedia Appendix 6; Table 3). Again, supplementary regression analyses for proportional bias were significant across all nonrescored and rescored algorithms. Regression slopes covered a wide range and were all negative with the exception of rescored Kripke 2010 (slope=0.08). Therefore, indicating a directional bias as WASO levels change (in particular, increased). *R*^2^ values covered a wide range. Detailed proportional bias regression statistics are reported in Multimedia Appendix 6, Table 3.

**Figure 10.**
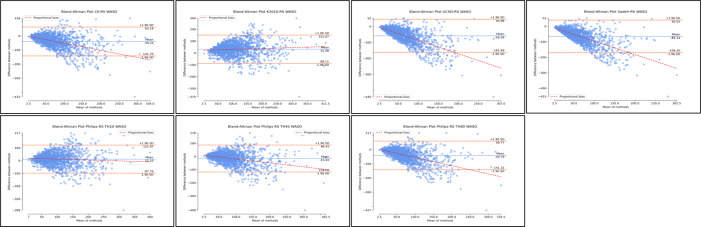
Bland-Altman distributions wake after sleep onset for rescored algorithms. Plots represent the Bland-Altman distributions for rescored algorithms of actigraphy in comparison to polysomnography (ground truth) for wake after sleep onset. RS: rescored algorithms; WASO: wake after sleep onset.

Among all the algorithms, the Philips threshold 20 algorithm had the best agreement with polysomnography for WASO, only underestimating by a small magnitude (nonrescored: MD=−3.98, SD −52.94; 95% LoA −107.74 to 99.77). Rescoring of this algorithm diminished the agreement of this algorithm to overestimate WASO (rescored: MD 12.10, SD −56.07; 95% LoA −97.80 to 122.01). With respect to the Philips algorithms at thresholds 40 and 80, both underestimated WASO. Rescoring lowered the magnitude of underestimations, improving agreement. Similarly, Coke-Kripke, UCSD, and Sadeh had large underestimations of WASO with some improvement after rescoring (Figure 10; Multimedia Appendix 6; Table 3) Kripke 2010, however, only slightly overestimated WASO, and rescoring considerably increased the magnitude of overestimation.

Exploratory subgroup analysis was conducted for 4 sleep problem groups, including apnea, individuals who used CPAP, insomnia, and RLS. For the subgroup analyses, we again examined AUC, accuracy metrics, and Bland-Altman distributions for agreement. The AUC was above 0.80 across all subgroups and algorithms, similar to the results across the entire population (Multimedia Appendix 7). This indicated all algorithms demonstrated excellent ability to discriminate sleep-wake regardless of the presence of individuals’ sleep problems.

Accuracy ranged between 76% and 81% across all algorithms and subgroups, with only minor differences of 1%‐4% between algorithms. Rescoring only resulted in minor improvements in algorithm accuracy of 1%‐3%. *F*_1_-scores demonstrated good balance, ranging from 0.80‐0.84. After rescoring, *F*_1_-scores remained relatively the same with marginal changes (0.01‐0.03) again remaining within the ok-to-good balance range of 0.79 to 0.85. Overall, accuracy and *F*_1_-score results were again highly similar to the results observed across the entire population sample. Complete subgroup results for accuracy, sensitivity, specificity, precision, and *F*_1_-score are presented in Multimedia Appendix 8.

Along these lines, MCC and Cohen κ results were extremely similar to the overall results, ranging between 0.40‐0.60 for both metrics and for both rescored and nonrescored algorithms. Therefore, all results demonstrated moderate positive correlations for MCC and moderate Cohen κ agreement across algorithms.

Repeated measures ANOVA analyses for accuracy were only significant for the apnea rescored algorithms (*F*_1.20,124.08_=5.27; ε=0.20; *P*=.02), RLS rescored algorithms (*F*_1.24,80.71_=5.70; ε=0.20; *P*=.01), and insomnia nonrescored algorithms (*F*_1.17,96.97_=3.85; ε=0.19; *P*=.05). All other nonaccuracy metrics demonstrated significant ANOVA for both nonrescored and rescored algorithms (Multimedia Appendix 9).

Similar to the full sample results, post hoc contrasts for accuracy across subgroups were very small to small (all Hedges *g* results <0.3). Post hoc contrasts for *F*_1_-score were very small to small, approaching medium effect sizes for nonrescored algorithms (all Hedges *g* results <0.35). *F*_1_-score contrast effect sizes for the rescored algorithm demonstrated diminished performance with medium effect sizes for the K2010 algorithm across all subgroups. Similarly, the Philips threshold 20 algorithm showed diminished performance with medium effect sizes within the CPAP and RLS groups and to a lesser extent (Hedges *g*<0.40) for the apnea and insomnia groups. Complete subgroup post hoc results are presented in Multimedia Appendix 9.

The apnea subgroup of the K2010 rescored algorithm demonstrated diminished performance compared to the CK (Hedges *g*=−0.52), UCSD (Hedges *g*=−0.49), Sadeh (Hedges *g*=−0.50), Philips threshold 80 (Hedges *g*=−0.47), and Philips threshold 40 (Hedges *g*=−0.37). The Philips threshold 20 also demonstrated worse performance with small, approaching medium effect sizes compared to the CK, UCSD, Sadeh, and Philips threshold 80 algorithms (ranging from (Hedges *g*=−0.33 to −0.37). Within the CPAP subgroup, rescored K2010 showed worse performance than CK (Hedges *g*=−0.53), UCSD (Hedges *g*=−0.51), Sadeh (Hedges *g*=−0.53), Philips threshold 80 (Hedges *g*=−0.47), and Philips threshold 40 (Hedges *g*=−0.35). The Philips threshold 20 algorithm again demonstrated worse performance with small, approaching medium effect sizes compared to the CK, UCSD, Sadeh, and Philips threshold 80 algorithms (ranging from (Hedges *g*=−0.37 to −0.43). To a lesser extent, within the insomnia subgroup, comparisons of algorithms were similar to the apnea group. In addition, both K2010 and Philips threshold demonstrated small, approaching medium effect sizes for Philips threshold 20 algorithms. Similarly, within the RLS subgroup, the K2010 rescored algorithm demonstrated diminished performance compared to the CK (Hedges *g*=−0.48), UCSD (Hedges *g*=−0.49), Sadeh (Hedges *g*=−0.53), Philips threshold 80 (Hedges *g*=−0.46), and Philips threshold 40 (Hedges *g*=−0.35). The Philips threshold 20 also demonstrated worse performance with small, approaching medium effect sizes compared to the CK, UCSD, Sadeh, and Philips threshold 80 algorithms (ranging from Hedges *g*=−0.33 to −0.40). Overall, the post hoc test revealed a wide range of effect sizes ranging from very small to large for sensitivity, specificity, and precision across all subgroups and algorithms. Complete subgroup post hoc results presented in Multimedia Appendix 9.

Repeated measures ANOVA analyses for MCC and Cohen κ also demonstrated a significant difference between algorithms for a variety of sleep problem subgroups. Both the apnea and insomnia subgroups demonstrated significant differences between algorithms for Cohen κ in both nonrescored (Apnea: *F*_1.24,127.66_=17.28; ε=0.21; *P*<.001; Insomnia: *F*_1.32,109.90_=28.14; ε=0.22; *P*<.001) and rescored algorithms (Apnea: *F*_1.31,134.72_=4.63; ε=0.22; *P*=.02; Insomnia: *F*_1.42,117.76_=5.94; ε=0.24; *P*=.008) and for MCC between nonrescored algorithms (Apnea: *F*_1.31,135.07_=4.51; ε=0.22; *P*=.03; Insomnia: *F*_1.50,124.90_=9.19; ε=0.25; *P*<.001) . Both CPAP and RLS subgroups showed significant differences between algorithms for only Cohen κ and only for nonrescored algorithms (CPAP: *F*_1.23,74.80_=8.31; ε=0.20; *P*=.003; RLS: *F*_1.24,80.75_=12.28; ε=0.21; *P*<.001). Complete subgroup ANOVA results within Multimedia Appendix 10.

A subsequent post hoc test revealed small effect sizes for MCC (Hedges *g*<0.25) for both the apnea and insomnia subgroups across nonrescored algorithm contrasts. With respect to Cohen κ, effect sizes ranged from very small to small for apnea in the subgroup across most nonrescored and algorithm contrasts (Multimedia Appendix 10). The exception was the nonrescored K2010 algorithm which demonstrated small-approaching-medium effects, having better Cohen κ agreement when compared with nonrescored UCSD and Sadeh algorithms (Multimedia Appendix 10). Similarly, to a lesser extent, within both CPAP and RLS subgroups, nonrescored K2010 had better Cohen κ agreement when compared with nonrescored UCSD and Sadeh algorithms (Hedges *g* range 0.27‐0.31). Complete subgroup post hoc results are presented in Multimedia Appendix 10.

Finally, the Bland-Altman distributions were examined for each algorithm, without and with rescoring applied, for 3 sleep metrics (TST, SE, and WASO) within each of the 4 subgroups (Multimedia Appendix 11). Overall, the subgroup results were again highly similar to those observed across the entire population sample. All algorithms demonstrated considerable mean difference and systematic bias for each subgroup analysis (Multimedia Appendix 11). Overall, rescoring did not improve agreement between the algorithm and ground truth, with some cases demonstrating poorer agreement after rescoring. Similar trends of bias were observed as the results from the entire dataset sample. For SE, all algorithms demonstrated heteroscedasticity for each subgroup (ie, estimates of SE were worse from polysomnography as SE decreased). The exception was the RLS group, which demonstrated random distributions for all algorithms. For WASO, all algorithms showed heteroscedasticity across all subgroups, where estimates of WASO were worse compared to polysomnography as WASO increased. For TST, there was no heteroscedasticity; that is, the distributions were random and evenly spread for all algorithms across all subgroups. For detailed Bland-Altman distribution statistics, see Multimedia Appendix 11.

## Discussion

### Principal Findings

Using the large MESA dataset, we established that traditional actigraphy algorithms can, with considerable accuracy, classify sleep and wake. As a first, this study comprehensively provides detailed benchmarks of traditional actigraphy algorithms. This study also provides the first rescoring performance and comparison across a wide range of actigraphy algorithms. This study is among the first to evaluate traditional actigraphy algorithms across a large population of older adults who are also at risk for, or have, health and sleep issues. To our knowledge, this is the first study to provide comprehensive benchmarks and statistical comparisons across an extensive list of the commonly used actigraphy algorithms with the diverse MESA population, while previous studies have focused on novel algorithm development and comparison. In doing so, we provide comprehensive metrics for not only users of these algorithms but also inform future research and practice.

Overall, the ROC-AUC and accuracy results demonstrate that all of the algorithms evaluated in this study are good options for evaluating sleep-wake activity. However, contrary to accuracy, our results for both MCC and Cohen κ demonstrate that traditional algorithms only provide moderate agreement and moderate positive correlations with ground truth polysomnography. These results suggest that though these actigraphy algorithms are a valuable analytic tool, there is a significant area of improvement. With novel technologies as well as improved analytic methods, we can improve our approach to sleep-wake assessment.

Along these lines, our results demonstrate that rescoring of actigraphy algorithms does not substantially improve sleep-wake classification accuracy. Rather, rescoring may significantly diminish the performance (eg, *F*_1_-score) of certain algorithms such as Kripke 2010 and Philips-Respironics. Further, our analyses of sleep problem participants revealed similar findings that rescoring may diminish sleep-wake classification performance. This suggests that researchers and clinicians may safely opt to not rescore and still retain high accuracy.

In addition to the primary results, in-depth ANOVA and post hoc analyses showed significant differences between algorithms despite small differences in accuracy and other metrics. However, examination of effect sizes for MCC, accuracy, and *F*_1_-score demonstrated very small to small effect sizes. Therefore, the significant differences between algorithms could be due to the large sample size of the dataset. The exception was Cohen κ, which demonstrated marginally higher performance for the Kripke 2010 algorithm (small to medium effect sizes). This, along with the Kripke 2010 algorithm demonstrating the highest accuracy, may suggest that this algorithm could be optimal in specific analysis scenarios. However, after rescoring, these effects were reduced with only very small to small effect sizes overall.

Our evaluation of the agreement between actigraphy and polysomnography for sleep metrics TST, SE, and WASO was conducted through Bland-Altman distribution analysis. This analysis demonstrated both over and underestimation of all 3 sleep metrics, underscored by large mean differences between actigraphy and polysomnography. The Bland-Altman distributions also demonstrated considerable systematic bias for SE and WASO. In addition, there were clear patterns that demonstrated agreement decreased as SE decreased or when WASO increased. This was supported by the regression analyses for proportional bias. This suggests that actigraphy may be less precise in accurately measuring sleep metrics in individuals who have irregular sleep or sleep problems. From the traditional algorithms, the Philips algorithm with a threshold of 40 demonstrated the highest agreement with polysomnography for both TST and SE. This may be in part due to the supplied cutoffs for sleep-wake detection, which may allow for the user to adjust the algorithm based on their target population and other factors to improve both sleep-wake scoring and sleep metric estimates [37]. However, an important note is that the MESA data were collected using Philips Actiwatch devices; therefore, it is expected that the Philips algorithms may perform better in these cases than the other algorithms [20,21]. For WASO, the Kripke 2010 algorithm had the highest agreement with polysomnography. This may be due to the larger number of epochs (30) that are assessed by the algorithm to compute a sleep-wake decision. In evaluating more epochs, this algorithm may be more sensitive to shorter waking periods [19]. This may also explain the lower accuracy of this algorithm, as it may be scoring more epochs as wake even when there is true sleep. This suggests a key point for current algorithm use and for future algorithm development, that some variability or ranges of thresholds and longer windows of epoch evaluation may provide better accuracy and improve estimates of sleep metrics.

Another explanation for the bias and heteroscedasticity observed is that the sample evaluated included many individuals who were either at risk of or had health conditions such as the sleep problems highlighted in the sample distributions (Table 1). Though not reported in the NSRR version of the MESA dataset, MESA and publications on this dataset have underscored that this sample includes individuals with hypertension, diabetes, etc [20,48,49]. Therefore, it is expected that these conditions may lead to fragmented and irregular sleep, which may have influenced the actigraph or the algorithm’s ability to accurately measure. Previous studies have noted that individuals with sleep problems or health conditions that may affect sleep demonstrate more variability in actigraphy results of both sleep-wake and sleep metrics [3,12-17]. Therefore, one important consideration was further tests of these algorithms with data from populations with health and sleep issues. Notably, these are the key populations who are recommended or given actigraphs for sleep assessment by researchers and clinicians. Therefore, the precision of their sleep or wake classification and sleep metrics by these algorithms is of great importance.

Along these lines, we first conducted an exploratory analysis of algorithm performance within sleep problem subgroups, specifically examining individuals with insomnia, RLS, apnea, and those who used a CPAP device. We found that traditional actigraphy algorithms do not perform particularly worse; rather, they are just as robust in determining sleep and wake or sleep metrics in individuals with sleep problems compared with the entire sample. These algorithms demonstrated very similar results and patterns to the entire sample with respect to AUC, accuracy, confusion matrix results, MCC, Cohen κ, and agreement (Bland-Altman distributions). However, generally, these algorithms perform well with respect to sleep-wake scoring but not as well for the measurement of specific sleep metrics in contrast to the overall population. Our results show that sleep disorders may skew Bland-Altman distributions as sleep patterns within these disorders deviate from norms. We theorize based on our results that this may result in larger mean differences when compared to ground truth. Based on our results, we theorize that this may create skewness in accuracy in particularly extreme cases. We suggest that clinicians pay attention to severe cases of sleep disorders and take into account our results and the decreased reliability of actigraphy-based sleep metrics such as TST due to outlier sleep patterns.

Overall, these results provide clinicians with nuanced benchmarks for individual algorithm performance on specific sleep disorders. This will allow clinicians to make an informed choice not only based on accuracy, but on sensitivity and specificity metrics to precisely analyze patient sleep data. With mean difference and Bland-Altman plots, clinicians now have benchmarks to provide them with information on how extreme cases may deviate from general trends. Further, both clinicians and manufacturers now have mean difference benchmarks, which reflect the difference between ground truth and actigraphy, allowing for corrections in patient data analyses if needed.

In the future, it would be interesting to compare the precision of traditional actigraphy algorithms across a variety of actigraph and accelerometer devices. Overall, both the main and subgroup results suggest that generally traditional actigraphy algorithms may have poorer agreement with polysomnography in fringe cases. Specifically, cases in which the individual’s sleep may be significantly different from the vast majority of the population. One hypothesis here is that the heteroscedasticity may be correlated to the severity of a sleep problem, which explains the poorer agreement as SE or WASO changes. Further analysis would allow us to gain a better understanding of this phenomenon.

With regard to rescoring and the sleep problem sub-samples, there were mixed results. Traditional use of the Cole-Kripke (1992) [6] algorithm proposed the use of rescoring; first, we investigated rescoring across all included algorithms [40]. Contrary to the belief that rescoring would significantly improve accuracy metrics, rescoring only slightly improved agreement for sleep metrics and may diminish specific performance variables. This could be due to the rescoring of smaller periods of sleep and wake, especially in the aforementioned population with sleep problems. Rescoring may be better suited across multiple nights, which may present more within-participant variability, whereas our sample was only over one night. Rescoring may also smooth out or erroneously change edge cases or cases in which individuals have sleep problems. This would, in part, explain the diminished performance of results. One future direction could be further optimization of rescoring criteria for improvements to sleep-wake detection across algorithms or to better suit individual algorithms specifically. This may also remove the diminished performance observed after rescoring with sleep problem populations. As a current recommendation, we suggest that rescoring be used only when absolutely necessary and that when rescoring, researchers compare results that are both rescored and nonrescored. Our findings suggest that rescoring does not provide any substantial benefits to nonrescored algorithm results. Further, we do not recommend rescoring for sleep disorder population data to preserve accurate sleep-wake scoring.

Overall, these results echo the findings of previous studies which have examined these algorithms. Haghayegh et al [50] examined the performance of the Coke-Kripke, Sadeh, and UCSD algorithms on a sample of 40 healthy adults. Similar to our results, they found accuracies of 85%‐86% across algorithms. Additionally, they also found varying levels of overall bias across algorithms for the algorithms’ estimation of total sleep time, sleep onset latency, and wake after sleep onset. Along these lines, Palotti et al [51] and Jokar et al [52] used a smaller subset of the MESA dataset to compare performance to the Cole-Kripke and Sadeh algorithms to their novel method both for night only and night-day. Cole-Kripke and Sadeh demonstrated mean accuracies ranging from 70%to 85% [53,54].

In addition, this study presented some limitations. Foremost, there has been recent work and novel algorithms for sleep and wake classification. Some notable examples include novel machine learning and other methods to evaluate activity data. These algorithms may be more precise and robust as sleep and wake classification. Though our current study did not directly evaluate these algorithms, previous research has provided some benchmarks for comparison. Palotti et al [51] developed novel Convolutional Neural Network and Long Short-Term Memory machine learning approaches using the MESA dataset for training and testing. Their novel approaches demonstrated better performance (88.2% and 87.7% respectively) than traditional algorithms (Cole-Kripke and Sadeh). In comparison to this study, their novel methods also demonstrate higher performance than traditional algorithms, albeit on a smaller subsample of the MESA dataset. Similarly, Nunes et al [55] developed a domain adversarial convolutional neural network method, trained on the MESA dataset. Their optimal method demonstrated the highest accuracy (80.1%) when compared to other models and traditional algorithms such as Cole-Kripke and Sadeh (71.9% and 69.6%). In contrast to our findings, their novel method could provide stable, more generalizable sleep-wake classification. Again, these results suggest that novel machine learning methods are the future for actigraphy analyses. Given the rapid development of novel actigraphy analytic methods, our future study aim is to examine the performance of newer algorithms and strategies with large testing samples in contrast to our current study findings.

Another limitation of this study is that we only examined sleep problem subgroups as an exploratory aim. Given the wide demographic, there were several other populations within the dataset, such as individuals with diabetes, cardiovascular illness, etc that could explain the lack of agreement or the heteroscedasticity observed. In addition, we did not conduct an analysis examining demographic subgroups such as sex, ethnicity, and age. These groups may have also contributed to the Bland-Altman distributions observed.

Given the similarities of the subgroup results to those of the entire dataset, there may be additional correlation-based analysis, which could help unpack why traditional actigraphy algorithms perform poorly as sleep metrics reach extreme values. As aforementioned, previous research denotes individuals with sleep problems may have lower SE, higher WASO, and a variety of other extreme sleep activity metrics. Therefore, this may be one important limitation of not only the actigraphy algorithms but the activity measurement devices themselves. To address this issue, future research should delve deeper and examine extreme cases and traditional algorithms’ ability to classify sleep and wake and accurately measure sleep metrics. This, in turn, may also allow us to gain a better understanding of specific patterns of sleep and wake in these populations, as well as examine whether specific populations alter the distribution variability observed. In addition, future research should comprehensively assess the performance of traditional and novel algorithms on subsets of the MESA dataset. In doing so, we could identify key markers that may be used to improve analysis performance.

Finally, our study also filtered out fragmented or poor-quality data from the dataset. Even though these data may not be suitable for the current accuracy analysis and comparison, they may highlight important limitations of actigraphy algorithms with respect to the estimation of sleep metrics and the classification of sleep and wake in extreme cases. Therefore, future research should assess a spectrum of poor- and good-quality data to gain a complete understanding of the performance of traditional actigraphy algorithms. In addition, future research should also compare the results of different actigraphy devices to determine, in unison with analytics, the most accurate actigraphy and accelerometer devices.

Overall, the results of this study provide significant support that traditional actigraphy algorithms can, with acceptable accuracy, detect sleep and wake in large, diverse population samples, including older adults or populations at risk of health conditions. This study provides researchers and clinicians with evidence that traditional algorithms can continue to be used to assess sleep-wake activity. However, traditional algorithms may present significant limitations in measurement precision of extreme sleep cases. Further, rescoring may not be a necessary step in the analysis of actigraphy data. The implications of this finding are highly important as researchers develop new algorithms and methods of actigraphy data analysis. Specifically, new algorithms should consider the variability of sleep and wake data in sleep problems or extreme cases to precisely measure sleep and wake activity. Further, the results denoted by the current study serve as an important reference point for new, developing strategies for actigraphy analyses.

### Conclusion

In conclusion, we emphasize the future direction for generalizable, accurate, and comprehensive actigraphy analyses through the application of machine learning and artificial intelligence models. As aforementioned, several researchers have now adapted artificial intelligence–based models and automated toolboxes to analyze actigraphy and reportedly extract more accurate and more detailed sleep-wake results [53-57]. Given the promise of these methods, we theorize that their assessment and usage to analyze actigraphy data in novel ways poses a promising future direction for more accurate and diverse actigraphy analyses for sleep assessment. Further, in unison with demographic analyses, first of their performance, then the usage of demographic and sleep problem data, we propose this would be a novel opportunity to develop more robust and accurate methods of sleep-wake prediction from activity and actigraphy data. In addition, novel methods using unsupervised machine learning may also provide new ways in which sleep-wake actigraphy data could be parsed to reveal underlying patterns of sleep and sleep problems [56,58]. As these methods are fitted to specific datasets, they could provide better generalizability for sleep-wake analyses [56,58].

## Supplementary material

10.2196/70778 Multimedia Appendix 1 Sensitivity analysis of outliers.

10.2196/70778 Multimedia Appendix 2 Formulae for statistical analyses.

10.2196/70778 Multimedia Appendix 3 Confusion metrics, Matthews correlation coefficient, and Cohen κ statistics.

10.2196/70778 Multimedia Appendix 4 ANOVA and post hoc results for Matthews correlation coefficient and Cohen κ.

10.2196/70778 Multimedia Appendix 5 Post hoc results for confusion matrix statistics.

10.2196/70778 Multimedia Appendix 6 Mean difference statistics.

10.2196/70778 Multimedia Appendix 7 Receiver operator characteristic curve and area under the curve statistics for sleep problem subgroups.

10.2196/70778 Multimedia Appendix 8 Subgroup confusion matrix statistics.

10.2196/70778 Multimedia Appendix 9 Subgroup ANOVA and post hoc results for confusion matrix statistics.

10.2196/70778 Multimedia Appendix 10 Subgroup ANOVA and post hoc results for Matthews correlation coefficient and Cohen κ.

10.2196/70778 Multimedia Appendix 11 Subgroup Bland-Altman statistics.

## Abbreviations:

AUC: area under the curve
CK: Cole-Kripke
CPAP: continuous positive airway pressure
ICC: intraclass correlation coefficient
K2010: Kripke 2010
LoA: limits of agreement
MCC: Matthews correlation coefficient
MESA: Multi-Ethnic Study of Atherosclerosis
NRS: nonrescored result
NSRR: National Sleep Research Resource
Philips: Philips-Respironics
RLS: restless leg syndrome
ROC: receiver operator characteristic curve
RR: rescored result
RS: Webster’s rescoring rules
SE: sleep efficiency
TST: total sleep time
UCSD: University of California San Diego
WASO: wake after sleep onset
