# Supplementary material for: Comparison and Validation of Actigraphy Algorithms Using a Large Community Dataset: Algorithm Validation Study
Source: JMIR Form Res. 2025 Dec 11;9:e70778. doi: 10.2196/70778 (PMC12697920; doi:10.2196/70778)
Supplement: Multimedia Appendix 1 [file formative-v9-e70778-s001.docx]

Multimedia Appendix A: Multimedia Appendix for Sensitivity Analysis of Outliers:

**Table S1**

Cole-Kripke (CK) Algorithm

| Metric | Unfiltered Average | Unfiltered SD | Filtered Average | Filtered SD | Absolute Change | Relative Change % |
| --- | --- | --- | --- | --- | --- | --- |
|  |  |  |  |  |  |  |
| sample size | 911777 | nan | 873194 | nan | -38583 | -4.23 |
| accuracy-nrs | 0.80 | 0.09 | 0.80 | 0.09 | -0.0027 | -0.34 |
| sensitivity-nrs | 0.95 | 0.05 | 0.95 | 0.05 | 0.0008 | 0.09 |
| specificity-nrs | 0.56 | 0.19 | 0.55 | 0.18 | -0.0106 | -1.90 |
| recall-nrs | 0.95 | 0.05 | 0.95 | 0.05 | 0.0008 | 0.09 |
| precision-nrs | 0.78 | 0.12 | 0.77 | 0.12 | -0.0016 | -0.20 |
| f1score-nrs | 0.85 | 0.08 | 0.85 | 0.08 | -0.0006 | -0.07 |
| mcc-nrs | 0.57 | nan | 0.56 | nan | -0.0099 | -1.73 |
| kappa-nrs | 0.54 | nan | 0.53 | nan | -0.0113 | -2.08 |
| accuracy-rs | 0.81 | 0.09 | 0.81 | 0.09 | -0.0024 | -0.29 |
| sensitivity-rs | 0.93 | 0.06 | 0.93 | 0.06 | 0.0012 | 0.13 |
| specificity-rs | 0.62 | 0.19 | 0.61 | 0.19 | -0.0094 | -1.50 |
| recall-rs | 0.93 | 0.06 | 0.93 | 0.06 | 0.0012 | 0.13 |
| precision-rs | 0.80 | 0.12 | 0.80 | 0.12 | -0.0017 | -0.21 |
| f1score-rs | 0.85 | 0.08 | 0.85 | 0.08 | -0.0004 | -0.05 |
| mcc-rs | 0.60 | nan | 0.59 | nan | -0.0085 | -1.42 |
| kappa-rs | 0.59 | nan | 0.58 | nan | -0.0095 | -1.62 |

**Table S2**

Kripke 2010 Algorithm

| Metric | Unfiltered Average | Unfiltered SD | Filtered Average | Filtered SD | Absolute Change | Relative Change % |
| --- | --- | --- | --- | --- | --- | --- |
|  |  |  |  |  |  |  |
| sample size | 911777 | nan | 873194 | nan | -38583 | -4.23 |
| accuracy-nrs | 0.80 | 0.09 | 0.80 | 0.09 | -0.002 | -0.25 |
| sensitivity-nrs | 0.84 | 0.10 | 0.84 | 0.10 | 0.0013 | 0.16 |
| specificity-nrs | 0.74 | 0.18 | 0.73 | 0.17 | -0.0068 | -0.92 |
| recall-nrs | 0.84 | 0.10 | 0.84 | 0.10 | 0.0013 | 0.16 |
| precision-nrs | 0.84 | 0.12 | 0.84 | 0.12 | -0.0017 | -0.20 |
| f1score-nrs | 0.83 | 0.09 | 0.83 | 0.09 | -0.0001 | -0.01 |
| mcc-nrs | 0.58 | nan | 0.57 | nan | -0.0071 | -1.23 |
| kappa-nrs | 0.58 | nan | 0.57 | nan | -0.0071 | -1.23 |
| accuracy-rs | 0.79 | 0.09 | 0.79 | 0.09 | -0.0015 | -0.19 |
| sensitivity-rs | 0.78 | 0.13 | 0.79 | 0.13 | 0.0019 | 0.24 |
| specificity-rs | 0.80 | 0.17 | 0.79 | 0.17 | -0.0052 | -0.66 |
| recall-rs | 0.78 | 0.13 | 0.79 | 0.13 | 0.0019 | 0.24 |
| precision-rs | 0.86 | 0.11 | 0.86 | 0.11 | -0.0014 | -0.16 |
| f1score-rs | 0.81 | 0.10 | 0.81 | 0.10 | 0.0006 | 0.07 |
| mcc-rs | 0.57 | nan | 0.57 | nan | -0.0054 | -0.94 |
| kappa-rs | 0.57 | nan | 0.56 | nan | -0.0052 | -0.91 |

**Table S3**

Philips Threshold 20 Algorithm

| Metric | Unfiltered Average | Unfiltered SD | Filtered Average | Filtered SD | Absolute Change | Relative Change % |
| --- | --- | --- | --- | --- | --- | --- |
|  |  |  |  |  |  |  |
| sample size | 911777 | nan | 873194 | nan | -38583 | -4.23 |
| accuracy-nrs | 0.80 | 0.08 | 0.79 | 0.08 | -0.0023 | -0.29 |
| sensitivity-nrs | 0.85 | 0.09 | 0.86 | 0.09 | 0.0009 | 0.11 |
| specificity-nrs | 0.70 | 0.17 | 0.69 | 0.17 | -0.0075 | -1.08 |
| recall-nrs | 0.85 | 0.09 | 0.86 | 0.09 | 0.0009 | 0.11 |
| precision-nrs | 0.82 | 0.12 | 0.82 | 0.12 | -0.0016 | -0.20 |
| f1score-nrs | 0.83 | 0.09 | 0.83 | 0.09 | -0.0003 | -0.04 |
| mcc-nrs | 0.56 | nan | 0.55 | nan | -0.008 | -1.42 |
| kappa-nrs | 0.56 | nan | 0.55 | nan | -0.0082 | -1.46 |
| accuracy-rs | 0.80 | 0.09 | 0.80 | 0.09 | -0.0018 | -0.23 |
| sensitivity-rs | 0.82 | 0.11 | 0.83 | 0.11 | 0.0015 | 0.18 |
| specificity-rs | 0.75 | 0.17 | 0.75 | 0.17 | -0.0063 | -0.83 |
| recall-rs | 0.82 | 0.11 | 0.83 | 0.11 | 0.0015 | 0.18 |
| precision-rs | 0.85 | 0.12 | 0.84 | 0.12 | -0.0015 | -0.18 |
| f1score-rs | 0.83 | 0.09 | 0.83 | 0.09 | 0.0001 | 0.02 |
| mcc-rs | 0.58 | nan | 0.57 | nan | -0.0065 | -1.12 |
| kappa-rs | 0.58 | nan | 0.57 | nan | -0.0065 | -1.12 |

**Table S4**

Philips Threshold 40 Algorithm

| Metric | Unfiltered Average | Unfiltered SD | Filtered Average | Filtered SD | Absolute Change | Relative Change % |
| --- | --- | --- | --- | --- | --- | --- |
|  |  |  |  |  |  |  |
| sample size | 911777 | nan | 873194 | nan | -38583 | -4.23 |
| accuracy-nrs | 0.80 | 0.09 | 0.80 | 0.09 | -0.0024 | -0.30 |
| sensitivity-nrs | 0.90 | 0.07 | 0.90 | 0.07 | 0.0008 | 0.09 |
| specificity-nrs | 0.63 | 0.18 | 0.62 | 0.18 | -0.0089 | -1.41 |
| recall-nrs | 0.90 | 0.07 | 0.90 | 0.07 | 0.0008 | 0.09 |
| precision-nrs | 0.80 | 0.12 | 0.80 | 0.12 | -0.0015 | -0.19 |
| f1score-nrs | 0.84 | 0.08 | 0.84 | 0.08 | -0.0005 | -0.05 |
| mcc-nrs | 0.57 | nan | 0.56 | nan | -0.0089 | -1.58 |
| kappa-nrs | 0.56 | nan | 0.55 | nan | -0.0095 | -1.71 |
| accuracy-rs | 0.80 | 0.09 | 0.81 | 0.09 | 0.0098 | 1.23 |
| sensitivity-rs | 0.84 | 0.10 | 0.88 | 0.08 | 0.0465 | 5.55 |
| specificity-rs | 0.73 | 0.17 | 0.68 | 0.18 | -0.0511 | -7.00 |
| recall-rs | 0.84 | 0.10 | 0.88 | 0.08 | 0.0465 | 5.55 |
| precision-rs | 0.84 | 0.12 | 0.82 | 0.12 | -0.0175 | -2.09 |
| f1score-rs | 0.83 | 0.09 | 0.84 | 0.08 | 0.0144 | 1.73 |
| mcc-rs | 0.59 | nan | 0.58 | nan | -0.0075 | -1.27 |
| kappa-rs | 0.59 | nan | 0.58 | nan | -0.0078 | -1.33 |

**Table S5**

Philips Threshold 80 Algorithm

| Metric | Unfiltered Average | Unfiltered SD | Filtered Average | Filtered SD | Absolute Change | Relative Change % |
| --- | --- | --- | --- | --- | --- | --- |
|  |  |  |  |  |  |  |
| sample size | 911777 | nan | 873194 | nan | -38583 | -4.23 |
| accuracy-nrs | 0.79 | 0.09 | 0.79 | 0.09 | -0.0027 | -0.34 |
| sensitivity-nrs | 0.94 | 0.05 | 0.94 | 0.05 | 0.0007 | 0.07 |
| specificity-nrs | 0.55 | 0.18 | 0.54 | 0.18 | -0.0105 | -1.90 |
| recall-nrs | 0.94 | 0.05 | 0.94 | 0.05 | 0.0007 | 0.07 |
| precision-nrs | 0.77 | 0.12 | 0.77 | 0.12 | -0.0014 | -0.18 |
| f1score-nrs | 0.84 | 0.08 | 0.84 | 0.08 | -0.0006 | -0.07 |
| mcc-nrs | 0.56 | nan | 0.55 | nan | -0.0101 | -1.82 |
| kappa-nrs | 0.53 | nan | 0.52 | nan | -0.0114 | -2.16 |
| accuracy-rs | 0.81 | 0.09 | 0.81 | 0.09 | -0.0025 | -0.31 |
| sensitivity-rs | 0.93 | 0.06 | 0.93 | 0.06 | 0.0009 | 0.10 |
| specificity-rs | 0.61 | 0.19 | 0.60 | 0.18 | -0.0096 | -1.59 |
| recall-rs | 0.93 | 0.06 | 0.93 | 0.06 | 0.0009 | 0.10 |
| precision-rs | 0.79 | 0.12 | 0.79 | 0.12 | -0.0016 | -0.21 |
| f1score-rs | 0.85 | 0.08 | 0.85 | 0.08 | -0.0006 | -0.07 |
| mcc-rs | 0.59 | nan | 0.58 | nan | -0.0091 | -1.55 |
| kappa-rs | 0.57 | nan | 0.56 | nan | -0.0101 | -1.77 |

**Table S6**

Sadeh Algorithm

| Metric | Unfiltered Average | Unfiltered SD | Filtered Average | Filtered SD | Absolute Change | Relative Change % |
| --- | --- | --- | --- | --- | --- | --- |
|  |  |  |  |  |  |  |
| sample size | 911777 | nan | 873194 | nan | -38583 | -4.23 |
| accuracy-nrs | 0.78 | 0.10 | 0.78 | 0.10 | -0.0031 | -0.39 |
| sensitivity-nrs | 0.98 | 0.03 | 0.98 | 0.03 | 0.0006 | 0.06 |
| specificity-nrs | 0.46 | 0.19 | 0.44 | 0.18 | -0.0125 | -2.75 |
| recall-nrs | 0.98 | 0.03 | 0.98 | 0.03 | 0.0006 | 0.06 |
| precision-nrs | 0.74 | 0.12 | 0.74 | 0.12 | -0.0014 | -0.19 |
| f1score-nrs | 0.84 | 0.09 | 0.84 | 0.09 | -0.0007 | -0.08 |
| mcc-nrs | 0.55 | nan | 0.54 | nan | -0.0115 | -2.10 |
| kappa-nrs | 0.49 | nan | 0.47 | nan | -0.0142 | -2.92 |
| accuracy-rs | 0.80 | 0.10 | 0.80 | 0.10 | -0.003 | -0.37 |
| sensitivity-rs | 0.97 | 0.04 | 0.97 | 0.04 | 0.0009 | 0.09 |
| specificity-rs | 0.52 | 0.20 | 0.51 | 0.19 | -0.0118 | -2.27 |
| recall-rs | 0.97 | 0.04 | 0.97 | 0.04 | 0.0009 | 0.09 |
| precision-rs | 0.77 | 0.12 | 0.76 | 0.12 | -0.0018 | -0.23 |
| f1score-rs | 0.85 | 0.09 | 0.85 | 0.09 | -0.0008 | -0.09 |
| mcc-rs | 0.58 | nan | 0.57 | nan | -0.0104 | -1.78 |
| kappa-rs | 0.54 | nan | 0.53 | nan | -0.0128 | -2.38 |

**Table S7**

UCSD Algorithm

| Metric | Unfiltered Average | Unfiltered SD | Filtered Average | Filtered SD | Absolute Change | Relative Change % |
| --- | --- | --- | --- | --- | --- | --- |
|  |  |  |  |  |  |  |
| sample size | 911777 | nan | 873194 | nan | -38583 | -4.23 |
| accuracy-nrs | 0.78 | 0.10 | 0.78 | 0.10 | -0.0029 | -0.38 |
| sensitivity-nrs | 0.97 | 0.03 | 0.97 | 0.03 | 0.0006 | 0.06 |
| specificity-nrs | 0.48 | 0.18 | 0.46 | 0.18 | -0.0119 | -2.50 |
| recall-nrs | 0.97 | 0.03 | 0.97 | 0.03 | 0.0006 | 0.06 |
| precision-nrs | 0.75 | 0.12 | 0.75 | 0.12 | -0.0013 | -0.18 |
| f1score-nrs | 0.84 | 0.09 | 0.84 | 0.09 | -0.0006 | -0.07 |
| mcc-nrs | 0.54 | nan | 0.53 | nan | -0.0112 | -2.07 |
| kappa-nrs | 0.49 | nan | 0.48 | nan | -0.0134 | -2.72 |
| accuracy-rs | 0.80 | 0.09 | 0.80 | 0.09 | -0.0028 | -0.35 |
| sensitivity-rs | 0.96 | 0.04 | 0.96 | 0.04 | 0.0008 | 0.08 |
| specificity-rs | 0.53 | 0.19 | 0.52 | 0.19 | -0.0112 | -2.09 |
| recall-rs | 0.96 | 0.04 | 0.96 | 0.04 | 0.0008 | 0.08 |
| precision-rs | 0.77 | 0.12 | 0.77 | 0.12 | -0.0016 | -0.20 |
| f1score-rs | 0.85 | 0.09 | 0.85 | 0.09 | -0.0007 | -0.08 |
| mcc-rs | 0.58 | nan | 0.57 | nan | -0.0101 | -1.75 |
| kappa-rs | 0.54 | nan | 0.53 | nan | -0.012 | -2.23 |
