# Supplementary material for: Comparison and Validation of Actigraphy Algorithms Using a Large Community Dataset: Algorithm Validation Study
Source: JMIR Form Res. 2025 Dec 11;9:e70778. doi: 10.2196/70778 (PMC12697920; doi:10.2196/70778)
Supplement: Multimedia Appendix 2 [file formative-v9-e70778-s002.docx]

#### Multimedia Appendix B: Formulae for Statistical Analyses

#### Epoch-By-Epoch Comparison.

Equations for confusion matrix analyses:

We used confusion matrix analyses to assess key metrics: accuracy, sensitivity, specificity, precision, and F_1_-score (measure of an algorithm’s predictive performance of sleep and wake).
We defined the confusion matrix metrics as follows:

1. True Positive (tp): PSG epoch and actigraphy epoch are both scored as sleep (both coded as 1)
2. False Positive (fp): PSG epoch is scored as wake and the actigraphy epoch is scored as sleep (coded as 0 and 1 respectively)
3. True Negative (tn): PSG epoch and actigraphy epoch are both scored as wake (both coded as 0)
4. False Negative (fn): PSG epoch is scored as sleep and the actigraphy epoch is scored as wake (coded as 1 and 0 respectively)
5. Accuracy (ACC) = (tp + tn) / (tp + tn + fp + fn) or Total Sample Count
6. Sensitivity/Recall/TPR = tp / (tp + fn)
7. Specificity/TNR = tn / (tn + fp)
8. Precision = tp / (tp + fp)
9. F_1_-score = (2 x (Precision x Recall)) / (Precision + Recall)

Equation for Cohen Kappa analysis was denoted as:

κ = P_o_ – P_e_ / (1-P_e_)

where P_o_ was the observed agreement between raters and P_e_ is the expected probability of change agreement [27,28]. For this study, k agreement ranges were denoted as 0.01-0.02: slight, 0.21-0.40: fair, 0.41– 0.60: moderate, 0.61–0.80: strong, and <0.80: almost perfect [27,28]. With 100% agreement between raters κ = 1.

Using confusion matrix metrics, MCC was denoted as:

MCC = (tp ⋅ tn) – (fp ⋅ fn) / √(tp+fp)(tp+fn)(tn+fp)(tn+fn)

For this study, the correlation ranges were interpreted as based on the result value’s numeric distance from 1 as the indicator of strength of relationship.

Receiver Operating Characteristic Curve (ROC) and Area Under the Curve (AUC) Analysis:

The ROC analyses were conducted in python with code assistance provided by ChatGPT [23]. The following metrics were considered for the ROC plots:

1. Sensitivity = tp / (tp + fn)
2. Specificity = tn / (tn + fp)
3. False Positive Rate (fpr) = fp / (tn + fp)
4. True Positive Rate (tpr) = tp / (tp + fn)
