# Supplementary material for: Comparison and Validation of Actigraphy Algorithms Using a Large Community Dataset: Algorithm Validation Study
Source: JMIR Form Res. 2025 Dec 11;9:e70778. doi: 10.2196/70778 (PMC12697920; doi:10.2196/70778)
Supplement: Multimedia Appendix 3 [file formative-v9-e70778-s003.docx]

Multimedia Appendix C: Confusion Metrics, MCC and Cohens k

Table S1. Cohen κ, MCC, and confusion matrix analysis results for non-rescored algorithms.^a^

| Algorithm | κ | MCC | Accuracy | Sensitivity | Specificity | Precision | F_1_-score |
| --- | --- | --- | --- | --- | --- | --- | --- |
|  |  |  |  |  |  |  |  |
| Cole-Kripke | 0.54 | 0.57 | 0.80(0.09) | 0.95(0.05) | 0.56(0.19) | 0.78(0.12) | 0.85(0.08) |
| Kripke 2010 | 0.58 | 0.58 | 0.80(0.09) | 0.84(0.10) | 0.74(0.18) | 0.84(0.12) | 0.83(0.09) |
| Sadeh | 0.56 | 0.56 | 0.78(0.10) | 0.98(0.03) | 0.46(0.19) | 0.74(0.12) | 0.84(0.09) |
| UCSD | 0.56 | 0.57 | 0.78(0.10) | 0.97(0.03) | 0.48(0.18) | 0.75(0.12) | 0.84(0.09) |
| Philips-Respironics (Threshold = 20) | 0.53 | 0.56 | 0.80(0.09) | 0.85(0.09) | 0.70(0.17) | 0.82(0.12) | 0.83(0.09) |
| Philips-Respironics (Threshold = 40) | 0.49 | 0.55 | 0.80(0.09) | 0.90(0.07) | 0.63(0.18) | 0.80(0.12) | 0.84(0.08) |
| Philips-Respironics (Threshold = 80) | 0.49 | 0.54 | 0.79(0.09) | 0.94(0.05) | 0.55(0.18) | 0.77(0.12) | 0.84(0.08) |

*^a. Accuracy, Sensitivity, Specificity, Precision, F^_1_^-score:^* ^mean (standard deviation).^

Table S2. Cohen κ, MCC, and confusion matrix analysis results for rescored algorithms.^a^

| Algorithm | κ | MCC | Accuracy | Sensitivity | Specificity | Precision | F_1_-score |
| --- | --- | --- | --- | --- | --- | --- | --- |
|  |  |  |  |  |  |  |  |
| Cole-Kripke | 0.59 | 0.60 | 0.81(0.09) | 0.93 (0.06) | 0.62 (0.19) | 0.80 (0.12) | 0.85 (0.08) |
| Kripke 2010 | 0.57 | 0.57 | 0.79(0.09) | 0.78 (0.13) | 0.80 (0.17) | 0.86 (0.11) | 0.81 (0.10) |
| Sadeh | 0.58 | 0.58 | 0.80(0.10) | 0.97 (0.04) | 0.52 (0.20) | 0.77 (0.12) | 0.85 (0.09) |
| UCSD | 0.59 | 0.59 | 0.80(0.09) | 0.96 (0.04) | 0.53 (0.19) | 0.77 (0.12) | 0.85 (0.09) |
| Philips-Respironics (Threshold = 20) | 0.57 | 0.59 | 0.80(0.09) | 0.82 (0.11) | 0.75 (0.17) | 0.85 (0.12) | 0.83 (0.09) |
| Philips-Respironics (Threshold = 40) | 0.58 | 0.54 | 0.81(0.09) | 0.88 (0.08) | 0.69 (0.18) | 0.82 (0.12) | 0.84 (0.08) |
| Philips-Respironics (Threshold = 80) | 0.54 | 0.58 | 0.81(0.09) | 0.93 (0.06) | 0.61 (0.19) | 0.79 (0.12) | 0.85 (0.08) |

*^a. Accuracy, Sensitivity, Specificity, Precision, F^_1_^-score:^* ^mean (standard deviation).^
