# Supplementary material for: Comparison and Validation of Actigraphy Algorithms Using a Large Community Dataset: Algorithm Validation Study
Source: JMIR Form Res. 2025 Dec 11;9:e70778. doi: 10.2196/70778 (PMC12697920; doi:10.2196/70778)
Supplement: Multimedia Appendix 4 [file formative-v9-e70778-s004.docx]

Multimedia Appendix D: Post Hoc Analysis Results for MCC and Cohens Kappa:

**Table 1**

Post hoc analysis for MCC for non-rescored algorithms.^a^

| **A** | **B** | ***T*** | ***df*** | ***P_uncorr._*** | ***P_adjusted_*** | **BF10** | **Hedges *g*** |
| --- | --- | --- | --- | --- | --- | --- | --- |
| K2010 | UCSD | 15.32 | 1438 | p<.001 | p<.001 | 3.82e+45 | 0.24 |
| K2010 | CK | 5.07 | 1438 | p<.001 | p<.001 | 9880.78 | 0.06 |
| K2010 | Philips-20 | 16.47 | 1438 | p<.001 | p<.001 | 2.117e+52 | 0.09 |
| K2010 | Philips-40 | 12.05 | 1438 | p<.001 | p<.001 | 2.756e+28 | 0.09 |
| K2010 | Philips-80 | 13.79 | 1438 | p<.001 | p<.001 | 1.52e+37 | 0.16 |
| K2010 | Sadeh | 11.93 | 1438 | p<.001 | p<.001 | 7.622e+27 | 0.21 |
| UCSD | CK | -33.75 | 1438 | p<.001 | p<.001 | 2.358e+180 | -0.18 |
| UCSD | Philips-20 | -10.51 | 1438 | p<.001 | p<.001 | 3.324e+21 | -0.15 |
| UCSD | Philips-40 | -14.23 | 1438 | p<.001 | p<.001 | 3.479e+39 | -0.15 |
| UCSD | Philips-80 | -13.34 | 1438 | p<.001 | p<.001 | 6.754e+34 | -0.08 |
| UCSD | Sadeh | -7.18 | 1438 | p<.001 | p<.001 | 2.735e+09 | -0.03 |
| CK | Philips-20 | 2.71 | 1438 | .007 | .142 | 1.15 | 0.03 |
| CK | Philips-40 | 4.10 | 1438 | p<.001 | p<.001 | 126.757 | 0.03 |
| CK | Philips-80 | 23.75 | 1438 | p<.001 | p<.001 | 4.543e+101 | 0.10 |
| CK | Sadeh | 19.71 | 1438 | p<.001 | p<.001 | 1.021e+73 | 0.15 |
| Philips-20 | Philips-40 | 0.05 | 1438 | .961 | 1.000 | 0.03 | 0.00 |
| Philips-20 | Philips-80 | 6.94 | 1438 | p<.001 | p<.001 | 5.403e+08 | 0.07 |
| Philips-20 | Sadeh | 7.46 | 1438 | p<.001 | p<.001 | 1.983e+10 | 0.12 |
| Philips-40 | Philips-80 | 11.70 | 1438 | p<.001 | p<.001 | 6.495e+26 | 0.07 |
| Philips-40 | Sadeh | 9.53 | 1438 | p<.001 | p<.001 | 3.634e+17 | 0.12 |
| Philips-80 | Sadeh | 6.34 | 1438 | p<.001 | p<.001 | 1.16e+07 | 0.05 |

*^a. Contrasts between A and B. Bonferroni correction applied. BF10 represents Bayesian factor of 10 results.^*

**Table 2**

Post hoc analysis for MCC for rescored algorithms.^a^

| **A** | **B** | ***T*** | ***df*** | ***P_uncorr._*** | ***P_adjusted_*** | **BF10** | **Hedges *g*** | |
| --- | --- | --- | --- | --- | --- | --- | --- | --- |
| K2010 | UCSD | 1.65 | 1438 | .098 | 1.000 | 0.12 | | 0.03 |
| K2010 | CK | -8.49 | 1438 | p<.001 | p<.001 | 5.102e+13 | | -0.12 |
| K2010 | Philips-20 | -2.79 | 1438 | .005 | .113 | 1.42 | | -0.02 |
| K2010 | Philips-40 | -7.12 | 1438 | p<.001 | p<.001 | 1.813e+09 | | -0.07 |
| K2010 | Philips-80 | -2.67 | 1438 | .008 | .160 | 1.04 | | -0.04 |
| K2010 | Sadeh | -0.30 | 1438 | .762 | 1.000 | 0.03 | | -0.01 |
| UCSD | CK | -21.39 | 1438 | p<.001 | p<.001 | 3.523e+84 | | -0.15 |
| UCSD | Philips-20 | -3.19 | 1438 | .001 | .030 | 4.7 | | -0.05 |
| UCSD | Philips-40 | -8.91 | 1438 | p<.001 | p<.001 | 1.668e+15 | | -0.10 |
| UCSD | Philips-80 | -9.82 | 1438 | p<.001 | p<.001 | 4.804e+18 | | -0.07 |
| UCSD | Sadeh | -8.00 | 1438 | p<.001 | p<.001 | 1.1e+12 | | -0.04 |
| CK | Philips-20 | 8.39 | 1438 | p<.001 | p<.001 | 2.319e+13 | | 0.10 |
| CK | Philips-40 | 6.23 | 1438 | p<.001 | p<.001 | 5.904e+06 | | 0.05 |
| CK | Philips-80 | 16.37 | 1438 | p<.001 | p<.001 | 5.366e+51 | | 0.08 |
| CK | Sadeh | 13.05 | 1438 | p<.001 | p<.001 | 2.235e+33 | | 0.11 |
| Philips-20 | Philips-40 | -8.03 | 1438 | p<.001 | p<.001 | 1.34e+12 | | -0.05 |
| Philips-20 | Philips-80 | -1.59 | 1438 | .111 | 1.000 | 0.1 | | -0.02 |
| Philips-20 | Sadeh | 0.81 | 1438 | .419 | 1.000 | 0.04 | | 0.01 |
| Philips-40 | Philips-80 | 5.04 | 1438 | p<.001 | p<.001 | 8623.36 | | 0.03 |
| Philips-40 | Sadeh | 4.99 | 1438 | p<.001 | p<.001 | 6568.94 | | 0.06 |
| Philips-80 | Sadeh | 3.62 | 1438 | p<.001 | .007 | 19.6 | | 0.03 |

*^a. Contrasts between A and B. Bonferroni correction applied. BF10 represents Bayesian factor of 10 results.^*

**Table 3**

Post hoc analysis for Cohens κ for non-rescored algorithms.^a^

| **A** | **B** | ***T*** | ***df*** | ***P_uncorr._*** | ***P_adjusted_*** | **BF10** | **Hedges *g*** |
| --- | --- | --- | --- | --- | --- | --- | --- |
| K2010 | UCSD | 23.40 | 1438 | p<.001 | p<.001 | 1.028e+99 | 0.42 |
| K2010 | CK | 11.46 | 1438 | p<.001 | p<.001 | 4.985e+25 | 0.16 |
| K2010 | Philips-20 | 14.65 | 1438 | p<.001 | p<.001 | 6.927e+41 | 0.08 |
| K2010 | Philips-40 | 12.47 | 1438 | p<.001 | p<.001 | 2.859e+30 | 0.11 |
| K2010 | Philips-80 | 17.91 | 1438 | p<.001 | p<.001 | 1.737e+61 | 0.24 |
| K2010 | Sadeh | 22.25 | 1438 | p<.001 | p<.001 | 4.571e+90 | 0.44 |
| UCSD | CK | -45.67 | 1438 | p<.001 | p<.001 | 8.206e+277 | -0.26 |
| UCSD | Philips-20 | -21.66 | 1438 | p<.001 | p<.001 | 2.978e+86 | -0.35 |
| UCSD | Philips-40 | -27.88 | 1438 | p<.001 | p<.001 | 1.501e+133 | -0.32 |
| UCSD | Philips-80 | -29.13 | 1438 | p<.001 | p<.001 | 1.175e+143 | -0.18 |
| UCSD | Sadeh | 6.78 | 1438 | p<.001 | p<.001 | 1.858e+08 | 0.02 |
| CK | Philips-20 | -6.78 | 1438 | p<.001 | p<.001 | 1.948e+08 | -0.08 |
| CK | Philips-40 | -7.26 | 1438 | p<.001 | p<.001 | 4.983e+09 | -0.06 |
| CK | Philips-80 | 21.72 | 1438 | p<.001 | p<.001 | 7.648e+86 | 0.08 |
| CK | Sadeh | 35.84 | 1438 | p<.001 | p<.001 | 3.329e+197 | 0.28 |
| Philips-20 | Philips-40 | 4.86 | 1438 | p<.001 | p<.001 | 3554.97 | 0.03 |
| Philips-20 | Philips-80 | 14.64 | 1438 | p<.001 | p<.001 | 6.228e+41 | 0.17 |
| Philips-20 | Sadeh | 20.64 | 1438 | p<.001 | p<.001 | 2.318e+79 | 0.37 |
| Philips-40 | Philips-80 | 21.45 | 1438 | p<.001 | p<.001 | 1.042e+85 | 0.14 |
| Philips-40 | Sadeh | 25.25 | 1438 | p<.001 | p<.001 | 7.978e+112 | 0.34 |
| Philips-80 | Sadeh | 24.25 | 1438 | p<.001 | p<.001 | 2.057e+105 | 0.20 |

*^a. Contrasts between A and B. Bonferroni correction applied. BF10 represents Bayesian factor of 10 results.^*

**Table 4**

Post hoc analysis for Cohens κ for rescored algorithms.^a^

| **A** | **B** | ***T*** | ***df*** | ***P_uncorr._*** | ***P_adjusted_*** | **BF10** | **Hedges *g*** |
| --- | --- | --- | --- | --- | --- | --- | --- |
| K2010 | UCSD | 6.98 | 1438 | p<.001 | p<.001 | 7.138e+08 | 0.15 |
| K2010 | CK | -4.72 | 1438 | p<.001 | p<.001 | 1831.88 | -0.08 |
| K2010 | Philips-20 | -5.65 | 1438 | p<.001 | p<.001 | 2.122e+05 | -0.04 |
| K2010 | Philips-40 | -7.39 | 1438 | p<.001 | p<.001 | 1.251e+10 | -0.09 |
| K2010 | Philips-80 | 0.00 | 1438 | .997 | 1.000 | 0.03 | 0.00 |
| K2010 | Sadeh | 6.56 | 1438 | p<.001 | p<.001 | 4.49e+07 | 0.15 |
| UCSD | CK | -29.92 | 1438 | p<.001 | p<.001 | 2.213e+149 | -0.22 |
| UCSD | Philips-20 | -10.62 | 1438 | p<.001 | p<.001 | 9.81e+21 | -0.19 |
| UCSD | Philips-40 | -18.53 | 1438 | p<.001 | p<.001 | 1.691e+65 | -0.23 |
| UCSD | Philips-80 | -20.88 | 1438 | p<.001 | p<.001 | 1.063e+81 | -0.15 |
| UCSD | Sadeh | 0.46 | 1438 | .644 | 1.000 | 0.03 | 0.00 |
| CK | Philips-20 | 2.79 | 1438 | .005 | .114 | 1.41 | 0.04 |
| CK | Philips-40 | -0.87 | 1438 | .382 | 1.000 | 0.04 | -0.01 |
| CK | Philips-80 | 16.78 | 1438 | p<.001 | p<.001 | 1.7e+54 | 0.08 |
| CK | Sadeh | 24.43 | 1438 | p<.001 | p<.001 | 4.981e+106 | 0.22 |
| Philips-20 | Philips-40 | -6.11 | 1438 | p<.001 | p<.001 | 2.886e+06 | -0.05 |
| Philips-20 | Philips-80 | 3.16 | 1438 | .002 | .033 | 4.32 | 0.04 |
| Philips-20 | Sadeh | 9.80 | 1438 | p<.001 | p<.001 | 4.217e+18 | 0.19 |
| Philips-40 | Philips-80 | 11.46 | 1438 | p<.001 | p<.001 | 4.907e+25 | 0.09 |
| Philips-40 | Sadeh | 16.32 | 1438 | p<.001 | p<.001 | 2.688e+51 | 0.23 |
| Philips-80 | Sadeh | 16.62 | 1438 | p<.001 | p<.001 | 1.741e+53 | 0.15 |

*^a. Contrasts between A and B. Bonferroni correction applied. BF10 represents Bayesian factor of 10 results.^*
