# Supplementary material for: Comparison and Validation of Actigraphy Algorithms Using a Large Community Dataset: Algorithm Validation Study
Source: JMIR Form Res. 2025 Dec 11;9:e70778. doi: 10.2196/70778 (PMC12697920; doi:10.2196/70778)
Supplement: Multimedia Appendix 5 [file formative-v9-e70778-s005.docx]

Multimedia Appendix E: Post Hoc Analysis Results for Confusion Matrix Metrics:

**Table S1**

Post hoc analysis for confusion matrix metrics for non-rescored algorithms

| Metric | A | B | *T* | *df* | *P_uncorr._* | *P_adjusted_* | BF10 | Hedges *g* |
| --- | --- | --- | --- | --- | --- | --- | --- | --- |
| Accuracy | K2010 | UCSD | 8.38 | 1438 | p<.001 | p<.001 | 2.068e+13 | 0.18 |
| Accuracy | K2010 | CK | -0.05 | 1438 | 0.964 | 1.000 | 0.03 | -0.001 |
| Accuracy | K2010 | Philips-20 | 7.19 | 1438 | p<.001 | p<.001 | 2.99e+09 | 0.05 |
| Accuracy | K2010 | Philips-40 | 0.60 | 1438 | 0.547 | 1.000 | 0.04 | 0.01 |
| Accuracy | K2010 | Philips-80 | 4.31 | 1438 | p<.001 | p<.001 | 290.9 | 0.07 |
| Accuracy | K2010 | Sadeh | 7.83 | 1438 | p<.001 | p<.001 | 3.055e+11 | 0.18 |
| Accuracy | UCSD | CK | -30.50 | 1438 | p<.001 | p<.001 | 1.008e+154 | -0.18 |
| Accuracy | UCSD | Philips-20 | -7.51 | 1438 | p<.001 | p<.001 | 2.944e+10 | -0.14 |
| Accuracy | UCSD | Philips-40 | -13.88 | 1438 | p<.001 | p<.001 | 4.549e+37 | -0.18 |
| Accuracy | UCSD | Philips-80 | -17.38 | 1438 | p<.001 | p<.001 | 7.983e+57 | -0.11 |
| Accuracy | UCSD | Sadeh | 1.26 | 1438 | 0.209 | 1.000 | 0.06 | 0.004 |
| Accuracy | CK | Philips-20 | 3.05 | 1438 | 0.002 | 0.049 | 3.04 | 0.04 |
| Accuracy | CK | Philips-40 | 0.87 | 1438 | 0.386 | 1.000 | 0.04 | 0.01 |
| Accuracy | CK | Philips-80 | 22.64 | 1438 | p<.001 | p<.001 | 3.109e+93 | 0.07 |
| Accuracy | CK | Sadeh | 23.08 | 1438 | p<.001 | p<.001 | 5.22e+96 | 0.18 |
| Accuracy | Philips-20 | Philips-40 | -5.32 | 1438 | p<.001 | p<.001 | 3.548e+04 | -0.04 |
| Accuracy | Philips-20 | Philips-80 | 2.15 | 1438 | 0.031 | 0.658 | 0.3 | 0.03 |
| Accuracy | Philips-20 | Sadeh | 7.00 | 1438 | p<.001 | p<.001 | 7.957e+08 | 0.14 |
| Accuracy | Philips-40 | Philips-80 | 9.18 | 1438 | p<.001 | p<.001 | 1.63e+16 | 0.07 |
| Accuracy | Philips-40 | Sadeh | 12.23 | 1438 | p<.001 | p<.001 | 2.019e+29 | 0.18 |
| Accuracy | Philips-80 | Sadeh | 13.58 | 1438 | p<.001 | p<.001 | 1.146e+36 | 0.11 |
| Sensitivity | K2010 | UCSD | -63.42 | 1436 | p<.001 | p<.001 | inf | -1.74 |
| Sensitivity | K2010 | CK | -65.89 | 1436 | p<.001 | p<.001 | inf | -1.38 |
| Sensitivity | K2010 | Philips-20 | -26.34 | 1436 | p<.001 | p<.001 | 1.484e+121 | -0.19 |
| Sensitivity | K2010 | Philips-40 | -62.62 | 1436 | p<.001 | p<.001 | inf | -0.74 |
| Sensitivity | K2010 | Philips-80 | -65.57 | 1436 | p<.001 | p<.001 | inf | -1.30 |
| Sensitivity | K2010 | Sadeh | -63.20 | 1436 | p<.001 | p<.001 | inf | -1.92 |
| Sensitivity | UCSD | CK | 43.64 | 1436 | p<.001 | p<.001 | 2.205e+261 | 0.52 |
| Sensitivity | UCSD | Philips-20 | 61.88 | 1436 | p<.001 | p<.001 | inf | 1.67 |
| Sensitivity | UCSD | Philips-40 | 55.76 | 1436 | p<.001 | p<.001 | inf | 1.26 |
| Sensitivity | UCSD | Philips-80 | 47.99 | 1436 | p<.001 | p<.001 | 2.827e+296 | 0.70 |
| Sensitivity | UCSD | Sadeh | -40.99 | 1436 | p<.001 | p<.001 | 5.182e+239 | -0.37 |
| Sensitivity | CK | Philips-20 | 61.79 | 1436 | p<.001 | p<.001 | inf | 1.27 |
| Sensitivity | CK | Philips-40 | 52.57 | 1436 | p<.001 | p<.001 | inf | 0.78 |
| Sensitivity | CK | Philips-80 | 18.87 | 1436 | p<.001 | p<.001 | 2.649e+67 | 0.14 |
| Sensitivity | CK | Sadeh | -47.05 | 1436 | p<.001 | p<.001 | 8.413e+288 | -0.84 |
| Sensitivity | Philips-20 | Philips-40 | -64.76 | 1436 | p<.001 | p<.001 | inf | -0.57 |
| Sensitivity | Philips-20 | Philips-80 | -64.18 | 1436 | p<.001 | p<.001 | inf | -1.18 |
| Sensitivity | Philips-20 | Sadeh | -62.57 | 1436 | p<.001 | p<.001 | inf | -1.87 |
| Sensitivity | Philips-40 | Philips-80 | -56.31 | 1436 | p<.001 | p<.001 | inf | -0.66 |
| Sensitivity | Philips-40 | Sadeh | -57.18 | 1436 | p<.001 | p<.001 | inf | -1.51 |
| Sensitivity | Philips-80 | Sadeh | -52.07 | 1436 | p<.001 | p<.001 | inf | -1.02 |
| Specificity | K2010 | UCSD | 100.50 | 1438 | p<.001 | p<.001 | inf | 1.46 |
| Specificity | K2010 | CK | 90.89 | 1438 | p<.001 | p<.001 | inf | 1.00 |
| Specificity | K2010 | Philips-20 | 51.56 | 1438 | p<.001 | p<.001 | inf | 0.24 |
| Specificity | K2010 | Philips-40 | 89.08 | 1438 | p<.001 | p<.001 | inf | 0.60 |
| Specificity | K2010 | Philips-80 | 98.33 | 1438 | p<.001 | p<.001 | inf | 1.05 |
| Specificity | K2010 | Sadeh | 96.76 | 1438 | p<.001 | p<.001 | inf | 1.55 |
| Specificity | UCSD | CK | -83.54 | 1438 | p<.001 | p<.001 | inf | -0.43 |
| Specificity | UCSD | Philips-20 | -95.00 | 1438 | p<.001 | p<.001 | inf | -1.23 |
| Specificity | UCSD | Philips-40 | -87.95 | 1438 | p<.001 | p<.001 | inf | -0.85 |
| Specificity | UCSD | Philips-80 | -74.41 | 1438 | p<.001 | p<.001 | inf | -0.41 |
| Specificity | UCSD | Sadeh | 34.52 | 1438 | p<.001 | p<.001 | 4.364e+186 | 0.11 |
| Specificity | CK | Philips-20 | -79.73 | 1438 | p<.001 | p<.001 | inf | -0.77 |
| Specificity | CK | Philips-40 | -64.25 | 1438 | p<.001 | p<.001 | inf | -0.40 |
| Specificity | CK | Philips-80 | 10.88 | 1438 | p<.001 | p<.001 | 1.339e+23 | 0.03 |
| Specificity | CK | Sadeh | 78.13 | 1438 | p<.001 | p<.001 | inf | 0.54 |
| Specificity | Philips-20 | Philips-40 | 82.35 | 1438 | p<.001 | p<.001 | inf | 0.37 |
| Specificity | Philips-20 | Philips-80 | 92.25 | 1438 | p<.001 | p<.001 | inf | 0.82 |
| Specificity | Philips-20 | Sadeh | 92.07 | 1438 | p<.001 | p<.001 | inf | 1.33 |
| Specificity | Philips-40 | Philips-80 | 83.34 | 1438 | p<.001 | p<.001 | inf | 0.44 |
| Specificity | Philips-40 | Sadeh | 84.60 | 1438 | p<.001 | p<.001 | inf | 0.95 |
| Specificity | Philips-80 | Sadeh | 71.80 | 1438 | p<.001 | p<.001 | inf | 0.51 |
| Precision | K2010 | UCSD | 62.20 | 1438 | p<.001 | p<.001 | inf | 0.75 |
| Precision | K2010 | CK | 57.54 | 1438 | p<.001 | p<.001 | inf | 0.53 |
| Precision | K2010 | Philips-20 | 40.38 | 1438 | p<.001 | p<.001 | 6.206e+234 | 0.16 |
| Precision | K2010 | Philips-40 | 56.17 | 1438 | p<.001 | p<.001 | inf | 0.35 |
| Precision | K2010 | Philips-80 | 60.56 | 1438 | p<.001 | p<.001 | inf | 0.57 |
| Precision | K2010 | Sadeh | 61.73 | 1438 | p<.001 | p<.001 | inf | 0.79 |
| Precision | UCSD | CK | -57.62 | 1438 | p<.001 | p<.001 | inf | -0.22 |
| Precision | UCSD | Philips-20 | -60.11 | 1438 | p<.001 | p<.001 | inf | -0.59 |
| Precision | UCSD | Philips-40 | -58.25 | 1438 | p<.001 | p<.001 | inf | -0.40 |
| Precision | UCSD | Philips-80 | -51.96 | 1438 | p<.001 | p<.001 | inf | -0.18 |
| Precision | UCSD | Sadeh | 23.18 | 1438 | p<.001 | p<.001 | 2.504e+97 | 0.04 |
| Precision | CK | Philips-20 | -52.46 | 1438 | p<.001 | p<.001 | inf | -0.37 |
| Precision | CK | Philips-40 | -44.69 | 1438 | p<.001 | p<.001 | 1.024e+270 | -0.18 |
| Precision | CK | Philips-80 | 17.73 | 1438 | p<.001 | p<.001 | 1.156e+60 | 0.03 |
| Precision | CK | Sadeh | 56.88 | 1438 | p<.001 | p<.001 | inf | 0.25 |
| Precision | Philips-20 | Philips-40 | 52.73 | 1438 | p<.001 | p<.001 | inf | 0.19 |
| Precision | Philips-20 | Philips-80 | 58.47 | 1438 | p<.001 | p<.001 | inf | 0.41 |
| Precision | Philips-20 | Sadeh | 59.86 | 1438 | p<.001 | p<.001 | inf | 0.63 |
| Precision | Philips-40 | Philips-80 | 56.27 | 1438 | p<.001 | p<.001 | inf | 0.22 |
| Precision | Philips-40 | Sadeh | 57.72 | 1438 | p<.001 | p<.001 | inf | 0.44 |
| Precision | Philips-80 | Sadeh | 51.62 | 1438 | p<.001 | p<.001 | inf | 0.22 |
| F_1_-score | K2010 | UCSD | -6.50 | 1438 | p<.001 | p<.001 | 3.125e+07 | -0.12 |
| F_1_-score | K2010 | CK | -13.85 | 1438 | p<.001 | p<.001 | 3.268e+37 | -0.20 |
| F_1_-score | K2010 | Philips-20 | -1.34 | 1438 | 0.180 | 1.000 | 0.07 | -0.01 |
| F_1_-score | K2010 | Philips-40 | -12.74 | 1438 | p<.001 | p<.001 | 6.508e+31 | -0.12 |
| F_1_-score | K2010 | Philips-80 | -10.14 | 1438 | p<.001 | p<.001 | 9.506e+19 | -0.15 |
| F_1_-score | K2010 | Sadeh | -6.88 | 1438 | p<.001 | p<.001 | 3.747e+08 | -0.13 |
| F_1_-score | UCSD | CK | -19.20 | 1438 | p<.001 | p<.001 | 3.839e+69 | -0.09 |
| F_1_-score | UCSD | Philips-20 | 7.24 | 1438 | p<.001 | p<.001 | 4.266e+09 | 0.11 |
| F_1_-score | UCSD | Philips-40 | -0.33 | 1438 | 0.744 | 1.000 | 0.03 | -0.003 |
| F_1_-score | UCSD | Philips-80 | -5.72 | 1438 | p<.001 | p<.001 | 2.969e+05 | -0.03 |
| F_1_-score | UCSD | Sadeh | -7.30 | 1438 | p<.001 | p<.001 | 6.583e+09 | -0.02 |
| F_1_-score | CK | Philips-20 | 16.26 | 1438 | p<.001 | p<.001 | 1.159e+51 | 0.20 |
| F_1_-score | CK | Philips-40 | 12.57 | 1438 | p<.001 | p<.001 | 9.393e+30 | 0.09 |
| F_1_-score | CK | Philips-80 | 23.15 | 1438 | p<.001 | p<.001 | 1.525e+97 | 0.06 |
| F_1_-score | CK | Sadeh | 11.89 | 1438 | p<.001 | p<.001 | 4.938e+27 | 0.07 |
| F_1_-score | Philips-20 | Philips-40 | -18.32 | 1438 | p<.001 | p<.001 | 7.018e+63 | -0.12 |
| F_1_-score | Philips-20 | Philips-80 | -12.15 | 1438 | p<.001 | p<.001 | 8.263e+28 | -0.14 |
| F_1_-score | Philips-20 | Sadeh | -7.68 | 1438 | p<.001 | p<.001 | 9.838e+10 | -0.13 |
| F_1_-score | Philips-40 | Philips-80 | -4.18 | 1438 | p<.001 | p<.001 | 171.42 | -0.02 |
| F_1_-score | Philips-40 | Sadeh | -1.16 | 1438 | 0.245 | 1.000 | 0.06 | -0.01 |
| F_1_-score | Philips-80 | Sadeh | 1.78 | 1438 | 0.075 | 1.000 | 0.14 | 0.01 |

*^a. Contrasts between A and B. Bonferroni correction applied. BF10 represents Bayesian factor of 10 results.^*

**Table S2**

Post hoc analysis for confusion matrix metrics for rescored algorithms

| Metric | A | B | *T* | *df* | *P_uncorr._* | *P_adjusted_* | BF10 | Hedges *g* |
| --- | --- | --- | --- | --- | --- | --- | --- | --- |
| Accuracy | K2010 | UCSD | -4.06 | 1438 | p<.001 | 0.001 | 104.03 | -0.10 |
| Accuracy | K2010 | CK | -12.92 | 1438 | p<.001 | p<.001 | 5.236e+32 | -0.26 |
| Accuracy | K2010 | Philips-20 | -11.98 | 1438 | p<.001 | p<.001 | 1.327e+28 | -0.10 |
| Accuracy | K2010 | Philips-40 | -15.17 | 1438 | p<.001 | p<.001 | 5.51e+44 | -0.22 |
| Accuracy | K2010 | Philips-80 | -9.43 | 1438 | p<.001 | p<.001 | 1.502e+17 | -0.20 |
| Accuracy | K2010 | Sadeh | -4.27 | 1438 | p<.001 | p<.001 | 253.18 | -0.11 |
| Accuracy | UCSD | CK | -19.59 | 1438 | p<.001 | p<.001 | 1.663e+72 | -0.16 |
| Accuracy | UCSD | Philips-20 | 0.31 | 1438 | 0.755 | 1.000 | 0.03 | 0.01 |
| Accuracy | UCSD | Philips-40 | -7.63 | 1438 | p<.001 | p<.001 | 7.095e+10 | -0.11 |
| Accuracy | UCSD | Philips-80 | -12.41 | 1438 | p<.001 | p<.001 | 1.558e+30 | -0.09 |
| Accuracy | UCSD | Sadeh | -3.42 | 1438 | p<.001 | 0.014 | 9.87 | -0.01 |
| Accuracy | CK | Philips-20 | 10.38 | 1438 | p<.001 | p<.001 | 1.008e+21 | 0.17 |
| Accuracy | CK | Philips-40 | 5.62 | 1438 | p<.001 | p<.001 | 1.752e+05 | 0.05 |
| Accuracy | CK | Philips-80 | 15.61 | 1438 | p<.001 | p<.001 | 1.889e+47 | 0.07 |
| Accuracy | CK | Sadeh | 14.95 | 1438 | p<.001 | p<.001 | 3.047e+43 | 0.14 |
| Accuracy | Philips-20 | Philips-40 | -13.34 | 1438 | p<.001 | p<.001 | 6.637e+34 | -0.12 |
| Accuracy | Philips-20 | Philips-80 | -6.13 | 1438 | p<.001 | p<.001 | 3.343e+06 | -0.10 |
| Accuracy | Philips-20 | Sadeh | -0.84 | 1438 | 0.400 | 1.000 | 0.04 | -0.02 |
| Accuracy | Philips-40 | Philips-80 | 2.24 | 1438 | 0.025 | 0.533 | 0.36 | 0.02 |
| Accuracy | Philips-40 | Sadeh | 6.02 | 1438 | p<.001 | p<.001 | 1.702e+06 | 0.10 |
| Accuracy | Philips-80 | Sadeh | 8.59 | 1438 | p<.001 | p<.001 | 1.162e+14 | 0.08 |
| Sensitivity | K2010 | UCSD | -66.19 | 1436 | p<.001 | p<.001 | inf | -1.87 |
| Sensitivity | K2010 | CK | -68.79 | 1436 | p<.001 | p<.001 | inf | -1.45 |
| Sensitivity | K2010 | Philips-20 | -40.94 | 1436 | p<.001 | p<.001 | 2.154e+239 | -0.34 |
| Sensitivity | K2010 | Philips-40 | -66.02 | 1436 | p<.001 | p<.001 | inf | -0.91 |
| Sensitivity | K2010 | Philips-80 | -68.23 | 1436 | p<.001 | p<.001 | inf | -1.47 |
| Sensitivity | K2010 | Sadeh | -65.93 | 1436 | p<.001 | p<.001 | inf | -1.99 |
| Sensitivity | UCSD | CK | 43.40 | 1436 | p<.001 | p<.001 | 2.714e+259 | 0.60 |
| Sensitivity | UCSD | Philips-20 | 61.49 | 1436 | p<.001 | p<.001 | inf | 1.66 |
| Sensitivity | UCSD | Philips-40 | 54.53 | 1436 | p<.001 | p<.001 | inf | 1.22 |
| Sensitivity | UCSD | Philips-80 | 45.32 | 1436 | p<.001 | p<.001 | 8.785e+274 | 0.63 |
| Sensitivity | UCSD | Sadeh | -32.10 | 1436 | p<.001 | p<.001 | 7.581e+166 | -0.26 |
| Sensitivity | CK | Philips-20 | 60.92 | 1436 | p<.001 | p<.001 | inf | 1.19 |
| Sensitivity | CK | Philips-40 | 48.74 | 1436 | p<.001 | p<.001 | 2.618e+302 | 0.65 |
| Sensitivity | CK | Philips-80 | 0.05 | 1436 | 0.961 | 1.000 | 0.03 | 0.00 |
| Sensitivity | CK | Sadeh | -46.77 | 1436 | p<.001 | p<.001 | 4.913e+286 | -0.80 |
| Sensitivity | Philips-20 | Philips-40 | -61.84 | 1436 | p<.001 | p<.001 | inf | -0.60 |
| Sensitivity | Philips-20 | Philips-80 | -63.68 | 1436 | p<.001 | p<.001 | inf | -1.21 |
| Sensitivity | Philips-20 | Sadeh | -61.99 | 1436 | p<.001 | p<.001 | inf | -1.80 |
| Sensitivity | Philips-40 | Philips-80 | -54.54 | 1436 | p<.001 | p<.001 | inf | -0.67 |
| Sensitivity | Philips-40 | Sadeh | -55.73 | 1436 | p<.001 | p<.001 | inf | -1.39 |
| Sensitivity | Philips-80 | Sadeh | -49.04 | 1436 | p<.001 | p<.001 | 7.321e+304 | -0.85 |
| Specificity | K2010 | UCSD | 88.91 | 1438 | p<.001 | p<.001 | inf | 1.46 |
| Specificity | K2010 | CK | 76.01 | 1438 | p<.001 | p<.001 | inf | 0.97 |
| Specificity | K2010 | Philips-20 | 41.12 | 1438 | p<.001 | p<.001 | 7.863e+240 | 0.25 |
| Specificity | K2010 | Philips-40 | 69.72 | 1438 | p<.001 | p<.001 | inf | 0.62 |
| Specificity | K2010 | Philips-80 | 82.51 | 1438 | p<.001 | p<.001 | inf | 1.07 |
| Specificity | K2010 | Sadeh | 85.67 | 1438 | p<.001 | p<.001 | inf | 1.51 |
| Specificity | UCSD | CK | -68.20 | 1438 | p<.001 | p<.001 | inf | -0.46 |
| Specificity | UCSD | Philips-20 | -84.32 | 1438 | p<.001 | p<.001 | inf | -1.20 |
| Specificity | UCSD | Philips-40 | -76.37 | 1438 | p<.001 | p<.001 | inf | -0.82 |
| Specificity | UCSD | Philips-80 | -60.15 | 1438 | p<.001 | p<.001 | inf | -0.38 |
| Specificity | UCSD | Sadeh | 18.80 | 1438 | p<.001 | p<.001 | 1.001e+67 | 0.07 |
| Specificity | CK | Philips-20 | -67.50 | 1438 | p<.001 | p<.001 | inf | -0.72 |
| Specificity | CK | Philips-40 | -50.18 | 1438 | p<.001 | p<.001 | inf | -0.35 |
| Specificity | CK | Philips-80 | 19.24 | 1438 | p<.001 | p<.001 | 7.307e+69 | 0.08 |
| Specificity | CK | Sadeh | 65.65 | 1438 | p<.001 | p<.001 | inf | 0.53 |
| Specificity | Philips-20 | Philips-40 | 63.69 | 1438 | p<.001 | p<.001 | inf | 0.37 |
| Specificity | Philips-20 | Philips-80 | 78.38 | 1438 | p<.001 | p<.001 | inf | 0.81 |
| Specificity | Philips-20 | Sadeh | 81.25 | 1438 | p<.001 | p<.001 | inf | 1.26 |
| Specificity | Philips-40 | Philips-80 | 66.67 | 1438 | p<.001 | p<.001 | inf | 0.44 |
| Specificity | Philips-40 | Sadeh | 73.18 | 1438 | p<.001 | p<.001 | inf | 0.88 |
| Specificity | Philips-80 | Sadeh | 58.99 | 1438 | p<.001 | p<.001 | inf | 0.45 |
| Precision | K2010 | UCSD | 56.69 | 1438 | p<.001 | p<.001 | inf | 0.80 |
| Precision | K2010 | CK | 49.75 | 1438 | p<.001 | p<.001 | inf | 0.54 |
| Precision | K2010 | Philips-20 | 30.75 | 1438 | p<.001 | p<.001 | 1.096e+156 | 0.16 |
| Precision | K2010 | Philips-40 | 46.08 | 1438 | p<.001 | p<.001 | 1.724e+281 | 0.37 |
| Precision | K2010 | Philips-80 | 53.13 | 1438 | p<.001 | p<.001 | inf | 0.61 |
| Precision | K2010 | Sadeh | 56.13 | 1438 | p<.001 | p<.001 | inf | 0.82 |
| Precision | UCSD | CK | -49.89 | 1438 | p<.001 | p<.001 | inf | -0.25 |
| Precision | UCSD | Philips-20 | -55.19 | 1438 | p<.001 | p<.001 | inf | -0.64 |
| Precision | UCSD | Philips-40 | -53.01 | 1438 | p<.001 | p<.001 | inf | -0.43 |
| Precision | UCSD | Philips-80 | -44.17 | 1438 | p<.001 | p<.001 | 6.64e+265 | -0.19 |
| Precision | UCSD | Sadeh | 10.80 | 1438 | p<.001 | p<.001 | 6.006e+22 | 0.02 |
| Precision | CK | Philips-20 | -45.44 | 1438 | p<.001 | p<.001 | 1.303e+276 | -0.39 |
| Precision | CK | Philips-40 | -35.94 | 1438 | p<.001 | p<.001 | 2.11e+198 | -0.18 |
| Precision | CK | Philips-80 | 20.69 | 1438 | p<.001 | p<.001 | 5.159e+79 | 0.06 |
| Precision | CK | Sadeh | 49.66 | 1438 | p<.001 | p<.001 | inf | 0.28 |
| Precision | Philips-20 | Philips-40 | 42.08 | 1438 | p<.001 | p<.001 | 4.967e+248 | 0.21 |
| Precision | Philips-20 | Philips-80 | 51.55 | 1438 | p<.001 | p<.001 | inf | 0.45 |
| Precision | Philips-20 | Sadeh | 54.73 | 1438 | p<.001 | p<.001 | inf | 0.66 |
| Precision | Philips-40 | Philips-80 | 47.87 | 1438 | p<.001 | p<.001 | 4.226e+295 | 0.24 |
| Precision | Philips-40 | Sadeh | 52.12 | 1438 | p<.001 | p<.001 | inf | 0.46 |
| Precision | Philips-80 | Sadeh | 44.09 | 1438 | p<.001 | p<.001 | 1.409e+265 | 0.22 |
| F_1_-score | K2010 | UCSD | -18.04 | 1438 | p<.001 | p<.001 | 1.083e+62 | -0.40 |
| F_1_-score | K2010 | CK | -24.97 | 1438 | p<.001 | p<.001 | 5.589e+110 | -0.46 |
| F_1_-score | K2010 | Philips-20 | -19.50 | 1438 | p<.001 | p<.001 | 3.846e+71 | -0.15 |
| F_1_-score | K2010 | Philips-40 | -25.89 | 1438 | p<.001 | p<.001 | 5.44e+117 | -0.35 |
| F_1_-score | K2010 | Philips-80 | -22.19 | 1438 | p<.001 | p<.001 | 1.974e+90 | -0.42 |
| F_1_-score | K2010 | Sadeh | -18.22 | 1438 | p<.001 | p<.001 | 1.557e+63 | -0.42 |
| F_1_-score | UCSD | CK | -8.99 | 1438 | p<.001 | p<.001 | 3.231e+15 | -0.06 |
| F_1_-score | UCSD | Philips-20 | 13.93 | 1438 | p<.001 | p<.001 | 8.53e+37 | 0.26 |
| F_1_-score | UCSD | Philips-40 | 5.08 | 1438 | p<.001 | p<.001 | 1.032e+04 | 0.06 |
| F_1_-score | UCSD | Philips-80 | -2.03 | 1438 | 0.043 | 0.900 | 0.23 | -0.01 |
| F_1_-score | UCSD | Sadeh | -8.86 | 1438 | p<.001 | p<.001 | 1.064e+15 | -0.02 |
| F_1_-score | CK | Philips-20 | 21.85 | 1438 | p<.001 | p<.001 | 6.814e+87 | 0.31 |
| F_1_-score | CK | Philips-40 | 15.69 | 1438 | p<.001 | p<.001 | 4.976e+47 | 0.12 |
| F_1_-score | CK | Philips-80 | 12.81 | 1438 | p<.001 | p<.001 | 1.354e+32 | 0.05 |
| F_1_-score | CK | Sadeh | 4.41 | 1438 | p<.001 | p<.001 | 461.60 | 0.03 |
| F_1_-score | Philips-20 | Philips-40 | -23.74 | 1438 | p<.001 | p<.001 | 3.659e+101 | -0.20 |
| F_1_-score | Philips-20 | Philips-80 | -18.76 | 1438 | p<.001 | p<.001 | 4.841e+66 | -0.27 |
| F_1_-score | Philips-20 | Sadeh | -14.42 | 1438 | p<.001 | p<.001 | 3.893e+40 | -0.28 |
| F_1_-score | Philips-40 | Philips-80 | -9.84 | 1438 | p<.001 | p<.001 | 5.947e+18 | -0.07 |
| F_1_-score | Philips-40 | Sadeh | -6.52 | 1438 | p<.001 | p<.001 | 3.53e+07 | -0.08 |
| F_1_-score | Philips-80 | Sadeh | -1.87 | 1438 | 0.062 | 1.000 | 0.17 | -0.01 |

*^a. Contrasts between A and B. Bonferroni correction applied. BF10 represents Bayesian factor of 10 results.^*
