# Supplementary material for: Comparison and Validation of Actigraphy Algorithms Using a Large Community Dataset: Algorithm Validation Study
Source: JMIR Form Res. 2025 Dec 11;9:e70778. doi: 10.2196/70778 (PMC12697920; doi:10.2196/70778)
Supplement: Multimedia Appendix 6 [file formative-v9-e70778-s006.docx]

Multimedia Appendix F: Mean Difference for Sleep Metrics:

**Table S1**

Mean difference results for total sleep time (TST) ^a^

*
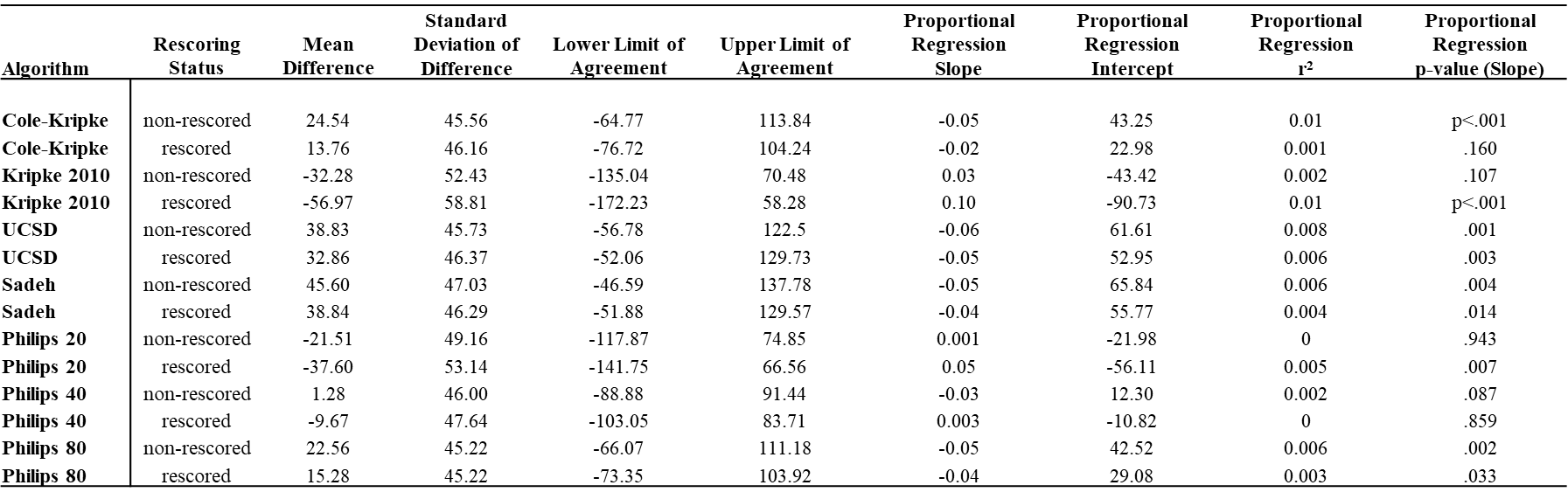
*

^a. Actigraphy and PSG metrics for Total Sleep Time (TST) including; mean difference, standard deviation of difference, lower and upper limits of agreement (95%), and proportional bias regression results for each respect algorithm both non rescored and rescored.^

**Table S2**

Mean difference results for sleep efficiency (SE) ^a^


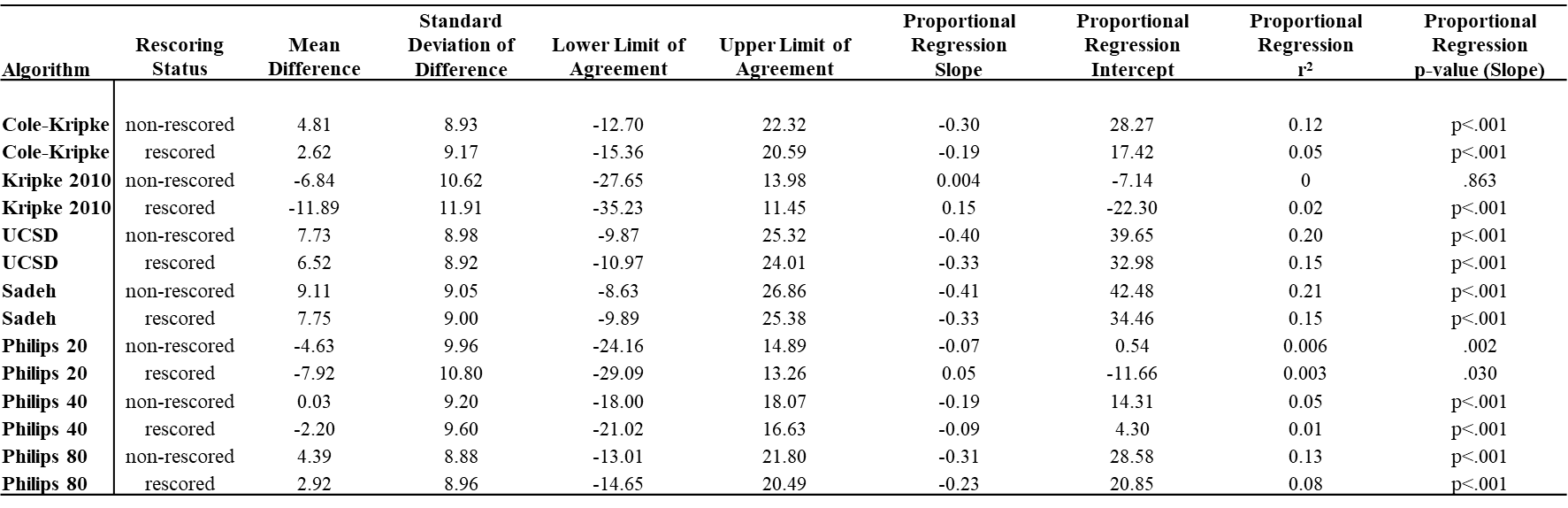


^a. Actigraphy and PSG metrics for Sleep Efficiency (SE) including; mean difference, standard deviation of difference, lower and upper limits of agreement (95%), and proportional bias regression results for each respect algorithm both non rescored and rescored.^

**Table S3**

Mean difference results for wake after sleep onset (WASO) ^a^


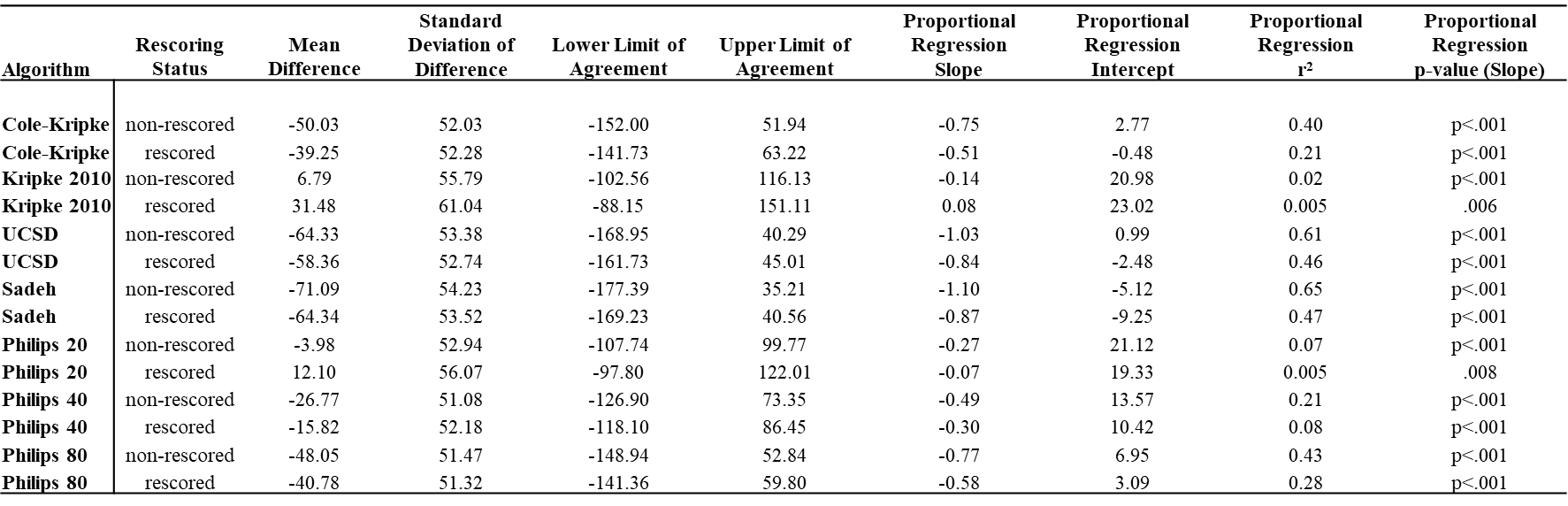


^a. Actigraphy and PSG metrics for Wake After Sleep Onset (WASO) including; mean difference, standard deviation of difference, lower and upper limits of agreement (95%), and proportional bias regression results for each respect algorithm both non rescored and rescored.^
