# Supplementary material for: Comparison and Validation of Actigraphy Algorithms Using a Large Community Dataset: Algorithm Validation Study
Source: JMIR Form Res. 2025 Dec 11;9:e70778. doi: 10.2196/70778 (PMC12697920; doi:10.2196/70778)
Supplement: Multimedia Appendix 7 [file formative-v9-e70778-s007.docx]

Multimedia Appendix G: – ROC AUC:

**Figure S1**

Algorithm ROC curves and AUC for Apnea subgroup. ^a^


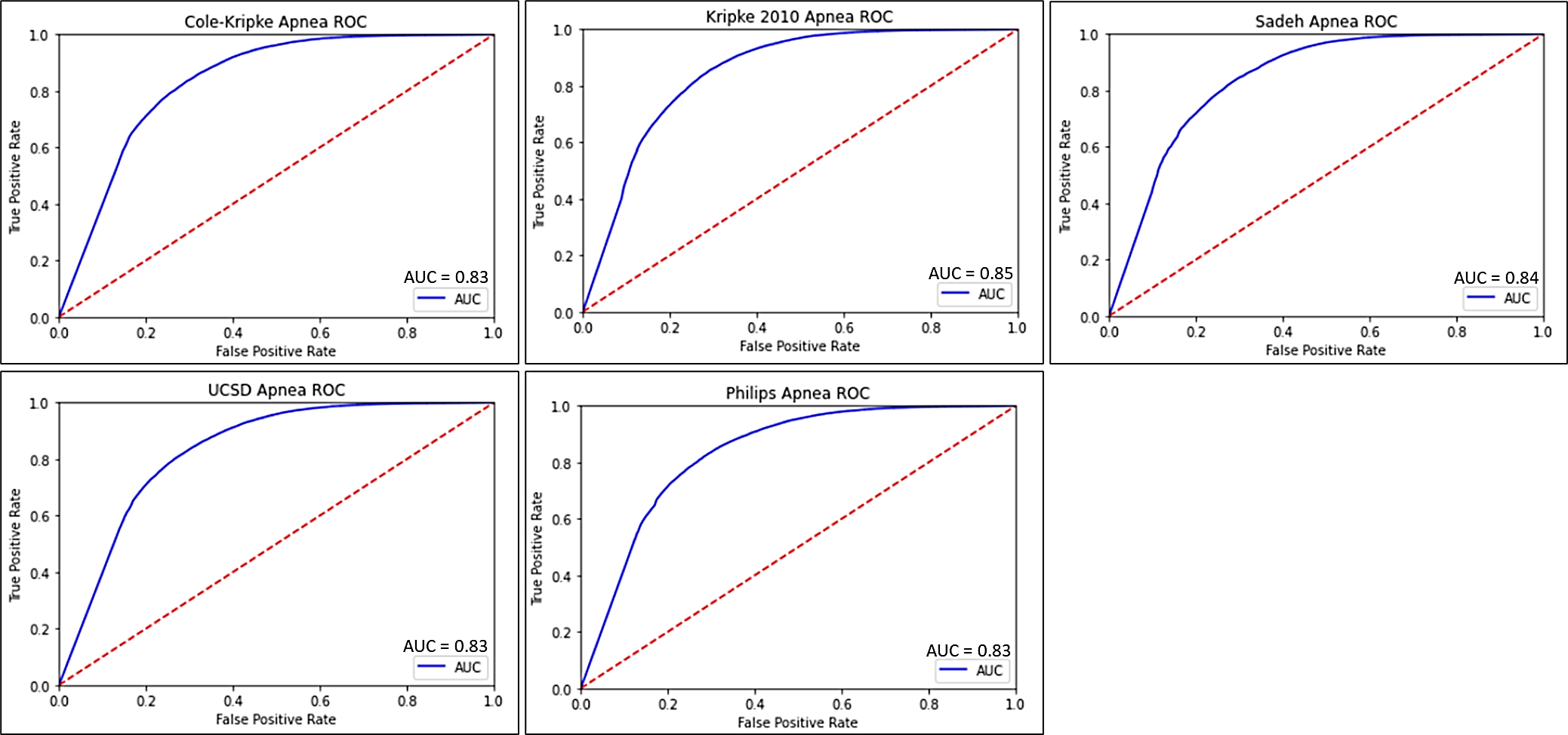


^a. Receiver Operating Characteristics curves and respective area under the curve (AUC) listed for each algorithm. Respective abbreviated algorithm names listed. The blue line represents the ROC curve for each respective algorithm. The y-axis represents the True Positive Rate while the x-axis represents the False Positive Rate. The red dotted line represents the line of random classification.^

The ROC analysis of participants with Apnea revealed Kripke 2010 has the highest AUC (0.85) followed by Sadeh (0.84), Cole-Kripke (0.83), Philips-Respironics (0.83), and UCSD (0.83). Across the board, all algorithms demonstrated excellent ability to discriminate sleep-wake (excellent performance). The ROC curves are presented with their respective AUC in Figure 1. An additional point of note is that participants who used a CPAP machine also had Apnea.

**Figure S2**

Algorithm ROC curves and AUC for CPAP subgroup. ^a^


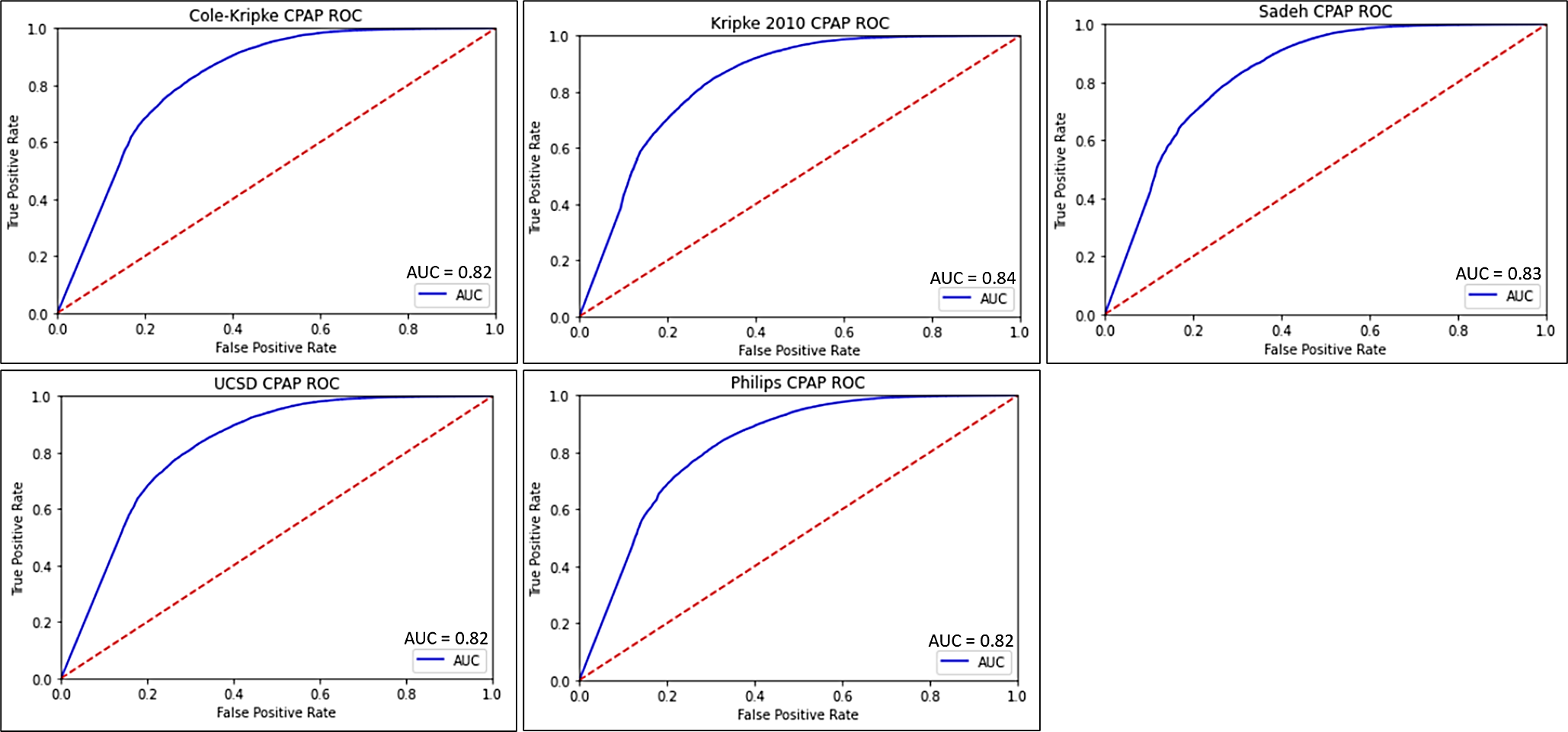


^a. Receiver Operating Characteristics curves and respective area under the curve (AUC) listed for each algorithm. Respective abbreviated algorithm names listed. The blue line represents the ROC curve for each respective algorithm. The y-axis represents the True Positive Rate while the x-axis represents the False Positive Rate. The red dotted line represents the line of random classification.^

The ROC analysis of participants with who use a CPAP device revealed Kripke 2010 has the highest AUC (0.84) followed by Sadeh (0.83), Cole-Kripke (0.82), Philips-Respironics (0.82), and UCSD (0.82). Across the board, all algorithms demonstrated excellent ability to discriminate sleep-wake (excellent performance). The ROC curves are presented with their respective AUC in Figure 2. An additional point of note is that participants who used a CPAP machine also had Apnea.

**Figure S3**

Algorithm ROC curves and AUC for Insomnia subgroup. ^a^


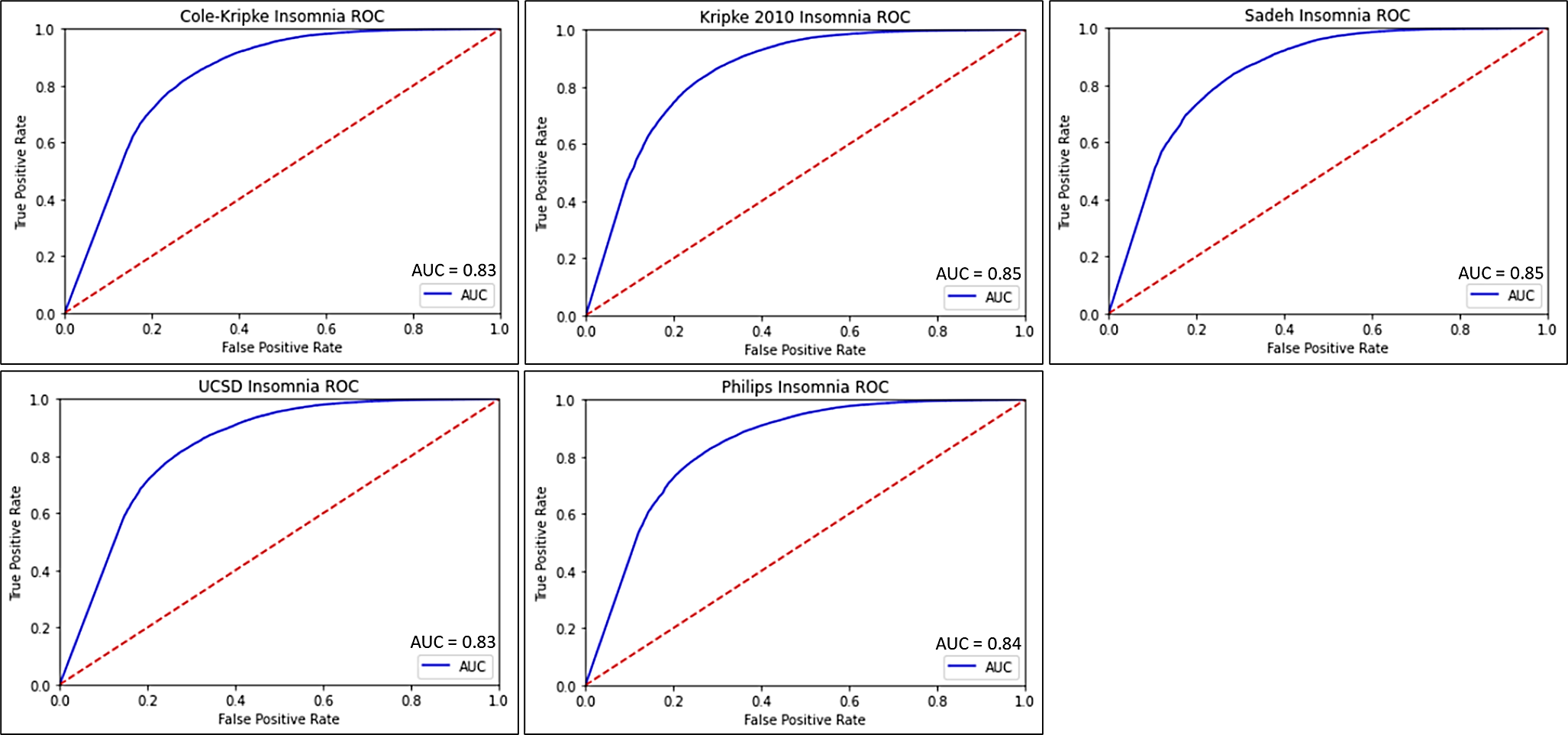


^a. Receiver Operating Characteristics curves and respective area under the curve (AUC) listed for each algorithm. Respective abbreviated algorithm names listed. The blue line represents the ROC curve for each respective algorithm. The y-axis represents the True Positive Rate while the x-axis represents the False Positive Rate. The red dotted line represents the line of random classification.^

The ROC analysis of participants with who had Insomnia revealed Kripke 2010 has the highest AUC (0.85) followed by Sadeh (0.85), Philips-Respironics (0.84), Cole-Kripke (0.83), and UCSD (0.83). Across the board, all algorithms demonstrated excellent ability to discriminate sleep-wake (excellent performance). The ROC curves are presented with their respective AUC in Figure 3.

**Figure S4**

Algorithm ROC curves and AUC for Restless Leg Syndrome subgroup. ^a^


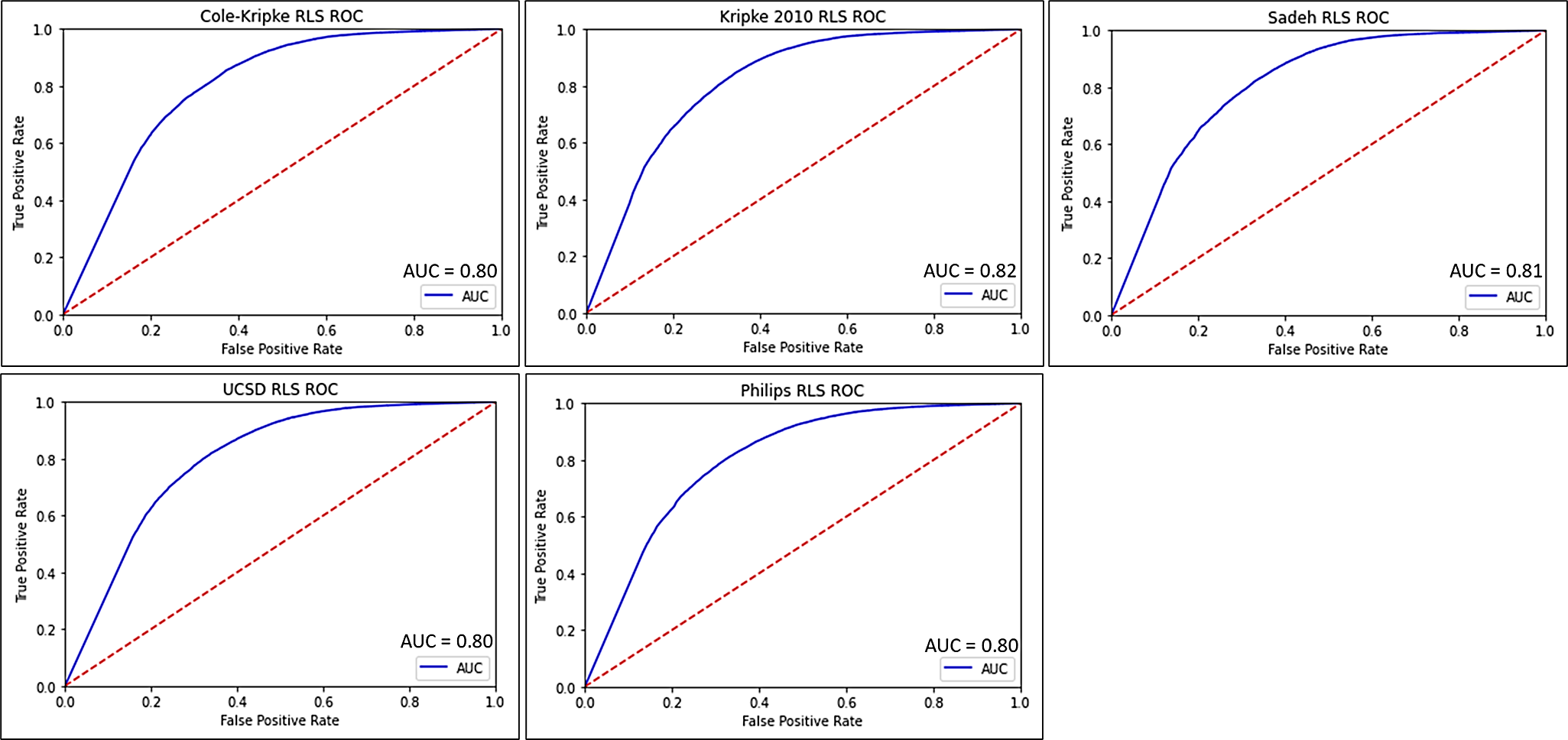


^a. Receiver Operating Characteristics curves and respective area under the curve (AUC) listed for each algorithm. Respective abbreviated algorithm names listed. The blue line represents the ROC curve for each respective algorithm. The y-axis represents the True Positive Rate while the x-axis represents the False Positive Rate. The red dotted line represents the line of random classification.^

The ROC analysis of participants with who had Restless Leg Syndrome (RLS) revealed Kripke 2010 has the highest AUC (0.82) followed by Sadeh (0.81), Philips-Respironics (0.80), Cole-Kripke (0.80), and UCSD (0.80). Across the board, all algorithms demonstrated excellent ability to discriminate sleep-wake (excellent performance). The ROC curves are presented with their respective AUC in Figure 4.
