# Supplementary material for: Comparison and Validation of Actigraphy Algorithms Using a Large Community Dataset: Algorithm Validation Study
Source: JMIR Form Res. 2025 Dec 11;9:e70778. doi: 10.2196/70778 (PMC12697920; doi:10.2196/70778)
Supplement: Multimedia Appendix 8 [file formative-v9-e70778-s008.docx]

Multimedia Appendix H: Subgroup Analysis Confusion Matrix:

**Table S1**

Confusion matrix analysis results for Apnea subgroup ^a^

| Algorithm | 𝜅 | MCC | Accuracy | Sensitivity | Specificity | Precision | F_1_-score |
| --- | --- | --- | --- | --- | --- | --- | --- |
| Cole-Kripke NRS | 0.53 | 0.56 | 0.79(0.10) | 0.94(0.05) | 0.57(0.19) | 0.77(0.12) | 0.84(0.08) |
| Cole-Kripke RS | 0.58 | 0.59 | 0.81(0.10) | 0.92(0.07) | 0.63(0.20) | 0.80(0.12) | 0.85(0.08) |
| Kripke 2010 NRS | 0.56 | 0.56 | 0.79(0.10) | 0.82(0.12) | 0.74(0.18) | 0.83(0.12) | 0.82(0.10) |
| Kripke 2010 RS | 0.55 | 0.56 | 0.78(0.10) | 0.77(0.15) | 0.80(0.18) | 0.86(0.12) | 0.80(0.11) |
| Sadeh NRS | 0.48 | 0.54 | 0.77(0.11) | 0.98(0.03) | 0.46(0.20) | 0.74(0.12) | 0.84(0.09) |
| Sadeh RS | 0.53 | 0.58 | 0.79(0.11) | 0.97(0.04) | 0.53(0.21) | 0.76(0.13) | 0.85(0.09) |
| UCSD NRS | 0.53 | 0.49 | 0.78(0.10) | 0.97(0.04) | 0.48(0.19) | 0.74(0.12) | 0.83(0.08) |
| UCSD RS | 0.54 | 0.57 | 0.79(0.10) | 0.96(0.04) | 0.55(0.20) | 0.76(0.12) | 0.85(0.08) |
| Philips20 NRS | 0.54 | 0.54 | 0.78(0.09) | 0.84(0.11) | 0.70(0.18) | 0.81(0.12) | 0.82(0.09) |
| Philips20 RS | 0.56 | 0.56 | 0.79(0.10) | 0.81(0.13) | 0.76(0.18) | 0.84(0.11) | 0.82(0.10) |
| Philips40 NRS | 0.54 | 0.55 | 0.79(0.09) | 0.89(0.09) | 0.64(0.19) | 0.79(0.12) | 0.83(0.08) |
| Philips40 RS | 0.58 | 0.58 | 0.80(0.10) | 0.87(0.10) | 0.70(0.19) | 0.82(0.12) | 0.84(0.09) |
| Philips80 NRS | 0.52 | 0.54 | 0.78(0.10) | 0.93(0.06) | 0.56(0.19) | 0.76(0.12) | 0.83(0.08) |
| Philips80 RS | 0.56 | 0.58 | 0.80(0.10) | 0.92(0.07) | 0.62(0.19) | 0.79(0.12) | 0.84(0.08) |

^a. Algorithms listed in abbreviated form: CK = Cole Kripke, K2010 = Kripke 2010, UCSD, Sadeh, and Philips. Accuracy, sensitivity, specificity, and precision listed as percent decimals. Philips results listed for each predefined threshold value. Non-rescored algorithm results (NRS) and rescored algorithm results (RS) results are listed respectively.^

**Table S2**

Confusion matrix analysis results for CPAP subgroup ^a^

| Algorithm | 𝜅 | mcc | Accuracy | Sensitivity | Specificity | Precision | F_1_-score |
| --- | --- | --- | --- | --- | --- | --- | --- |
| Cole-Kripke NRS | 0.51 | 0.56 | 0.78(0.09) | 0.94(0.05) | 0.54(0.19) | 0.76(0.12) | 0.83(0.08) |
| Cole-Kripke RS | 0.56 | 0.59 | 0.80(0.10) | 0.92(0.07) | 0.61(0.20) | 0.78(0.12) | 0.84(0.08) |
| Kripke 2010 NRS | 0.54 | 0.56 | 0.78(0.10) | 0.81(0.14) | 0.73(0.19) | 0.82(0.12) | 0.80(0.10) |
| Kripke 2010 RS | 0.54 | 0.56 | 0.77(0.12) | 0.76(0.17) | 0.79(0.18) | 0.85(0.12) | 0.79(0.13) |
| Sadeh NRS | 0.46 | 0.54 | 0.77(0.10) | 0.98(0.02) | 0.44(0.20) | 0.73(0.13) | 0.83(0.09) |
| Sadeh RS | 0.51 | 0.58 | 0.79(0.10) | 0.97(0.03) | 0.51(0.21) | 0.75(0.13) | 0.84(0.08) |
| UCSD NRS | 0.47 | 0.49 | 0.77(0.10) | 0.97(0.03) | 0.47(0.19) | 0.73(0.13) | 0.83(0.08) |
| UCSD RS | 0.51 | 0.57 | 0.79(0.10) | 0.96(0.04) | 0.52(0.21) | 0.75(0.13) | 0.84(0.08) |
| Philips20 NRS | 0.51 | 0.54 | 0.77(0.09) | 0.82(0.13) | 0.69(0.18) | 0.80(0.12) | 0.80(0.10) |
| Philips20 RS | 0.54 | 0.56 | 0.78(0.10) | 0.79(0.15) | 0.75(0.18) | 0.83(0.11) | 0.80(0.11) |
| Philips40 NRS | 0.52 | 0.55 | 0.78(0.09) | 0.88(0.10) | 0.62(0.19) | 0.78(0.12) | 0.82(0.09) |
| Philips40 RS | 0.55 | 0.58 | 0.79(0.10) | 0.86(0.11) | 0.68(0.19) | 0.81(0.12) | 0.82(0.09) |
| Philips80 NRS | 0.50 | 0.54 | 0.77(0.09) | 0.93(0.06) | 0.54(0.19) | 0.76(0.12) | 0.83(0.08) |
| Philips80 RS | 0.54 | 0.58 | 0.79(0.10) | 0.92(0.07) | 0.60(0.20) | 0.78(0.12) | 0.84(0.08) |

^a. Algorithms listed in abbreviated form: CK = Cole Kripke, K2010 = Kripke 2010, UCSD, Sadeh, and Philips.. Accuracy, sensitivity, specificity, and precision listed as percent decimals. Philips results listed for each predefined threshold value. Non-rescored algorithm results (NRS) and rescored algorithm results (RS) results are listed respectively.^

**Table S3**

Confusion matrix analysis results for Insomnia subgroup ^a^

| Algorithm | 𝜅 | MCC | Accuracy | Sensitivity | Specificity | Precision | F_1_-score |
| --- | --- | --- | --- | --- | --- | --- | --- |
| Cole-Kripke NRS | 0.52 | 0.53 | 0.79(0.10) | 0.94(0.06) | 0.55(0. 0.19) | 0.77(0.13) | 0.84(0.09) |
| Cole-Kripke RS | 0.58 | 0.58 | 0.81(0.09) | 0.93(0.07) | 0.62(0. 0.19) | 0.80(0.12) | 0.85(0.09) |
| Kripke 2010 NRS | 0.57 | 0.56 | 0.80(0.09) | 0.84(0.11) | 0.73(0. 0.18) | 0.84(0.12) | 0.83(0.09) |
| Kripke 2010 RS | 0.56 | 0.55 | 0.79(0.10) | 0.78(0.14) | 0.80(0.16) | 0.86(0.11) | 0.81(0.10) |
| Sadeh NRS | 0.47 | 0.48 | 0.78(0.10) | 0.98(0.04) | 0.45(0.19) | 0.74(0.13) | 0.84(0.09) |
| Sadeh RS | 0.53 | 0.53 | 0.80(0.10) | 0.97(0.05) | 0.52(0.20) | 0.77(0.13) | 0.85(0.09) |
| UCSD NRS | 0.48 | 0.49 | 0.78(0.10) | 0.97(0.04) | 0.47(0.19) | 0.75(0.13) | 0.84(0.09) |
| UCSD RS | 0.53 | 0.57 | 0.80(0.10) | 0.96(0.05) | 0.53(0.20) | 0.77(0.13) | 0.85(0.09) |
| Philips20 NRS | 0.55 | 0.54 | 0.79(0.08) | 0.86(0.09) | 0.68(0.18) | 0.82(0.12) | 0.83(0.08) |
| Philips20 RS | 0.57 | 0.56 | 0.80(0.09) | 0.83(0.11) | 0.75(0.17) | 0.84(0.11) | 0.83(0.09) |
| Philips40 NRS | 0.54 | 0.55 | 0.79(0.09) | 0.90(0.07) | 0.62(0.19) | 0.79(0.12) | 0.84(0.08) |
| Philips40 RS | 0.58 | 0.58 | 0.81(0.08) | 0.88(0.08) | 0.68(0.19) | 0.82(0.12) | 0.84(0.08) |
| Philips80 NRS | 0.51 | 0.54 | 0.79(0.09) | 0.94(0.06) | 0.54(0.19) | 0.77(0.13) | 0.84(0.09) |
| Philips80 RS | 0.55 | 0.58 | 0.80(0.09) | 0.93(0.07) | 0.60(0.20) | 0.79(0.13) | 0.85(0.09) |

^a. Algorithms listed in abbreviated form: CK = Cole Kripke, K2010 = Kripke 2010, UCSD, Sadeh, and Philips. Accuracy, sensitivity, specificity, and precision listed as percent decimals. Philips results listed for each predefined threshold value. Non-rescored algorithm results (NRS) and rescored algorithm results (RS) results are listed respectively.^

**Table S4**

Confusion matrix analysis results for Restless Leg Syndrome subgroup ^a^

| Algorithm | 𝜅 | mcc | Accuracy | Sensitivity | Specificity | Precision | F_1_-score |
| --- | --- | --- | --- | --- | --- | --- | --- |
| Cole-Kripke NRS | 0.48 | 0.51 | 0.77(0.10) | 0.93(0.05) | 0.52(0.19) | 0.76(0.12) | 0.83(0.08) |
| Cole-Kripke RS | 0.53 | 0.54 | 0.79(0.10) | 0.91(0.09) | 0.60(0.20) | 0.79(0.12) | 0.84(0.09) |
| Kripke 2010 NRS | 0.50 | 0.50 | 0.76(0.10) | 0.81(0.12) | 0.70(0.18) | 0.81(0.12) | 0.80(0.10) |
| Kripke 2010 RS | 0.50 | 0.51 | 0.76(0.10) | 0.76(0.14) | 0.77(0.18) | 0.84(0.13) | 0.78(0.11) |
| Sadeh NRS | 0.44 | 0.50 | 0.76(0.11) | 0.97(0.03) | 0.43(0.20) | 0.74(0.12) | 0.83(0.09) |
| Sadeh RS | 0.50 | 0.53 | 0.78(0.10) | 0.95(0.06) | 0.50(0.20) | 0.76(0.12) | 0.84(0.09) |
| UCSD NRS | 0.44 | 0.49 | 0.76(0.10) | 0.95(0.04) | 0.45(0.19) | 0.74(0.12) | 0.83(0.08) |
| UCSD RS | 0.49 | 0.52 | 0.78(0.10) | 0.94(0.06) | 0.51(0.19) | 0.76(0.12) | 0.84(0.09) |
| Philips20 NRS | 0.49 | 0.49 | 0.76(0.09) | 0.83(0.11) | 0.65(0.18) | 0.79(0.12) | 0.80(0.09) |
| Philips20 RS | 0.52 | 0.51 | 0.77(0.10) | 0.80(0.13) | 0.72(0.18) | 0.82(0.12) | 0.80(0.10) |
| Philips40 NRS | 0.49 | 0.49 | 0.77(0.09) | 0.88(0.09) | 0.59(0.19) | 0.78(0.12) | 0.82(0.08) |
| Philips40 RS | 0.52 | 0.52 | 0.78(0.09) | 0.86(0.10) | 0.65(0.19) | 0.80(0.12) | 0.82(0.09) |
| Philips80 NRS | 0.48 | 0.50 | 0.77(0.10) | 0.92(0.06) | 0.52(0.19) | 0.76(0.12) | 0.83(0.08) |
| Philips80 RS | 0.51 | 0.53 | 0.78(0.10) | 0.91(0.08) | 0.58(0.19) | 0.78(0.12) | 0.83(0.09) |

^a. Algorithms listed in abbreviated form: CK = Cole Kripke, K2010 = Kripke 2010, UCSD, Sadeh, and Philips. Accuracy, sensitivity, specificity, and precision listed as percent decimals. Philips results listed for each predefined threshold value. Non-rescored algorithm results (NRS) and rescored algorithm results (RS) results are listed respectively.^
