# Supplementary material for: Comparison and Validation of Actigraphy Algorithms Using a Large Community Dataset: Algorithm Validation Study
Source: JMIR Form Res. 2025 Dec 11;9:e70778. doi: 10.2196/70778 (PMC12697920; doi:10.2196/70778)
Supplement: Multimedia Appendix 9 [file formative-v9-e70778-s009.docx]

Multimedia Appendix I: Sleep Problem Subgroup Confusion Matrix Metric Repeated Measures Anova and Post Hoc:

**Table S1**

Repeated measures Anova for confusion matrix metrics for non-rescored algorithms Apnea.^a^

| Metric | SS | MS | *F* | *df* | η²g | Eps (ε) | | *P_uncorr._* | df*_adjusted_* | *P_adjusted_* |
| --- | --- | --- | --- | --- | --- | --- | --- | --- | --- | --- |
| Accuracy_algorithm_ | 0.02 | 0.04 | 2.97 | 6 | 0.003 | | 0.19 | .007 | 1.16 | .081 |
| Accuracy_error_ | 0.80 | 0.001 |  | 618 |  | |  |  | 119.36 |  |
| Sensitivity_algorithm_ | 2.59 | 0.43 | 205.44 | 6 | 0.35 | | 0.19 | p<.001 | 1.14 | p<.001 |
| Sensitivity_error_ | 1.30 | 0.002 |  | 618 |  | |  |  | 117.14 |  |
| Specificity_algorithm_ | 7.20 | 1.20 | 513.72 | 6 | 0.22 | | 0.21 | p<.001 | 1.25 | p<.001 |
| Specificity_error_ | 1.44 | 0.002 |  | 618 |  | |  |  | 128.94 |  |
| Precision_algorithm_ | 0.78 | 0.13 | 203.69 | 6 | 0.07 | | 0.19 | p<.001 | 1.16 | p<.001 |
| Precision_error_ | 0.40 | 0.001 |  | 618 |  | |  |  | 119.95 |  |
| f_1_-score_algorithm_ | 0.07 | 0.01 | 12.30 | 6 | 0.01 | | 0.20 | p<.001 | 1.19 | p<.001 |
| f_1_-score_error_ | 0.57 | 0.001 |  | 618 |  | |  |  | 122.15 |  |

*^a. Adjusted values for df and p values refer to respective Greenhouse Geiser corrections, SS = sum of squares MS = mean squares , and η²g = general eta squared.^*

**Table S2**

Post hoc analysis for confusion matrix metrics for non-rescored algorithms Apnea.^a^

| Metric | A | B | *T* | *df* | *P_uncorr._* | *P_adjusted_* | BF10 | Hedges *g* |
| --- | --- | --- | --- | --- | --- | --- | --- | --- |
| Sensitivity | K2010 | UCSD | -15.11 | 103 | p<.001 | p<.001 | 4.949e+24 | -1.63 |
| Sensitivity | K2010 | CK | -15.25 | 103 | p<.001 | p<.001 | 9.45e+24 | -1.30 |
| Sensitivity | K2010 | Philips-20 | -7.01 | 103 | p<.001 | p<.001 | 3.762e+07 | -0.16 |
| Sensitivity | K2010 | Philips-40 | -16.29 | 103 | p<.001 | p<.001 | 1.119e+27 | -0.65 |
| Sensitivity | K2010 | Philips-80 | -15.72 | 103 | p<.001 | p<.001 | 8.531e+25 | -1.20 |
| Sensitivity | K2010 | Sadeh | -15.11 | 103 | p<.001 | p<.001 | 5.047e+24 | -1.79 |
| Sensitivity | UCSD | CK | 12.35 | 103 | p<.001 | p<.001 | 9.622e+18 | 0.55 |
| Sensitivity | UCSD | Philips-20 | 14.17 | 103 | p<.001 | p<.001 | 6.264e+22 | 1.52 |
| Sensitivity | UCSD | Philips-40 | 12.62 | 103 | p<.001 | p<.001 | 3.565e+19 | 1.17 |
| Sensitivity | UCSD | Philips-80 | 11.39 | 103 | p<.001 | p<.001 | 8.162e+16 | 0.68 |
| Sensitivity | UCSD | Sadeh | -10.48 | 103 | p<.001 | p<.001 | 8.872e+14 | -0.34 |
| Sensitivity | CK | Philips-20 | 13.87 | 103 | p<.001 | p<.001 | 1.448e+22 | 1.17 |
| Sensitivity | CK | Philips-40 | 11.71 | 103 | p<.001 | p<.001 | 3.92e+17 | 0.73 |
| Sensitivity | CK | Philips-80 | 5.22 | 103 | p<.001 | p<.001 | 1.467e+04 | 0.14 |
| Sensitivity | CK | Sadeh | -12.66 | 103 | p<.001 | p<.001 | 4.27e+19 | -0.85 |
| Sensitivity | Philips-20 | Philips-40 | -14.93 | 103 | p<.001 | p<.001 | 2.206e+24 | -0.50 |
| Sensitivity | Philips-20 | Philips-80 | -14.62 | 103 | p<.001 | p<.001 | 5.215e+23 | -1.06 |
| Sensitivity | Philips-20 | Sadeh | -14.35 | 103 | p<.001 | p<.001 | 1.46e+23 | -1.69 |
| Sensitivity | Philips-40 | Philips-80 | -12.80 | 103 | p<.001 | p<.001 | 8.323e+19 | -0.61 |
| Sensitivity | Philips-40 | Sadeh | -12.97 | 103 | p<.001 | p<.001 | 1.928e+20 | -1.38 |
| Sensitivity | Philips-80 | Sadeh | -12.27 | 103 | p<.001 | p<.001 | 6.228e+18 | -0.97 |
| Specificity | K2010 | UCSD | 25.46 | 103 | p<.001 | p<.001 | 6.17e+42 | 1.38 |
| Specificity | K2010 | CK | 23.52 | 103 | p<.001 | p<.001 | 6.364e+39 | 0.95 |
| Specificity | K2010 | Philips-20 | 13.87 | 103 | p<.001 | p<.001 | 1.493e+22 | 0.23 |
| Specificity | K2010 | Philips-40 | 23.52 | 103 | p<.001 | p<.001 | 6.228e+39 | 0.57 |
| Specificity | K2010 | Philips-80 | 26.33 | 103 | p<.001 | p<.001 | 1.185e+44 | 1.00 |
| Specificity | K2010 | Sadeh | 23.77 | 103 | p<.001 | p<.001 | 1.556e+40 | 1.48 |
| Specificity | UCSD | CK | -23.07 | 103 | p<.001 | p<.001 | 1.216e+39 | -0.42 |
| Specificity | UCSD | Philips-20 | -23.62 | 103 | p<.001 | p<.001 | 9.035e+39 | -1.18 |
| Specificity | UCSD | Philips-40 | -22.38 | 103 | p<.001 | p<.001 | 9.361e+37 | -0.81 |
| Specificity | UCSD | Philips-80 | -18.01 | 103 | p<.001 | p<.001 | 2.111e+30 | -0.39 |
| Specificity | UCSD | Sadeh | 8.98 | 103 | p<.001 | p<.001 | 5.01e+11 | 0.11 |
| Specificity | CK | Philips-20 | -20.19 | 103 | p<.001 | p<.001 | 1.826e+34 | -0.74 |
| Specificity | CK | Philips-40 | -16.98 | 103 | p<.001 | p<.001 | 2.347e+28 | -0.38 |
| Specificity | CK | Philips-80 | 3.20 | 103 | 0.002 | 0.038 | 12.818 | 0.04 |
| Specificity | CK | Sadeh | 20.03 | 103 | p<.001 | p<.001 | 9.56e+33 | 0.53 |
| Specificity | Philips-20 | Philips-40 | 19.74 | 103 | p<.001 | p<.001 | 2.929e+33 | 0.36 |
| Specificity | Philips-20 | Philips-80 | 24.45 | 103 | p<.001 | p<.001 | 1.799e+41 | 0.79 |
| Specificity | Philips-20 | Sadeh | 22.26 | 103 | p<.001 | p<.001 | 5.897e+37 | 1.28 |
| Specificity | Philips-40 | Philips-80 | 23.75 | 103 | p<.001 | p<.001 | 1.458e+40 | 0.43 |
| Specificity | Philips-40 | Sadeh | 20.69 | 103 | p<.001 | p<.001 | 1.354e+35 | 0.92 |
| Specificity | Philips-80 | Sadeh | 16.67 | 103 | p<.001 | p<.001 | 6.07e+27 | 0.50 |
| Precision | K2010 | UCSD | 15.04 | 103 | p<.001 | p<.001 | 3.706e+24 | 0.73 |
| Precision | K2010 | CK | 13.59 | 103 | p<.001 | p<.001 | 3.852e+21 | 0.52 |
| Precision | K2010 | Philips-20 | 10.27 | 103 | p<.001 | p<.001 | 3.118e+14 | 0.17 |
| Precision | K2010 | Philips-40 | 14.03 | 103 | p<.001 | p<.001 | 3.092e+22 | 0.35 |
| Precision | K2010 | Philips-80 | 14.96 | 103 | p<.001 | p<.001 | 2.522e+24 | 0.56 |
| Precision | K2010 | Sadeh | 14.56 | 103 | p<.001 | p<.001 | 3.964e+23 | 0.77 |
| Precision | UCSD | CK | -16.32 | 103 | p<.001 | p<.001 | 1.27e+27 | -0.21 |
| Precision | UCSD | Philips-20 | -15.07 | 103 | p<.001 | p<.001 | 4.129e+24 | -0.57 |
| Precision | UCSD | Philips-40 | -14.32 | 103 | p<.001 | p<.001 | 1.224e+23 | -0.39 |
| Precision | UCSD | Philips-80 | -12.50 | 103 | p<.001 | p<.001 | 1.96e+19 | -0.18 |
| Precision | UCSD | Sadeh | 5.94 | 103 | p<.001 | p<.001 | 2.942e+05 | 0.04 |
| Precision | CK | Philips-20 | -12.73 | 103 | p<.001 | p<.001 | 5.939e+19 | -0.36 |
| Precision | CK | Philips-40 | -10.23 | 103 | p<.001 | p<.001 | 2.583e+14 | -0.18 |
| Precision | CK | Philips-80 | 4.98 | 103 | p<.001 | p<.001 | 5630.092 | 0.04 |
| Precision | CK | Sadeh | 14.66 | 103 | p<.001 | p<.001 | 6.167e+23 | 0.25 |
| Precision | Philips-20 | Philips-40 | 13.30 | 103 | p<.001 | p<.001 | 9.69e+20 | 0.18 |
| Precision | Philips-20 | Philips-80 | 15.34 | 103 | p<.001 | p<.001 | 1.487e+25 | 0.40 |
| Precision | Philips-20 | Sadeh | 14.57 | 103 | p<.001 | p<.001 | 4.053e+23 | 0.61 |
| Precision | Philips-40 | Philips-80 | 14.43 | 103 | p<.001 | p<.001 | 2.079e+23 | 0.21 |
| Precision | Philips-40 | Sadeh | 13.69 | 103 | p<.001 | p<.001 | 6.129e+21 | 0.43 |
| Precision | Philips-80 | Sadeh | 11.74 | 103 | p<.001 | p<.001 | 4.579e+17 | 0.22 |
| f1score | K2010 | UCSD | -2.95 | 103 | 0.004 | 0.082 | 6.443 | -0.22 |
| f1score | K2010 | CK | -4.56 | 103 | p<.001 | p<.001 | 1141.983 | -0.29 |
| f1score | K2010 | Philips-20 | -0.39 | 103 | .699 | 1.000 | 0.117 | -0.01 |
| f1score | K2010 | Philips-40 | -4.29 | 103 | p<.001 | p<.001 | 423.633 | -0.16 |
| f1score | K2010 | Philips-80 | -3.81 | 103 | P<.001 | .005 | 81.142 | -0.22 |
| f1score | K2010 | Sadeh | -2.92 | 103 | .004 | .090 | 5.896 | -0.23 |
| f1score | UCSD | CK | -3.95 | 103 | p<.001 | .003 | 127.917 | -0.07 |
| f1score | UCSD | Philips-20 | 3.31 | 103 | .001 | .027 | 17.171 | 0.21 |
| f1score | UCSD | Philips-40 | 1.38 | 103 | .172 | 1.000 | 0.271 | 0.06 |
| f1score | UCSD | Philips-80 | -0.09 | 103 | .930 | 1.000 | 0.109 | 0.00 |
| f1score | UCSD | Sadeh | -1.49 | 103 | .139 | 1.000 | 0.317 | -0.02 |
| f1score | CK | Philips-20 | 5.20 | 103 | p<.001 | p<.001 | 1.327e+04 | 0.28 |
| f1score | CK | Philips-40 | 4.13 | 103 | p<.001 | 0.002 | 237.225 | 0.13 |
| f1score | CK | Philips-80 | 6.74 | 103 | p<.001 | p<.001 | 1.073e+07 | 0.07 |
| f1score | CK | Sadeh | 2.12 | 103 | .036 | .763 | 0.929 | 0.05 |
| f1score | Philips-20 | Philips-40 | -5.46 | 103 | p<.001 | p<.001 | 3.847e+04 | -0.15 |
| f1score | Philips-20 | Philips-80 | -4.42 | 103 | p<.001 | p<.001 | 671.079 | -0.22 |
| f1score | Philips-20 | Sadeh | -3.26 | 103 | .002 | .032 | 15.151 | -0.23 |
| f1score | Philips-40 | Philips-80 | -2.47 | 103 | .015 | .315 | 1.969 | -0.06 |
| f1score | Philips-40 | Sadeh | -1.50 | 103 | .137 | 1.000 | 0.321 | -0.08 |
| f1score | Philips-80 | Sadeh | -0.46 | 103 | .645 | 1.000 | 0.121 | -0.01 |

*^a. Contrasts between A and B. Bonferroni correction applied. BF10 represents Bayesian factor of 10 results.^*

**Table S3**

Repeated measures Anova for confusion matrix metrics for rescored algorithms Apnea.^a^

| Metric | SS | MS | *F* | *df* | η²g | Eps (ε) | *P_uncorr._* | | df*_adjusted_* | *P_adjusted_* |
| --- | --- | --- | --- | --- | --- | --- | --- | --- | --- | --- |
| Accuracy_algorithm_ | 0.06 | 0.01 | 5.27 | 6 | 0.01 | 0.20 | p<.001 | | 1.20 | .018 |
| Accuracy_error_ | 1.09 | 0.002 |  | 618 |  |  |  | | 124.08 |  |
| Sensitivity_algorithm_ | 3.91 | 0.65 | 227.80 | 6 | 0.38 | 0.20 | p<.001 | | 1.19 | p<.001 |
| Sensitivity_error_ | 1.77 | 0.002 |  | 618 |  |  |  | | 122.26 |  |
| Specificity_algorithm_ | 7.09 | 1.18 | 372.51 | 6 | 0.21 | 0.23 | p<.001 | | 1.38 | p<.001 |
| Specificity_error_ | 1.96 | 0.003 |  | 618 |  |  |  | | 142.64 |  |
| Precision_algorithm_ | 0.89 | 0.15 | 159.00 | 6 | 0.08 | 0.21 | p<.001 | | 1.29 | p<.001 |
| Precision_error_ | 0.58 | 0.001 |  | 618 |  |  |  | | 132.56 |  |
| f_1_-score_algorithm_ | 0.26 | 0.04 | 29.14 | 6 | 0.04 | 0.20 | p<.001 | | 1.21 | p<.001 |
| f_1_-score_error_ | 0.91 | 0.001 |  | 618 |  |  | |  | 124.57 |  |

**Table S4**

Post hoc analysis for confusion matrix metrics for rescored algorithms Apnea.^a^

| Metric | A | B | *T* | *df* | *P_uncorr._* | *P_adjusted_* | BF10 | Hedges *g* |
| --- | --- | --- | --- | --- | --- | --- | --- | --- |
| Accuracy | K2010 | UCSD | -1.70 | 103 | .091 | 1.000 | 0.44 | -0.16 |
| Accuracy | K2010 | CK | -3.88 | 103 | p<.001 | .004 | 102.03 | -0.28 |
| Accuracy | K2010 | Philips-20 | -4.02 | 103 | p<.001 | .002 | 163.54 | -0.10 |
| Accuracy | K2010 | Philips-40 | -4.57 | 103 | p<.001 | p<.001 | 1154.14 | -0.22 |
| Accuracy | K2010 | Philips-80 | -3.07 | 103 | .003 | .057 | 8.84 | -0.22 |
| Accuracy | K2010 | Sadeh | -1.63 | 103 | .106 | 1.000 | 0.39 | -0.16 |
| Accuracy | UCSD | CK | -4.23 | 103 | p<.001 | .001 | 343.23 | -0.12 |
| Accuracy | UCSD | Philips-20 | 0.73 | 103 | .468 | 1.000 | 0.14 | 0.06 |
| Accuracy | UCSD | Philips-40 | -1.09 | 103 | .277 | 1.000 | 0.19 | -0.06 |
| Accuracy | UCSD | Philips-80 | -2.09 | 103 | .039 | .816 | 0.88 | -0.06 |
| Accuracy | UCSD | Sadeh | -0.39 | 103 | .698 | 1.000 | 0.12 | -0.01 |
| Accuracy | CK | Philips-20 | 3.05 | 103 | .003 | .060 | 8.41 | 0.19 |
| Accuracy | CK | Philips-40 | 1.96 | 103 | .053 | 1.000 | 0.68 | 0.07 |
| Accuracy | CK | Philips-80 | 4.44 | 103 | p<.001 | p<.001 | 723.42 | 0.06 |
| Accuracy | CK | Sadeh | 3.16 | 103 | .002 | .04 | 11.26 | 0.12 |
| Accuracy | Philips-20 | Philips-40 | -3.65 | 103 | p<.001 | .009 | 48.59 | -0.12 |
| Accuracy | Philips-20 | Philips-80 | -2.09 | 103 | .039 | .818 | 0.88 | -0.12 |
| Accuracy | Philips-20 | Sadeh | -0.73 | 103 | .469 | 1.000 | 0.14 | -0.06 |
| Accuracy | Philips-40 | Philips-80 | -0.03 | 103 | .974 | 1.000 | 0.11 | 0.00 |
| Accuracy | Philips-40 | Sadeh | 0.87 | 103 | .388 | 1.000 | 0.16 | 0.05 |
| Accuracy | Philips-80 | Sadeh | 1.48 | 103 | .143 | 1.000 | 0.31 | 0.05 |
| Sensitivity | K2010 | UCSD | -16.07 | 103 | p<.001 | p<.001 | 4.104e+26 | -1.78 |
| Sensitivity | K2010 | CK | -16.38 | 103 | p<.001 | p<.001 | 1.655e+27 | -1.37 |
| Sensitivity | K2010 | Philips-20 | -11.14 | 103 | p<.001 | p<.001 | 2.385e+16 | -0.30 |
| Sensitivity | K2010 | Philips-40 | -17.04 | 103 | p<.001 | p<.001 | 3.082e+28 | -0.82 |
| Sensitivity | K2010 | Philips-80 | -16.76 | 103 | p<.001 | p<.001 | 8.836e+27 | -1.38 |
| Sensitivity | K2010 | Sadeh | -16.02 | 103 | p<.001 | p<.001 | 3.222e+26 | -1.90 |
| Sensitivity | UCSD | CK | 11.79 | 103 | p<.001 | p<.001 | 5.998e+17 | 0.64 |
| Sensitivity | UCSD | Philips-20 | 14.70 | 103 | p<.001 | p<.001 | 7.559e+23 | 1.56 |
| Sensitivity | UCSD | Philips-40 | 12.78 | 103 | p<.001 | p<.001 | 7.825e+19 | 1.17 |
| Sensitivity | UCSD | Philips-80 | 10.60 | 103 | p<.001 | p<.001 | 1.584e+15 | 0.64 |
| Sensitivity | UCSD | Sadeh | -9.05 | 103 | p<.001 | p<.001 | 7.157e+11 | -0.28 |
| Sensitivity | CK | Philips-20 | 14.58 | 103 | p<.001 | p<.001 | 4.16e+23 | 1.12 |
| Sensitivity | CK | Philips-40 | 11.61 | 103 | p<.001 | p<.001 | 2.505e+17 | 0.64 |
| Sensitivity | CK | Philips-80 | 0.01 | 103 | 0.995 | 1 | 0.11 | 0.00 |
| Sensitivity | CK | Sadeh | -12.13 | 103 | p<.001 | p<.001 | 3.248e+18 | -0.88 |
| Sensitivity | Philips-20 | Philips-40 | -15.43 | 103 | p<.001 | p<.001 | 2.166e+25 | -0.53 |
| Sensitivity | Philips-20 | Philips-80 | -15.41 | 103 | p<.001 | p<.001 | 2.012e+25 | -1.12 |
| Sensitivity | Philips-20 | Sadeh | -14.82 | 103 | p<.001 | p<.001 | 1.299e+24 | -1.70 |
| Sensitivity | Philips-40 | Philips-80 | -13.19 | 103 | p<.001 | p<.001 | 5.718e+20 | -0.64 |
| Sensitivity | Philips-40 | Sadeh | -13.06 | 103 | p<.001 | p<.001 | 2.972e+20 | -1.35 |
| Sensitivity | Philips-80 | Sadeh | -11.45 | 103 | p<.001 | p<.001 | 1.094e+17 | -0.88 |
| Specificity | K2010 | UCSD | 21.85 | 103 | p<.001 | p<.001 | 1.235e+37 | 1.36 |
| Specificity | K2010 | CK | 18.63 | 103 | p<.001 | p<.001 | 2.977e+31 | 0.90 |
| Specificity | K2010 | Philips-20 | 11.26 | 103 | p<.001 | p<.001 | 4.295e+16 | 0.23 |
| Specificity | K2010 | Philips-40 | 18.25 | 103 | p<.001 | p<.001 | 5.884e+30 | 0.57 |
| Specificity | K2010 | Philips-80 | 21.66 | 103 | p<.001 | p<.001 | 5.847e+36 | 1.02 |
| Specificity | K2010 | Sadeh | 21.01 | 103 | p<.001 | p<.001 | 4.792e+35 | 1.44 |
| Specificity | UCSD | CK | -19.31 | 103 | p<.001 | p<.001 | 5.068e+32 | -0.44 |
| Specificity | UCSD | Philips-20 | -20.40 | 103 | p<.001 | p<.001 | 4.281e+34 | -1.15 |
| Specificity | UCSD | Philips-40 | -19.15 | 103 | p<.001 | p<.001 | 2.575e+32 | -0.79 |
| Specificity | UCSD | Philips-80 | -14.95 | 103 | p<.001 | p<.001 | 2.45e+24 | -0.36 |
| Specificity | UCSD | Sadeh | 6.28 | 103 | p<.001 | p<.001 | 1.343e+06 | 0.09 |
| Specificity | CK | Philips-20 | -16.02 | 103 | p<.001 | p<.001 | 3.252e+26 | -0.69 |
| Specificity | CK | Philips-40 | -12.72 | 103 | p<.001 | p<.001 | 5.848e+19 | -0.34 |
| Specificity | CK | Philips-80 | 5.60 | 103 | p<.001 | p<.001 | 6.83e+04 | 0.09 |
| Specificity | CK | Sadeh | 17.72 | 103 | p<.001 | p<.001 | 5.941e+29 | 0.52 |
| Specificity | Philips-20 | Philips-40 | 16.01 | 103 | p<.001 | p<.001 | 3.183e+26 | 0.35 |
| Specificity | Philips-20 | Philips-80 | 20.36 | 103 | p<.001 | p<.001 | 3.581e+34 | 0.80 |
| Specificity | Philips-20 | Sadeh | 19.73 | 103 | p<.001 | p<.001 | 2.847e+33 | 1.23 |
| Specificity | Philips-40 | Philips-80 | 18.58 | 103 | p<.001 | p<.001 | 2.386e+31 | 0.44 |
| Specificity | Philips-40 | Sadeh | 18.14 | 103 | p<.001 | p<.001 | 3.616e+30 | 0.87 |
| Specificity | Philips-80 | Sadeh | 14.80 | 103 | p<.001 | p<.001 | 1.181e+24 | 0.45 |
| Precision | K2010 | UCSD | 13.77 | 103 | p<.001 | p<.001 | 9.29e+21 | 0.77 |
| Precision | K2010 | CK | 11.84 | 103 | p<.001 | p<.001 | 7.575e+17 | 0.53 |
| Precision | K2010 | Philips-20 | 7.85 | 103 | p<.001 | p<.001 | 1.946e+09 | 0.15 |
| Precision | K2010 | Philips-40 | 11.64 | 103 | p<.001 | p<.001 | 2.815e+17 | 0.36 |
| Precision | K2010 | Philips-80 | 13.33 | 103 | p<.001 | p<.001 | 1.086e+21 | 0.60 |
| Precision | K2010 | Sadeh | 13.42 | 103 | p<.001 | p<.001 | 1.68e+21 | 0.80 |
| Precision | UCSD | CK | -14.15 | 103 | p<.001 | p<.001 | 5.577e+22 | -0.24 |
| Precision | UCSD | Philips-20 | -13.28 | 103 | p<.001 | p<.001 | 8.754e+20 | -0.63 |
| Precision | UCSD | Philips-40 | -12.73 | 103 | p<.001 | p<.001 | 6.171e+19 | -0.42 |
| Precision | UCSD | Philips-80 | -10.76 | 103 | p<.001 | p<.001 | 3.652e+15 | -0.18 |
| Precision | UCSD | Sadeh | 3.94 | 103 | p<.001 | .003 | 127.64 | 0.03 |
| Precision | CK | Philips-20 | -10.32 | 103 | p<.001 | p<.001 | 4.052e+14 | -0.39 |
| Precision | CK | Philips-40 | -8.03 | 103 | p<.001 | p<.001 | 4.69e+09 | -0.18 |
| Precision | CK | Philips-80 | 5.99 | 103 | p<.001 | p<.001 | 3.625e+05 | 0.06 |
| Precision | CK | Sadeh | 13.46 | 103 | p<.001 | p<.001 | 2.123e+21 | 0.28 |
| Precision | Philips-20 | Philips-40 | 10.44 | 103 | p<.001 | p<.001 | 7.297e+14 | 0.21 |
| Precision | Philips-20 | Philips-80 | 12.86 | 103 | p<.001 | p<.001 | 1.133e+20 | 0.45 |
| Precision | Philips-20 | Sadeh | 12.97 | 103 | p<.001 | p<.001 | 1.95e+20 | 0.67 |
| Precision | Philips-40 | Philips-80 | 12.12 | 103 | p<.001 | p<.001 | 3.003e+18 | 0.24 |
| Precision | Philips-40 | Sadeh | 12.20 | 103 | p<.001 | p<.001 | 4.534e+18 | 0.45 |
| Precision | Philips-80 | Sadeh | 10.57 | 103 | p<.001 | p<.001 | 1.364e+15 | 0.22 |
| f1score | K2010 | UCSD | -5.48 | 103 | p<.001 | p<.001 | 4.255e+04 | -0.49 |
| f1score | K2010 | CK | -6.94 | 103 | p<.001 | p<.001 | 2.704e+07 | -0.52 |
| f1score | K2010 | Philips-20 | -5.85 | 103 | p<.001 | p<.001 | 1.987e+05 | -0.16 |
| f1score | K2010 | Philips-40 | -7.19 | 103 | p<.001 | p<.001 | 8.67e+07 | -0.37 |
| f1score | K2010 | Philips-80 | -6.43 | 103 | p<.001 | p<.001 | 2.64e+06 | -0.47 |
| f1score | K2010 | Sadeh | -5.40 | 103 | p<.001 | p<.001 | 3.042e+04 | -0.50 |
| f1score | UCSD | CK | -1.32 | 103 | .188 | 1.000 | 0.25 | -0.03 |
| f1score | UCSD | Philips-20 | 4.50 | 103 | p<.001 | p<.001 | 902.62 | 0.34 |
| f1score | UCSD | Philips-40 | 2.43 | 103 | .017 | .351 | 1.80 | 0.13 |
| f1score | UCSD | Philips-80 | 0.66 | 103 | .512 | 1.000 | 0.13 | 0.02 |
| f1score | UCSD | Sadeh | -1.94 | 103 | .054 | 1.000 | 0.66 | -0.02 |
| f1score | CK | Philips-20 | 6.16 | 103 | p<.001 | p<.001 | 7.806e+05 | 0.37 |
| f1score | CK | Philips-40 | 4.79 | 103 | p<.001 | p<.001 | 2633.662 | 0.16 |
| f1score | CK | Philips-80 | 3.92 | 103 | p<.001 | .003 | 117.24 | 0.05 |
| f1score | CK | Sadeh | 0.36 | 103 | .722 | 1.000 | 0.12 | 0.01 |
| f1score | Philips-20 | Philips-40 | -6.25 | 103 | p<.001 | p<.001 | 1.158e+06 | -0.22 |
| f1score | Philips-20 | Philips-80 | -5.55 | 103 | p<.001 | p<.001 | 5.675e+04 | -0.33 |
| f1score | Philips-20 | Sadeh | -4.48 | 103 | p<.001 | p<.001 | 851.73 | -0.36 |
| f1score | Philips-40 | Philips-80 | -3.61 | 103 | p<.001 | .010 | 43.70 | -0.11 |
| f1score | Philips-40 | Sadeh | -2.55 | 103 | .012 | .254 | 2.37 | -0.15 |
| f1score | Philips-80 | Sadeh | -1.20 | 103 | .233 | 1.000 | 0.22 | -0.04 |

*^a. Contrasts between A and B. Bonferroni correction applied. BF10 represents Bayesian factor of 10 results.^*

**Table S5**

Repeated measures Anova for confusion matrix metrics for non-rescored algorithms CPAP.^a^

| Metric | SS | MS | *F* | *df* | η²g | Eps (ε) | *P_uncorr._* | df*_adjusted_* | *P_adjusted_* |
| --- | --- | --- | --- | --- | --- | --- | --- | --- | --- |
| Accuracy_algorithm_ | 0.01 | 0.001 | 1.19 | 6 | 0.003 | 0.19 | .311 | 1.14 | .286 |
| Accuracy_error_ | 0.55 | 0.002 |  | 366 |  |  |  | 69.78 |  |
| Sensitivity_algorithm_ | 1.83 | 0.30 | 104.44 | 6 | 0.34 | 0.18 | p<.001 | 1.11 | p<.001 |
| Sensitivity_error_ | 1.07 | 0.003 |  | 366 |  |  |  | 67.60 |  |
| Specificity_algorithm_ | 4.54 | 0.76 | 312.87 | 6 | 0.22 | 0.21 | p<.001 | 1.24 | p<.001 |
| Specificity_error_ | 0.88 | 0.002 |  | 366 |  |  |  | 75.54 |  |
| Precision_algorithm_ | 0.45 | 0.07 | 100.46 | 6 | 0.07 | 0.19 | p<.001 | 1.12 | p<.001 |
| Precision_error_ | 0.27 | 0.001 |  | 366 |  |  |  | 68.36 |  |
| f_1_-score_algorithm_ | 0.07 | 0.01 | 9.06 | 6 | 0.02 | 0.19 | p<.001 | 1.17 | .002 |
| f_1_-score_error_ | 0.44 | 0.001 |  | 366 |  |  |  | 71.36 |  |

*^a. Adjusted values for df and p values refer to respective Greenhouse Geiser corrections, SS = sum of squares MS = mean squares , and η²g = general eta squared.^*

**Table S6**

Post hoc analysis for confusion matrix metrics for non-rescored algorithms CPAP.^a^

| Metric | A | B | *T* | *df* | | *P_uncorr._* | *P_adjusted_* | BF10 | Hedges *g* |
| --- | --- | --- | --- | --- | --- | --- | --- | --- | --- |
| Sensitivity | K2010 | UCSD | -10.56 | 61 | p<.001 | | p<.001 | 3.213e+12 | -0.16 |
| Sensitivity | K2010 | CK | -10.68 | 61 | p<.001 | | p<.001 | 4.841e+12 | -0.28 |
| Sensitivity | K2010 | Philips-20 | -4.59 | 61 | p<.001 | | p<.001 | 846.71 | -0.10 |
| Sensitivity | K2010 | Philips-40 | -11.36 | 61 | p<.001 | | p<.001 | 5.635e+13 | -0.22 |
| Sensitivity | K2010 | Philips-80 | -10.87 | 61 | p<.001 | | p<.001 | 9.742e+12 | -0.22 |
| Sensitivity | K2010 | Sadeh | -10.58 | 61 | p<.001 | | p<.001 | 3.365e+12 | -0.16 |
| Sensitivity | UCSD | CK | 8.69 | 61 | p<.001 | | p<.001 | 3.039e+09 | -0.12 |
| Sensitivity | UCSD | Philips-20 | 10.27 | 61 | p<.001 | | p<.001 | 1.083e+12 | 0.06 |
| Sensitivity | UCSD | Philips-40 | 9.18 | 61 | p<.001 | | p<.001 | 1.928e+10 | -0.06 |
| Sensitivity | UCSD | Philips-80 | 8.49 | 61 | p<.001 | | p<.001 | 1.424e+09 | -0.06 |
| Sensitivity | UCSD | Sadeh | -7.77 | 61 | p<.001 | | p<.001 | 8.974e+07 | -0.01 |
| Sensitivity | CK | Philips-20 | 10.20 | 61 | p<.001 | | p<.001 | 8.511e+11 | 0.19 |
| Sensitivity | CK | Philips-40 | 8.86 | 61 | p<.001 | | p<.001 | 5.729e+09 | 0.07 |
| Sensitivity | CK | Philips-80 | 5.42 | 61 | p<.001 | | p<.001 | 1.462e+04 | 0.06 |
| Sensitivity | CK | Sadeh | -8.91 | 61 | p<.001 | | p<.001 | 6.916e+09 | 0.12 |
| Sensitivity | Philips-20 | Philips-40 | -10.71 | 61 | p<.001 | | p<.001 | 5.373e+12 | -0.12 |
| Sensitivity | Philips-20 | Philips-80 | -10.52 | 61 | p<.001 | | p<.001 | 2.687e+12 | -0.12 |
| Sensitivity | Philips-20 | Sadeh | -10.36 | 61 | p<.001 | | p<.001 | 1.521e+12 | -0.06 |
| Sensitivity | Philips-40 | Philips-80 | -9.22 | 61 | p<.001 | | p<.001 | 2.231e+10 | 0.00 |
| Sensitivity | Philips-40 | Sadeh | -9.38 | 61 | p<.001 | | p<.001 | 3.945e+10 | 0.05 |
| Sensitivity | Philips-80 | Sadeh | -8.94 | 61 | p<.001 | | p<.001 | 7.628e+09 | 0.05 |
| Specificity | K2010 | UCSD | 19.60 | 61 | p<.001 | | p<.001 | 6.19e+24 | -1.78 |
| Specificity | K2010 | CK | 18.50 | 61 | p<.001 | | p<.001 | 3.246e+23 | -1.37 |
| Specificity | K2010 | Philips-20 | 10.55 | 61 | p<.001 | | p<.001 | 3.018e+12 | -0.30 |
| Specificity | K2010 | Philips-40 | 18.60 | 61 | p<.001 | | p<.001 | 4.329e+23 | -0.82 |
| Specificity | K2010 | Philips-80 | 20.64 | 61 | p<.001 | | p<.001 | 9.211e+25 | -1.38 |
| Specificity | K2010 | Sadeh | 18.75 | 61 | p<.001 | | p<.001 | 6.399e+23 | -1.90 |
| Specificity | UCSD | CK | -17.61 | 61 | p<.001 | | p<.001 | 2.678e+22 | 0.64 |
| Specificity | UCSD | Philips-20 | -18.01 | 61 | p<.001 | | p<.001 | 8.359e+22 | 1.56 |
| Specificity | UCSD | Philips-40 | -16.96 | 61 | p<.001 | | p<.001 | 4.224e+21 | 1.17 |
| Specificity | UCSD | Philips-80 | -14.04 | 61 | p<.001 | | p<.001 | 5.242e+17 | 0.64 |
| Specificity | UCSD | Sadeh | 7.37 | 61 | p<.001 | | p<.001 | 1.99e+07 | -0.28 |
| Specificity | CK | Philips-20 | -15.99 | 61 | p<.001 | | p<.001 | 2.375e+20 | 1.12 |
| Specificity | CK | Philips-40 | -13.33 | 61 | p<.001 | | p<.001 | 5.118e+16 | 0.64 |
| Specificity | CK | Philips-80 | 0.64 | 61 | .520 | | 1.000 | 0.17 | 0.00 |
| Specificity | CK | Sadeh | 15.92 | 61 | p<.001 | | p<.001 | 1.892e+20 | -0.88 |
| Specificity | Philips-20 | Philips-40 | 15.78 | 61 | p<.001 | | p<.001 | 1.248e+20 | -0.53 |
| Specificity | Philips-20 | Philips-80 | 18.81 | 61 | p<.001 | | p<.001 | 7.585e+23 | -1.12 |
| Specificity | Philips-20 | Sadeh | 17.56 | 61 | p<.001 | | p<.001 | 2.353e+22 | -1.70 |
| Specificity | Philips-40 | Philips-80 | 17.72 | 61 | p<.001 | | p<.001 | 3.666e+22 | -0.64 |
| Specificity | Philips-40 | Sadeh | 16.27 | 61 | p<.001 | | p<.001 | 5.457e+20 | -1.35 |
| Specificity | Philips-80 | Sadeh | 13.58 | 61 | p<.001 | | p<.001 | 1.149e+17 | -0.88 |
| Precision | K2010 | UCSD | 10.37 | 61 | p<.001 | | p<.001 | 1.558e+12 | -1.78 |
| Precision | K2010 | CK | 9.39 | 61 | p<.001 | | p<.001 | 4.182e+10 | -1.37 |
| Precision | K2010 | Philips-20 | 7.25 | 61 | p<.001 | | p<.001 | 1.256e+07 | -0.30 |
| Precision | K2010 | Philips-40 | 9.94 | 61 | p<.001 | | p<.001 | 3.287e+11 | -0.82 |
| Precision | K2010 | Philips-80 | 10.40 | 61 | p<.001 | | p<.001 | 1.782e+12 | -1.38 |
| Precision | K2010 | Sadeh | 10.14 | 61 | p<.001 | | p<.001 | 6.691e+11 | -1.90 |
| Precision | UCSD | CK | -12.22 | 61 | p<.001 | | p<.001 | 1.149e+15 | 0.64 |
| Precision | UCSD | Philips-20 | -10.66 | 61 | p<.001 | | p<.001 | 4.503e+12 | 1.56 |
| Precision | UCSD | Philips-40 | -9.88 | 61 | p<.001 | | p<.001 | 2.583e+11 | 1.17 |
| Precision | UCSD | Philips-80 | -9.03 | 61 | p<.001 | | p<.001 | 1.095e+10 | 0.64 |
| Precision | UCSD | Sadeh | 4.59 | 61 | p<.001 | | p<.001 | 853.86 | -0.28 |
| Precision | CK | Philips-20 | -9.17 | 61 | p<.001 | | p<.001 | 1.842e+10 | 1.12 |
| Precision | CK | Philips-40 | -7.20 | 61 | p<.001 | | p<.001 | 1.049e+07 | 0.64 |
| Precision | CK | Philips-80 | 2.67 | 61 | 0.010 | | 0.205 | 3.54 | 0.00 |
| Precision | CK | Sadeh | 10.75 | 61 | p<.001 | | p<.001 | 6.342e+12 | -0.88 |
| Precision | Philips-20 | Philips-40 | 10.42 | 61 | p<.001 | | p<.001 | 1.91e+12 | -0.53 |
| Precision | Philips-20 | Philips-80 | 11.01 | 61 | p<.001 | | p<.001 | 1.601e+13 | -1.12 |
| Precision | Philips-20 | Sadeh | 10.47 | 61 | p<.001 | | p<.001 | 2.298e+12 | -1.70 |
| Precision | Philips-40 | Philips-80 | 9.77 | 61 | p<.001 | | p<.001 | 1.724e+11 | -0.64 |
| Precision | Philips-40 | Sadeh | 9.62 | 61 | p<.001 | | p<.001 | 9.716e+10 | -1.35 |
| Precision | Philips-80 | Sadeh | 8.66 | 61 | p<.001 | | p<.001 | 2.688e+09 | -0.88 |
| f1score | K2010 | UCSD | -2.60 | 61 | .012 | | .245 | 3.03 | 0.77 |
| f1score | K2010 | CK | -3.59 | 61 | p<.001 | | .014 | 38.80 | 0.53 |
| f1score | K2010 | Philips-20 | 0.07 | 61 | .947 | | 1.000 | 0.14 | 0.15 |
| f1score | K2010 | Philips-40 | -3.38 | 61 | .001 | | .026 | 21.66 | 0.36 |
| f1score | K2010 | Philips-80 | -3.13 | 61 | .003 | | .056 | 11.02 | 0.60 |
| f1score | K2010 | Sadeh | -2.56 | 61 | .013 | | .271 | 2.78 | 0.80 |
| f1score | UCSD | CK | -2.01 | 61 | .049 | | 1.000 | 0.91 | -0.24 |
| f1score | UCSD | Philips-20 | 3.12 | 61 | .003 | | .058 | 10.66 | -0.63 |
| f1score | UCSD | Philips-40 | 1.63 | 61 | .107 | | 1.000 | 0.49 | -0.42 |
| f1score | UCSD | Philips-80 | 0.60 | 61 | .554 | | 1.000 | 0.17 | -0.18 |
| f1score | UCSD | Sadeh | -1.12 | 61 | .268 | | 1.000 | 0.25 | 0.03 |
| f1score | CK | Philips-20 | 4.33 | 61 | p<.001 | | .001 | 369.15 | -0.39 |
| f1score | CK | Philips-40 | 3.35 | 61 | .001 | | .029 | 19.57 | -0.18 |
| f1score | CK | Philips-80 | 5.02 | 61 | p<.001 | | p<.001 | 3639.04 | 0.06 |
| f1score | CK | Sadeh | 0.95 | 61 | .347 | | 1.000 | 0.21 | 0.28 |
| f1score | Philips-20 | Philips-40 | -4.61 | 61 | p<.001 | | p<.001 | 901.95 | 0.21 |
| f1score | Philips-20 | Philips-80 | -3.89 | 61 | p<.001 | | .005 | 93.56 | 0.45 |
| f1score | Philips-20 | Sadeh | -3.06 | 61 | .003 | | .068 | 9.23 | 0.67 |
| f1score | Philips-40 | Philips-80 | -2.35 | 61 | .022 | | .461 | 1.77 | 0.24 |
| f1score | Philips-40 | Sadeh | -1.65 | 61 | .104 | | 1.000 | 0.50 | 0.45 |
| f1score | Philips-80 | Sadeh | -0.83 | 61 | .409 | | 1.000 | 0.19 |  |

1. *^Contrasts between A and B. Bonferroni correction applied. BF10 represents Bayesian factor of 10 results.^*

**Table S7**

Repeated measures Anova for confusion matrix metrics for rescored algorithms CPAP.^a^

| Metric | SS | MS | *F* | *df* | η²g | Eps (ε) | | *P_uncorr._* | df*_adjusted_* | | *P_adjusted_* |
| --- | --- | --- | --- | --- | --- | --- | --- | --- | --- | --- | --- |
| Accuracy_algorithm_ | 0.03 | 0.01 | 2.74 | 6 | 0.01 | 0.20 | .013 | | | 1.18 | .096 |
| Accuracy_error_ | 0.75 | 0.002 |  | 366 |  |  |  | | | 72.14 |  |
| Sensitivity_algorithm_ | 2.68 | 0.45 | 115.35 | 6 | 0.35 | 0.19 | p<.001 | | | 1.14 | p<.001 |
| Sensitivity_error_ | 1.42 | 0.004 |  | 366 |  |  |  | | | 69.61 |  |
| Specificity_algorithm_ | 4.63 | 0.77 | 237.66 | 6 | 0.22 | 0.23 | p<.001 | | | 1.36 | p<.001 |
| Specificity_error_ | 1.19 | 0.003 |  | 366 |  |  |  | | | 83.06 |  |
| Precision_algorithm_ | 0.53 | 0.09 | 83.59 | 6 | 0.08 | 0.21 | p<.001 | | | 1.24 | p<.001 |
| Precision_error_ | 0.39 | 0.001 |  | 366 |  |  |  | | | 75.80 |  |
| f_1_-score_algorithm_ | 0.20 | 0.03 | 17.27 | 6 | 0.05 | 0.20 | p<.001 | | | 1.18 | p<.001 |
| f_1_-score_error_ | 0.70 | 0.002 |  | 366 |  |  |  | | | 71.95 |  |

*^a. Adjusted values for df and p values refer to respective Greenhouse Geiser corrections, SS = sum of squares MS = mean squares , and η²g = general eta squared.^*

**Table S8**

Post hoc analysis for confusion matrix metrics for rescored algorithms CPAP.^a^

| Metric | A | B | *T* | | *df* | *P_uncorr._* | *P_adjusted_* | BF10 | Hedges *g* |
| --- | --- | --- | --- | --- | --- | --- | --- | --- | --- |
| Sensitivity | K2010 | UCSD | | -11.24 | 61 | p<.001 | p<.001 | 3.7e+13 | -1.63 |
| Sensitivity | K2010 | CK | | -11.50 | 61 | p<.001 | p<.001 | 9.408e+13 | -1.26 |
| Sensitivity | K2010 | Philips-20 | | -8.09 | 61 | p<.001 | p<.001 | 3.081e+08 | -0.23 |
| Sensitivity | K2010 | Philips-40 | | -12.11 | 61 | p<.001 | p<.001 | 8.012e+14 | -0.71 |
| Sensitivity | K2010 | Philips-80 | | -11.67 | 61 | p<.001 | p<.001 | 1.717e+14 | -1.23 |
| Sensitivity | K2010 | Sadeh-0 | | -11.19 | 61 | p<.001 | p<.001 | 3.064e+13 | -1.75 |
| Sensitivity | UCSD | Ck | | 8.24 | 61 | p<.001 | p<.001 | 5.498e+08 | 0.66 |
| Sensitivity | UCSD | Philips-20 | | 10.65 | 61 | p<.001 | p<.001 | 4.382e+12 | 1.51 |
| Sensitivity | UCSD | Philips-40 | | 9.31 | 61 | p<.001 | p<.001 | 3.058e+10 | 1.17 |
| Sensitivity | UCSD | Philips-80 | | 7.91 | 61 | p<.001 | p<.001 | 1.526e+08 | 0.70 |
| Sensitivity | UCSD | Sadeh-0 | | -6.81 | 61 | p<.001 | p<.001 | 2.423e+06 | -0.33 |
| Sensitivity | Ck | Philips-20 | | 10.80 | 61 | p<.001 | p<.001 | 7.544e+12 | 1.09 |
| Sensitivity | Ck | Philips-40 | | 8.95 | 61 | p<.001 | p<.001 | 7.953e+09 | 0.66 |
| Sensitivity | Ck | Philips-80 | | 1.53 | 61 | .131 | 1.000 | 0.42 | 0.05 |
| Sensitivity | Ck | Sadeh-0 | | -8.49 | 61 | p<.001 | p<.001 | 1.382e+09 | -0.90 |
| Sensitivity | Philips-20 | Philips-40 | | -11.04 | 61 | p<.001 | p<.001 | 1.776e+13 | -0.49 |
| Sensitivity | Philips-20 | Philips-80 | | -11.09 | 61 | p<.001 | p<.001 | 2.123e+13 | -1.05 |
| Sensitivity | Philips-20 | Sadeh-0 | | -10.68 | 61 | p<.001 | p<.001 | 4.978e+12 | -1.64 |
| Sensitivity | Philips-40 | Philips-80 | | -9.54 | 61 | p<.001 | p<.001 | 7.192e+10 | -0.61 |
| Sensitivity | Philips-40 | Sadeh-0 | | -9.44 | 61 | p<.001 | p<.001 | 5.057e+10 | -1.34 |
| Sensitivity | Philips-80 | Sadeh-0 | | -8.34 | 61 | p<.001 | p<.001 | 7.924e+08 | -0.95 |
| Specificity | K2010 | UCSD | | 17.08 | 61 | p<.001 | p<.001 | 5.922e+21 | 1.39 |
| Specificity | K2010 | Ck | | 14.65 | 61 | p<.001 | p<.001 | 3.774e+18 | 0.95 |
| Specificity | K2010 | Philips-20 | | 8.35 | 61 | p<.001 | p<.001 | 8.252e+08 | 0.24 |
| Specificity | K2010 | Philips-40 | | 14.02 | 61 | p<.001 | p<.001 | 4.997e+17 | 0.59 |
| Specificity | K2010 | Philips-80 | | 16.95 | 61 | p<.001 | p<.001 | 4.012e+21 | 1.05 |
| Specificity | K2010 | Sadeh-0 | | 16.68 | 61 | p<.001 | p<.001 | 1.845e+21 | 1.48 |
| Specificity | UCSD | Ck | | -15.50 | 61 | p<.001 | p<.001 | 5.248e+19 | -0.43 |
| Specificity | UCSD | Philips-20 | | -16.26 | 61 | p<.001 | p<.001 | 5.316e+20 | -1.17 |
| Specificity | UCSD | Philips-40 | | -15.28 | 61 | p<.001 | p<.001 | 2.671e+19 | -0.80 |
| Specificity | UCSD | Philips-80 | | -11.93 | 61 | p<.001 | p<.001 | 4.194e+14 | -0.36 |
| Specificity | UCSD | Sadeh-0 | | 5.59 | 61 | p<.001 | p<.001 | 2.71e+04 | 0.09 |
| Specificity | Ck | Philips-20 | | -12.82 | 61 | p<.001 | p<.001 | 9.204e+15 | -0.73 |
| Specificity | Ck | Philips-40 | | -10.04 | 61 | p<.001 | p<.001 | 4.633e+11 | -0.36 |
| Specificity | Ck | Philips-80 | | 3.73 | 61 | p<.001 | .009 | 58.24 | 0.07 |
| Specificity | Ck | Sadeh-0 | | 15.18 | 61 | p<.001 | p<.001 | 1.994e+19 | 0.52 |
| Specificity | Philips-20 | Philips-40 | | 13.06 | 61 | p<.001 | p<.001 | 2.083e+16 | 0.37 |
| Specificity | Philips-20 | Philips-80 | | 16.42 | 61 | p<.001 | p<.001 | 8.624e+20 | 0.82 |
| Specificity | Philips-20 | Sadeh-0 | | 16.04 | 61 | p<.001 | p<.001 | 2.753e+20 | 1.26 |
| Specificity | Philips-40 | Philips-80 | | 15.18 | 61 | p<.001 | p<.001 | 1.96e+19 | 0.45 |
| Specificity | Philips-40 | Sadeh-0 | | 14.93 | 61 | p<.001 | p<.001 | 8.942e+18 | 0.89 |
| Specificity | Philips-80 | Sadeh-0 | | 12.38 | 61 | p<.001 | p<.001 | 2.015e+15 | 0.45 |
| Precision | K2010 | UCSD | | 9.83 | 61 | p<.001 | p<.001 | 2.115e+11 | 0.77 |
| Precision | K2010 | CK | | 8.61 | 61 | p<.001 | p<.001 | 2.169e+09 | 0.54 |
| Precision | K2010 | Philips-20 | | 5.66 | 61 | p<.001 | p<.001 | 3.445e+04 | 0.16 |
| Precision | K2010 | Philips-40 | | 8.64 | 61 | p<.001 | p<.001 | 2.512e+09 | 0.36 |
| Precision | K2010 | Philips-80 | | 9.72 | 61 | p<.001 | p<.001 | 1.409e+11 | 0.61 |
| Precision | K2010 | Sadeh-0 | | 9.65 | 61 | p<.001 | p<.001 | 1.085e+11 | 0.80 |
| Precision | UCSD | CK | | -10.51 | 61 | p<.001 | p<.001 | 2.645e+12 | -0.23 |
| Precision | UCSD | Philips-20 | | -9.65 | 61 | p<.001 | p<.001 | 1.105e+11 | -0.64 |
| Precision | UCSD | Philips-40 | | -8.95 | 61 | p<.001 | p<.001 | 8.002e+09 | -0.42 |
| Precision | UCSD | Philips-80 | | -7.92 | 61 | p<.001 | p<.001 | 1.62e+08 | -0.17 |
| Precision | UCSD | Sadeh-0 | | 3.55 | 61 | p<.001 | .016 | 34.52 | 0.04 |
| Precision | CK | Philips-20 | | -7.63 | 61 | p<.001 | p<.001 | 5.374e+07 | -0.40 |
| Precision | CK | Philips-40 | | -5.57 | 61 | p<.001 | p<.001 | 2.464e+04 | -0.19 |
| Precision | CK | Philips-80 | | 4.34 | 61 | p<.001 | .001 | 380.72 | 0.06 |
| Precision | CK | Sadeh-0 | | 10.17 | 61 | p<.001 | p<.001 | 7.567e+11 | 0.27 |
| Precision | Philips-20 | Philips-40 | | 8.46 | 61 | p<.001 | p<.001 | 1.27e+09 | 0.22 |
| Precision | Philips-20 | Philips-80 | | 9.56 | 61 | p<.001 | p<.001 | 7.851e+10 | 0.47 |
| Precision | Philips-20 | Sadeh-0 | | 9.47 | 61 | p<.001 | p<.001 | 5.702e+10 | 0.67 |
| Precision | Philips-40 | Philips-80 | | 8.46 | 61 | p<.001 | p<.001 | 1.241e+09 | 0.25 |
| Precision | Philips-40 | Sadeh-0 | | 8.69 | 61 | p<.001 | p<.001 | 2.96e+09 | 0.45 |
| Precision | Philips-80 | Sadeh-0 | | 7.88 | 61 | p<.001 | p<.001 | 1.358e+08 | 0.21 |
| f1score | K2010 | UCSD | | -4.13 | 61 | p<.001 | .002 | 196.22 | -0.51 |
| f1score | K2010 | CK | | -5.13 | 61 | p<.001 | p<.001 | 5337.78 | -0.53 |
| f1score | K2010 | Philips-20 | | -3.81 | 61 | p<.001 | 0.007 | 74.03 | -0.13 |
| f1score | K2010 | Philips-40 | | -5.12 | 61 | p<.001 | p<.001 | 5149.41 | -0.35 |
| f1score | K2010 | Philips-80 | | -4.68 | 61 | p<.001 | p<.001 | 1133.35 | -0.47 |
| f1score | K2010 | Sadeh-0 | | -4.08 | 61 | p<.001 | .003 | 164.31 | -0.53 |
| f1score | UCSD | CK | | -0.58 | 61 | .563 | 1.000 | 0.16 | -0.02 |
| f1score | UCSD | Philips-20 | | 3.83 | 61 | p<.001 | .006 | 76.88 | 0.41 |
| f1score | UCSD | Philips-40 | | 2.33 | 61 | .023 | .483 | 1.70 | 0.18 |
| f1score | UCSD | Philips-80 | | 1.25 | 61 | .217 | 1.000 | 0.29 | 0.05 |
| f1score | UCSD | Sadeh-0 | | -1.45 | 61 | .154 | 1.000 | 0.37 | -0.02 |
| f1score | CK | Philips-20 | | 5.04 | 61 | p<.001 | p<.001 | 3877.22 | 0.43 |
| f1score | CK | Philips-40 | | 4.08 | 61 | p<.001 | .003 | 166.68 | 0.20 |
| f1score | CK | Philips-80 | | 3.95 | 61 | p<.001 | .004 | 110.58 | 0.07 |
| f1score | CK | Sadeh-0 | | 0.00 | 61 | .999 | 1.000 | 0.14 | 0.00 |
| f1score | Philips-20 | Philips-40 | | -4.89 | 61 | p<.001 | p<.001 | 2351.79 | -0.24 |
| f1score | Philips-20 | Philips-80 | | -4.45 | 61 | p<.001 | p<.001 | 540.50 | -0.37 |
| f1score | Philips-20 | Sadeh-0 | | -3.78 | 61 | p<.001 | .007 | 67.74 | -0.43 |
| f1score | Philips-40 | Philips-80 | | -2.99 | 61 | .004 | .085 | 7.60 | -0.13 |
| f1score | Philips-40 | Sadeh-0 | | -2.35 | 61 | .022 | .465 | 1.76 | -0.20 |
| f1score | Philips-80 | Sadeh-0 | | -1.45 | 61 | .153 | 1.000 | 0.37 | -0.07 |

*^a. Contrasts between A and B. Bonferroni correction applied. BF10 represents Bayesian factor of 10 results.^*

**Table S9**

Repeated measures Anova for confusion matrix metrics for non-rescored algorithms Insomnia.^a^

| Metric | SS | MS | *F* | df | η²g | Eps (ε) | *P_uncorr._* | df*_adjusted_* | *P_adjusted_* |
| --- | --- | --- | --- | --- | --- | --- | --- | --- | --- |
| Accuracy_algorithm_ | 0.03 | 0.004 | 3.85 | 6 | 0.005 | 0.19 | p<.001 | 1.17 | .046 |
| Accuracy_error_ | 0.55 | 0.001 |  | 498 |  |  |  | 96.97 |  |
| Sensitivity_algorithm_ | 1.56 | 0.26 | 262.00 | 6 | 0.34 | 0.20 | p<.001 | 1.23 | p<.001 |
| Sensitivity_error_ | 0.49 | 0.001 |  | 498 |  |  |  | 101.74 |  |
| Specificity_algorithm_ | 5.82 | 0.97 | 578.18 | 6 | 0.21 | 0.24 | p<.001 | 1.41 | p<.001 |
| Specificity_error_ | 0.84 | 0.002 |  | 498 |  |  |  | 117.11 |  |
| Precision_algorithm_ | 0.57 | 0.09 | 209.90 | 6 | 0.06 | 0.21 | p<.001 | 1.25 | p<.001 |
| Precision_error_ | 0.22 | 0.0005 |  | 498 |  |  |  | 103.48 |  |
| f_1_-score_algorithm_ | 0.02 | 0.003 | 4.76 | 6 | 0.004 | 0.20 | p<.001 | 1.20 | .025 |
| f_1_-score_error_ | 0.30 | 0.001 |  | 498 |  |  |  | 99.29 |  |

*^a. Adjusted values for df and p values refer to respective Greenhouse Geiser corrections, SS = sum of squares MS = mean squares , and η²g = general eta squared.^*

**Table S10**

Post hoc analysis for confusion matrix metrics for non-rescored algorithms Insomnia.^a^

| Metric | A | B | *T* | *df* | *P_uncorr._* | *P_adjusted_* | BF10 | Hedges *g* | |
| --- | --- | --- | --- | --- | --- | --- | --- | --- | --- |
| Accuracy | K2010 | UCSD | 1.99 | 83 | .049 | 1.000 | 0.79 | 0.18 |  |
| Accuracy | K2010 | CK | 0.34 | 83 | .732 | 1.000 | 0.13 | 0.02 |  |
| Accuracy | K2010 | Philips-20 | 1.77 | 83 | .080 | 1.000 | 0.54 | 0.05 |  |
| Accuracy | K2010 | Philips-40 | 0.45 | 83 | .652 | 1.000 | 0.13 | 0.02 |  |
| Accuracy | K2010 | Philips-80 | 1.31 | 83 | .192 | 1.000 | 0.28 | 0.09 |  |
| Accuracy | K2010 | Sadeh | 1.82 | 83 | .073 | 1.000 | 0.58 | 0.17 |  |
| Accuracy | UCSD | CK | -6.24 | 83 | p<.001 | p<.001 | 6.76e+05 | -0.15 |  |
| Accuracy | UCSD | Philips-20 | -1.72 | 83 | .089 | 1.000 | 0.50 | -0.13 |  |
| Accuracy | UCSD | Philips-40 | -3.09 | 83 | .003 | .057 | 9.63 | -0.16 |  |
| Accuracy | UCSD | Philips-80 | -3.47 | 83 | .001 | .017 | 28.42 | -0.08 |  |
| Accuracy | UCSD | Sadeh | -0.17 | 83 | .867 | 1.000 | 0.12 | 0.00 |  |
| Accuracy | CK | Philips-20 | 0.46 | 83 | .645 | 1.000 | 0.13 | 0.03 |  |
| Accuracy | CK | Philips-40 | -0.10 | 83 | .918 | 1.000 | 0.12 | 0.00 |  |
| Accuracy | CK | Philips-80 | 5.00 | 83 | p<.001 | p<.001 | 4989.01 | 0.06 |  |
| Accuracy | CK | Sadeh | 4.57 | 83 | p<.001 | p<.001 | 1027.29 | 0.14 |  |
| Accuracy | Philips-20 | Philips-40 | -1.07 | 83 | .288 | 1.000 | 0.21 | -0.03 |  |
| Accuracy | Philips-20 | Philips-80 | 0.78 | 83 | .440 | 1.000 | 0.16 | 0.04 |  |
| Accuracy | Philips-20 | Sadeh | 1.54 | 83 | .127 | 1.000 | 0.38 | 0.13 |  |
| Accuracy | Philips-40 | Philips-80 | 2.49 | 83 | .015 | .308 | 2.20 | 0.07 |  |
| Accuracy | Philips-40 | Sadeh | 2.64 | 83 | .010 | .211 | 3.06 | 0.15 |  |
| Accuracy | Philips-80 | Sadeh | 2.54 | 83 | .013 | .273 | 2.44 | 0.08 |  |
| Sensitivity | K2010 | UCSD | -16.33 | 83 | p<.001 | p<.001 | 2.931e+24 | -1.58 |  |
| Sensitivity | K2010 | CK | -17.29 | 83 | p<.001 | p<.001 | 1.064e+26 | -1.21 |  |
| Sensitivity | K2010 | Philips-20 | -6.48 | 83 | p<.001 | p<.001 | 1.862e+06 | -0.20 |  |
| Sensitivity | K2010 | Philips-40 | -14.60 | 83 | p<.001 | p<.001 | 3.313e+21 | -0.71 |  |
| Sensitivity | K2010 | Philips-80 | -15.94 | 83 | p<.001 | p<.001 | 6.402e+23 | -1.19 |  |
| Sensitivity | K2010 | Sadeh | -16.22 | 83 | p<.001 | p<.001 | 1.88e+24 | -1.76 |  |
| Sensitivity | UCSD | CK | 10.60 | 83 | p<.001 | p<.001 | 1.382e+14 | 0.43 |  |
| Sensitivity | UCSD | Philips-20 | 17.21 | 83 | p<.001 | p<.001 | 7.866e+25 | 1.53 |  |
| Sensitivity | UCSD | Philips-40 | 15.94 | 83 | p<.001 | p<.001 | 6.453e+23 | 1.07 |  |
| Sensitivity | UCSD | Philips-80 | 14.23 | 83 | p<.001 | p<.001 | 7.277e+20 | 0.54 |  |
| Sensitivity | UCSD | Sadeh | -10.87 | 83 | p<.001 | p<.001 | 4.616e+14 | -0.30 |  |
| Sensitivity | CK | Philips-20 | 17.76 | 83 | p<.001 | p<.001 | 6.043e+26 | 1.10 |  |
| Sensitivity | CK | Philips-40 | 15.27 | 83 | p<.001 | p<.001 | 4.818e+22 | 0.61 |  |
| Sensitivity | CK | Philips-80 | 2.97 | 83 | .004 | .082 | 7.02 | 0.07 |  |
| Sensitivity | CK | Sadeh | -11.78 | 83 | p<.001 | p<.001 | 2.467e+16 | -0.68 |  |
| Sensitivity | Philips-20 | Philips-40 | -17.44 | 83 | p<.001 | p<.001 | 1.866e+26 | -0.55 |  |
| Sensitivity | Philips-20 | Philips-80 | -17.28 | 83 | p<.001 | p<.001 | 1.017e+26 | -1.08 |  |
| Sensitivity | Philips-20 | Sadeh | -17.21 | 83 | p<.001 | p<.001 | 7.99e+25 | -1.74 |  |
| Sensitivity | Philips-40 | Philips-80 | -15.43 | 83 | p<.001 | p<.001 | 9.036e+22 | -0.57 |  |
| Sensitivity | Philips-40 | Sadeh | -16.00 | 83 | p<.001 | p<.001 | 8.138e+23 | -1.32 |  |
| Sensitivity | Philips-80 | Sadeh | -14.61 | 83 | p<.001 | p<.001 | 3.408e+21 | -0.82 |  |
| Specificity | K2010 | UCSD | 28.13 | 83 | p<.001 | p<.001 | 5.643e+40 | 1.40 |  |
| Specificity | K2010 | CK | 24.53 | 83 | p<.001 | p<.001 | 2.486e+36 | 0.97 |  |
| Specificity | K2010 | Philips-20 | 13.73 | 83 | p<.001 | p<.001 | 9.413e+19 | 0.25 |  |
| Specificity | K2010 | Philips-40 | 25.21 | 83 | p<.001 | p<.001 | 1.81e+37 | 0.60 |  |
| Specificity | K2010 | Philips-80 | 27.84 | 83 | p<.001 | p<.001 | 2.573e+40 | 1.03 |  |
| Specificity | K2010 | Sadeh | 26.81 | 83 | p<.001 | p<.001 | 1.619e+39 | 1.51 |  |
| Specificity | UCSD | CK | -25.80 | 83 | p<.001 | p<.001 | 9.809e+37 | -0.40 |  |
| Specificity | UCSD | Philips-20 | -24.98 | 83 | p<.001 | p<.001 | 9.264e+36 | -1.14 |  |
| Specificity | UCSD | Philips-40 | -22.74 | 83 | p<.001 | p<.001 | 1.132e+34 | -0.77 |  |
| Specificity | UCSD | Philips-80 | -18.10 | 83 | p<.001 | p<.001 | 2.049e+27 | -0.35 |  |
| Specificity | UCSD | Sadeh | 9.00 | 83 | p<.001 | p<.001 | 1.133e+11 | 0.12 |  |
| Specificity | CK | Philips-20 | -19.60 | 83 | p<.001 | p<.001 | 4.039e+29 | -0.72 |  |
| Specificity | CK | Philips-40 | -14.56 | 83 | p<.001 | p<.001 | 2.788e+21 | -0.36 |  |
| Specificity | CK | Philips-80 | 3.99 | 83 | p<.001 | .003 | 141.79 | 0.05 |  |
| Specificity | CK | Sadeh | 21.98 | 83 | p<.001 | p<.001 | 1.064e+33 | 0.52 |  |
| Specificity | Philips-20 | Philips-40 | 21.42 | 83 | p<.001 | p<.001 | 1.756e+32 | 0.35 |  |
| Specificity | Philips-20 | Philips-80 | 24.81 | 83 | p<.001 | p<.001 | 5.782e+36 | 0.77 |  |
| Specificity | Philips-20 | Sadeh | 24.16 | 83 | p<.001 | p<.001 | 8.486e+35 | 1.25 |  |
| Specificity | Philips-40 | Philips-80 | 21.52 | 83 | p<.001 | p<.001 | 2.424e+32 | 0.41 |  |
| Specificity | Philips-40 | Sadeh | 21.83 | 83 | p<.001 | p<.001 | 6.467e+32 | 0.88 |  |
| Specificity | Philips-80 | Sadeh | 17.96 | 83 | p<.001 | p<.001 | 1.23e+27 | 0.47 |  |
| Precision | K2010 | UCSD | 15.78 | 83 | p<.001 | p<.001 | 3.428e+23 | 0.69 |  |
| Precision | K2010 | CK | 14.78 | 83 | p<.001 | p<.001 | 6.697e+21 | 0.50 |  |
| Precision | K2010 | Philips-20 | 10.09 | 83 | p<.001 | p<.001 | 1.44e+13 | 0.16 |  |
| Precision | K2010 | Philips-40 | 14.68 | 83 | p<.001 | p<.001 | 4.481e+21 | 0.34 |  |
| Precision | K2010 | Philips-80 | 15.91 | 83 | p<.001 | p<.001 | 5.751e+23 | 0.54 |  |
| Precision | K2010 | Sadeh | 15.47 | 83 | p<.001 | p<.001 | 1.044e+23 | 0.73 |  |
| Precision | UCSD | CK | -14.56 | 83 | p<.001 | p<.001 | 2.763e+21 | -0.18 |  |
| Precision | UCSD | Philips-20 | -14.38 | 83 | p<.001 | p<.001 | 1.355e+21 | -0.53 |  |
| Precision | UCSD | Philips-40 | -14.40 | 83 | p<.001 | p<.001 | 1.444e+21 | -0.35 |  |
| Precision | UCSD | Philips-80 | -12.00 | 83 | p<.001 | p<.001 | 6.312e+16 | -0.15 |  |
| Precision | UCSD | Sadeh | 5.52 | 83 | p<.001 | p<.001 | 3.683e+04 | 0.03 |  |
| Precision | CK | Philips-20 | -12.26 | 83 | p<.001 | p<.001 | 1.926e+17 | -0.34 |  |
| Precision | CK | Philips-40 | -10.38 | 83 | p<.001 | p<.001 | 5.175e+13 | -0.16 |  |
| Precision | CK | Philips-80 | 4.72 | 83 | p<.001 | p<.001 | 1768.749 | 0.03 |  |
| Precision | CK | Sadeh | 13.64 | 83 | p<.001 | p<.001 | 6.563e+19 | 0.22 |  |
| Precision | Philips-20 | Philips-40 | 12.20 | 83 | p<.001 | p<.001 | 1.473e+17 | 0.18 |  |
| Precision | Philips-20 | Philips-80 | 14.35 | 83 | p<.001 | p<.001 | 1.205e+21 | 0.38 |  |
| Precision | Philips-20 | Sadeh | 14.19 | 83 | p<.001 | p<.001 | 6.201e+20 | 0.56 |  |
| Precision | Philips-40 | Philips-80 | 14.59 | 83 | p<.001 | p<.001 | 3.092e+21 | 0.20 |  |
| Precision | Philips-40 | Sadeh | 13.96 | 83 | p<.001 | p<.001 | 2.428e+20 | 0.38 |  |
| Precision | Philips-80 | Sadeh | 11.60 | 83 | p<.001 | p<.001 | 1.111e+16 | 0.18 |  |
| f1score | K2010 | UCSD | -1.54 | 83 | .128 | 1.000 | 0.37 | -0.11 |  |
| f1score | K2010 | CK | -3.11 | 83 | .003 | .054 | 10.20 | -0.18 |  |
| f1score | K2010 | Philips-20 | -0.36 | 83 | .717 | 1.000 | 0.13 | -0.01 |  |
| f1score | K2010 | Philips-40 | -2.78 | 83 | .007 | .142 | 4.31 | -0.12 |  |
| f1score | K2010 | Philips-80 | -2.17 | 83 | .033 | .698 | 1.10 | -0.13 |  |
| f1score | K2010 | Sadeh | -1.72 | 83 | .089 | 1.000 | 0.49 | -0.13 |  |
| f1score | UCSD | CK | -3.22 | 83 | .002 | .039 | 13.68 | -0.06 |  |
| f1score | UCSD | Philips-20 | 1.78 | 83 | .079 | 1.000 | 0.54 | 0.10 |  |
| f1score | UCSD | Philips-40 | -0.03 | 83 | .978 | 1.000 | 0.12 | 0.00 |  |
| f1score | UCSD | Philips-80 | -0.80 | 83 | .425 | 1.000 | 0.16 | -0.01 |  |
| f1score | UCSD | Sadeh | -2.54 | 83 | .013 | .272 | 2.45 | -0.02 |  |
| f1score | CK | Philips-20 | 3.90 | 83 | p<.001 | .004 | 106.91 | 0.17 |  |
| f1score | CK | Philips-40 | 2.70 | 83 | .008 | .177 | 3.56 | 0.06 |  |
| f1score | CK | Philips-80 | 4.41 | 83 | p<.001 | .001 | 574.15 | 0.05 |  |
| f1score | CK | Sadeh | 1.58 | 83 | .118 | 1.000 | 0.40 | 0.04 |  |
| f1score | Philips-20 | Philips-40 | -4.54 | 83 | p<.001 | p<.001 | 932.19 | -0.11 |  |
| f1score | Philips-20 | Philips-80 | -2.78 | 83 | .007 | .140 | 4.36 | -0.12 |  |
| f1score | Philips-20 | Sadeh | -1.99 | 83 | .050 | 1.000 | 0.78 | -0.13 |  |
| f1score | Philips-40 | Philips-80 | -0.64 | 83 | .526 | 1.000 | 0.15 | -0.01 |  |
| f1score | Philips-40 | Sadeh | -0.50 | 83 | .616 | 1.000 | 0.14 | -0.02 |  |
| f1score | Philips-80 | Sadeh | -0.34 | 83 | .736 | 1.000 | 0.13 | -0.01 |  |

*^a. Contrasts between A and B. Bonferroni correction applied. BF10 represents Bayesian factor of 10 results.^*

**Table S11**

Repeated measures Anova for confusion matrix metrics for rescored algorithms Insomnia.^a^

| Metric | SS | MS | *F* | *df* | η²g | Eps (ε) | *P_uncorr._* | df*_adjusted_* | *P_adjusted_* |
| --- | --- | --- | --- | --- | --- | --- | --- | --- | --- |
| Accuracy_algorithm_ | 0.03 | 0.005 | 3.21 | 6 | 0.01 | 0.21 | .004 | 1.17 | .067 |
| Accuracy_error_ | 0.79 | 0.002 |  | 498 |  |  |  | 96.97 |  |
| Sensitivity_algorithm_ | 2.60 | 0.43 | 253.63 | 6 | 0.36 | 0.21 | p<.001 | 1.23 | p<.001 |
| Sensitivity_error_ | 0.85 | 0.002 |  | 498 |  |  |  | 101.74 |  |
| Specificity_algorithm_ | 5.83 | 0.97 | 452.29 | 6 | 0.21 | 0.28 | p<.001 | 1.41 | p<.001 |
| Specificity_error_ | 1.07 | 0.002 |  | 498 |  |  |  | 117.11 |  |
| Precision_algorithm_ | 0.65 | 0.11 | 186.05 | 6 | 0.07 | 0.23 | p<.001 | 1.25 | p<.001 |
| Precision_error_ | 0.29 | 0.001 |  | 498 |  |  |  | 103.48 |  |
| f_1_-score_algorithm_ | 0.13 | 0.02 | 19.75 | 6 | 0.03 | 0.21 | p<.001 | 1.20 | p<.001 |
| f_1_-score_error_ | 0.54 | 0.001 |  | 498 |  |  |  | 99.29 |  |

*^a. Adjusted values for df and p values refer to respective Greenhouse Geiser corrections, SS = sum of squares MS = mean squares , and η²g = general eta squared.^*

**Table S12**

Post hoc analysis for confusion matrix metrics for rescored algorithms Insomnia.^a^

| Metric | A | B | *T* | *df* | *P_uncorr._* | *P_adjusted_* | BF10 | Hedges *g* |
| --- | --- | --- | --- | --- | --- | --- | --- | --- |
| Sensitivity | K2010 | UCSD | -16.42 | 83 | p<.001 | p<.001 | 4.023e+24 | -1.71 |
| Sensitivity | K2010 | CK | -16.88 | 83 | p<.001 | p<.001 | 2.34e+25 | -1.31 |
| Sensitivity | K2010 | Philips-20 | -9.57 | 83 | p<.001 | p<.001 | 1.432e+12 | -0.37 |
| Sensitivity | K2010 | Philips-40 | -14.65 | 83 | p<.001 | p<.001 | 4.002e+21 | -0.90 |
| Sensitivity | K2010 | Philips-80 | -15.89 | 83 | p<.001 | p<.001 | 5.252e+23 | -1.35 |
| Sensitivity | K2010 | Sadeh | -16.39 | 83 | p<.001 | p<.001 | 3.626e+24 | -1.82 |
| Sensitivity | UCSD | CK | 11.02 | 83 | p<.001 | p<.001 | 8.804e+14 | 0.49 |
| Sensitivity | UCSD | Philips-20 | 16.85 | 83 | p<.001 | p<.001 | 2.111e+25 | 1.54 |
| Sensitivity | UCSD | Philips-40 | 15.88 | 83 | p<.001 | p<.001 | 5.184e+23 | 1.09 |
| Sensitivity | UCSD | Philips-80 | 12.34 | 83 | p<.001 | p<.001 | 2.759e+17 | 0.50 |
| Sensitivity | UCSD | Sadeh | -7.90 | 83 | p<.001 | p<.001 | 8.312e+08 | -0.22 |
| Sensitivity | CK | Philips-20 | 16.74 | 83 | p<.001 | p<.001 | 1.37e+25 | 1.06 |
| Sensitivity | CK | Philips-40 | 13.40 | 83 | p<.001 | p<.001 | 2.39e+19 | 0.55 |
| Sensitivity | CK | Philips-80 | -0.53 | 83 | .601 | 1.000 | 0.14 | -0.01 |
| Sensitivity | CK | Sadeh | -11.99 | 83 | p<.001 | p<.001 | 6.117e+16 | -0.67 |
| Sensitivity | Philips-20 | Philips-40 | -15.91 | 83 | p<.001 | p<.001 | 5.832e+23 | -0.58 |
| Sensitivity | Philips-20 | Philips-80 | -16.57 | 83 | p<.001 | p<.001 | 7.238e+24 | -1.11 |
| Sensitivity | Philips-20 | Sadeh | -16.99 | 83 | p<.001 | p<.001 | 3.546e+25 | -1.69 |
| Sensitivity | Philips-40 | Philips-80 | -14.44 | 83 | p<.001 | p<.001 | 1.733e+21 | -0.58 |
| Sensitivity | Philips-40 | Sadeh | -16.00 | 83 | p<.001 | p<.001 | 8.321e+23 | -1.26 |
| Sensitivity | Philips-80 | Sadeh | -12.91 | 83 | p<.001 | p<.001 | 3.035e+18 | -0.69 |
| Specificity | K2010 | UCSD | 24.79 | 83 | p<.001 | p<.001 | 5.328e+36 | 1.45 |
| Specificity | K2010 | CK | 21.20 | 83 | p<.001 | p<.001 | 8.431e+31 | 1.00 |
| Specificity | K2010 | Philips-20 | 11.38 | 83 | p<.001 | p<.001 | 4.246e+15 | 0.31 |
| Specificity | K2010 | Philips-40 | 19.48 | 83 | p<.001 | p<.001 | 2.679e+29 | 0.67 |
| Specificity | K2010 | Philips-80 | 22.91 | 83 | p<.001 | p<.001 | 1.949e+34 | 1.12 |
| Specificity | K2010 | Sadeh | 24.46 | 83 | p<.001 | p<.001 | 2.082e+36 | 1.54 |
| Specificity | UCSD | CK | -20.66 | 83 | p<.001 | p<.001 | 1.438e+31 | -0.43 |
| Specificity | UCSD | Philips-20 | -22.98 | 83 | p<.001 | p<.001 | 2.413e+34 | -1.13 |
| Specificity | UCSD | Philips-40 | -20.66 | 83 | p<.001 | p<.001 | 1.446e+31 | -0.75 |
| Specificity | UCSD | Philips-80 | -15.56 | 83 | p<.001 | p<.001 | 1.502e+23 | -0.32 |
| Specificity | UCSD | Sadeh | 5.31 | 83 | p<.001 | p<.001 | 1.591e+04 | 0.08 |
| Specificity | CK | Philips-20 | -17.53 | 83 | p<.001 | p<.001 | 2.618e+26 | -0.68 |
| Specificity | CK | Philips-40 | -11.87 | 83 | p<.001 | p<.001 | 3.601e+16 | -0.32 |
| Specificity | CK | Philips-80 | 6.38 | 83 | p<.001 | p<.001 | 1.196e+06 | 0.11 |
| Specificity | CK | Sadeh | 19.23 | 83 | p<.001 | p<.001 | 1.135e+29 | 0.51 |
| Specificity | Philips-20 | Philips-40 | 16.84 | 83 | p<.001 | p<.001 | 1.977e+25 | 0.36 |
| Specificity | Philips-20 | Philips-80 | 21.23 | 83 | p<.001 | p<.001 | 9.437e+31 | 0.80 |
| Specificity | Philips-20 | Sadeh | 22.66 | 83 | p<.001 | p<.001 | 8.926e+33 | 1.21 |
| Specificity | Philips-40 | Philips-80 | 17.34 | 83 | p<.001 | p<.001 | 1.296e+26 | 0.43 |
| Specificity | Philips-40 | Sadeh | 20.40 | 83 | p<.001 | p<.001 | 6.077e+30 | 0.84 |
| Specificity | Philips-80 | Sadeh | 15.59 | 83 | p<.001 | p<.001 | 1.684e+23 | 0.40 |
| Precision | K2010 | UCSD | 14.75 | 83 | p<.001 | p<.001 | 5.938e+21 | 0.76 |
| Precision | K2010 | CK | 13.50 | 83 | p<.001 | p<.001 | 3.725e+19 | 0.54 |
| Precision | K2010 | Philips-20 | 7.97 | 83 | p<.001 | p<.001 | 1.134e+09 | 0.18 |
| Precision | K2010 | Philips-40 | 12.43 | 83 | p<.001 | p<.001 | 4.103e+17 | 0.38 |
| Precision | K2010 | Philips-80 | 14.51 | 83 | p<.001 | p<.001 | 2.313e+21 | 0.61 |
| Precision | K2010 | Sadeh | 14.84 | 83 | p<.001 | p<.001 | 8.636e+21 | 0.79 |
| Precision | UCSD | CK | -13.49 | 83 | p<.001 | p<.001 | 3.552e+19 | -0.22 |
| Precision | UCSD | Philips-20 | -14.05 | 83 | p<.001 | p<.001 | 3.553e+20 | -0.58 |
| Precision | UCSD | Philips-40 | -13.99 | 83 | p<.001 | p<.001 | 2.744e+20 | -0.38 |
| Precision | UCSD | Philips-80 | -10.46 | 83 | p<.001 | p<.001 | 7.686e+13 | -0.15 |
| Precision | UCSD | Sadeh | 3.43 | 83 | p<.001 | 0.020 | 24.84 | 0.03 |
| Precision | CK | Philips-20 | -11.42 | 83 | p<.001 | p<.001 | 5.112e+15 | -0.36 |
| Precision | CK | Philips-40 | -8.45 | 83 | p<.001 | p<.001 | 9.529e+09 | -0.16 |
| Precision | CK | Philips-80 | 6.52 | 83 | p<.001 | p<.001 | 2.167e+06 | 0.07 |
| Precision | CK | Sadeh | 13.26 | 83 | p<.001 | p<.001 | 1.328e+19 | 0.25 |
| Precision | Philips-20 | Philips-40 | 11.25 | 83 | p<.001 | p<.001 | 2.462e+15 | 0.20 |
| Precision | Philips-20 | Philips-80 | 13.57 | 83 | p<.001 | p<.001 | 4.942e+19 | 0.43 |
| Precision | Philips-20 | Sadeh | 14.25 | 83 | p<.001 | p<.001 | 7.979e+20 | 0.61 |
| Precision | Philips-40 | Philips-80 | 12.84 | 83 | p<.001 | p<.001 | 2.277e+18 | 0.23 |
| Precision | Philips-40 | Sadeh | 14.11 | 83 | p<.001 | p<.001 | 4.507e+20 | 0.41 |
| Precision | Philips-80 | Sadeh | 10.22 | 83 | p<.001 | p<.001 | 2.585e+13 | 0.18 |
| f1score | K2010 | UCSD | -4.32 | 83 | p<.001 | p<.001 | 425.86 | -0.40 |
| f1score | K2010 | CK | -5.92 | 83 | p<.001 | p<.001 | 1.826e+05 | -0.44 |
| f1score | K2010 | Philips-20 | -4.24 | 83 | p<.001 | .001 | 320.92 | -0.18 |
| f1score | K2010 | Philips-40 | -5.52 | 83 | p<.001 | p<.001 | 3.626e+04 | -0.36 |
| f1score | K2010 | Philips-80 | -4.85 | 83 | p<.001 | p<.001 | 2844.621 | -0.39 |
| f1score | K2010 | Sadeh | -4.47 | 83 | p<.001 | p<.001 | 722.059 | -0.42 |
| f1score | UCSD | CK | -1.54 | 83 | .128 | 1.000 | 0.37 | -0.04 |
| f1score | UCSD | Philips-20 | 3.46 | 83 | p<.001 | .018 | 27.587 | 0.25 |
| f1score | UCSD | Philips-40 | 1.43 | 83 | .158 | 1.000 | 0.32 | 0.06 |
| f1score | UCSD | Philips-80 | 0.59 | 83 | .554 | 1.000 | 0.14 | 0.01 |
| f1score | UCSD | Sadeh | -2.30 | 83 | .024 | .503 | 1.45 | -0.03 |
| f1score | CK | Philips-20 | 5.51 | 83 | p<.001 | p<.001 | 3.469e+04 | 0.29 |
| f1score | CK | Philips-40 | 3.91 | 83 | p<.001 | .004 | 110.78 | 0.11 |
| f1score | CK | Philips-80 | 3.50 | 83 | p<.001 | .016 | 30.73 | 0.05 |
| f1score | CK | Sadeh | 0.51 | 83 | .613 | 1.000 | 0.14 | 0.01 |
| f1score | Philips-20 | Philips-40 | -5.65 | 83 | p<.001 | p<.001 | 6.175e+04 | -0.19 |
| f1score | Philips-20 | Philips-80 | -4.19 | 83 | p<.001 | .001 | 277.62 | -0.23 |
| f1score | Philips-20 | Sadeh | -3.73 | 83 | p<.001 | .007 | 61.33 | -0.27 |
| f1score | Philips-40 | Philips-80 | -1.79 | 83 | .077 | 1.000 | 0.55 | -0.05 |
| f1score | Philips-40 | Sadeh | -1.90 | 83 | .061 | 1.000 | 0.67 | -0.09 |
| f1score | Philips-80 | Sadeh | -1.46 | 83 | .147 | 1.000 | 0.34 | -0.04 |

*^a. Contrasts between A and B. Bonferroni correction applied. BF10 represents Bayesian factor of 10 results.^*

**Table S13**

Repeated measures Anova for confusion matrix metrics for non-rescored algorithms RLS.^a^

| Metric | SS | MS | | *F* | *df* | η²g | Eps (ε) | *P_uncorr._* | df*_adjusted_* | *P_adjusted_* |
| --- | --- | --- | --- | --- | --- | --- | --- | --- | --- | --- |
| Accuracy_algorithm_ | 0.01 | | 0.001 | 1.71 | 6 | 0.002 | 0.20 | 0.117 | 1.17 | .195 |
| Accuracy_error_ | 0.31 | | 0.001 |  | 390 |  |  |  | 76.32 |  |
| Sensitivity_algorithm_ | 1.50 | | 0.25 | 187.25 | 6 | 0.29 | 0.20 | p<.001 | 1.18 | p<.001 |
| Sensitivity_error_ | 0.52 | | 0.001 |  | 390 |  |  |  | 76.58 |  |
| Specificity_algorithm_ | 4.03 | | 0.67 | 511.39 | 6 | 0.21 | 0.22 | p<.001 | 1.31 | p<.001 |
| Specificity_error_ | 0.51 | | 0.001 |  | 390 |  |  |  | 85.47 |  |
| Precision_algorithm_ | 0.32 | | 0.05 | 175.11 | 6 | 0.05 | 0.21 | p<.001 | 1.25 | p<.001 |
| Precision_error_ | 0.12 | | 0.0003 |  | 390 |  |  |  | 81.05 |  |
| f_1_-score_algorithm_ | 0.06 | | 0.01 | 17.24 | 6 | 0.01 | 0.20 | p<.001 | 1.17 | p<.001 |
| f_1_-score_error_ | 0.23 | | 0.001 |  | 390 |  |  |  | 76.14 |  |

*^a. Adjusted values for df and p values refer to respective Greenhouse Geiser corrections, SS = sum of squares MS = mean squares , and η²g = general eta squared.^*

**Table S14**

Post hoc analysis for confusion matrix metrics for non-rescored algorithms RLS.^a^

| Metric | A | B | *T* | *df* | *P_uncorr._* | *P_adjusted_* | BF10 | Hedges *g* | |
| --- | --- | --- | --- | --- | --- | --- | --- | --- | --- |
| Sensitivity | K2010 | UCSD | -14.20 | 65 | p<.001 | p<.001 | 3.339e+18 | -1.45 |  |
| Sensitivity | K2010 | CK | -14.72 | 65 | p<.001 | p<.001 | 1.958e+19 | -1.13 |  |
| Sensitivity | K2010 | Philips-20 | -5.99 | 65 | p<.001 | p<.001 | 1.366e+05 | -0.16 |  |
| Sensitivity | K2010 | Philips-40 | -13.39 | 65 | p<.001 | p<.001 | 2.092e+17 | -0.63 |  |
| Sensitivity | K2010 | Philips-80 | -14.53 | 65 | p<.001 | p<.001 | 1.038e+19 | -1.10 |  |
| Sensitivity | K2010 | Sadeh | -14.06 | 65 | p<.001 | p<.001 | 2.121e+18 | -1.61 |  |
| Sensitivity | UCSD | CK | 9.85 | 65 | p<.001 | p<.001 | 4.455e+11 | 0.37 |  |
| Sensitivity | UCSD | Philips-20 | 13.83 | 65 | p<.001 | p<.001 | 9.575e+17 | 1.36 |  |
| Sensitivity | UCSD | Philips-40 | 13.09 | 65 | p<.001 | p<.001 | 7.208e+16 | 0.95 |  |
| Sensitivity | UCSD | Philips-80 | 11.22 | 65 | p<.001 | p<.001 | 7.955e+13 | 0.44 |  |
| Sensitivity | UCSD | Sadeh | -9.60 | 65 | p<.001 | p<.001 | 1.672e+11 | -0.23 |  |
| Sensitivity | CK | Philips-20 | 13.68 | 65 | p<.001 | p<.001 | 5.714e+17 | 1.01 |  |
| Sensitivity | CK | Philips-40 | 11.84 | 65 | p<.001 | p<.001 | 8.153e+14 | 0.57 |  |
| Sensitivity | CK | Philips-80 | 2.41 | 65 | .019 | .398 | 1.95 | 0.05 |  |
| Sensitivity | CK | Sadeh | -10.84 | 65 | p<.001 | p<.001 | 1.953e+13 | -0.58 |  |
| Sensitivity | Philips-20 | Philips-40 | -13.80 | 65 | p<.001 | p<.001 | 8.511e+17 | -0.49 |  |
| Sensitivity | Philips-20 | Philips-80 | -14.19 | 65 | p<.001 | p<.001 | 3.295e+18 | -0.98 |  |
| Sensitivity | Philips-20 | Sadeh | -13.78 | 65 | p<.001 | p<.001 | 8.044e+17 | -1.53 |  |
| Sensitivity | Philips-40 | Philips-80 | -13.20 | 65 | p<.001 | p<.001 | 1.055e+17 | -0.53 |  |
| Sensitivity | Philips-40 | Sadeh | -13.11 | 65 | p<.001 | p<.001 | 7.759e+16 | -1.15 |  |
| Sensitivity | Philips-80 | Sadeh | -11.72 | 65 | p<.001 | p<.001 | 5.116e+14 | -0.66 |  |
| Specificity | K2010 | UCSD | 25.11 | 65 | p<.001 | p<.001 | 6.557e+31 | 1.37 |  |
| Specificity | K2010 | CK | 23.45 | 65 | p<.001 | p<.001 | 1.286e+30 | 0.93 |  |
| Specificity | K2010 | Philips-20 | 12.28 | 65 | p<.001 | p<.001 | 3.963e+15 | 0.23 |  |
| Specificity | K2010 | Philips-40 | 21.23 | 65 | p<.001 | p<.001 | 4.607e+27 | 0.57 |  |
| Specificity | K2010 | Philips-80 | 22.55 | 65 | p<.001 | p<.001 | 1.369e+29 | 0.97 |  |
| Specificity | K2010 | Sadeh | 23.49 | 65 | p<.001 | p<.001 | 1.401e+30 | 1.43 |  |
| Specificity | UCSD | CK | -21.07 | 65 | p<.001 | p<.001 | 3.022e+27 | -0.42 |  |
| Specificity | UCSD | Philips-20 | -24.85 | 65 | p<.001 | p<.001 | 3.55e+31 | -1.17 |  |
| Specificity | UCSD | Philips-40 | -23.79 | 65 | p<.001 | p<.001 | 2.911e+30 | -0.81 |  |
| Specificity | UCSD | Philips-80 | -21.77 | 65 | p<.001 | p<.001 | 1.9e+28 | -0.40 |  |
| Specificity | UCSD | Sadeh | 6.27 | 65 | p<.001 | p<.001 | 3.832e+05 | 0.08 |  |
| Specificity | CK | Philips-20 | -21.72 | 65 | p<.001 | p<.001 | 1.672e+28 | -0.72 |  |
| Specificity | CK | Philips-40 | -18.12 | 65 | p<.001 | p<.001 | 8.191e+23 | -0.37 |  |
| Specificity | CK | Philips-80 | 2.22 | 65 | .030 | .635 | 1.32 | 0.03 |  |
| Specificity | CK | Sadeh | 19.31 | 65 | p<.001 | p<.001 | 2.54e+25 | 0.49 |  |
| Specificity | Philips-20 | Philips-40 | 20.22 | 65 | p<.001 | p<.001 | 3.146e+26 | 0.35 |  |
| Specificity | Philips-20 | Philips-80 | 21.58 | 65 | p<.001 | p<.001 | 1.156e+28 | 0.76 |  |
| Specificity | Philips-20 | Sadeh | 23.04 | 65 | p<.001 | p<.001 | 4.702e+29 | 1.22 |  |
| Specificity | Philips-40 | Philips-80 | 18.33 | 65 | p<.001 | p<.001 | 1.529e+24 | 0.40 |  |
| Specificity | Philips-40 | Sadeh | 21.48 | 65 | p<.001 | p<.001 | 8.96e+27 | 0.87 |  |
| Specificity | Philips-80 | Sadeh | 19.77 | 65 | p<.001 | p<.001 | 9.048e+25 | 0.47 |  |
| Precision | K2010 | UCSD | 14.04 | 65 | p<.001 | p<.001 | 1.989e+18 | 0.60 |  |
| Precision | K2010 | CK | 13.03 | 65 | p<.001 | p<.001 | 5.81e+16 | 0.41 |  |
| Precision | K2010 | Philips-20 | 8.17 | 65 | p<.001 | p<.001 | 6.232e+08 | 0.14 |  |
| Precision | K2010 | Philips-40 | 11.36 | 65 | p<.001 | p<.001 | 1.373e+14 | 0.28 |  |
| Precision | K2010 | Philips-80 | 12.87 | 65 | p<.001 | p<.001 | 3.302e+16 | 0.44 |  |
| Precision | K2010 | Sadeh | 13.46 | 65 | p<.001 | p<.001 | 2.678e+17 | 0.61 |  |
| Precision | UCSD | CK | -13.67 | 65 | p<.001 | p<.001 | 5.487e+17 | -0.19 |  |
| Precision | UCSD | Philips-20 | -14.45 | 65 | p<.001 | p<.001 | 7.977e+18 | -0.47 |  |
| Precision | UCSD | Philips-40 | -14.39 | 65 | p<.001 | p<.001 | 6.479e+18 | -0.33 |  |
| Precision | UCSD | Philips-80 | -13.77 | 65 | p<.001 | p<.001 | 7.885e+17 | -0.17 |  |
| Precision | UCSD | Sadeh | 1.96 | 65 | .054 | 1.00 | 0.82 | 0.01 |  |
| Precision | CK | Philips-20 | -12.92 | 65 | p<.001 | p<.001 | 3.991e+16 | -0.28 |  |
| Precision | CK | Philips-40 | -11.07 | 65 | p<.001 | p<.001 | 4.555e+13 | -0.14 |  |
| Precision | CK | Philips-80 | 2.78 | 65 | .007 | .147 | 4.60 | 0.02 |  |
| Precision | CK | Sadeh | 12.52 | 65 | p<.001 | p<.001 | 9.504e+15 | 0.20 |  |
| Precision | Philips-20 | Philips-40 | 11.42 | 65 | p<.001 | p<.001 | 1.692e+14 | 0.14 |  |
| Precision | Philips-20 | Philips-80 | 13.12 | 65 | p<.001 | p<.001 | 8.169e+16 | 0.30 |  |
| Precision | Philips-20 | Sadeh | 13.71 | 65 | p<.001 | p<.001 | 6.395e+17 | 0.48 |  |
| Precision | Philips-40 | Philips-80 | 12.04 | 65 | p<.001 | p<.001 | 1.652e+15 | 0.16 |  |
| Precision | Philips-40 | Sadeh | 13.44 | 65 | p<.001 | p<.001 | 2.464e+17 | 0.34 |  |
| Precision | Philips-80 | Sadeh | 12.58 | 65 | p<.001 | p<.001 | 1.201e+16 | 0.18 |  |
| f1score | K2010 | UCSD | -3.60 | 65 | p<.001 | .013 | 40.61 | -0.26 |  |
| f1score | K2010 | CK | -4.93 | 65 | p<.001 | p<.001 | 2893.93 | -0.29 |  |
| f1score | K2010 | Philips-20 | -0.91 | 65 | .365 | 1.000 | 0.20 | -0.02 |  |
| f1score | K2010 | Philips-40 | -4.38 | 65 | p<.001 | .001 | 451.56 | -0.18 |  |
| f1score | K2010 | Philips-80 | -4.39 | 65 | p<.001 | .001 | 466.22 | -0.26 |  |
| f1score | K2010 | Sadeh | -3.90 | 65 | p<.001 | .005 | 97.51 | -0.30 |  |
| f1score | UCSD | CK | -1.93 | 65 | .058 | 1.000 | 0.77 | -0.03 |  |
| f1score | UCSD | Philips-20 | 3.98 | 65 | p<.001 | .004 | 125.05 | 0.24 |  |
| f1score | UCSD | Philips-40 | 2.24 | 65 | .029 | .599 | 1.38 | 0.09 |  |
| f1score | UCSD | Philips-80 | 0.03 | 65 | .977 | 1.000 | 0.14 | 0.00 |  |
| f1score | UCSD | Sadeh | -4.90 | 65 | p<.001 | p<.001 | 2576.16 | -0.05 |  |
| f1score | CK | Philips-20 | 5.55 | 65 | p<.001 | p<.001 | 2.632e+04 | 0.27 |  |
| f1score | CK | Philips-40 | 4.39 | 65 | p<.001 | .001 | 458.91 | 0.12 |  |
| f1score | CK | Philips-80 | 2.98 | 65 | .004 | .086 | 7.41 | 0.04 |  |
| f1score | CK | Sadeh | -0.56 | 65 | .578 | 1.000 | 0.16 | -0.01 |  |
| f1score | Philips-20 | Philips-40 | -6.00 | 65 | p<.001 | p<.001 | 1.387e+05 | -0.16 |  |
| f1score | Philips-20 | Philips-80 | -5.14 | 65 | p<.001 | p<.001 | 5968.16 | -0.24 |  |
| f1score | Philips-20 | Sadeh | -4.29 | 65 | p<.001 | .001 | 336.55 | -0.29 |  |
| f1score | Philips-40 | Philips-80 | -3.56 | 65 | p<.001 | .015 | 35.49 | -0.09 |  |
| f1score | Philips-40 | Sadeh | -2.99 | 65 | .004 | .082 | 7.68 | -0.14 |  |
| f1score | Philips-80 | Sadeh | -2.10 | 65 | .040 | .832 | 1.05 | -0.05 |  |

*^a. Contrasts between A and B. Bonferroni correction applied. BF10 represents Bayesian factor of 10 results.^*

**Table S15**

Repeated measures Anova for confusion matrix metrics for rescored algorithms RLS.^a^

| Metric | SS | MS | *F* | *df* | η²g | Eps (ε) | *P_uncorr._* | df*_adjusted_* | *P_adjusted_* |
| --- | --- | --- | --- | --- | --- | --- | --- | --- | --- |
| Accuracy_algorithm_ | 0.04 | 0.007 | 5.71 | 6 | 0.01 | 0.21 | p<.001 | 1.24 | .014 |
| Accuracy_error_ | 0.48 | 0.001 |  | 390 |  |  |  | 80.71 |  |
| Sensitivity_algorithm_ | 2.23 | 0.37 | 192.11 | 6 | 0.32 | 0.21 | p<.001 | 1.25 | p<.001 |
| Sensitivity_error_ | 0.75 | 0.002 |  | 390 |  |  |  | 80.95 |  |
| Specificity_algorithm_ | 4.11 | 0.68 | 360.04 | 6 | 0.20 | 0.28 | p<.001 | 1.67 | p<.001 |
| Specificity_error_ | 0.74 | 0.002 |  | 390 |  |  |  | 108.78 |  |
| Precision_algorithm_ | 0.38 | 0.06 | 134.79 | 6 | 0.05 | 0.25 | p<.001 | 1.50 | p<.001 |
| Precision_error_ | 0.18 | 0.0005 |  | 390 |  |  |  | 97.44 |  |
| f_1_-score_algorithm_ | 0.18 | 0.03 | 27.19 | 6 | 0.04 | 0.20 | p<.001 | 1.21 | p<.001 |
| f_1_-score_error_ | 0.43 | 0.001 |  | 390 |  |  |  | 78.41 |  |

*^a. Adjusted values for df and p values refer to respective Greenhouse Geiser corrections, SS = sum of squares MS = mean squares , and η²g = general eta squared.^*

**Table S16**

Post hoc analysis for confusion matrix metrics for rescored algorithms RLS.^a^

| Metric | A | B | *T* | *df* | *P_uncorr._* | *P_adjusted_* | BF10 | Hedges *g* |
| --- | --- | --- | --- | --- | --- | --- | --- | --- |
| Accuracy | K2010 | UCSD | -1.93 | 65 | .058 | 1.000 | 0.77 | -0.19 |
| Accuracy | K2010 | CK | -3.69 | 65 | p<.001 | .010 | 51.72 | -0.29 |
| Accuracy | K2010 | Philips-20 | -2.90 | 65 | .005 | .108 | 6.03 | -0.09 |
| Accuracy | K2010 | Philips-40 | -3.46 | 65 | .001 | .020 | 26.94 | -0.20 |
| Accuracy | K2010 | Philips-80 | -3.09 | 65 | .003 | .062 | 9.91 | -0.25 |
| Accuracy | K2010 | Sadeh | -2.21 | 65 | .031 | .644 | 1.30 | -0.23 |
| Accuracy | UCSD | CK | -3.28 | 65 | .002 | .035 | 16.20 | -0.10 |
| Accuracy | UCSD | Philips-20 | 1.23 | 65 | .223 | 1.000 | 0.28 | 0.10 |
| Accuracy | UCSD | Philips-40 | -0.10 | 65 | .922 | 1.000 | 0.14 | -0.01 |
| Accuracy | UCSD | Philips-80 | -2.06 | 65 | .043 | .908 | 0.98 | -0.05 |
| Accuracy | UCSD | Sadeh | -2.50 | 65 | .015 | .312 | 2.40 | -0.04 |
| Accuracy | CK | Philips-20 | 3.18 | 65 | .002 | .047 | 12.54 | 0.21 |
| Accuracy | CK | Philips-40 | 2.83 | 65 | .006 | .130 | 5.14 | 0.10 |
| Accuracy | CK | Philips-80 | 2.57 | 65 | .012 | .262 | 2.79 | 0.05 |
| Accuracy | CK | Sadeh | 1.63 | 65 | .107 | 1.000 | 0.48 | 0.06 |
| Accuracy | Philips-20 | Philips-40 | -2.94 | 65 | .005 | .095 | 6.78 | -0.11 |
| Accuracy | Philips-20 | Philips-80 | -2.53 | 65 | .014 | .292 | 2.54 | -0.16 |
| Accuracy | Philips-20 | Sadeh | -1.60 | 65 | .114 | 1.000 | 0.45 | -0.14 |
| Accuracy | Philips-40 | Philips-80 | -1.57 | 65 | .120 | 1.000 | 0.44 | -0.05 |
| Accuracy | Philips-40 | Sadeh | -0.62 | 65 | .540 | 1.000 | 0.16 | -0.04 |
| Accuracy | Philips-80 | Sadeh | 0.40 | 65 | .689 | 1.000 | 0.15 | 0.01 |
| Sensitivity | K2010 | UCSD | -14.80 | 65 | p<.001 | p<.001 | 2.558e+19 | -1.63 |
| Sensitivity | K2010 | CK | -15.26 | 65 | p<.001 | p<.001 | 1.137e+20 | -1.22 |
| Sensitivity | K2010 | Philips-20 | -9.37 | 65 | p<.001 | p<.001 | 6.945e+10 | -0.29 |
| Sensitivity | K2010 | Philips-40 | -13.97 | 65 | p<.001 | p<.001 | 1.55e+18 | -0.80 |
| Sensitivity | K2010 | Philips-80 | -15.07 | 65 | p<.001 | p<.001 | 6.28e+19 | -1.26 |
| Sensitivity | K2010 | Sadeh | -14.58 | 65 | p<.001 | p<.001 | 1.2e+19 | -1.73 |
| Sensitivity | UCSD | CK | 9.82 | 65 | p<.001 | p<.001 | 3.965e+11 | 0.46 |
| Sensitivity | UCSD | Philips-20 | 13.78 | 65 | p<.001 | p<.001 | 7.956e+17 | 1.41 |
| Sensitivity | UCSD | Philips-40 | 13.26 | 65 | p<.001 | p<.001 | 1.324e+17 | 0.99 |
| Sensitivity | UCSD | Philips-80 | 10.07 | 65 | p<.001 | p<.001 | 1.038e+12 | 0.45 |
| Sensitivity | UCSD | Sadeh | -6.27 | 65 | p<.001 | p<.001 | 3.905e+05 | -0.17 |
| Sensitivity | CK | Philips-20 | 13.41 | 65 | p<.001 | p<.001 | 2.214e+17 | 0.97 |
| Sensitivity | CK | Philips-40 | 11.05 | 65 | p<.001 | p<.001 | 4.246e+13 | 0.50 |
| Sensitivity | CK | Philips-80 | -0.84 | 65 | .404 | 1.000 | 0.19 | -0.02 |
| Sensitivity | CK | Sadeh | -10.39 | 65 | p<.001 | p<.001 | 3.477e+12 | -0.60 |
| Sensitivity | Philips-20 | Philips-40 | -13.05 | 65 | p<.001 | p<.001 | 6.369e+16 | -0.52 |
| Sensitivity | Philips-20 | Philips-80 | -13.90 | 65 | p<.001 | p<.001 | 1.22e+18 | -1.01 |
| Sensitivity | Philips-20 | Sadeh | -13.58 | 65 | p<.001 | p<.001 | 4.055e+17 | -1.52 |
| Sensitivity | Philips-40 | Philips-80 | -12.54 | 65 | p<.001 | p<.001 | 1.029e+16 | -0.53 |
| Sensitivity | Philips-40 | Sadeh | -12.97 | 65 | p<.001 | p<.001 | 4.717e+16 | -1.12 |
| Sensitivity | Philips-80 | Sadeh | -10.24 | 65 | p<.001 | p<.001 | 1.972e+12 | -0.60 |
| Specificity | K2010 | UCSD | 22.74 | 65 | p<.001 | p<.001 | 2.2e+29 | 1.37 |
| Specificity | K2010 | CK | 19.09 | 65 | p<.001 | p<.001 | 1.36e+25 | 0.90 |
| Specificity | K2010 | Philips-20 | 9.24 | 65 | p<.001 | p<.001 | 4.11e+10 | 0.24 |
| Specificity | K2010 | Philips-40 | 17.34 | 65 | p<.001 | p<.001 | 7.962e+22 | 0.62 |
| Specificity | K2010 | Philips-80 | 19.88 | 65 | p<.001 | p<.001 | 1.222e+26 | 0.99 |
| Specificity | K2010 | Sadeh | 21.06 | 65 | p<.001 | p<.001 | 3.007e+27 | 1.39 |
| Specificity | UCSD | CK | -16.47 | 65 | p<.001 | p<.001 | 5.506e+21 | -0.44 |
| Specificity | UCSD | Philips-20 | -21.89 | 65 | p<.001 | p<.001 | 2.58e+28 | -1.13 |
| Specificity | UCSD | Philips-40 | -19.49 | 65 | p<.001 | p<.001 | 4.139e+25 | -0.74 |
| Specificity | UCSD | Philips-80 | -18.67 | 65 | p<.001 | p<.001 | 4.105e+24 | -0.37 |
| Specificity | UCSD | Sadeh | 2.74 | 65 | .008 | .167 | 4.12 | 0.05 |
| Specificity | CK | Philips-20 | -17.52 | 65 | p<.001 | p<.001 | 1.389e+23 | -0.66 |
| Specificity | CK | Philips-40 | -12.12 | 65 | p<.001 | p<.001 | 2.279e+15 | -0.29 |
| Specificity | CK | Philips-80 | 4.07 | 65 | p<.001 | .003 | 166.445 | 0.08 |
| Specificity | CK | Sadeh | 14.24 | 65 | p<.001 | p<.001 | 3.869e+18 | 0.48 |
| Specificity | Philips-20 | Philips-40 | 16.29 | 65 | p<.001 | p<.001 | 3.172e+21 | 0.38 |
| Specificity | Philips-20 | Philips-80 | 18.09 | 65 | p<.001 | p<.001 | 7.526e+23 | 0.75 |
| Specificity | Philips-20 | Sadeh | 19.86 | 65 | p<.001 | p<.001 | 1.17e+26 | 1.16 |
| Specificity | Philips-40 | Philips-80 | 12.95 | 65 | p<.001 | p<.001 | 4.501e+16 | 0.37 |
| Specificity | Philips-40 | Sadeh | 16.94 | 65 | p<.001 | p<.001 | 2.365e+22 | 0.78 |
| Specificity | Philips-80 | Sadeh | 15.37 | 65 | p<.001 | p<.001 | 1.667e+20 | 0.41 |
| Precision | K2010 | UCSD | 12.77 | 65 | p<.001 | p<.001 | 2.32e+16 | 0.64 |
| Precision | K2010 | CK | 11.51 | 65 | p<.001 | p<.001 | 2.425e+14 | 0.42 |
| Precision | K2010 | Philips-20 | 5.84 | 65 | p<.001 | p<.001 | 7.692e+04 | 0.13 |
| Precision | K2010 | Philips-40 | 10.20 | 65 | p<.001 | p<.001 | 1.717e+12 | 0.31 |
| Precision | K2010 | Philips-80 | 12.00 | 65 | p<.001 | p<.001 | 1.444e+15 | 0.47 |
| Precision | K2010 | Sadeh | 12.21 | 65 | p<.001 | p<.001 | 3.113e+15 | 0.64 |
| Precision | UCSD | CK | -10.68 | 65 | p<.001 | p<.001 | 1.066e+13 | -0.22 |
| Precision | UCSD | Philips-20 | -13.11 | 65 | p<.001 | p<.001 | 7.784e+16 | -0.52 |
| Precision | UCSD | Philips-40 | -12.34 | 65 | p<.001 | p<.001 | 5.043e+15 | -0.34 |
| Precision | UCSD | Philips-80 | -10.57 | 65 | p<.001 | p<.001 | 6.999e+12 | -0.17 |
| Precision | UCSD | Sadeh | 0.46 | 65 | .644 | 1.000 | 0.15 | 0.01 |
| Precision | CK | Philips-20 | -11.24 | 65 | p<.001 | p<.001 | 8.773e+13 | -0.30 |
| Precision | CK | Philips-40 | -7.56 | 65 | p<.001 | p<.001 | 5.644e+07 | -0.12 |
| Precision | CK | Philips-80 | 3.87 | 65 | p<.001 | .005 | 90.60 | 0.05 |
| Precision | CK | Sadeh | 9.47 | 65 | p<.001 | p<.001 | 1.025e+11 | 0.22 |
| Precision | Philips-20 | Philips-40 | 10.30 | 65 | p<.001 | p<.001 | 2.53e+12 | 0.18 |
| Precision | Philips-20 | Philips-80 | 11.73 | 65 | p<.001 | p<.001 | 5.355e+14 | 0.35 |
| Precision | Philips-20 | Sadeh | 12.39 | 65 | p<.001 | p<.001 | 6.026e+15 | 0.53 |
| Precision | Philips-40 | Philips-80 | 9.17 | 65 | p<.001 | p<.001 | 3.115e+10 | 0.17 |
| Precision | Philips-40 | Sadeh | 11.20 | 65 | p<.001 | p<.001 | 7.462e+13 | 0.34 |
| Precision | Philips-80 | Sadeh | 8.76 | 65 | p<.001 | p<.001 | 6.395e+09 | 0.18 |
| f1score | K2010 | UCSD | -5.17 | 65 | p<.001 | p<.001 | 6710.03 | -0.49 |
| f1score | K2010 | CK | -6.34 | 65 | p<.001 | p<.001 | 4.974e+05 | -0.48 |
| f1score | K2010 | Philips-20 | -4.70 | 65 | p<.001 | p<.001 | 1321.42 | -0.15 |
| f1score | K2010 | Philips-40 | -5.83 | 65 | p<.001 | p<.001 | 7.514e+04 | -0.35 |
| f1score | K2010 | Philips-80 | -5.84 | 65 | p<.001 | p<.001 | 7.619e+04 | -0.46 |
| f1score | K2010 | Sadeh | -5.35 | 65 | p<.001 | p<.001 | 1.291e+04 | -0.53 |
| f1score | UCSD | CK | -0.02 | 65 | .983 | 1.000 | 0.14 | 0.00 |
| f1score | UCSD | Philips-20 | 4.60 | 65 | p<.001 | p<.001 | 933.33 | 0.36 |
| f1score | UCSD | Philips-40 | 3.38 | 65 | .001 | .026 | 21.36 | 0.16 |
| f1score | UCSD | Philips-80 | 1.12 | 65 | .269 | 1.000 | 0.24 | 0.03 |
| f1score | UCSD | Sadeh | -3.21 | 65 | .002 | .043 | 13.67 | -0.05 |
| f1score | CK | Philips-20 | 5.84 | 65 | p<.001 | p<.001 | 7.643e+04 | 0.35 |
| f1score | CK | Philips-40 | 5.08 | 65 | p<.001 | p<.001 | 4848.30 | 0.15 |
| f1score | CK | Philips-80 | 1.69 | 65 | .095 | 1.000 | 0.52 | 0.03 |
| f1score | CK | Sadeh | -1.38 | 65 | .172 | 1.000 | 0.33 | -0.04 |
| f1score | Philips-20 | Philips-40 | -5.50 | 65 | p<.001 | p<.001 | 2.206e+04 | -0.21 |
| f1score | Philips-20 | Philips-80 | -5.39 | 65 | p<.001 | p<.001 | 1.465e+04 | -0.33 |
| f1score | Philips-20 | Sadeh | -4.83 | 65 | p<.001 | p<.001 | 2043.08 | -0.40 |
| f1score | Philips-40 | Philips-80 | -4.34 | 65 | p<.001 | .001 | 397.23 | -0.13 |
| f1score | Philips-40 | Sadeh | -3.89 | 65 | p<.001 | .005 | 96.47 | -0.20 |
| f1score | Philips-80 | Sadeh | -2.39 | 65 | .020 | .420 | 1.86 | -0.07 |

*^a. Contrasts between A and B. Bonferroni correction applied. BF10 represents Bayesian factor of 10 results.^*
