# Supplementary material for: Comparison and Validation of Actigraphy Algorithms Using a Large Community Dataset: Algorithm Validation Study
Source: JMIR Form Res. 2025 Dec 11;9:e70778. doi: 10.2196/70778 (PMC12697920; doi:10.2196/70778)
Supplement: Multimedia Appendix 10 [file formative-v9-e70778-s010.docx]

Multimedia Appendix J: Sleep Problem Subgroup MCC and Kappa Repeated Measures Anova and Post Hoc:

**Table S1**

MCC repeated measures ANOVA for non-rescored and rescored algorithms - Apnea^a^

| Source | Rescore | SS | MS | *F* | η²g | Eps (ε) | *df* | *P_uncorr._* | df*_adjusted_* | *P_adjusted_* |
| --- | --- | --- | --- | --- | --- | --- | --- | --- | --- | --- |
| MCC_algorithm_ | NRS | 0.08 | 0.01 | 4.51 | 0.003 | 0.22 | 6 | p<.001 | 1.31 | .026 |
| MCC_error_ | NRS | 1.80 | 0.003 |  |  |  | 618 |  | 135.07 |  |
| MCC_algorithm_ | RS | 0.05 | 0.01 | 2.27 | 0.002 | 0.23 | 6 | .036 | 1.41 | .124 |
| MCC_error_ | RS | 2.45 | 0.004 |  |  |  | 618 |  | 144.74 |  |

*^a. Adjusted values for df and p values refer to respective Greenhouse Geiser corrections, SS = sum of squares MS = mean squares , and η²g = general eta squared.^*

**Table S2**

MCC post hoc results for non-rescored algorithms - Apnea^a^

| **A** | **B** | ***T*** | ***df*** | ***P_uncorr._*** | ***P_adjusted_*** | **BF10** | **Hedges *g*** |
| --- | --- | --- | --- | --- | --- | --- | --- |
| K2010-nrs | UCSD-nrs | 2.71 | 103 | .008 | .166 | 3.45 | 0.16 |
| K2010-nrs | CK-nrs | 0.39 | 103 | .694 | 1.000 | 0.12 | 0.02 |
| K2010-nrs | Philips-20-nrs | 4.47 | 103 | p<.001 | p<.001 | 801.24 | 0.08 |
| K2010-nrs | Philips-40-nrs | 2.50 | 103 | .014 | .296 | 2.08 | 0.06 |
| K2010-nrs | Philips-80-nrs | 2.66 | 103 | .009 | .189 | 3.08 | 0.11 |
| K2010-nrs | Sadeh-nrs | 1.93 | 103 | .056 | 1.000 | 0.65 | 0.13 |
| UCSD-nrs | CK-nrs | -7.56 | 103 | p<.001 | p<.001 | 4.96E+08 | -0.14 |
| UCSD-nrs | Philips-20-nrs | -1.40 | 103 | .164 | 1.000 | 0.28 | -0.08 |
| UCSD-nrs | Philips-40-nrs | -2.36 | 103 | .020 | .418 | 1.55 | -0.10 |
| UCSD-nrs | Philips-80-nrs | -2.14 | 103 | .035 | .737 | 0.96 | -0.05 |
| UCSD-nrs | Sadeh-nrs | -2.09 | 103 | .039 | .817 | 0.88 | -0.03 |
| CK-nrs | Philips-20-nrs | 1.55 | 103 | .123 | 1.000 | 0.35 | 0.07 |
| CK-nrs | Philips-40-nrs | 1.70 | 103 | .092 | 1.000 | 0.44 | 0.05 |
| CK-nrs | Philips-80-nrs | 6.72 | 103 | p<.001 | p<.001 | 9.48E+06 | 0.09 |
| CK-nrs | Sadeh-nrs | 3.95 | 103 | p<.001 | .003 | 128.42 | 0.11 |
| Philips-20-nrs | Philips-40-nrs | -0.96 | 103 | .337 | 1.000 | 0.17 | -0.02 |
| Philips-20-nrs | Philips-80-nrs | 0.69 | 103 | .495 | 1.000 | 0.14 | 0.02 |
| Philips-20-nrs | Sadeh-nrs | 0.74 | 103 | .460 | 1.000 | 0.14 | 0.05 |
| Philips-40-nrs | Philips-80-nrs | 2.16 | 103 | .033 | .702 | 1.00 | 0.04 |
| Philips-40-nrs | Sadeh-nrs | 1.34 | 103 | .184 | 1.000 | 0.26 | 0.06 |
| Philips-80-nrs | Sadeh-nrs | 0.64 | 103 | .522 | 1.000 | 0.13 | 0.02 |

*^a. Contrasts between A and B. Bonferroni correction applied. BF10 represents Bayesian factor of 10 results.^*

**Table S3**

Cohens Kappa repeated measures ANOVA for non-rescored and rescored algorithms - Apnea^a^

| Source | Rescore | SS | MS | *F* | η²g | Eps (ε) | *df* | *P_uncorr._* | df*_adjusted_* | *P_adjusted_* |
| --- | --- | --- | --- | --- | --- | --- | --- | --- | --- | --- |
| K_algorithm_ | NRS | 0.43 | 0.07 | 17.28 | 0.01 | 0.21 | 6 | p<.001 | 1.24 | <0.001 |
| K_Error_ | NRS | 2.57 | 0.004 |  |  |  | 618 |  | 127.66 |  |
| K_algorithm_ | RS | 0.16 | 0.03 | 4.63 | 0.01 | 0.22 | 6 | p<.001 | 1.31 | .024 |
| K_Error_ | RS | 3.61 | 0.005 |  |  |  | 618 |  | 134.72 |  |

*^a. Adjusted values for df and p values refer to respective Greenhouse Geiser corrections, SS = sum of squares MS = mean squares , and η²g = general eta squared.^*

**Table S4**

Cohens Kappa post hoc results for non-rescored and rescored algorithms - Apnea^a^

| **A** | **B** | ***T*** | ***df*** | ***P_uncorr._*** | ***P_adjusted_*** | **BF10** | **Hedges *g*** |
| --- | --- | --- | --- | --- | --- | --- | --- |
| K2010-nrs | UCSD-nrs | 4.60 | 103 | p<.001 | p<.001 | 1302.68 | 0.31 |
| K2010-nrs | CK-nrs | 1.74 | 103 | .085 | 1.000 | 0.47 | 0.10 |
| K2010-nrs | Philips-20-nrs | 3.89 | 103 | p<.001 | .004 | 106.24 | 0.08 |
| K2010-nrs | Philips-40-nrs | 2.37 | 103 | .020 | .413 | 1.56 | 0.07 |
| K2010-nrs | Philips-80-nrs | 3.47 | 103 | .001 | .016 | 27.90 | 0.17 |
| K2010-nrs | Sadeh-nrs | 4.35 | 103 | p<.001 | .001 | 523.89 | 0.33 |
| UCSD-nrs | CK-nrs | -11.02 | 103 | p<.001 | p<.001 | 1.29E+16 | -0.22 |
| UCSD-nrs | Philips-20-nrs | -3.93 | 103 | p<.001 | .003 | 122.99 | -0.25 |
| UCSD-nrs | Philips-40-nrs | -5.51 | 103 | p<.001 | p<.001 | 47950.00 | -0.24 |
| UCSD-nrs | Philips-80-nrs | -5.84 | 103 | p<.001 | p<.001 | 194100.00 | -0.14 |
| UCSD-nrs | Sadeh-nrs | 1.47 | 103 | .143 | 1.000 | 0.31 | 0.02 |
| CK-nrs | Philips-20-nrs | -0.45 | 103 | .655 | 1.000 | 0.12 | -0.02 |
| CK-nrs | Philips-40-nrs | -0.72 | 103 | .471 | 1.000 | 0.14 | -0.02 |
| CK-nrs | Philips-80-nrs | 6.44 | 103 | p<.001 | p<.001 | 2.68E+06 | 0.08 |
| CK-nrs | Sadeh-nrs | 7.89 | 103 | p<.001 | p<.001 | 2.44E+09 | 0.24 |
| Philips-20-nrs | Philips-40-nrs | 0.02 | 103 | .987 | 1.000 | 0.11 | 0.00 |
| Philips-20-nrs | Philips-80-nrs | 2.33 | 103 | .022 | .455 | 1.44 | 0.10 |
| Philips-20-nrs | Sadeh-nrs | 3.76 | 103 | p<.001 | .006 | 70.25 | 0.26 |
| Philips-40-nrs | Philips-80-nrs | 4.29 | 103 | p<.001 | .001 | 426.98 | 0.10 |
| Philips-40-nrs | Sadeh-nrs | 4.93 | 103 | p<.001 | p<.001 | 4546.59 | 0.26 |
| Philips-80-nrs | Sadeh-nrs | 4.71 | 103 | p<.001 | p<.001 | 2001.34 | 0.16 |
| K2010-rs | UCSD-rs | 0.75 | 103 | .455 | 1.000 | 0.14 | 0.06 |
| K2010-rs | CK-rs | -1.98 | 103 | .050 | 1.000 | 0.71 | -0.13 |
| K2010-rs | Philips-20-rs | -2.32 | 103 | .022 | .472 | 1.39 | -0.05 |
| K2010-rs | Philips-40-rs | -2.75 | 103 | .007 | .149 | 3.79 | -0.11 |
| K2010-rs | Philips-80-rs | -0.87 | 103 | .385 | 1.000 | 0.16 | -0.05 |
| K2010-rs | Sadeh-rs | 0.76 | 103 | .451 | 1.000 | 0.14 | 0.06 |
| UCSD-rs | CK-rs | -6.63 | 103 | p<.001 | p<.001 | 6.45E+06 | -0.18 |
| UCSD-rs | Philips-20-rs | -1.61 | 103 | .110 | 1.000 | 0.38 | -0.11 |
| UCSD-rs | Philips-40-rs | -3.46 | 103 | .001 | .016 | 27.31 | -0.17 |
| UCSD-rs | Philips-80-rs | -3.98 | 103 | p<.001 | .003 | 144.18 | -0.11 |
| UCSD-rs | Sadeh-rs | 0.40 | 103 | .692 | 1.000 | 0.12 | 0.01 |
| CK-rs | Philips-20-rs | 1.36 | 103 | .177 | 1.000 | 0.27 | 0.07 |
| CK-rs | Philips-40-rs | 0.41 | 103 | .686 | 1.000 | 0.12 | 0.01 |
| CK-rs | Philips-80-rs | 4.62 | 103 | p<.001 | p<.001 | 1415.71 | 0.07 |
| CK-rs | Sadeh-rs | 5.25 | 103 | p<.001 | p<.001 | 1.62E+04 | 0.19 |
| Philips-20-rs | Philips-40-rs | -2.10 | 103 | .038 | .798 | 0.90 | -0.06 |
| Philips-20-rs | Philips-80-rs | 0.00 | 103 | .998 | 1.000 | 0.11 | 0.00 |
| Philips-20-rs | Sadeh-rs | 1.54 | 103 | .126 | 1.000 | 0.34 | 0.12 |
| Philips-40-rs | Philips-80-rs | 2.24 | 103 | .027 | .565 | 1.20 | 0.06 |
| Philips-40-rs | Sadeh-rs | 3.09 | 103 | .003 | .055 | 9.24 | 0.18 |
| Philips-80-rs | Sadeh-rs | 3.21 | 103 | .002 | .037 | 13.09 | 0.12 |

*^a. Contrasts between A and B. Bonferroni correction applied. BF10 represents Bayesian factor of 10 results.^*

**Table S5**

MCC repeated measures ANOVA for non-rescored and rescored algorithms - CPAP^a^

| Source | Rescore | SS | MS | *F* | η²g | Eps (ε) | *df* | *P_uncorr._* | df*_adjusted_* | *P_adjusted_* |
| --- | --- | --- | --- | --- | --- | --- | --- | --- | --- | --- |
| MCC_algorithm_ | NRS | 0.04 | 0.01 | 1.91 | 0.002 | 0.22 | 6 | .079 | 1.30 | .168 |
| MCC_Error_ | NRS | 1.21 | 0.003 |  |  |  | 366 |  | 79.21 |  |
| MCC_algorithm_ | RS | 0.03 | 0.005 | 1.09 | 0.002 | 0.23 | 6 | .366 | 1.38 | .320 |
| MCC_Error_ | RS | 1.61 | 0.004 |  |  |  | 366 |  | 84.13 |  |

*^a. Adjusted values for df and p values refer to respective Greenhouse Geiser corrections, SS = sum of squares MS = mean squares , and η²g = general eta squared.^*

**Table S6**

Cohens Kappa repeated measures ANOVA for non-rescored and rescored algorithms - CPAP^a^

| Source | Rescore | SS | MS | *F* | η²g | Eps (ε) | *df* | *P_uncorr._* | df*_adjusted_* | *P_adjusted_* |
| --- | --- | --- | --- | --- | --- | --- | --- | --- | --- | --- |
| K_algorithm_ | NRS | 0.23 | 0.04 | 8.31 | 0.01 | 0.20 | 6 | p<.001 | 1.23 | .003 |
| K_Error_ | NRS | 1.72 | 0.004 |  |  |  | 366 |  | 74.80 |  |
| K_algorithm_ | RS | 0.09 | 0.01 | 2.23 | 0.004 | 0.21 | 6 | .039 | 1.28 | .1331 |
| K_Error_ | RS | 2.40 | 0.01 |  |  |  | 366 |  | 78.24 |  |

*^a. Adjusted values for df and p values refer to respective Greenhouse Geiser corrections, SS = sum of squares MS = mean squares , and η²g = general eta squared.^*

**Table S7**

Cohens Kappa post hoc results for non-rescored and rescored algorithms - CPAP^a^

| **A** | **B** | ***T*** | ***df*** | ***P_uncorr._*** | ***P_adjusted_*** | **BF10** | **Hedge’s *g*** |
| --- | --- | --- | --- | --- | --- | --- | --- |
| K2010-nrs | UCSD-nrs | 3.21 | 61 | .002 | .045 | 13.38 | 0.30 |
| K2010-nrs | CK-nrs | 1.36 | 61 | .177 | 1.000 | 0.34 | 0.10 |
| K2010-nrs | Philips-20-nrs | 3.62 | 61 | .001 | .013 | 41.95 | 0.09 |
| K2010-nrs | Philips-40-nrs | 1.97 | 61 | .053 | 1.000 | 0.85 | 0.08 |
| K2010-nrs | Philips-80-nrs | 2.52 | 61 | .014 | .302 | 2.53 | 0.17 |
| K2010-nrs | Sadeh-nrs | 3.11 | 61 | .003 | .059 | 10.54 | 0.31 |
| UCSD-nrs | CK-nrs | -7.55 | 61 | p<.001 | p<.001 | 3.95 | -0.20 |
| UCSD-nrs | Philips-20-nrs | -2.56 | 61 | .013 | .271 | 2.78 | -0.22 |
| UCSD-nrs | Philips-40-nrs | -3.66 | 61 | .001 | .011 | 47.05 | -0.23 |
| UCSD-nrs | Philips-80-nrs | -3.98 | 61 | p<.001 | .004 | 122.62 | -0.13 |
| UCSD-nrs | Sadeh-nrs | 1.09 | 61 | .280 | 1.000 | 0.24 | 0.02 |
| CK-nrs | Philips-20-nrs | -0.18 | 61 | .855 | 1.000 | 0.14 | -0.01 |
| CK-nrs | Philips-40-nrs | -0.54 | 61 | .592 | 1.000 | 0.16 | -0.02 |
| CK-nrs | Philips-80-nrs | 4.21 | 61 | p<.001 | .002 | 246.15 | 0.07 |
| CK-nrs | Sadeh-nrs | 5.62 | 61 | p<.001 | p<.001 | 3.04 | 0.22 |
| Philips-20-nrs | Philips-40-nrs | -0.34 | 61 | .737 | 1.000 | 0.15 | -0.01 |
| Philips-20-nrs | Philips-80-nrs | 1.40 | 61 | .165 | 1.000 | 0.35 | 0.08 |
| Philips-20-nrs | Sadeh-nrs | 2.55 | 61 | .013 | .279 | 2.71 | 0.24 |
| Philips-40-nrs | Philips-80-nrs | 2.82 | 61 | .007 | .137 | 5.01 | 0.09 |
| Philips-40-nrs | Sadeh-nrs | 3.40 | 61 | .001 | .025 | 22.85 | 0.24 |
| Philips-80-nrs | Sadeh-nrs | 3.37 | 61 | .001 | .028 | 20.67 | 0.15 |

*^a. Contrasts between A and B. Bonferroni correction applied. BF10 represents Bayesian factor of 10 results.^*

**Table S8**

MCC repeated measures ANOVA for non-rescored and rescored algorithms - Insomnia^a^

| Source | Rescore | SS | MS | *F* | η²g | Eps (ε) | *df* | *P_uncorr._* | df*_adjusted_* | *P_adjusted_* |
| --- | --- | --- | --- | --- | --- | --- | --- | --- | --- | --- |
| MCC_algorithm_ | NRS | 0.12 | 0.02 | 9.19 | 0.01 | 0.25 | 6 | p<.001 | 1.50 | .001 |
| MCC_Error_ | NRS | 1.05 | 0.002 |  |  |  | 498 |  | 124.90 |  |
| MCC_algorithm_ | RS | 0.03 | 0.01 | 1.86 | 0.001 | 0.28 | 6 | .085 | 1.70 | .165 |
| MCC_Error_ | RS | 1.44 | 0.003 |  |  |  | 498 |  | 141.08 |  |

*^a. Adjusted values for df and p values refer to respective Greenhouse Geiser corrections, SS = sum of squares MS = mean squares , and η²g = general eta squared.^*

**Table S9**

MCC post hoc results for non-rescored algorithms - Insomnia^a^

| **A** | **B** | ***T*** | ***df*** | ***P_uncorr._*** | ***P_ajdusted_*** | **BF10** | **Hedge’s *g*** |
| --- | --- | --- | --- | --- | --- | --- | --- |
| K2010-nrs | UCSD-nrs | 4.10 | 83 | p<.001 | .002 | 206.46 | 0.23 |
| K2010-nrs | CK-nrs | 2.09 | 83 | .039 | .827 | 0.95 | 0.09 |
| K2010-nrs | Philips-20-nrs | 4.10 | 83 | p<.001 | .002 | 201.09 | 0.10 |
| K2010-nrs | Philips-40-nrs | 3.48 | 83 | .001 | .017 | 28.72 | 0.11 |
| K2010-nrs | Philips-80-nrs | 4.31 | 83 | p<.001 | .001 | 407.51 | 0.18 |
| K2010-nrs | Sadeh-nrs | 3.15 | 83 | .002 | .048 | 11.23 | 0.20 |
| UCSD-nrs | CK-nrs | -7.09 | 83 | p<.001 | p<.001 | 2.43E+07 | -0.14 |
| UCSD-nrs | Philips-20-nrs | -2.58 | 83 | .012 | .246 | 2.67 | -0.13 |
| UCSD-nrs | Philips-40-nrs | -3.43 | 83 | .001 | .020 | 24.75 | -0.13 |
| UCSD-nrs | Philips-80-nrs | -2.22 | 83 | .029 | .605 | 1.24 | -0.05 |
| UCSD-nrs | Sadeh-nrs | -2.14 | 83 | .036 | .746 | 1.04 | -0.03 |
| CK-nrs | Philips-20-nrs | 0.30 | 83 | .761 | 1.000 | 0.13 | 0.01 |
| CK-nrs | Philips-40-nrs | 0.62 | 83 | .535 | 1.000 | 0.15 | 0.02 |
| CK-nrs | Philips-80-nrs | 5.57 | 83 | p<.001 | p<.001 | 4.43E+04 | 0.09 |
| CK-nrs | Sadeh-nrs | 3.85 | 83 | p<.001 | .005 | 90.10 | 0.11 |
| Philips-20-nrs | Philips-40-nrs | 0.22 | 83 | .823 | 1.000 | 0.12 | 0.00 |
| Philips-20-nrs | Philips-80-nrs | 2.35 | 83 | .021 | .445 | 1.61 | 0.08 |
| Philips-20-nrs | Sadeh-nrs | 1.75 | 83 | .084 | 1.000 | 0.52 | 0.10 |
| Philips-40-nrs | Philips-80-nrs | 3.97 | 83 | p<.001 | .003 | 133.49 | 0.07 |
| Philips-40-nrs | Sadeh-nrs | 2.15 | 83 | .035 | .725 | 1.06 | 0.10 |
| Philips-80-nrs | Sadeh-nrs | 0.67 | 83 | .504 | 1.000 | 0.15 | 0.02 |

*^a. Contrasts between A and B. Bonferroni correction applied. BF10 represents Bayesian factor of 10 results.^*

**Table S10**

Cohens Kappa repeated measures ANOVA for non-rescored and rescored algorithms - Insomnia^a^

| Source | Rescore | SS | MS | *F* | η²g | Eps (ε) | *df* | *P_uncorr._* | df*_adjusted_* | *P_adjusted_* |
| --- | --- | --- | --- | --- | --- | --- | --- | --- | --- | --- |
| K_algorithm_ | NRS | 0.53 | 0.09 | 28.14 | 0.02 | 0.22 | 6 | p<.001 | 1.32 | p<.001 |
| K_Error_ | NRS | 1.56 | 0.003 |  |  |  | 498 |  | 109.90 |  |
| K_algorithm_ | RS | 0.16 | 0.03 | 5.94 | 0.01 | 0.24 | 6 | p<.001 | 1.42 | .008 |
| K_Error_ | RS | 2.27 | 0.004 |  |  |  | 498 |  | 117.76 |  |

*^a. Adjusted values for df and p values refer to respective Greenhouse Geiser corrections, SS = sum of squares MS = mean squares , and η²g = general eta squared.^*

**Table S11**

Cohens Kappa post hoc results for non-rescored and rescored algorithms - Insomnia^a^

| **A** | **B** | ***T*** | ***df*** | ***P_uncorr._*** | ***P_adjusted_*** | **BF10** | **Hedge’s *g*** |
| --- | --- | --- | --- | --- | --- | --- | --- |
| K2010-nrs | UCSD-nrs | 5.88 | 83 | p<.001 | p<.001 | 1.51E+05 | 0.41 |
| K2010-nrs | CK-nrs | 3.47 | 83 | .001 | .017 | 28.34 | 0.18 |
| K2010-nrs | Philips-20-nrs | 3.65 | 83 | p<.001 | .010 | 48.70 | 0.09 |
| K2010-nrs | Philips-40-nrs | 3.45 | 83 | .001 | .019 | 26.38 | 0.13 |
| K2010-nrs | Philips-80-nrs | 4.95 | 83 | p<.001 | p<.001 | 4095.01 | 0.26 |
| K2010-nrs | Sadeh-nrs | 5.70 | 83 | p<.001 | p<.001 | 7.37E+04 | 0.43 |
| UCSD-nrs | CK-nrs | -10.35 | 83 | p<.001 | p<.001 | 4.61E+13 | -0.21 |
| UCSD-nrs | Philips-20-nrs | -5.31 | 83 | p<.001 | p<.001 | 1.60E+04 | -0.31 |
| UCSD-nrs | Philips-40-nrs | -6.78 | 83 | p<.001 | p<.001 | 6.59E+06 | -0.28 |
| UCSD-nrs | Philips-80-nrs | -6.19 | 83 | p<.001 | p<.001 | 5.40E+05 | -0.14 |
| UCSD-nrs | Sadeh-nrs | 1.76 | 83 | .081 | 1.000 | 0.53 | 0.02 |
| CK-nrs | Philips-20-nrs | -2.07 | 83 | .041 | .863 | 0.92 | -0.09 |
| CK-nrs | Philips-40-nrs | -2.16 | 83 | .034 | .707 | 1.09 | -0.06 |
| CK-nrs | Philips-80-nrs | 5.25 | 83 | p<.001 | p<.001 | 1.29E+04 | 0.07 |
| CK-nrs | Sadeh-nrs | 8.43 | 83 | p<.001 | p<.001 | 9.03E+09 | 0.23 |
| Philips-20-nrs | Philips-40-nrs | 1.53 | 83 | .130 | 1.000 | 0.37 | 0.03 |
| Philips-20-nrs | Philips-80-nrs | 4.15 | 83 | p<.001 | .002 | 241.15 | 0.17 |
| Philips-20-nrs | Sadeh-nrs | 5.17 | 83 | p<.001 | p<.001 | 9416.15 | 0.34 |
| Philips-40-nrs | Philips-80-nrs | 6.17 | 83 | p<.001 | p<.001 | 5.00E+05 | 0.14 |
| Philips-40-nrs | Sadeh-nrs | 6.29 | 83 | p<.001 | p<.001 | 8.27E+05 | 0.30 |
| Philips-80-nrs | Sadeh-nrs | 5.45 | 83 | p<.001 | p<.001 | 2.79E+04 | 0.16 |
| K2010-rs | UCSD-rs | 1.71 | 83 | .091 | 1.000 | 0.48 | 0.15 |
| K2010-rs | CK-rs | -0.83 | 83 | .410 | 1.000 | 0.17 | -0.05 |
| K2010-rs | Philips-20-rs | -0.71 | 83 | .483 | 1.000 | 0.15 | -0.03 |
| K2010-rs | Philips-40-rs | -1.09 | 83 | .277 | 1.000 | 0.21 | -0.06 |
| K2010-rs | Philips-80-rs | 0.63 | 83 | .530 | 1.000 | 0.15 | 0.04 |
| K2010-rs | Sadeh-rs | 1.70 | 83 | .092 | 1.000 | 0.48 | 0.15 |
| UCSD-rs | CK-rs | -6.99 | 83 | p<.001 | p<.001 | 1.57E+07 | -0.19 |
| UCSD-rs | Philips-20-rs | -2.45 | 83 | .016 | .342 | 2.01 | -0.17 |
| UCSD-rs | Philips-40-rs | -4.30 | 83 | p<.001 | .001 | 402.61 | -0.20 |
| UCSD-rs | Philips-80-rs | -3.94 | 83 | p<.001 | .004 | 121.67 | -0.10 |
| UCSD-rs | Sadeh-rs | 0.24 | 83 | .811 | 1.000 | 0.12 | 0.00 |
| CK-rs | Philips-20-rs | 0.56 | 83 | .580 | 1.000 | 0.14 | 0.03 |
| CK-rs | Philips-40-rs | -0.09 | 83 | .927 | 1.000 | 0.12 | 0.00 |
| CK-rs | Philips-80-rs | 5.06 | 83 | p<.001 | p<.001 | 6249.44 | 0.09 |
| CK-rs | Sadeh-rs | 6.23 | 83 | p<.001 | p<.001 | 6.54E+05 | 0.19 |
| Philips-20-rs | Philips-40-rs | -1.07 | 83 | .287 | 1.000 | 0.21 | -0.03 |
| Philips-20-rs | Philips-80-rs | 1.33 | 83 | .187 | 1.000 | 0.28 | 0.07 |
| Philips-20-rs | Sadeh-rs | 2.43 | 83 | .017 | .362 | 1.92 | 0.17 |
| Philips-40-rs | Philips-80-rs | 3.49 | 83 | .001 | .016 | 29.70 | 0.10 |
| Philips-40-rs | Sadeh-rs | 4.12 | 83 | p<.001 | .002 | 215.24 | 0.20 |
| Philips-80-rs | Sadeh-rs | 3.37 | 83 | .001 | .024 | 21.28 | 0.10 |

*^a. Contrasts between A and B. Bonferroni correction applied. BF10 represents Bayesian factor of 10 results.^*

**Table S12**

MCC repeated measures ANOVA for non-rescored and rescored algorithms - Insomnia^a^

| Source | Rescore | SS | MS | *F* | η²g | Eps (ε) | *df* | *P_uncorr._* | df*_adjusted_* | *P_adjusted_* |
| --- | --- | --- | --- | --- | --- | --- | --- | --- | --- | --- |
| MCC_algorithm_ | NRS | 0.03 | 0.01 | 2.67 | 0.002 | 0.23 | 6 | 0.015 | 1.38 | 0.093 |
| MCC_Error_ | NRS | 0.82 | 0.002 |  |  |  | 390 |  | 89.57 |  |
| MCC_algorithm_ | RS | 0.03 | 0.01 | 1.77 | 0.002 | 0.25 | 6 | 0.104 | 1.50 | 0.183 |
| MCC_Error_ | RS | 1.17 | 0.003 |  |  |  | 390 |  | 97.71 |  |

*^a. Adjusted values for df and p values refer to respective Greenhouse Geiser corrections, SS = sum of squares MS = mean squares , and η²g = general eta squared.^*

**Table S13**

K repeated measures ANOVA for non-rescored and rescored algorithms - RLS^a^

| Source | Rescore | SS | MS | *F* | η²g | Eps (ε) | *df* | *P_uncorr._* | df*_adjusted_* | *P_adjusted_* |
| --- | --- | --- | --- | --- | --- | --- | --- | --- | --- | --- |
| K_algorithm_ | NRS | 0.20 | 0.03 | 12.28 | 0.01 | 0.21 | 6 | p<.001 | 1.24 | p<.001 |
| K_Error_ | NRS | 1.08 | 0.003 |  |  |  | 390 |  | 80.75 |  |
| K_algorithm_ | RS | 0.07 | 0.01 | 2.71 | 0.004 | 0.22 | 6 | .014 | 1.33 | .092 |
| K_Error_ | RS | 1.68 | 0.004 |  |  |  | 390 |  | 86.72 |  |

*^a. Adjusted values for df and p values refer to respective Greenhouse Geiser corrections, SS = sum of squares MS = mean squares , and η²g = general eta squared.^*

**Table S14**

Cohens Kappa post hoc results for non-rescored and rescored algorithms - RLS^a^

| **A** | **B** | ***T*** | ***df*** | ***P_uncorr._*** | ***P_adjusted_*** | **BF10** | **Hedge’s *g*** |
| --- | --- | --- | --- | --- | --- | --- | --- |
| K2010-nrs | UCSD-nrs | 4.13 | 65 | p<.001 | .002 | 201.37 | 0.30 |
| K2010-nrs | CK-nrs | 1.50 | 65 | .139 | 1.000 | 0.39 | 0.08 |
| K2010-nrs | Philips-20-nrs | 3.08 | 65 | .003 | .063 | 9.74 | 0.07 |
| K2010-nrs | Philips-40-nrs | 1.75 | 65 | .085 | 1.000 | 0.57 | 0.07 |
| K2010-nrs | Philips-80-nrs | 2.44 | 65 | .018 | .369 | 2.08 | 0.14 |
| K2010-nrs | Sadeh-nrs | 3.41 | 65 | .001 | .023 | 23.66 | 0.27 |
| UCSD-nrs | CK-nrs | -9.76 | 65 | p<.001 | p<.001 | 3.19E+11 | -0.21 |
| UCSD-nrs | Philips-20-nrs | -3.62 | 65 | .001 | .012 | 42.27 | -0.23 |
| UCSD-nrs | Philips-40-nrs | -5.53 | 65 | p<.001 | p<.001 | 2.42E+04 | -0.24 |
| UCSD-nrs | Philips-80-nrs | -8.05 | 65 | p<.001 | p<.001 | 3.91E+08 | -0.16 |
| UCSD-nrs | Sadeh-nrs | -1.32 | 65 | .190 | 1.000 | 0.31 | -0.02 |
| CK-nrs | Philips-20-nrs | -0.25 | 65 | .800 | 1.000 | 0.14 | -0.01 |
| CK-nrs | Philips-40-nrs | -0.68 | 65 | .497 | 1.000 | 0.17 | -0.02 |
| CK-nrs | Philips-80-nrs | 3.42 | 65 | .001 | .023 | 23.89 | 0.05 |
| CK-nrs | Sadeh-nrs | 6.44 | 65 | p<.001 | p<.001 | 7.43E+05 | 0.19 |
| Philips-20-nrs | Philips-40-nrs | -0.32 | 65 | .747 | 1.000 | 0.14 | -0.01 |
| Philips-20-nrs | Philips-80-nrs | 1.43 | 65 | .157 | 1.000 | 0.36 | 0.07 |
| Philips-20-nrs | Sadeh-nrs | 2.86 | 65 | .006 | .121 | 5.46 | 0.20 |
| Philips-40-nrs | Philips-80-nrs | 2.75 | 65 | .008 | .162 | 4.22 | 0.08 |
| Philips-40-nrs | Sadeh-nrs | 4.11 | 65 | p<.001 | .002 | 190.79 | 0.21 |
| Philips-80-nrs | Sadeh-nrs | 4.81 | 65 | p<.001 | p<.001 | 1897.33 | 0.14 |

*^a. Contrasts between A and B. Bonferroni correction applied. BF10 represents Bayesian factor of 10 results.^*
