# Supplementary material for: Comparison and Validation of Actigraphy Algorithms Using a Large Community Dataset: Algorithm Validation Study
Source: JMIR Form Res. 2025 Dec 11;9:e70778. doi: 10.2196/70778 (PMC12697920; doi:10.2196/70778)

# Multimedia Appendix K: Subgroup Analysis Bland Altman:

## Insomnia Subgroup

### Bland-Altman Distributions Sleep Efficiency

For the Insomnia subpopulation, all algorithms demonstrated some mean difference between the algorithm estimates and PSG measures of sleep efficacy. The Philips Algorithm with a threshold of 40 which had minimal difference. The Bland Altman distribution demonstrated some systematic bias. However, the points were relatively randomly distributed i.e., did not show a specific distribution pattern. There were only a few outliers, and we expect these would not significantly impact results. The estimates do not appear to get worse as sleep efficiency decreased or increased. The Philips threshold 80, Cole-Kriple, Sadeh, and UCSD all overestimated sleep efficacy. While Philips threshold 20 and Kripke 2010 underestimated sleep efficacy.

Rescoring resulted similar results with minor, no improvements, or larger discrepancies in mean difference and distributions. All algorithms demonstrated some mean difference between the algorithm estimates and PSG measures of sleep efficacy. The Cole-Kripke, Philips Threshold 40 and 80 algorithms had minimal difference. The Bland Altman distribution demonstrated some systematic bias. However, the points were relatively randomly distributed i.e., did not show a specific distribution pattern. (All algorithms demonstrated some indication of heteroscedasticity as the spread of points increased as SE decreased. There were only a few outliers, and we expect these would not significantly impact results. The estimates do not appear to get worse as sleep efficiency decreased or increased. The Cole-Kripke, Philips threshold 80, UCSD, and Sadeh all overestimated sleep efficacy. While Philips threshold 40, Philips threshold 20 and Kripke 2010 underestimated sleep efficacy.

**Figure S1**

Bland-Altman distribution sleep efficiency (SE) for Insomnia subgroup non-rescored algorithms.^a^


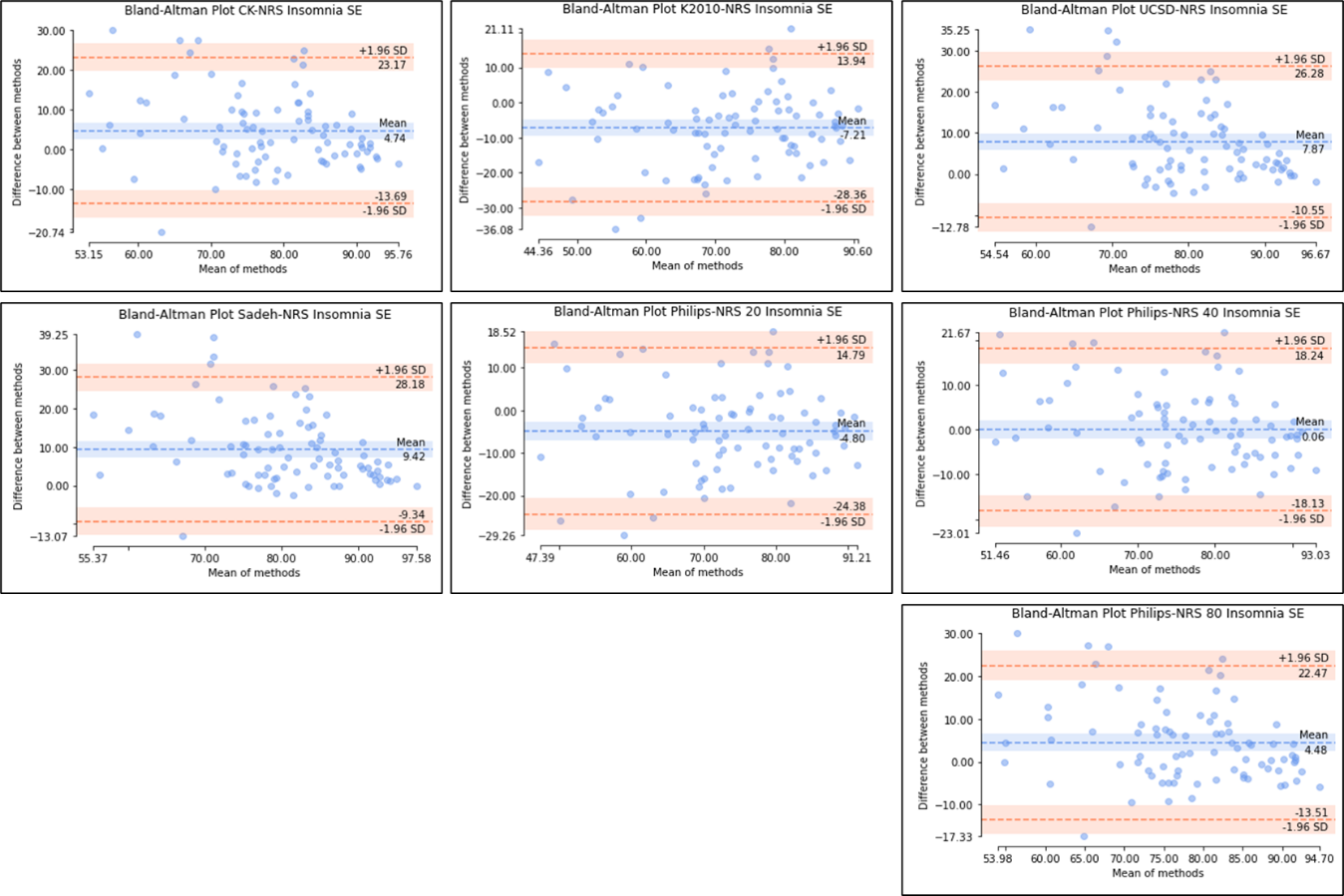


^a. Plots represent the Bland Altman distributions of non-rescored actigraphy algorithms in comparison to polysomnography (PSG; ground truth) for Sleep Efficiency (SE) for the Insomnia subgroup. The y-axis represents the difference between methods while the x-axis represents the mean of methods. The mean difference, standard deviation of the difference, lower and upper limits of agreement (95%) are listed below each respective graph. Each graph showcases the limits in the orange dashed lights while the blue dashed line represents the mean difference. Abbreviated names for each algorithm are as follows: CK = Cole Kripke, K2010 = Kripke 2010, UCSD, Sadeh, and Philips.^

**Figure S2**

Bland-Altman distribution sleep efficiency (SE) for Insomnia subgroup rescored algorithms.^a^


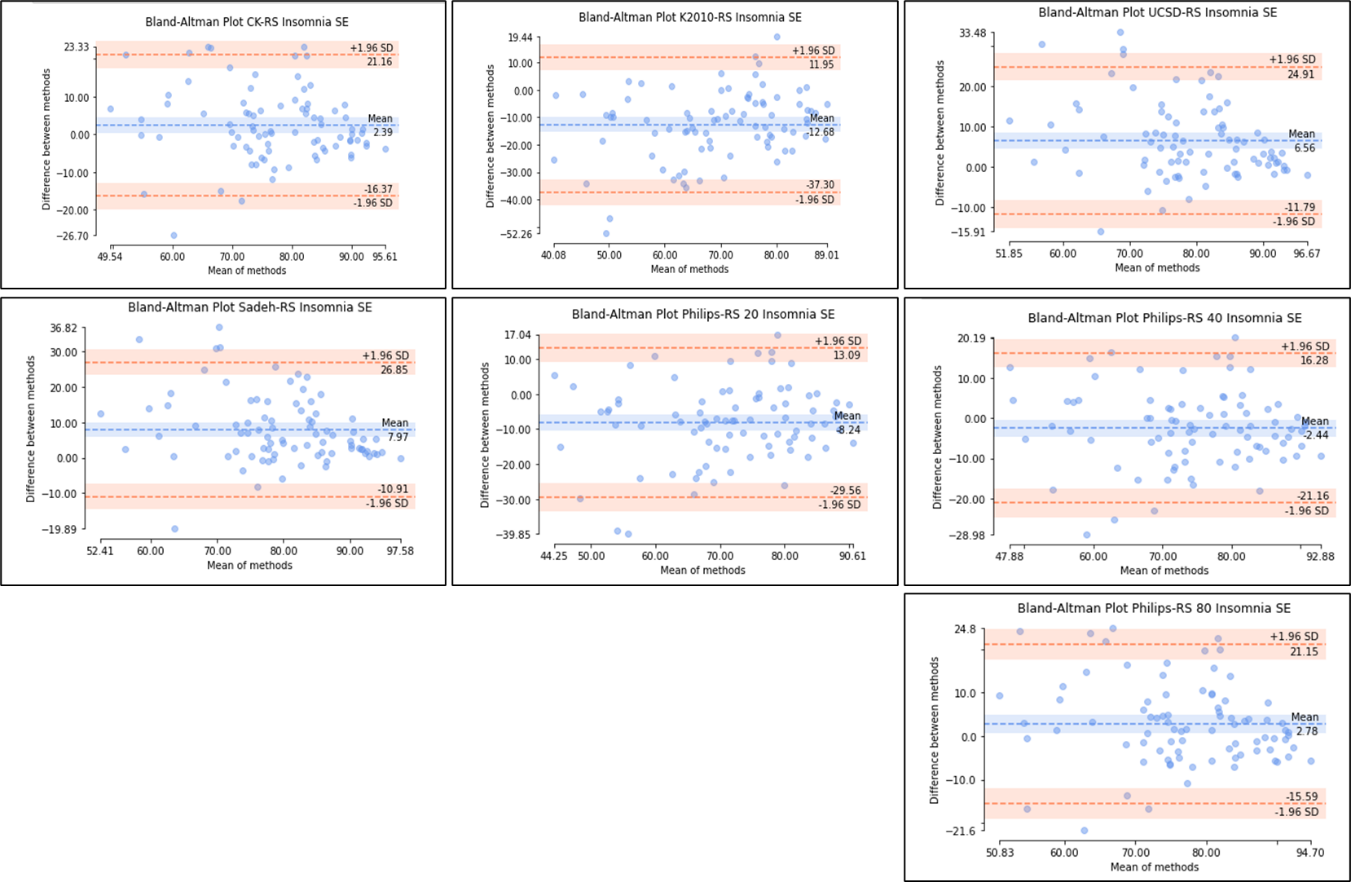


^a. Plots represent the Bland Altman distributions of rescored actigraphy algorithms in comparison to polysomnography (PSG; ground truth) for Sleep Efficiency (SE) for the Insomnia subgroup. The y-axis represents the difference between methods while the x-axis represents the mean of methods. The mean difference, standard deviation of the difference, lower and upper limits of agreement (95%) are listed below each respective graph. Each graph showcases the limits in the orange dashed lights while the blue dashed line represents the mean difference. Abbreviated names for each algorithm are as follows: CK = Cole Kripke, K2010 = Kripke 2010, UCSD, Sadeh, and Philips.^

**Table S1**

Mean difference statistics sleep efficiency (SE) for Insomnia subgroup non-rescored algorithms.


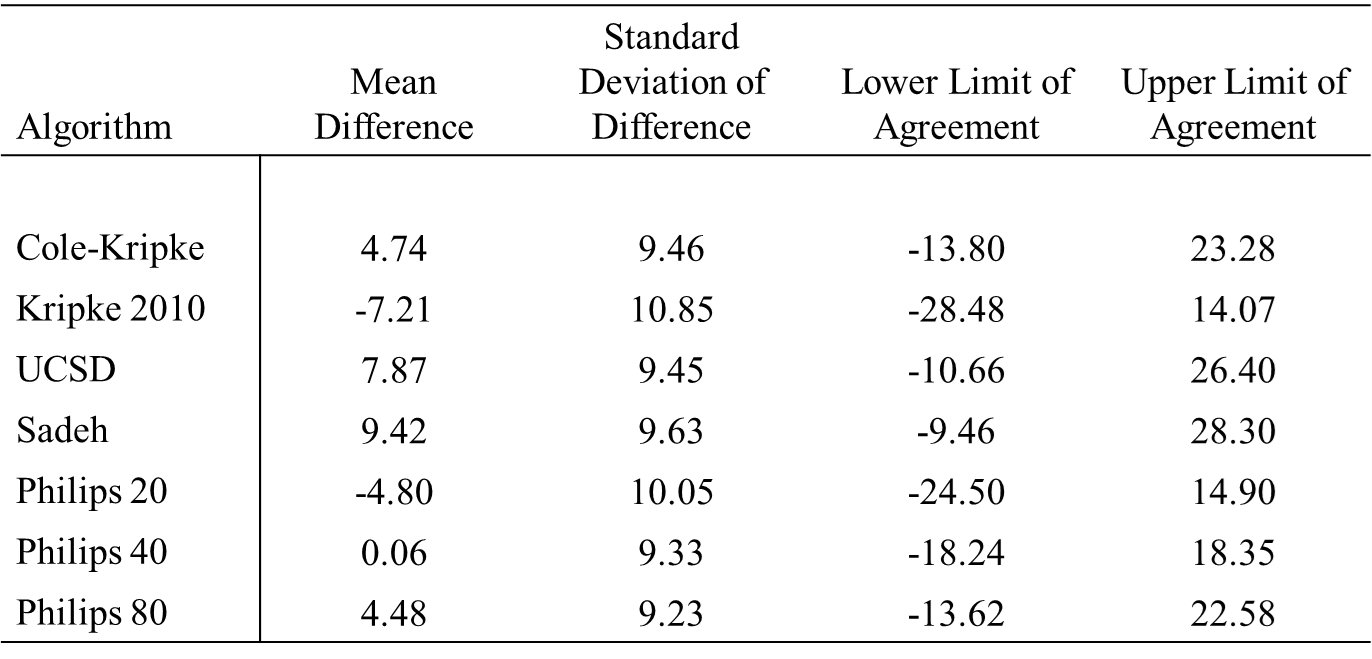


**Table S2**

Mean difference statistics sleep efficiency (SE) for Insomnia subgroup rescored algorithms.


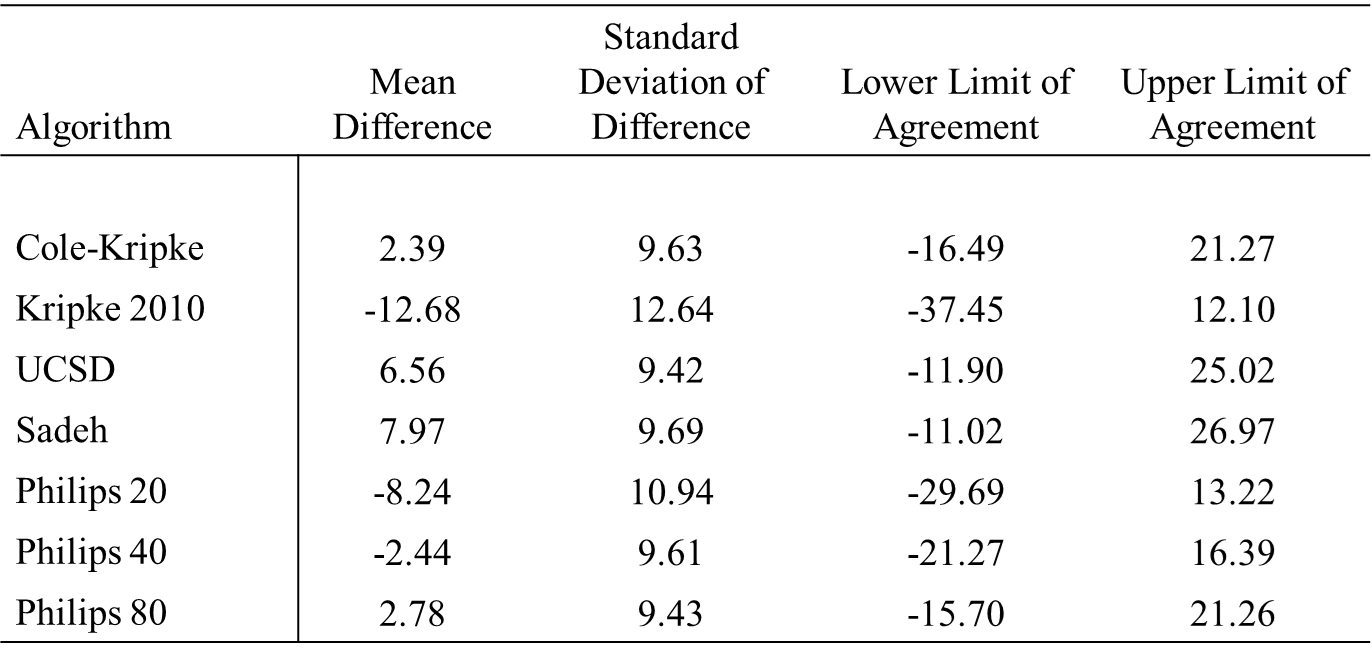


### Bland-Altman Distributions Total Sleep Time

For the Insomnia subpopulation, all algorithms demonstrate some mean difference between the algorithm estimates and PSG measures of total sleep time. The Philips Algorithm with a threshold of 40 had minimal difference. The Bland Altman distribution demonstrated some systematic bias. However, the points were relatively randomly distributed i.e., did not show a specific distribution pattern. There were only a few outliers, and we expect these would not significantly impact results. The estimates do not appear to get worse as total sleep time decreased or increased. With respect to specific algorithms, the Philips threshold 40, Philips threshold 80, Cole-Kriple, UCSD, and Sadeh all overestimated total sleep time. While Philips threshold 20 and Kripke 2010 underestimate total sleep time.

Rescoring resulted similar results with minor, no improvements, or larger discrepancies in mean difference and distributions. All algorithms demonstrate some mean difference between the algorithm estimates and PSG measures of total sleep time. The Philips Algorithm with a threshold of 40 and Cole-Kripke had the smallest difference. The Bland Altman distribution demonstrated some systematic bias. However, the points were relatively randomly distributed i.e., did not show a specific distribution pattern. There were only a few outliers, and we expect these would not significantly impact results. The estimates do not appear to get worse as total sleep time decreased or increased. With respect to specific algorithms, the Cole-Kripke, Philips threshold 80, UCSD, and Sadeh all overestimated total sleep time. While Philips threshold 40, Philips threshold 20 and Kripke 2010 underestimate total sleep time.

**Figure S3**

Bland-Altman distribution total sleep time (TST) for Insomnia subgroup non-rescored algorithms. ^a^


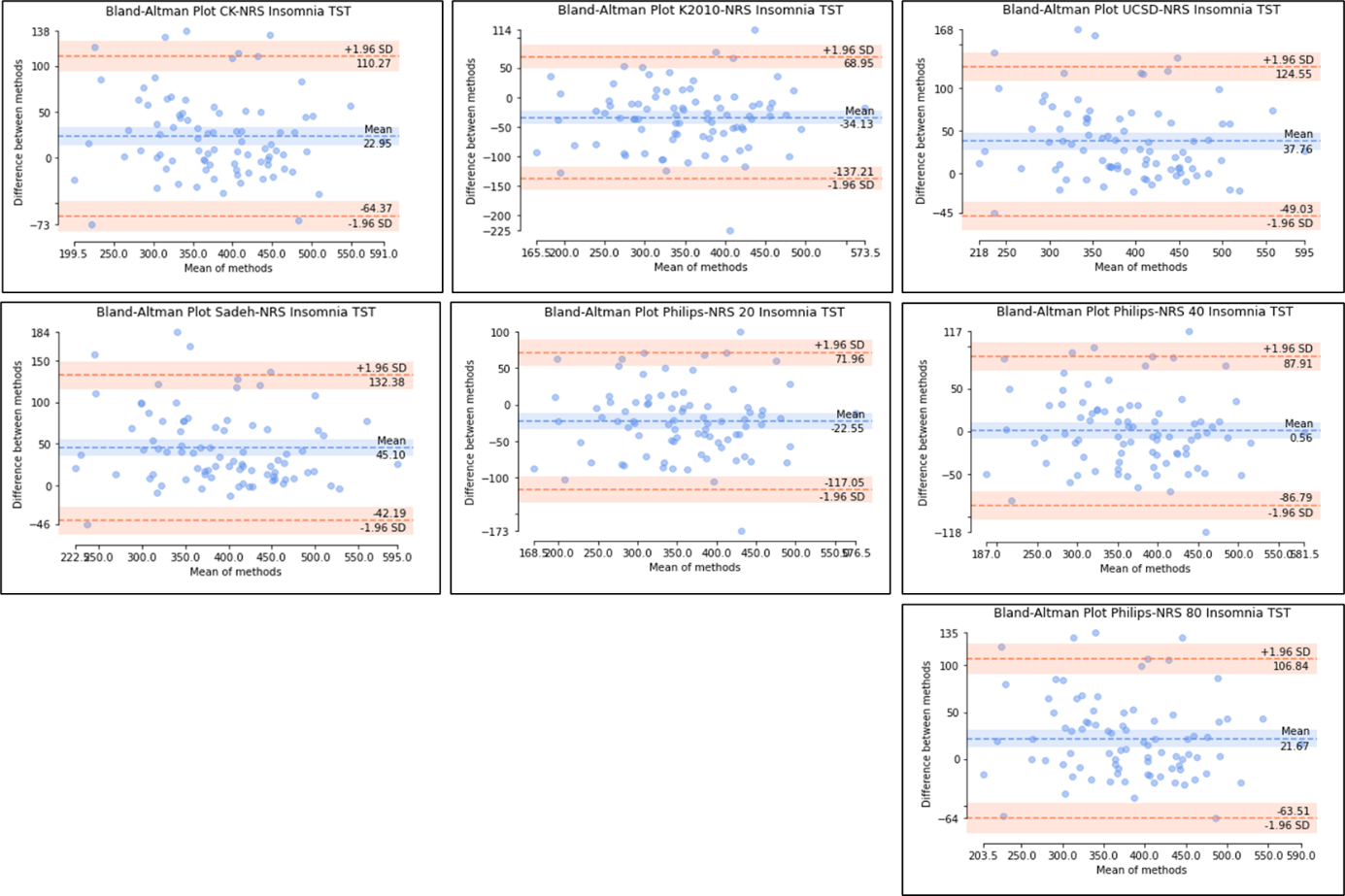


^a. Plots represent the Bland Altman distributions of non-rescored actigraphy algorithms in comparison to polysomnography (PSG; ground truth) for Total Sleep Time (TST) for the Insomnia subgroup. The y-axis represents the difference between methods while the x-axis represents the mean of methods. The mean difference, standard deviation of the difference, lower and upper limits of agreement (95%) are listed below each respective graph. Each graph showcases the limits in the orange dashed lights while the blue dashed line represents the mean difference. Abbreviated names for each algorithm are as follows: CK = Cole Kripke, K2010 = Kripke 2010, UCSD, Sadeh, and Philips.^

**Figure S4**

Mean difference statistics total sleep time (TST) for Insomnia subgroup rescored algorithms.^a^


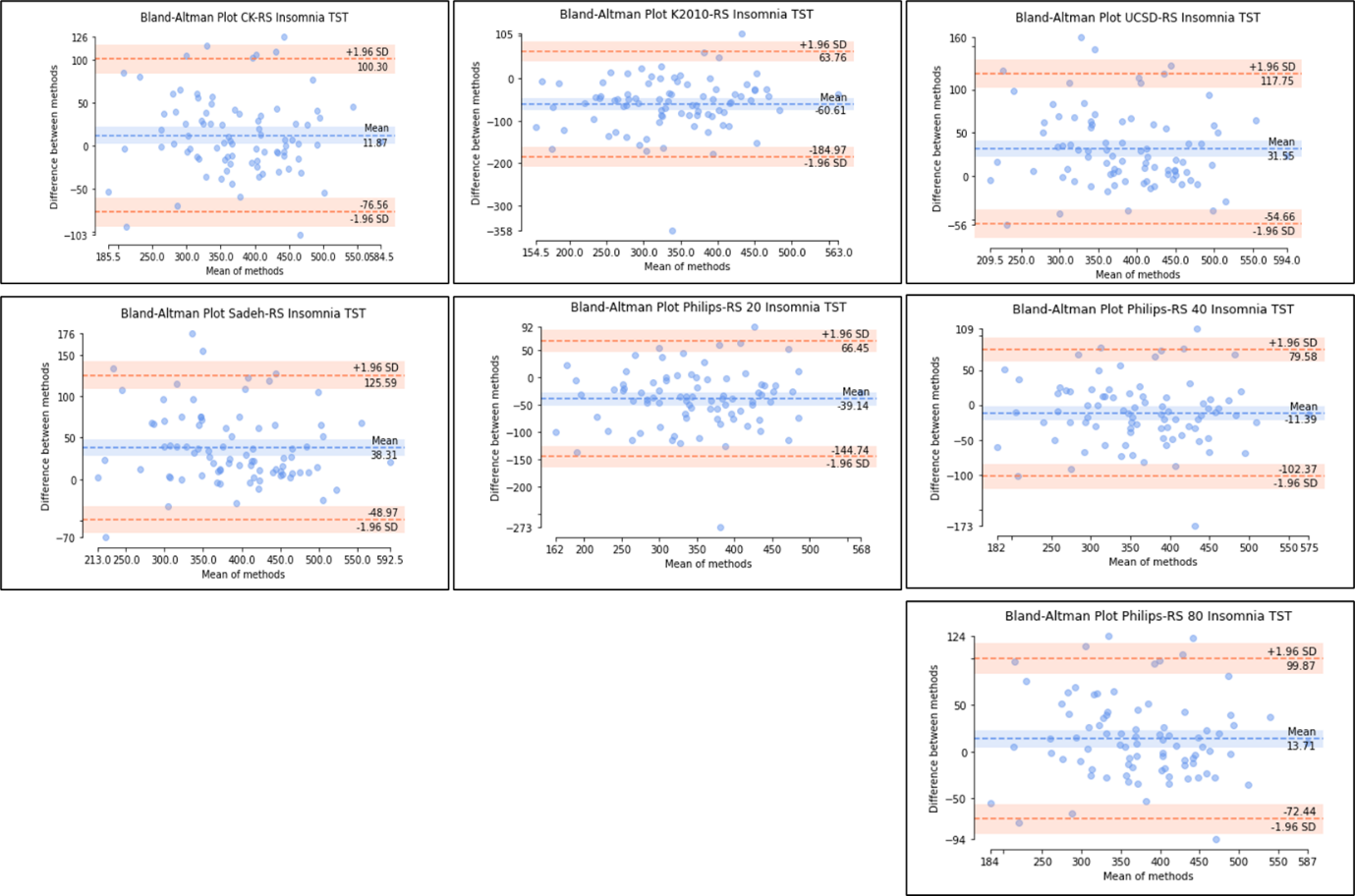


^a. Plots represent the Bland Altman distributions of rescored actigraphy algorithms in comparison to polysomnography (PSG; ground truth) for Total Sleep Time (TST) for the Insomnia subgroup. The y-axis represents the difference between methods while the x-axis represents the mean of methods. The mean difference, standard deviation of the difference, lower and upper limits of agreement (95%) are listed below each respective graph. Each graph showcases the limits in the orange dashed lights while the blue dashed line represents the mean difference. Abbreviated names for each algorithm are as follows: CK = Cole Kripke, K2010 = Kripke 2010, UCSD, Sadeh, and Philips.^

**Table S3**

Mean difference statistics total sleep time (TST) for Insomnia subgroup non-rescored algorithms.


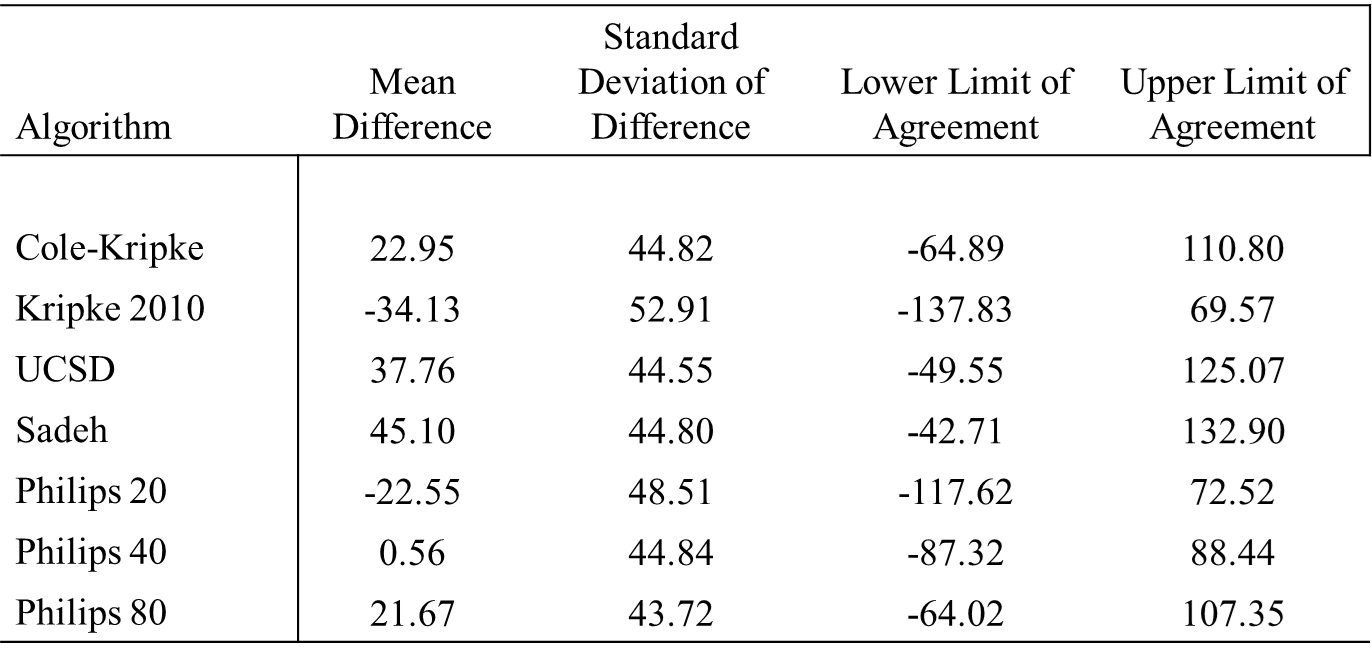


**Table S4**

Mean difference statistics total sleep time (TST) for Insomnia subgroup rescored algorithms.


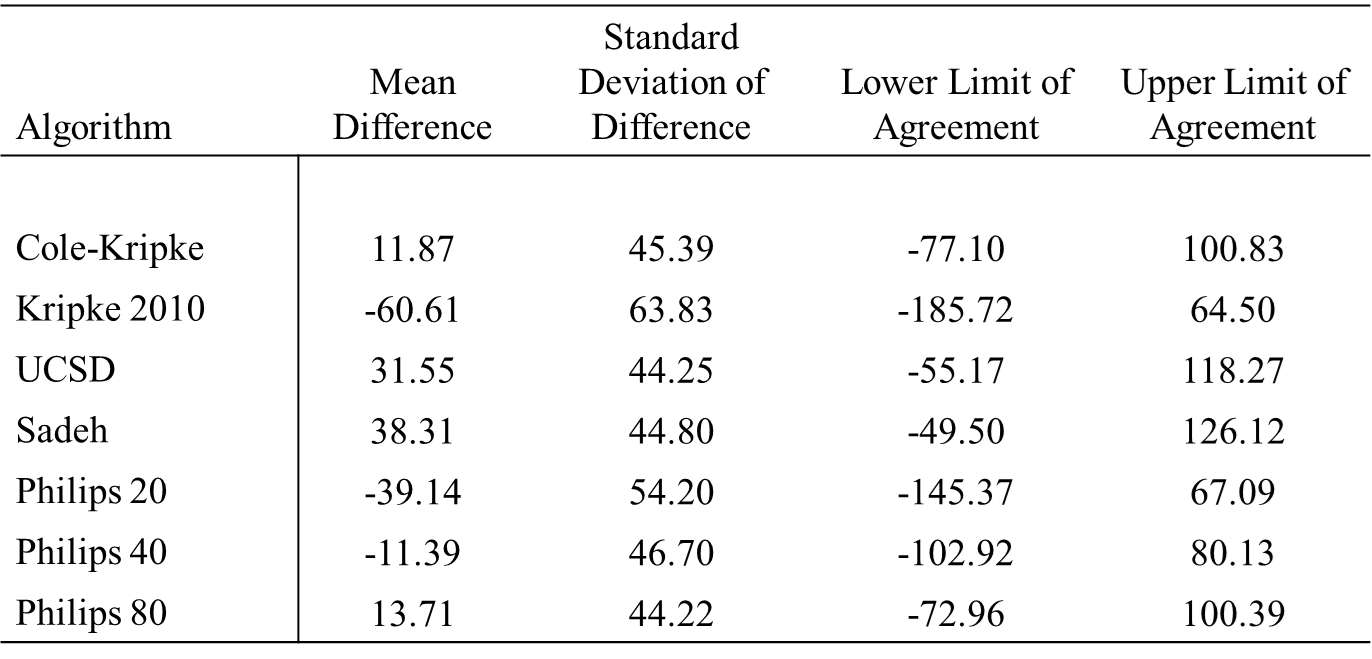


### Bland-Altman Distributions Wake After Sleep Onset

For the Insomnia subpopulation, all algorithms demonstrate some mean difference between the algorithm estimates and PSG measures of WASO. The Philips Algorithm with a threshold of 20 had minimal difference. The Bland Altman distribution demonstrate clear systematic bias. The Cole-Kripke , UCSD, Sadeh, and Philips Threshold 80 demonstrated clear heteroscedasticity. That is, WASO estimates were better at lower WASO averages and become considerably worse as WASO increased. There were only a few outliers, and we expect these would not significantly impact results. The Philips algorithms, Cole-Kripke, UCSD, and Sadeh all underestimated WASO. While Kripke 2010 overestimated total sleep WASO.

Rescoring resulted similar results with minor, no improvements, or larger discrepancies in mean difference and distributions. All algorithms demonstrate some mean difference between the algorithm estimates and PSG measures of WASO. The Philips Algorithm with a threshold of 40 had the smallest difference. The Bland Altman distribution demonstrate clear systematic bias. The Cole-Kripke , UCSD, Sadeh, and Philips Threshold 80 demonstrated clear heteroscedasticity. That is, WASO estimates were better at lower WASO averages and become considerably worse as WASO increased. There were only a few outliers, and we expect these would not significantly impact results. The Philips with threshold 20 and Kripke 2010 overestimated total sleep WASO. While Philips with threshold 40, Philips with threshold 80, Cole-Kripke, UCSD, and Sadeh algorithms all underestimated WASO.

**Figure S5**

Mean difference statistics wake after sleep onset (WASO) for Insomnia subgroup non-rescored algorithms.^a^


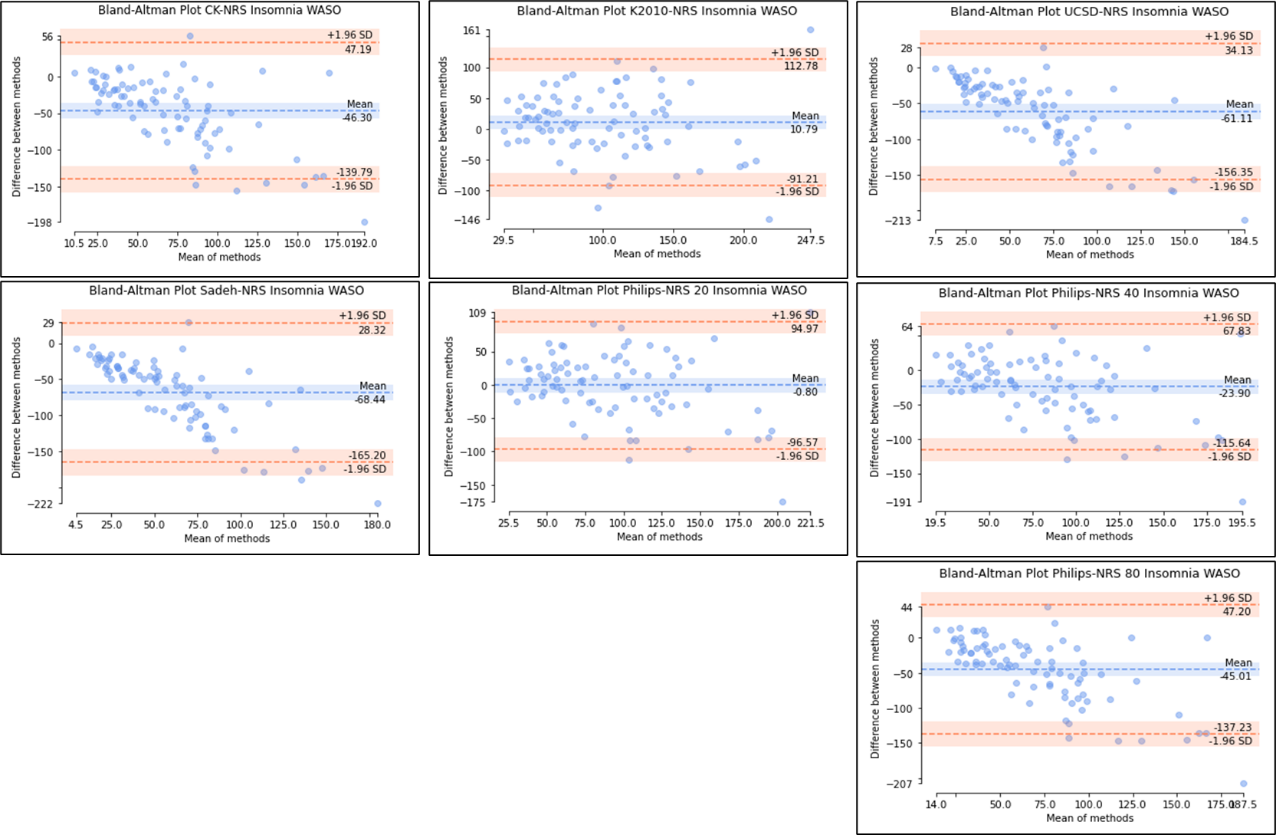


^a. Plots represent the Bland Altman distributions of non-rescored actigraphy algorithms in comparison to polysomnography (PSG; ground truth) for Wake After Sleep Onset (WASO) for the Insomnia subgroup. The y-axis represents the difference between methods while the x-axis represents the mean of methods. The mean difference, standard deviation of the difference, lower and upper limits of agreement (95%) are listed below each respective graph. Each graph showcases the limits in the orange dashed lights while the blue dashed line represents the mean difference. Abbreviated names for each algorithm are as follows: CK = Cole Kripke, K2010 = Kripke 2010, UCSD, Sadeh, and Philips.^

**Figure S6**

Mean difference statistics wake after sleep onset (WASO) for Insomnia subgroup rescored algorithms.^a^


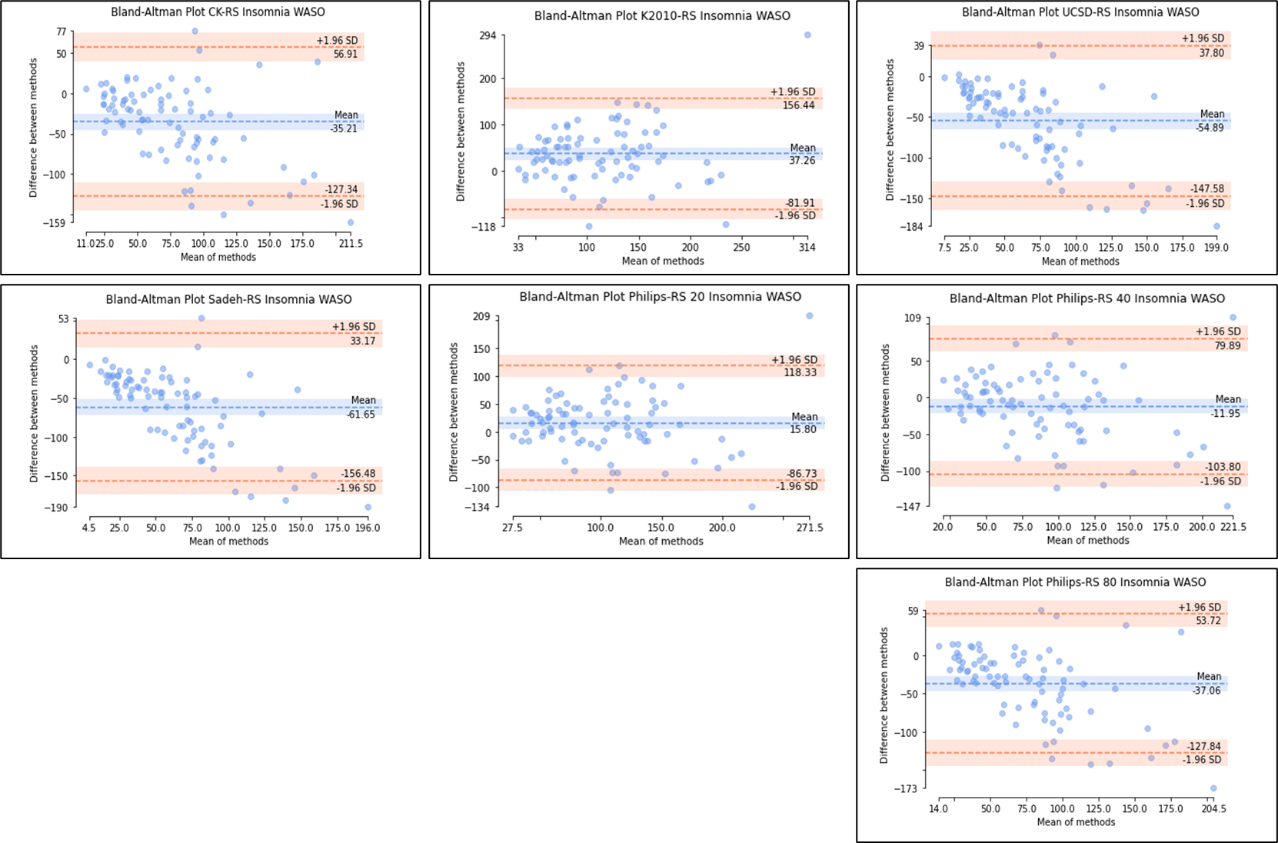


^a. Plots represent the Bland Altman distributions of rescored actigraphy algorithms in comparison to polysomnography (PSG; ground truth) for Wake After Sleep Onset (WASO) for the Insomnia subgroup. The y-axis represents the difference between methods while the x-axis represents the mean of methods. The mean difference, standard deviation of the difference, lower and upper limits of agreement (95%) are listed below each respective graph. Each graph showcases the limits in the orange dashed lights while the blue dashed line represents the mean difference. Abbreviated names for each algorithm are as follows: CK = Cole Kripke, K2010 = Kripke 2010, UCSD, Sadeh, and Philips.^

**Table S5**

Mean difference statistics wake after sleep onset (WASO) for Insomnia subgroup non-rescored algorithms.


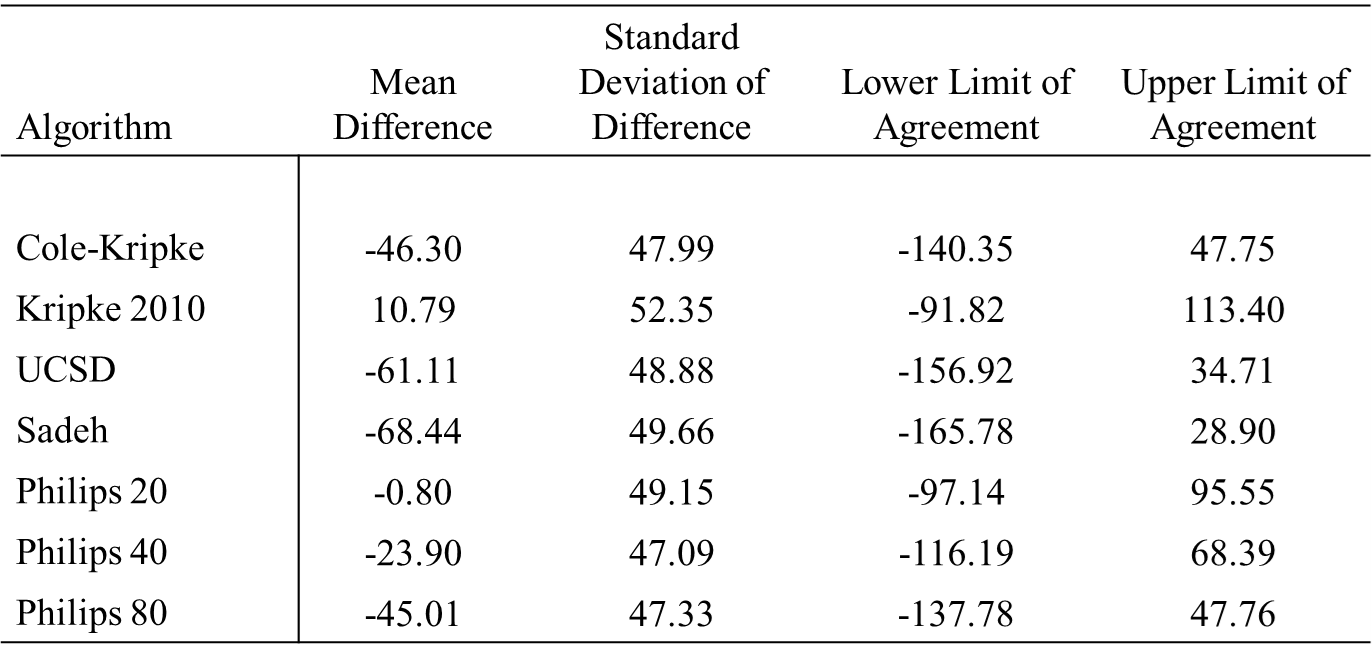


**Table S6**

Mean difference statistics wake after sleep onset (WASO) for Insomnia subgroup rescored algorithms.


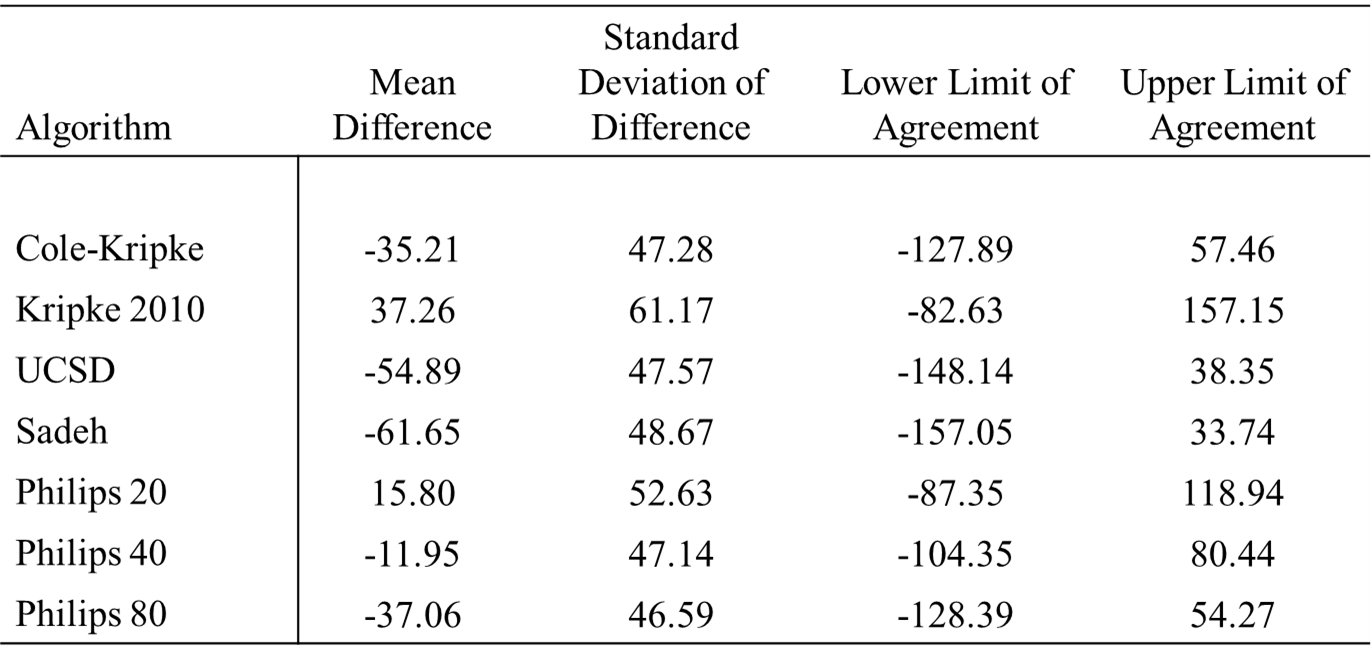


## Restless Leg Syndrome (RLS) Subgroup

### Bland-Altman Distributions Sleep Efficiency

For the RLS subpopulation, all algorithms demonstrated some mean difference between the algorithm estimates and PSG measures of sleep efficacy. The Philips Algorithm with a threshold of 40 which had minimal difference. The Bland Altman distribution demonstrated some systematic bias. However, the points were relatively randomly distributed i.e., did not show a specific distribution pattern. There were only a few outliers, and we expect these would not significantly impact results. The estimates do not appear to get worse as sleep efficiency decreased or increased. The Philips threshold 80, Cole-Kriple, UCSD, and Sadeh all overestimated sleep efficacy. While Philips threshold 40, Philips threshold 20, and Kripke 2010 underestimated sleep efficacy.

Rescoring resulted similar results with minor, no improvements, or larger discrepancies in mean difference and distributions. All algorithms demonstrated some mean difference between the algorithm estimates and PSG measures of sleep efficacy. The Cole-Kripke and Philips Algorithm with a threshold of 80 which had minimal difference. The Bland Altman distribution demonstrated some systematic bias. However, the points were relatively randomly distributed i.e., did not show a specific distribution pattern. There were only a few outliers, and we expect these would not significantly impact results. The estimates do not appear to get worse as sleep efficiency decreased or increased. The Cole-Kripke, Philips threshold 80, UCSD, and Sadeh algorithms all overestimated sleep efficacy. While Philips threshold 40, Philips threshold 20, and Kripke 2010 underestimated sleep efficacy.

**Figure S7**

Mean difference statistics sleep efficacy (SE) for RLS subgroup non-rescored algorithms.^a^


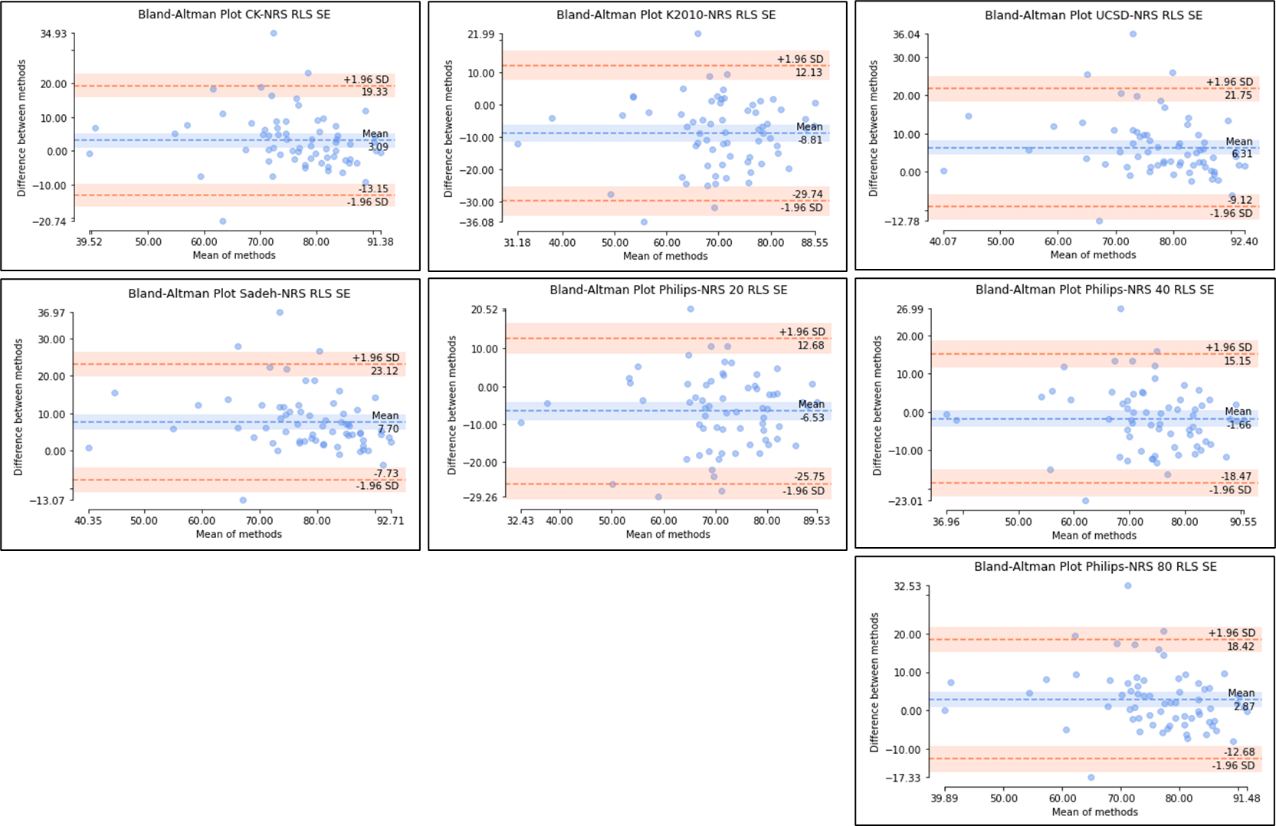


^a. Plots represent the Bland Altman distributions of non-rescored actigraphy algorithms in comparison to polysomnography (PSG; ground truth) for Sleep Efficacy (SE) for the RLS subgroup. The y-axis represents the difference between methods while the x-axis represents the mean of methods. The mean difference, standard deviation of the difference, lower and upper limits of agreement (95%) are listed below each respective graph. Each graph showcases the limits in the orange dashed lights while the blue dashed line represents the mean difference. Abbreviated names for each algorithm are as follows: CK = Cole Kripke, K2010 = Kripke 2010, UCSD, Sadeh, and Philips.^

**Figure S8**

Mean difference statistics sleep efficacy (SE) for RLS subgroup rescored algorithms.^a^


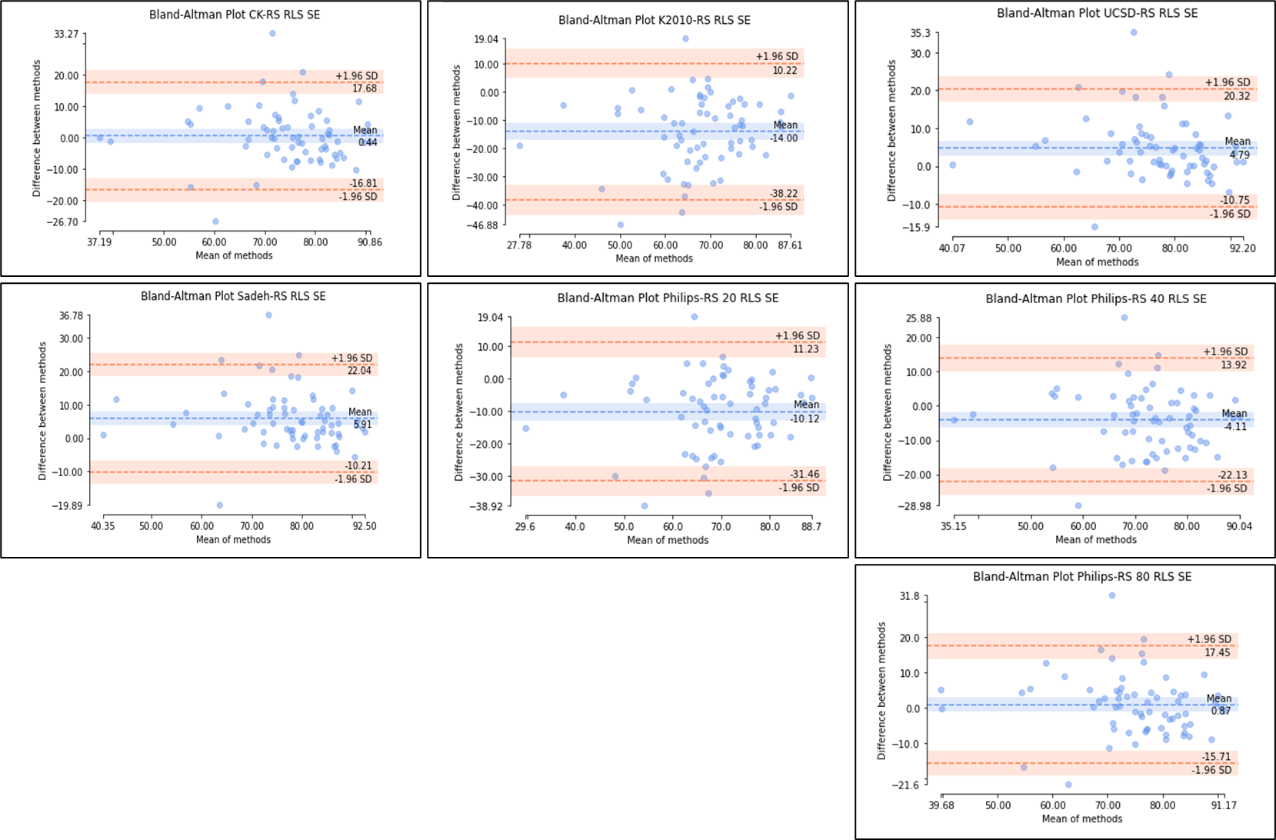


^a. Plots represent the Bland Altman distributions of rescored actigraphy algorithms in comparison to polysomnography (PSG; ground truth) for Sleep Efficacy (SE) for the RLS subgroup. The y-axis represents the difference between methods while the x-axis represents the mean of methods. The mean difference, standard deviation of the difference, lower and upper limits of agreement (95%) are listed below each respective graph. Each graph showcases the limits in the orange dashed lights while the blue dashed line represents the mean difference. Abbreviated names for each algorithm are as follows: CK = Cole Kripke, K2010 = Kripke 2010, UCSD, Sadeh, and Philips.^

**Table S7**

Mean difference statistics sleep efficacy (SE) for RLS subgroup non-rescored algorithms.


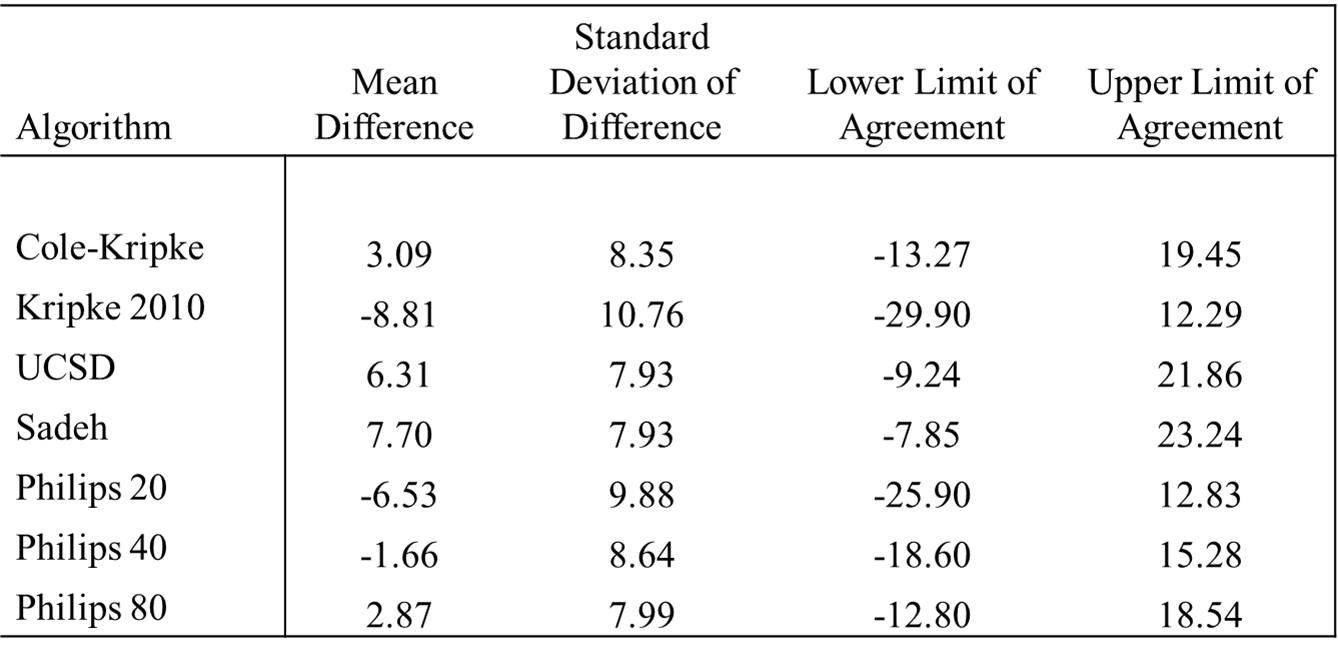


**Table S8**

Mean difference statistics sleep efficacy (SE) for RLS subgroup rescored algorithm
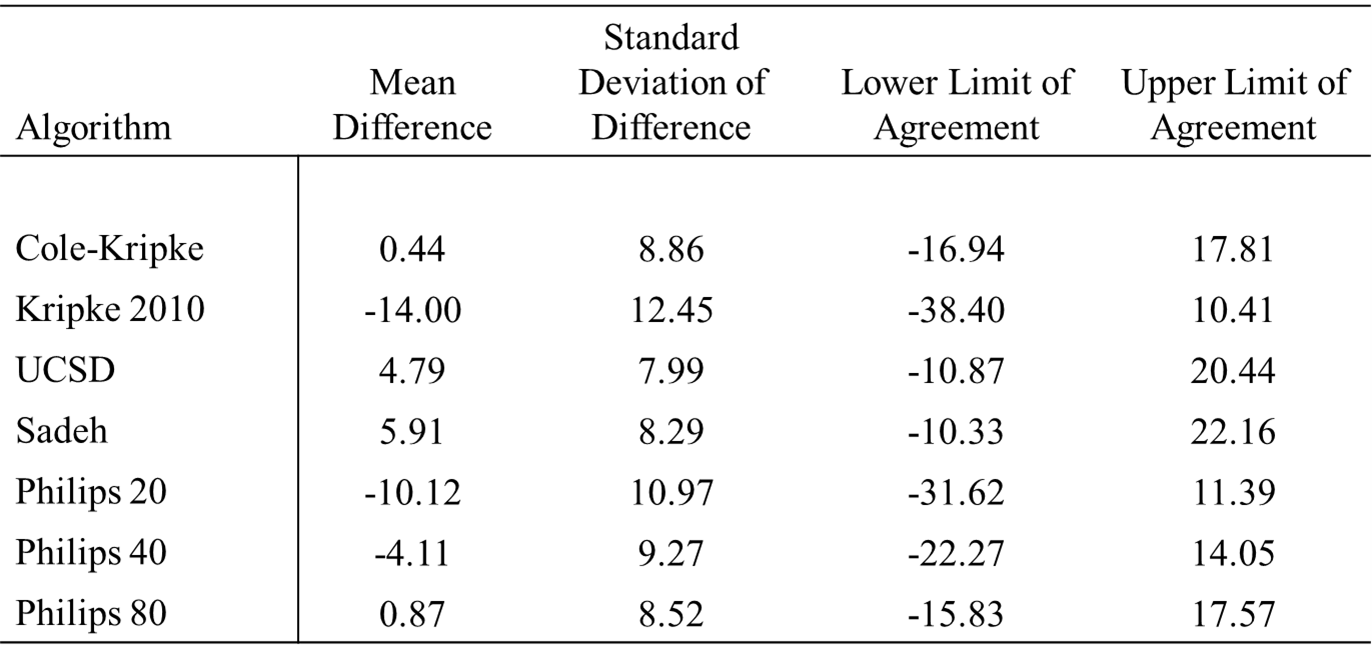


### Bland-Altman Distributions Total Sleep Time

For the RLS subpopulation, all algorithms demonstrate some mean difference between the algorithm estimates and PSG measures of total sleep time. The Philips Algorithm with a threshold of 40 had minimal difference. The Bland Altman distribution demonstrated some systematic bias. However, the points were relatively randomly distributed i.e., did not show a specific distribution pattern. There were only a few outliers, and we expect these would not significantly impact results. The estimates do not appear to get worse as total sleep time decreased or increased. With respect to specific algorithms, the Philips threshold 80, Cole-Kripke, UCSD, and Sadeh all overestimated total sleep time. While Philips threshold 40, Philips threshold 20, Kripke 2010 and underestimate total sleep time.

Rescoring resulted similar results with minor, no improvements, or larger discrepancies in mean difference and distributions. All algorithms demonstrate some mean difference between the algorithm estimates and PSG measures of total sleep time. The Cole-Kripke algorithm had minimal difference. The Bland Altman distribution demonstrated some systematic bias. However, the points were relatively randomly distributed i.e., did not show a specific distribution pattern. There were only a few outliers, and we expect these would not significantly impact results. The estimates do not appear to get worse as total sleep time decreased or increased. With respect to specific algorithms, Cole-Kripke, Philips threshold 80, UCSD, and Sadeh all overestimated total sleep time. While the Philips threshold 40, Philips threshold 20 and Kripke 2010 underestimate total sleep time.

**Figure S9**

Mean difference statistics total sleep time (TST) for RLS subgroup non-rescored algorithms.^a^


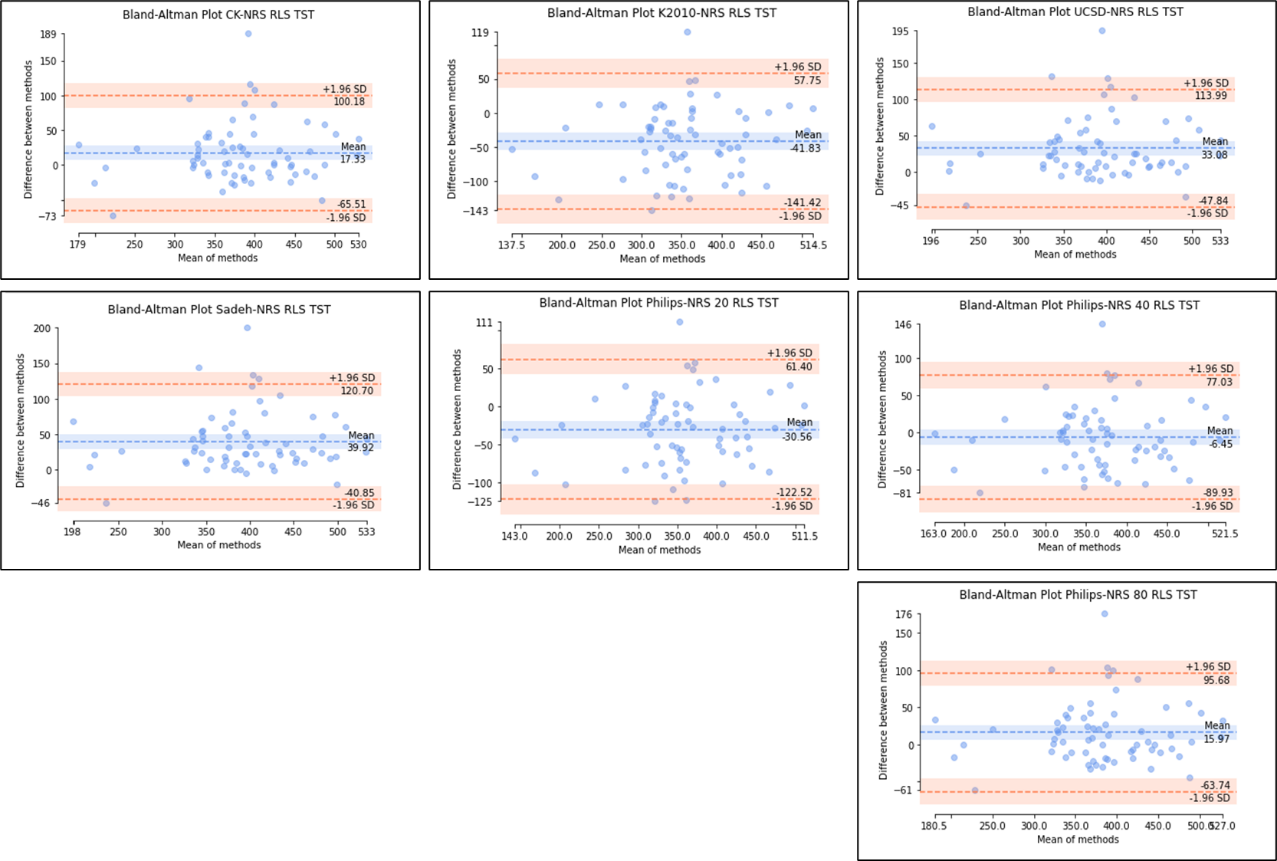


^a. Plots represent the Bland Altman distributions of non-rescored actigraphy algorithms in comparison to polysomnography (PSG; ground truth) for Total Sleep Time (TST) for the RLS subgroup. The y-axis represents the difference between methods while the x-axis represents the mean of methods. The mean difference, standard deviation of the difference, lower and upper limits of agreement (95%) are listed below each respective graph. Each graph showcases the limits in the orange dashed lights while the blue dashed line represents the mean difference. Abbreviated names for each algorithm are as follows: CK = Cole Kripke, K2010 = Kripke 2010, UCSD, Sadeh, and Philips.^

**Figure S10**

Mean difference statistics total sleep time (TST) for RLS subgroup rescored algorithms.^a^


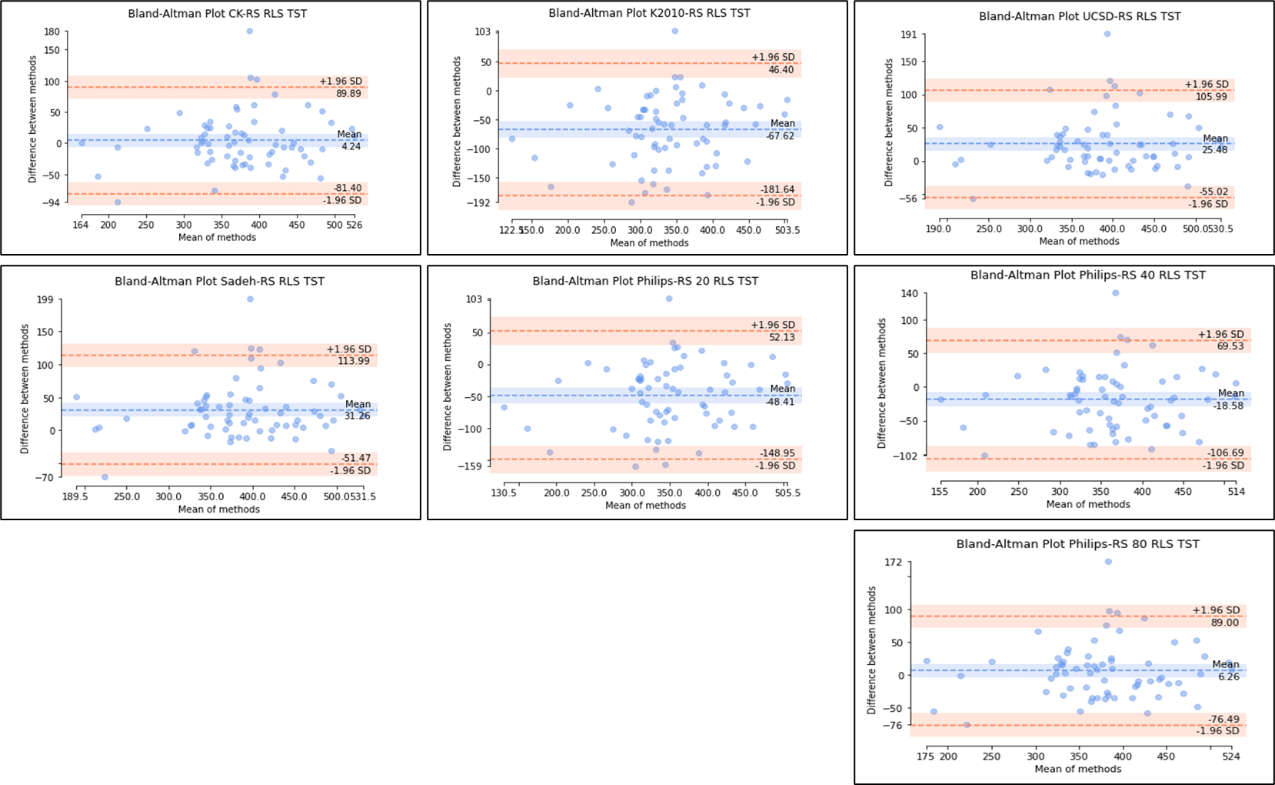


^a. Plots represent the Bland Altman distributions of rescored actigraphy algorithms in comparison to polysomnography (PSG; ground truth) for Total Sleep Time (TST) for the RLS subgroup. The y-axis represents the difference between methods while the x-axis represents the mean of methods. The mean difference, standard deviation of the difference, lower and upper limits of agreement (95%) are listed below each respective graph. Each graph showcases the limits in the orange dashed lights while the blue dashed line represents the mean difference. Abbreviated names for each algorithm are as follows: CK = Cole Kripke, K2010 = Kripke 2010, UCSD, Sadeh, and Philips.^

**Table S9**

Mean difference statistics total sleep time (TST) for RLS subgroup non-rescored algorithms.


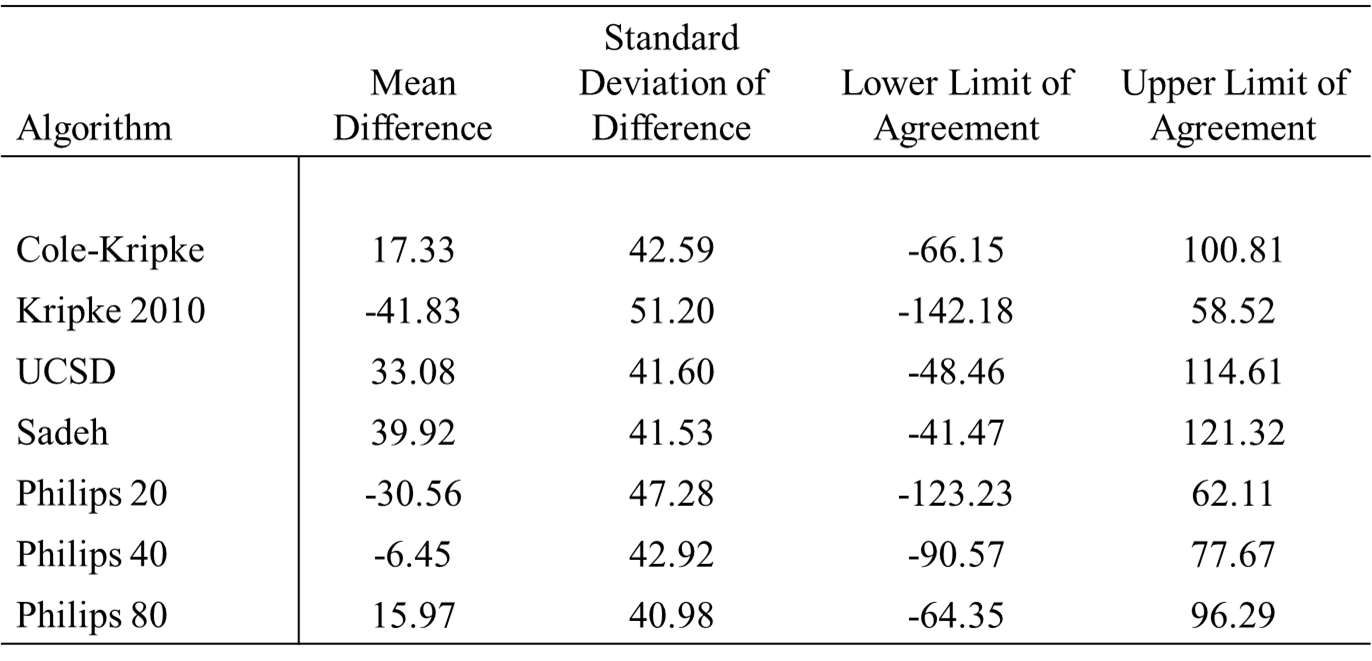


**Table S10**

Mean difference statistics total sleep time (TST) for RLS subgroup rescored algorithms.


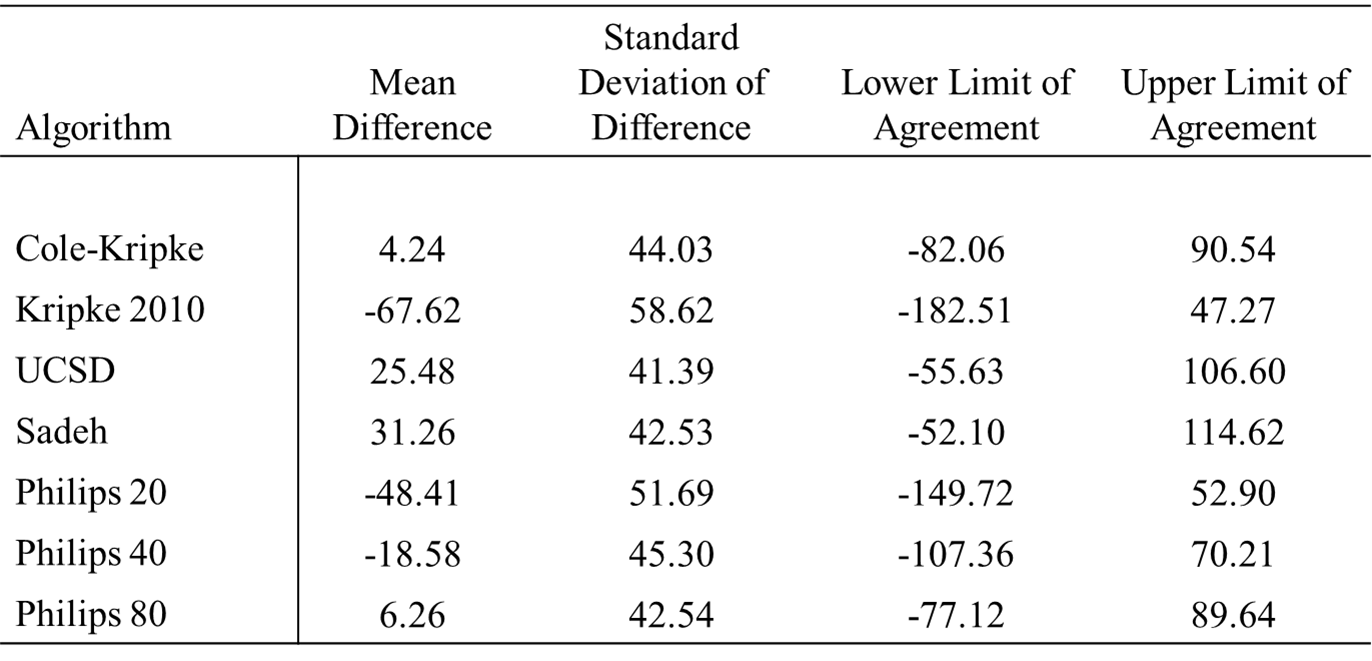


### Bland-Altman Distributions Wake After Sleep Onset

For the RLS subpopulation, all algorithms demonstrate some mean difference between the algorithm estimates and PSG measures of WASO. The Philips Algorithm with a threshold of 20 had minimal difference. The Bland Altman distribution demonstrate clear systematic bias. The Cole-Kripke , UCSD, Sadeh, and Philips Threshold 80 demonstrate clear heteroscedasticity. While the Kripke 2010, Philips threshold 20 and 40 demonstrated some indication of heteroscedasticity as the spread of points increased relative to WASO. That is, WASO estimates were better at lower WASO averages and become considerably worse as WASO increased. There were only a few outliers, and we expect these would not significantly impact results. The Philips threshold 40, Philips threshold 80, Cole-Kripke, UCSD, and Sadeh all underestimated WASO. While Philips threshold 20 and Kripke 2010 overestimated total sleep WASO.

Rescoring resulted similar results with minor, no improvements, or larger discrepancies in mean difference and distributions. All algorithms demonstrate some mean difference between the algorithm estimates and PSG measures of WASO. The Philips Algorithm with a threshold of 20 had the smallest difference. The Bland Altman distribution demonstrate clear systematic bias. The Cole-Kripke , UCSD, Sadeh, and Philips Threshold 80 demonstrated clear heteroscedasticity. While the Kripke 2010, Philips threshold 20 and 40 algorithms demonstrated some heteroscedasticity. That is, WASO estimates were better at lower WASO averages and become considerably worse as WASO increased. There were only a few outliers, and we expect these would not significantly impact results. The Philips threshold 40, Philips threshold 80, Cole-Kripke, UCSD, and Sadeh all underestimated WASO. While Philips threshold 20 and Kripke 2010 overestimated total sleep WASO.

**Figure S11**

Mean difference statistics wake after sleep onset (WASO) for RLS subgroup non-rescored algorithms.^a^


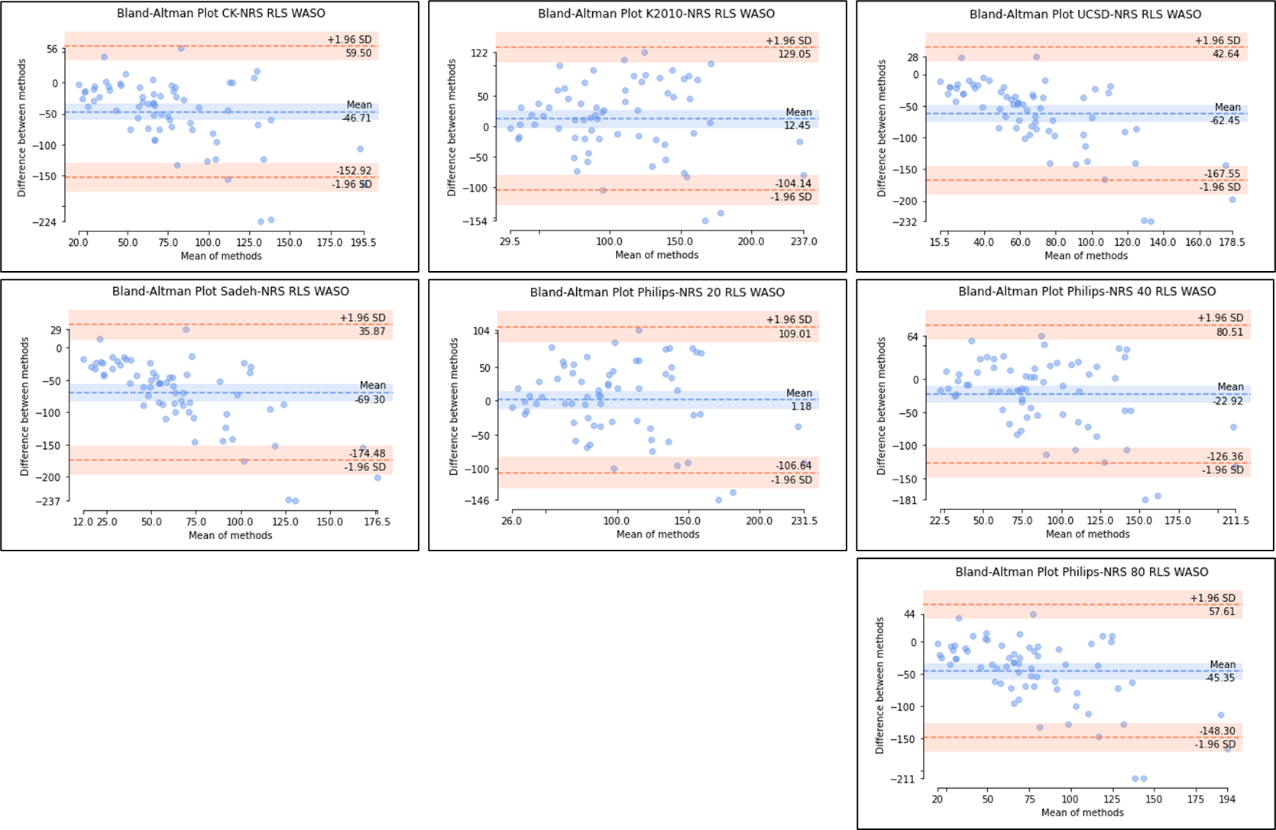


^a. Plots represent the Bland Altman distributions of non-rescored actigraphy algorithms in comparison to polysomnography (PSG; ground truth) for Total Wake After Sleep Onset (WASO) for the RLS subgroup. The y-axis represents the difference between methods while the x-axis represents the mean of methods. The mean difference, standard deviation of the difference, lower and upper limits of agreement (95%) are listed below each respective graph. Each graph showcases the limits in the orange dashed lights while the blue dashed line represents the mean difference. Abbreviated names for each algorithm are as follows: CK = Cole Kripke, K2010 = Kripke 2010, UCSD, Sadeh, and Philips.^

**Figure S12**

Mean difference statistics wake after sleep onset (WASO) for RLS subgroup rescored algorithms.^a^


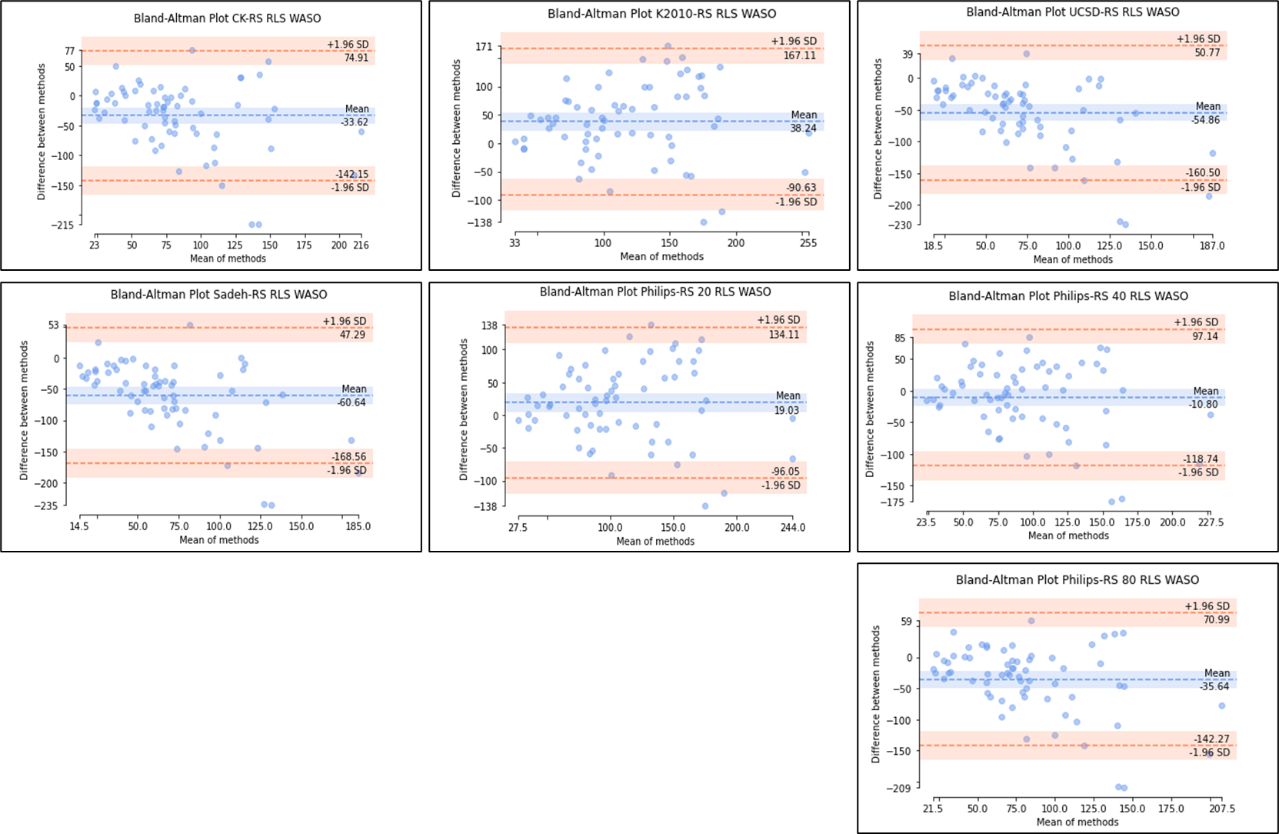


^a. Plots represent the Bland Altman distributions of rescored actigraphy algorithms in comparison to polysomnography (PSG; ground truth) for Total Wake After Sleep Onset (WASO) for the RLS subgroup. The y-axis represents the difference between methods while the x-axis represents the mean of methods. The mean difference, standard deviation of the difference, lower and upper limits of agreement (95%) are listed below each respective graph. Each graph showcases the limits in the orange dashed lights while the blue dashed line represents the mean difference. Abbreviated names for each algorithm are as follows: CK = Cole Kripke, K2010 = Kripke 2010, UCSD, Sadeh, and Philips.^

**Table S11**

Mean difference statistics wake after sleep onset (WASO) for RLS subgroup non-rescored algorithms.


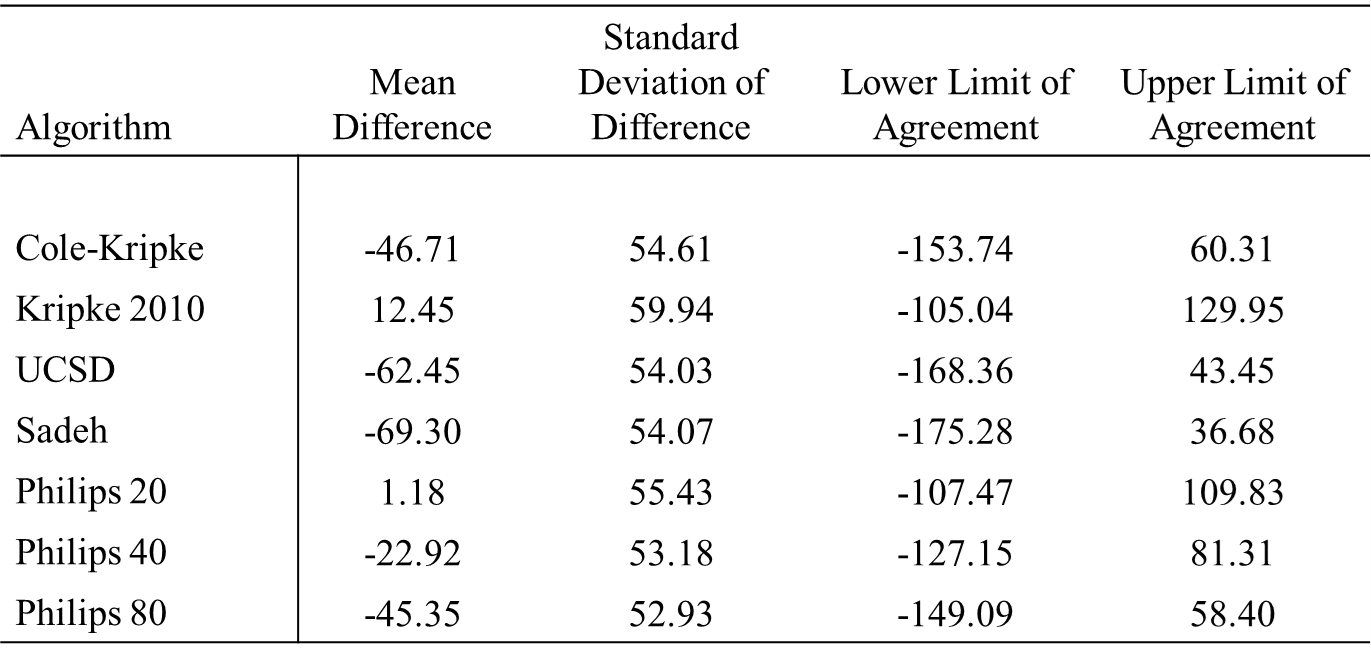


**Table S12**

Mean difference statistics wake after sleep onset (WASO) for RLS Syndrome subgroup rescored algorithms.


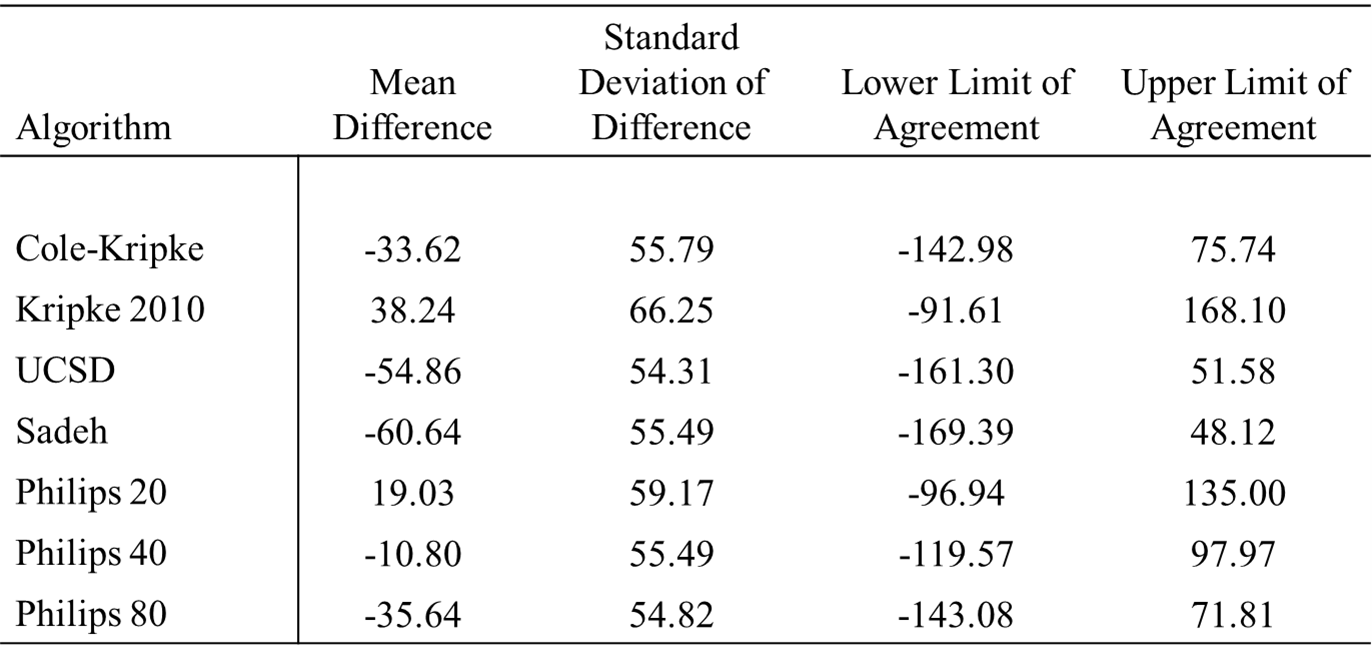


## Apnea Subgroup

### Bland-Altman Distributions Sleep Efficiency

For the Apnea subpopulation, all algorithms demonstrated some mean difference between the algorithm estimates and PSG measures of sleep efficacy. The Philips Algorithm with a threshold of 40 which had minimal difference. The Bland Altman distribution demonstrated some systematic bias. The Cole-Kripke , UCSD, Sadeh, and Philips Threshold 80 demonstrate some heteroscedasticity. While the Kripke 2010, Philips threshold 20 and 40 demonstrated some indication of heteroscedasticity as the spread of points increased as SE decreased. There were only a few outliers, and we expect these would not significantly impact results. That is, sleep efficacy estimates were better at higher sleep efficacy averages and become considerably worse as sleep efficacy decreases. The Philips threshold 80, Cole-Kriple, UCSD, and Sadeh all overestimated sleep efficacy. While Philips threshold 40, Philips threshold 20 and Kripke 2010 underestimated sleep efficacy.

Rescoring resulted similar results with minor, no improvements, or larger discrepancies in mean difference and distributions. All algorithms demonstrated some mean difference between the algorithm estimates and PSG measures of sleep efficacy. The Cole-Kripke and Philips Algorithm with a threshold of 80 which had minimal difference. The Bland Altman distribution demonstrated some systematic bias. All algorithms demonstrated some heteroscedasticity. There were only a few outliers, and we expect these would not significantly impact results. The estimates do appear to get worse as sleep efficiency decreased. The Cole-Kripke, Philips threshold 80, UCSD, and Sadeh algorithms all overestimated sleep efficacy. While Philips threshold 40, Philips threshold 20, and Kripke 2010 underestimated sleep efficacy.

**Figure S13**

Mean difference statistics sleep efficacy (SE) for Apnea subgroup non-rescored algorithms.^a^


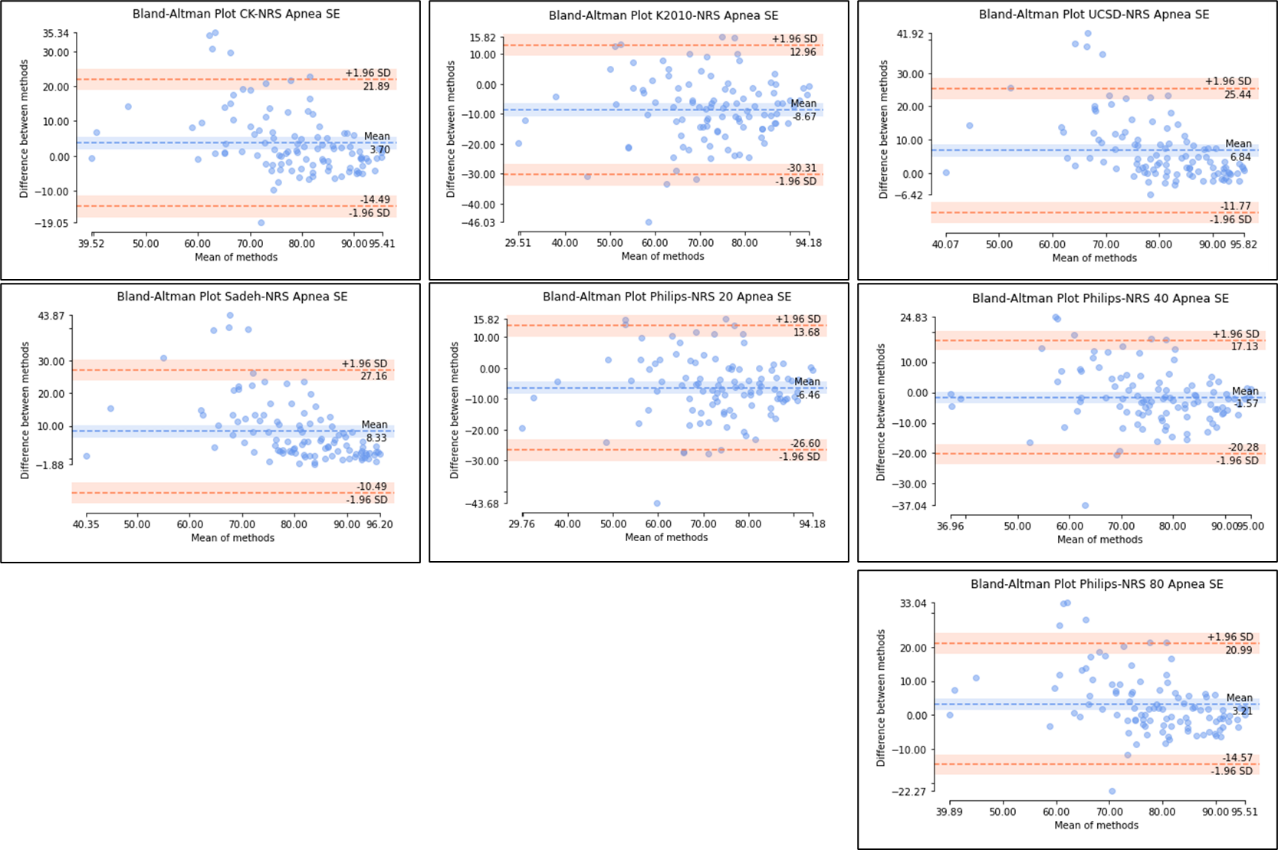


^a. Plots represent the Bland Altman distributions of non-rescored actigraphy algorithms in comparison to polysomnography (PSG; ground truth) for Sleep Efficacy (SE) for the Apnea subgroup. The y-axis represents the difference between methods while the x-axis represents the mean of methods. The mean difference, standard deviation of the difference, lower and upper limits of agreement (95%) are listed below each respective graph. Each graph showcases the limits in the orange dashed lights while the blue dashed line represents the mean difference. Abbreviated names for each algorithm are as follows: CK = Cole Kripke, K2010 = Kripke 2010, UCSD, Sadeh, and Philips.^

**Figure S14**

Mean difference statistics sleep efficacy (SE) for Apnea subgroup rescored algorithms.^a^


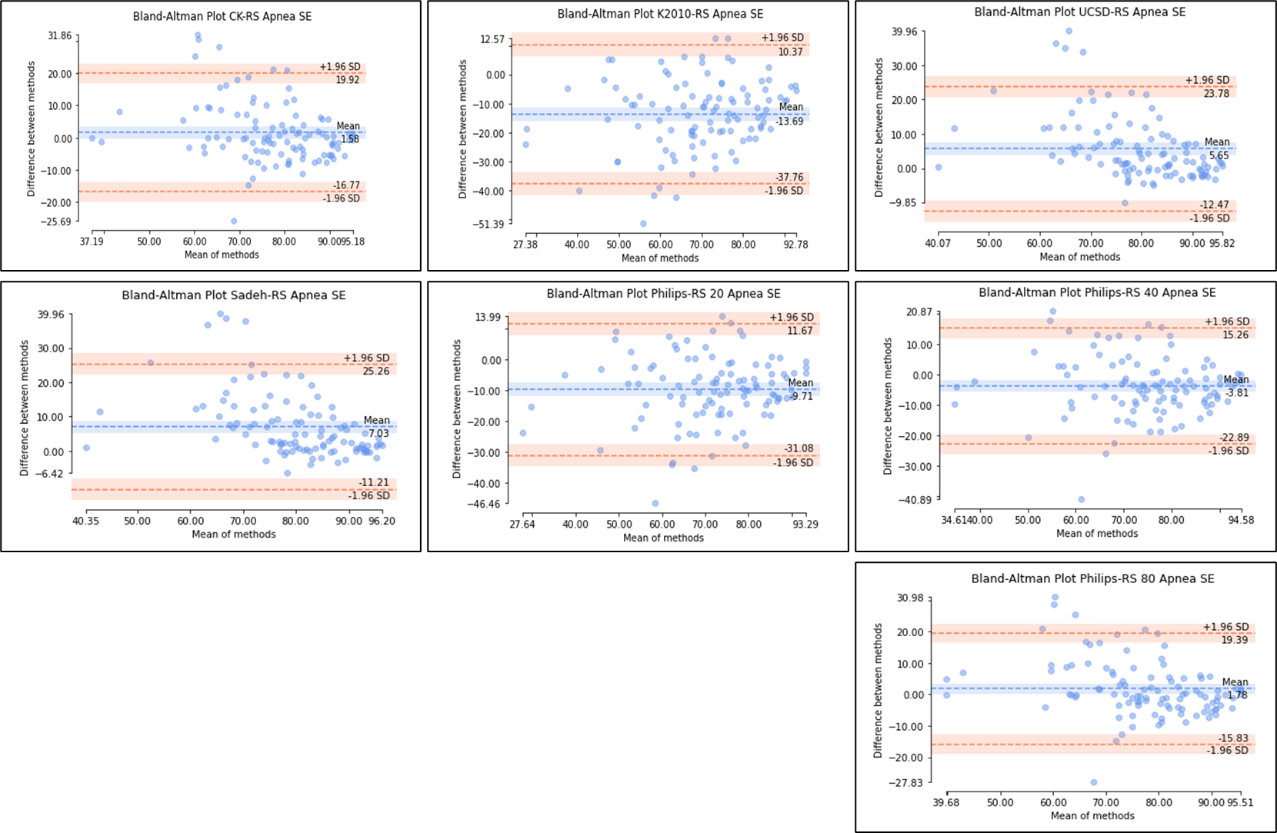


^a. Plots represent the Bland Altman distributions of rescored actigraphy algorithms in comparison to polysomnography (PSG; ground truth) for Sleep Efficacy (SE) for the Apnea subgroup. The y-axis represents the difference between methods while the x-axis represents the mean of methods. The mean difference, standard deviation of the difference, lower and upper limits of agreement (95%) are listed below each respective graph. Each graph showcases the limits in the orange dashed lights while the blue dashed line represents the mean difference. Abbreviated names for each algorithm are as follows: CK = Cole Kripke, K2010 = Kripke 2010, UCSD, Sadeh, Philips.^

**Table S13**

Mean difference statistics sleep efficacy (SE) for Apnea subgroup non-rescored algorithms.


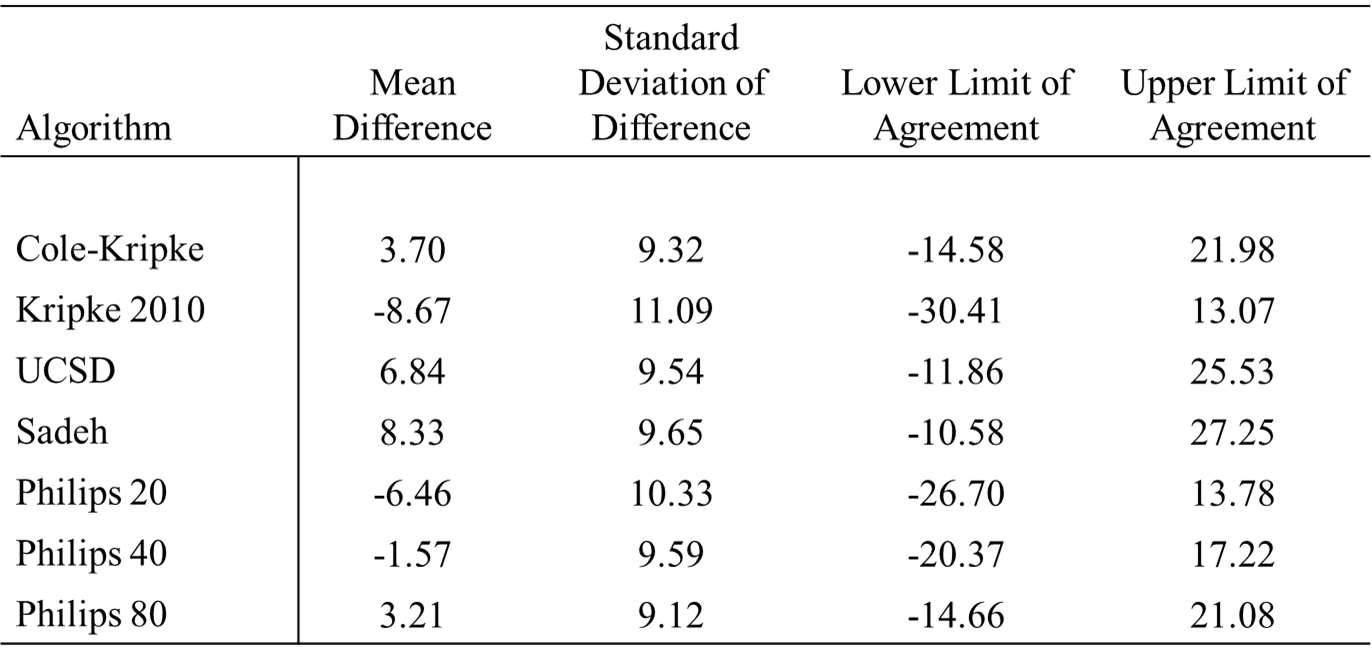


**Table S14**

Mean difference statistics sleep efficacy (SE) for Apnea subgroup rescored algorithms.


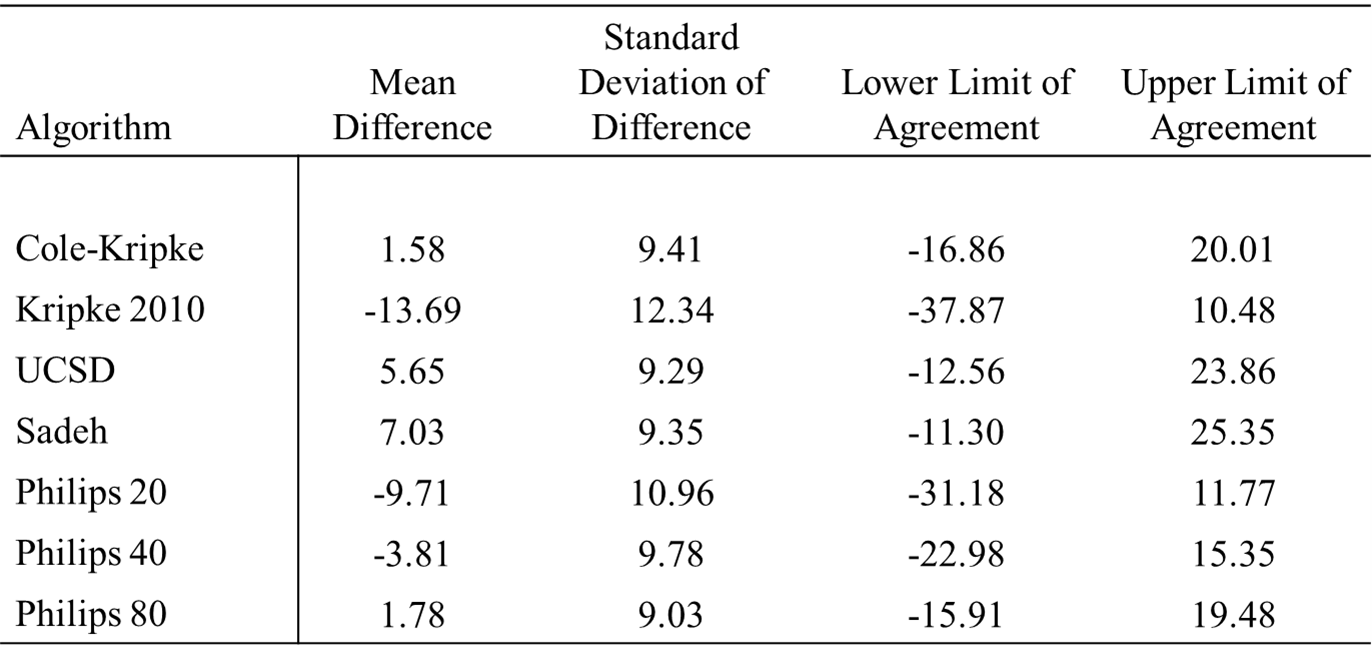


### Bland-Altman Distributions Total Sleep Time

For the Apnea subpopulation, all algorithms demonstrate some mean difference between the algorithm estimates and PSG measures of total sleep time. The Philips Algorithm with a threshold of 40 had minimal difference. The Bland Altman distribution demonstrated some systematic bias. However, the points were relatively randomly distributed i.e., did not show a specific distribution pattern. There were only a few outliers, and we expect these would not significantly impact results. The estimates do not appear to get worse as total sleep time decreased or increased. With respect to specific algorithms, the Philips threshold 80, Cole-Kriple, UCSD, and Sadeh all overestimated total sleep time. While Philips threshold 40, Philips threshold 20 and Kripke 2010 underestimated total sleep time.

Rescoring resulted similar results with minor, no improvements, or larger discrepancies in mean difference and distributions. All algorithms demonstrate some mean difference between the algorithm estimates and PSG measures of total sleep time. The Cole-Kripke and Philips Algorithm with a threshold of 80 had the smallest difference. The Bland Altman distribution demonstrated some systematic bias. However, the points were relatively randomly distributed i.e., did not show a specific distribution pattern. There were only a few outliers, and we expect these would not significantly impact results. The estimates do not appear to get worse as total sleep time decreased or increased. With respect to specific algorithms, the Cole-Kripke, Philips threshold 80, UCSD, and Sadeh all overestimated total sleep time. While Philips threshold 40, Philips threshold 20, and Kripke 2010 underestimate total sleep time.

**Figure S15**

Mean difference statistics total sleep time (TST) for Apnea subgroup non-rescored algorithms.^a^


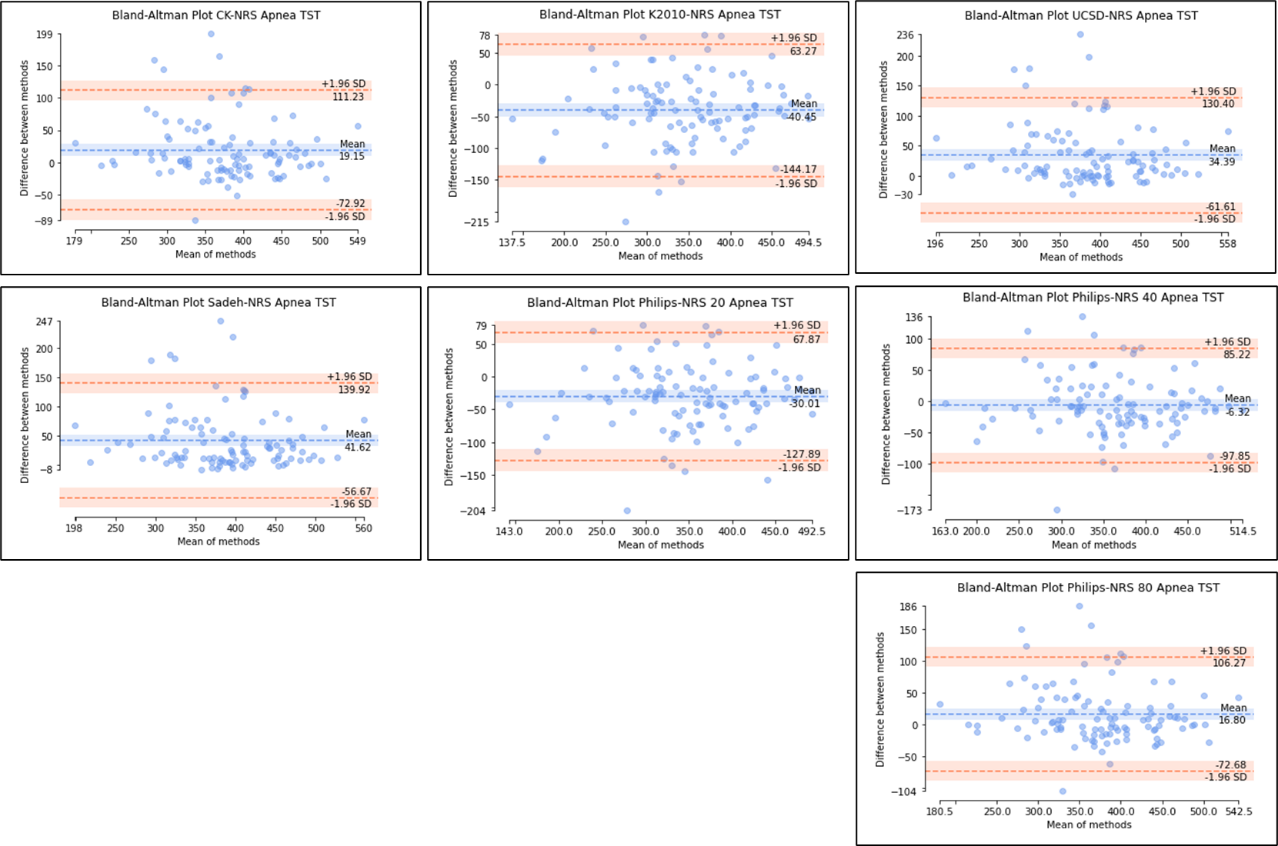


^a. Plots represent the Bland Altman distributions of non-rescored actigraphy algorithms in comparison to polysomnography (PSG; ground truth) for Total Sleep Time (TST) for the Apnea subgroup. The y-axis represents the difference between methods while the x-axis represents the mean of methods. The mean difference, standard deviation of the difference, lower and upper limits of agreement (95%) are listed below each respective graph. Each graph showcases the limits in the orange dashed lights while the blue dashed line represents the mean difference. Abbreviated names for each algorithm are as follows: CK = Cole Kripke, K2010 = Kripke 2010, UCSD, Sadeh, and Philips.^

**Figure S16**

Mean difference statistics total sleep time (TST) for Apnea subgroup rescored algorithms.^a^


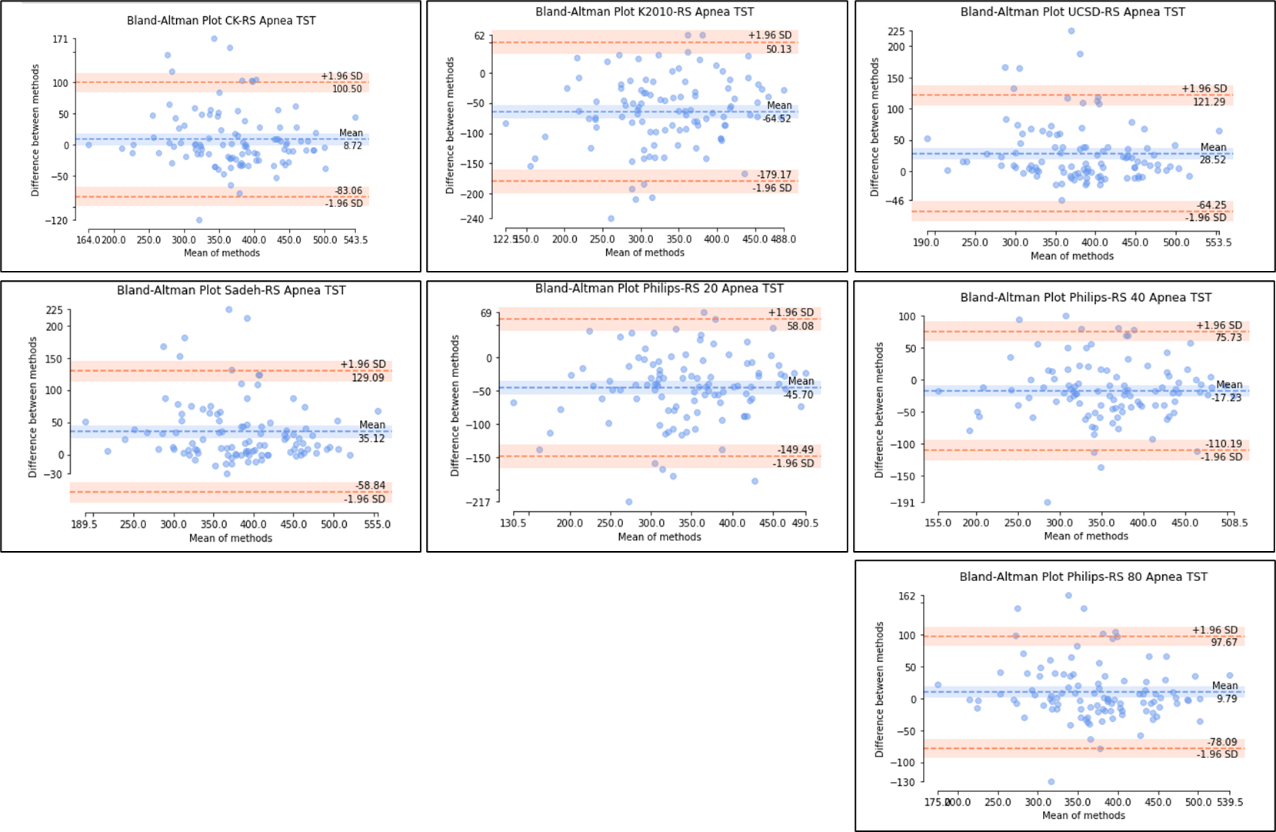


^a. Plots represent the Bland Altman distributions of rescored actigraphy algorithms in comparison to polysomnography (PSG; ground truth) for Total Sleep Time (TST) for the Apnea subgroup. The y-axis represents the difference between methods while the x-axis represents the mean of methods. The mean difference, standard deviation of the difference, lower and upper limits of agreement (95%) are listed below each respective graph. Each graph showcases the limits in the orange dashed lights while the blue dashed line represents the mean difference. Abbreviated names for each algorithm are as follows: CK = Cole Kripke, K2010 = Kripke 2010, UCSD, Sadeh, and Philips.^

**Table S15**

Mean difference statistics total sleep time (TST) for Apnea subgroup non-rescored algorithms.


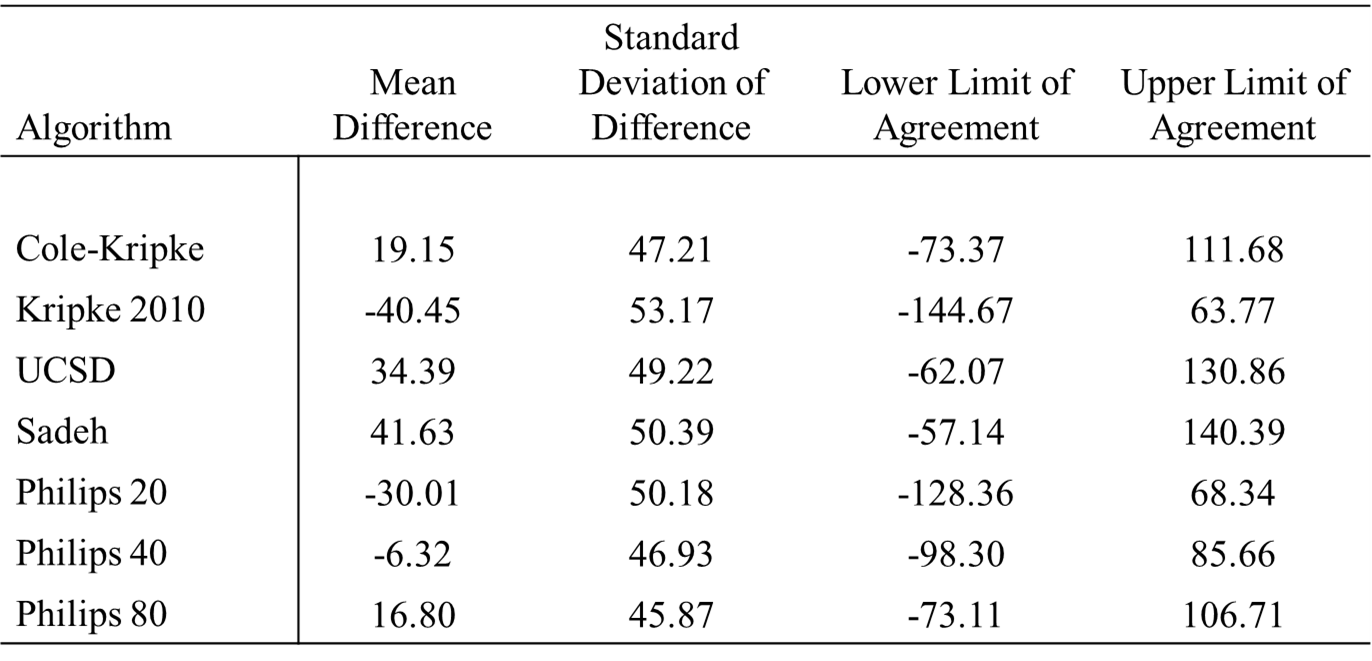


**Table S16**

Mean difference statistics total sleep time (TST) for Apnea subgroup rescored algorithms.


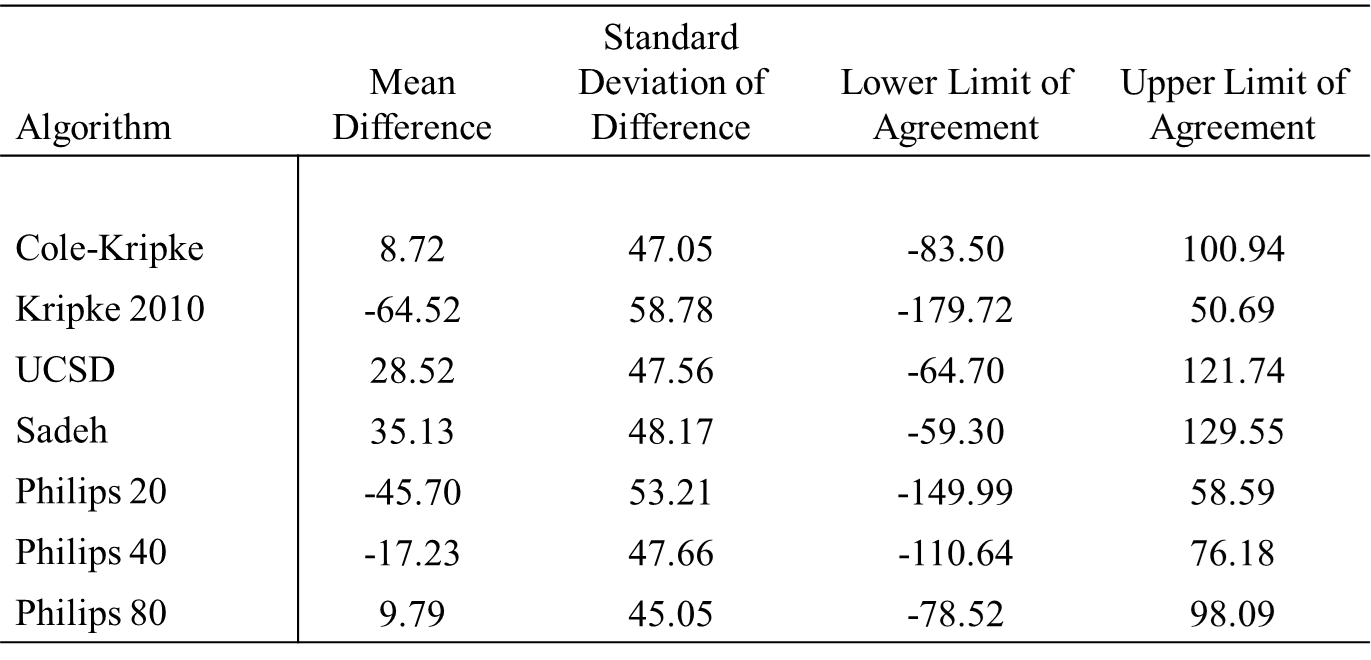


### Bland-Altman Distributions Wake After Sleep Onset

For the Apnea subpopulation, all algorithms demonstrate some mean difference between the algorithm estimates and PSG measures of WASO. The Philips Algorithm with a threshold of 20 had minimal difference. The Bland Altman distribution demonstrate clear systematic bias. The Cole-Kripke, UCSD, Sadeh, and Philips Threshold 80 demonstrate clear heteroscedasticity. While the Kripke 2010, Philips threshold 20 and 40 demonstrated some indication of heteroscedasticity as the spread of points increased relative to WASO. That is, WASO estimates were better at lower WASO averages and become considerably worse as WASO increased There were only a few outliers, and we expect these would not significantly impact results. The Philips threshold 20 and Kripke 2010 algorithms overestimated total sleep WASO. While the Philips threshold 40, Philips threshold 80, Cole-Kripke, UCSD, and Sadeh all underestimated WASO.

Rescoring resulted similar results with minor, no improvements, or larger discrepancies in mean difference and distributions. All algorithms demonstrate some mean difference between the algorithm estimates and PSG measures of WASO. The Philips Algorithm with a threshold of 20 had the smallest difference. The Bland Altman distribution demonstrate clear systematic bias. The all algorithms demonstrate clear heteroscedasticity. That is, WASO estimates were better at lower WASO averages and become considerably worse as WASO increased. There were only a few outliers, and we expect these would not significantly impact results. The Philips threshold 40, Cole-Kripke, Philips threshold 80, UCSD, and Sadeh all underestimated WASO. While Philips threshold 20 and Kripke 2010 overestimated total sleep WASO.

**Figure S17**

Mean difference statistics wake after sleep onset (WASO) for Apnea subgroup non-rescored algorithms.^a^


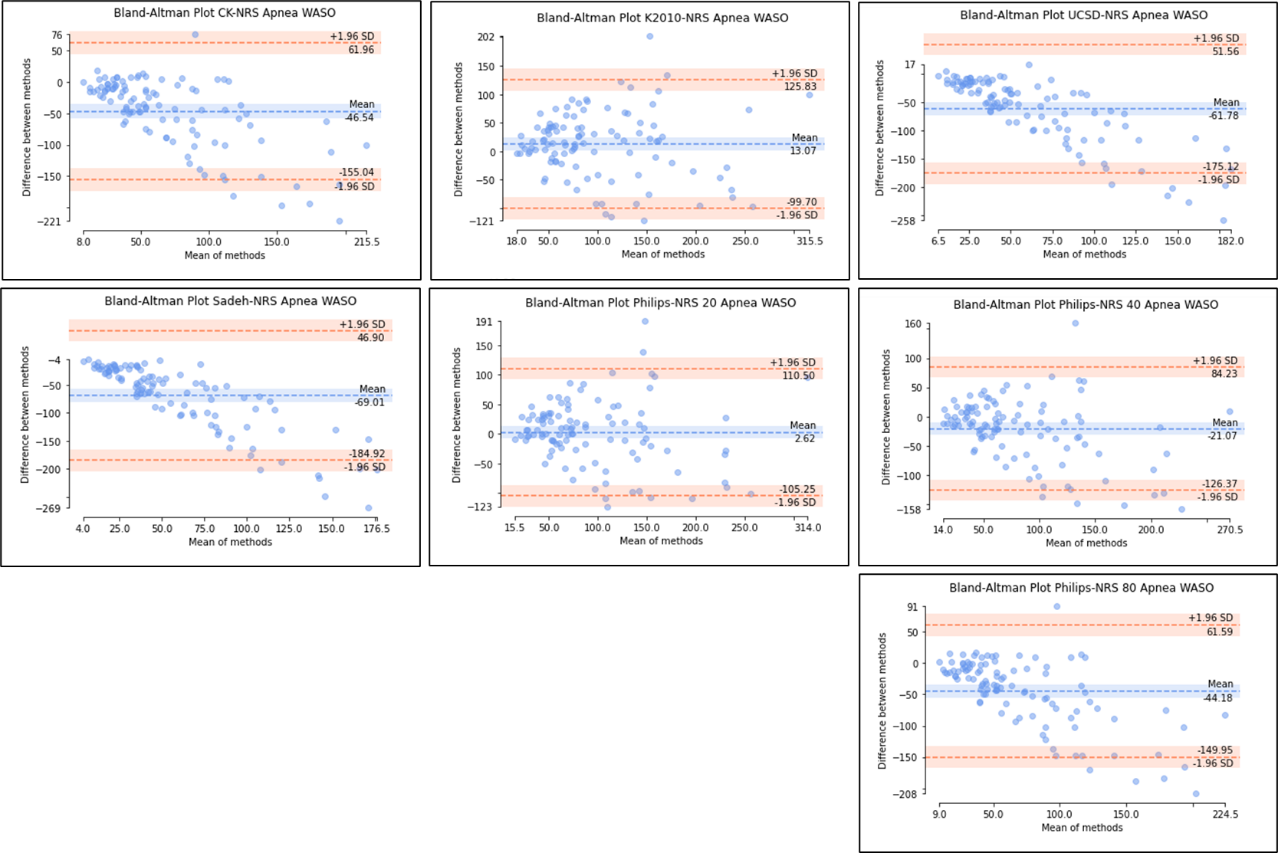


^a. Plots represent the Bland Altman distributions of non-rescored actigraphy algorithms in comparison to polysomnography (PSG; ground truth) for Wake After Sleep Onset (WASO) for the Apnea Subgroup. The y-axis represents the difference between methods while the x-axis represents the mean of methods. The mean difference, standard deviation of the difference, lower and upper limits of agreement (95%) are listed below each respective graph. Each graph showcases the limits in the orange dashed lights while the blue dashed line represents the mean difference. Abbreviated names for each algorithm are as follows: CK = Cole Kripke, K2010 = Kripke 2010, UCSD, Sadeh, and Philips.^

**Figure S18**

Mean difference statistics wake after sleep onset (WASO) for Apnea subgroup rescored algorithms.^a^


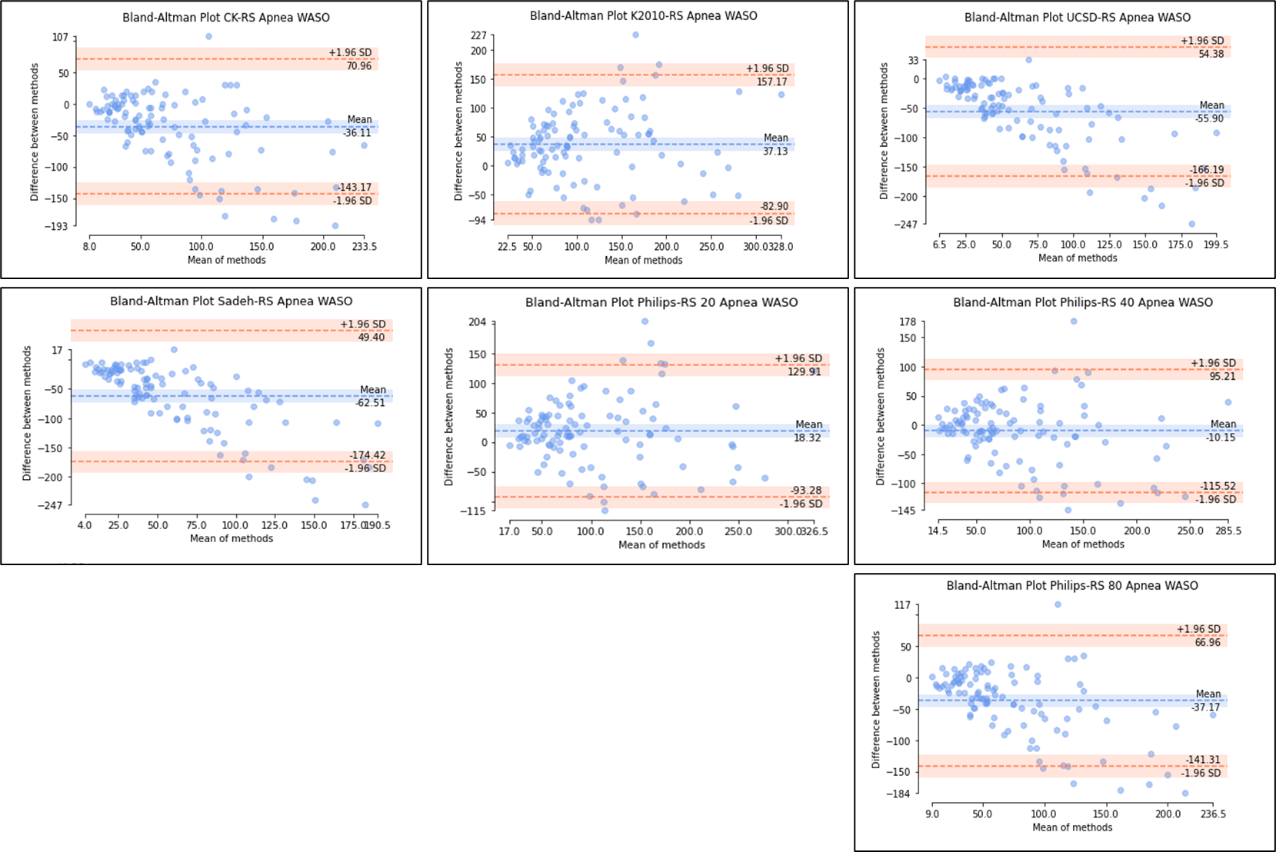


^a. Plots represent the Bland Altman distributions of rescored actigraphy algorithms in comparison to polysomnography (PSG; ground truth) for Wake After Sleep Onset (WASO) for the Apnea Subgroup. The y-axis represents the difference between methods while the x-axis represents the mean of methods. The mean difference, standard deviation of the difference, lower and upper limits of agreement (95%) are listed below each respective graph. Each graph showcases the limits in the orange dashed lights while the blue dashed line represents the mean difference. Abbreviated names for each algorithm are as follows: CK = Cole Kripke, K2010 = Kripke 2010, UCSD, Sadeh, and Philips.^

**Table S17**

Mean difference statistics wake after sleep onset (WASO) for Apnea subgroup non-rescored algorithms.


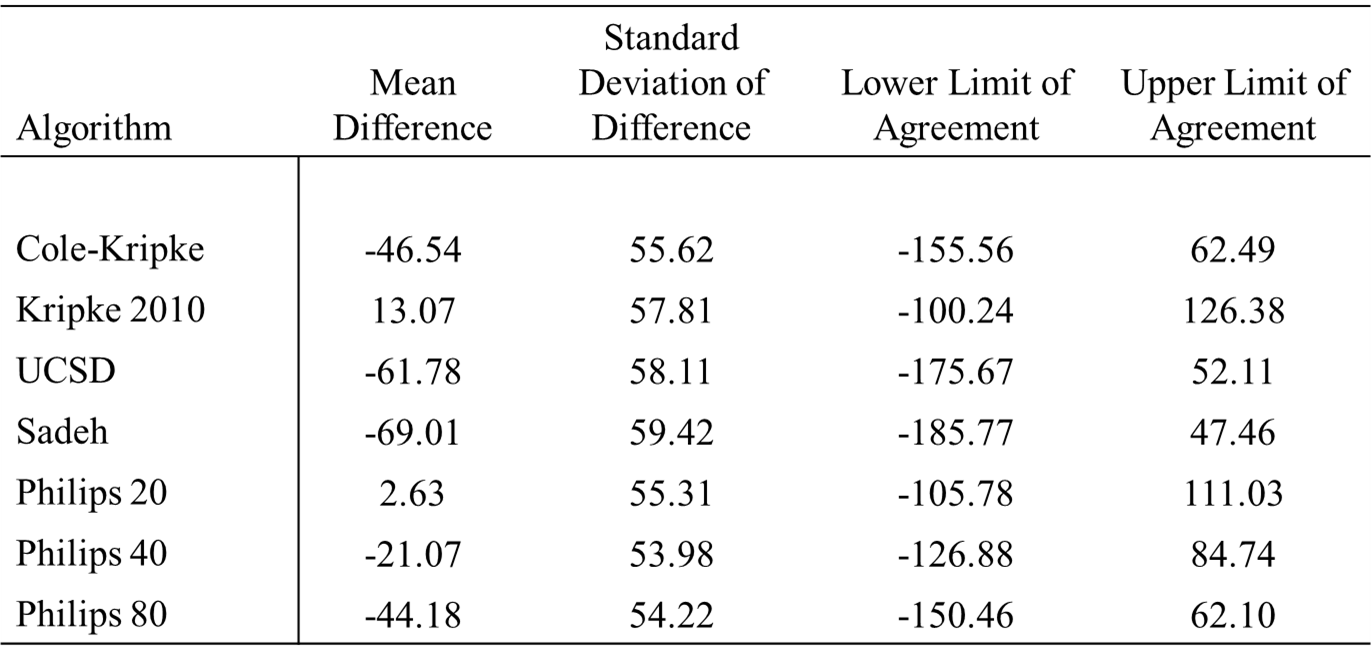


**Table S18**

Mean difference statistics wake after sleep onset (WASO) for Apnea subgroup rescored algorithms.


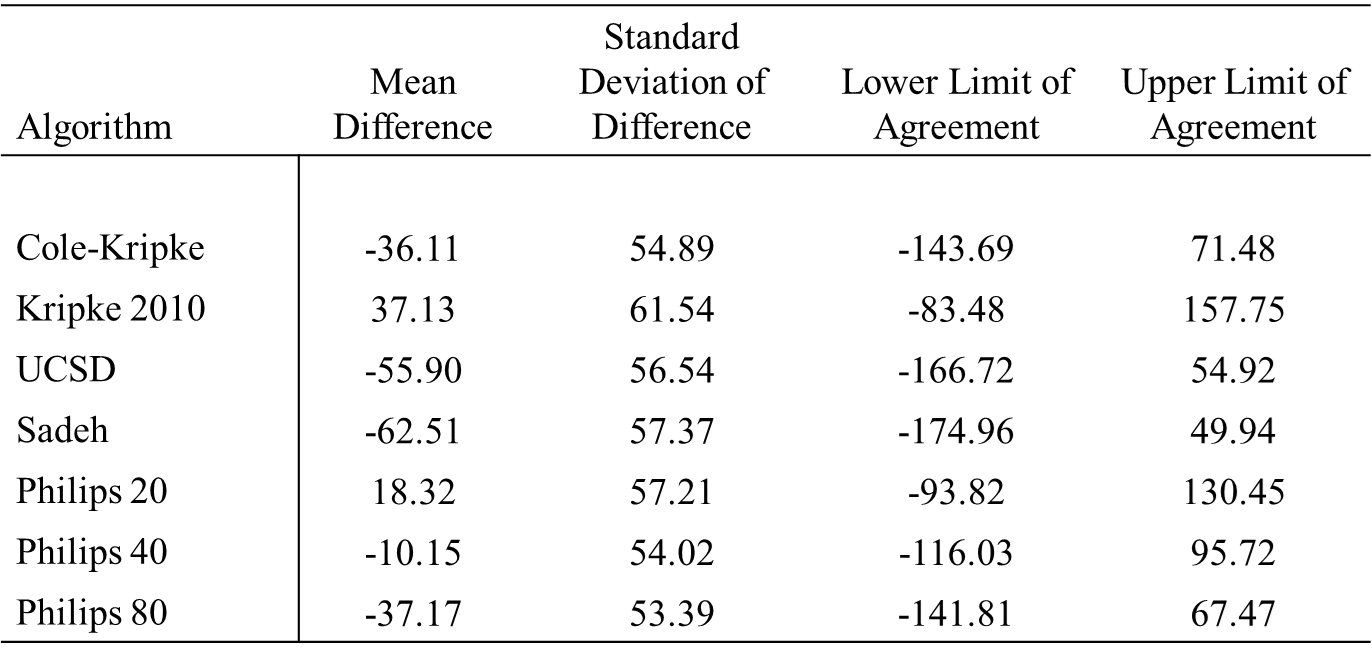


## CPAP Subgroup

### Bland-Altman Distributions Sleep Efficiency

For the CPAP subpopulation, all algorithms demonstrated some mean difference between the algorithm estimates and PSG measures of sleep efficacy. The Philips Algorithm with a threshold of 40 which had minimal difference. The Bland Altman distribution demonstrated some systematic bias. All algorithms also demonstrated some indication of heteroscedasticity as the spread of points increased as SE decreased. There were only a few outliers, and we expect these would not significantly impact results. The estimates do appear to get worse as sleep efficiency decreased. The Philips threshold 80, Cole-Kripke, Sadeh, and UCSD all overestimated sleep efficacy. While Philips threshold 40, Philips threshold 20 and Kripke 2010 underestimated sleep efficacy.

Rescoring resulted similar results with minor, no improvements, or larger discrepancies in mean difference and distributions. All algorithms demonstrated some mean difference between the algorithm estimates and PSG measures of sleep efficacy. The Philips Algorithm with a threshold of 80 and Cole-Kripke which had minimal difference. The Bland Altman distribution demonstrated some systematic bias. All algorithms demonstrated some heteroscedasticity. There were only a few outliers, and we expect these would not significantly impact results. The estimates do appear to get worse as sleep efficiency decreased. The Philips threshold 80, Cole-Kripke, UCSD, and Sadeh algorithms all overestimated sleep efficacy. While Philips threshold 40, Philips threshold 20, and Kripke 2010 underestimated sleep efficacy.

**Figure S19**

Mean difference statistics sleep efficacy (SE) for CPAP subgroup non-rescored algorithms.^a^


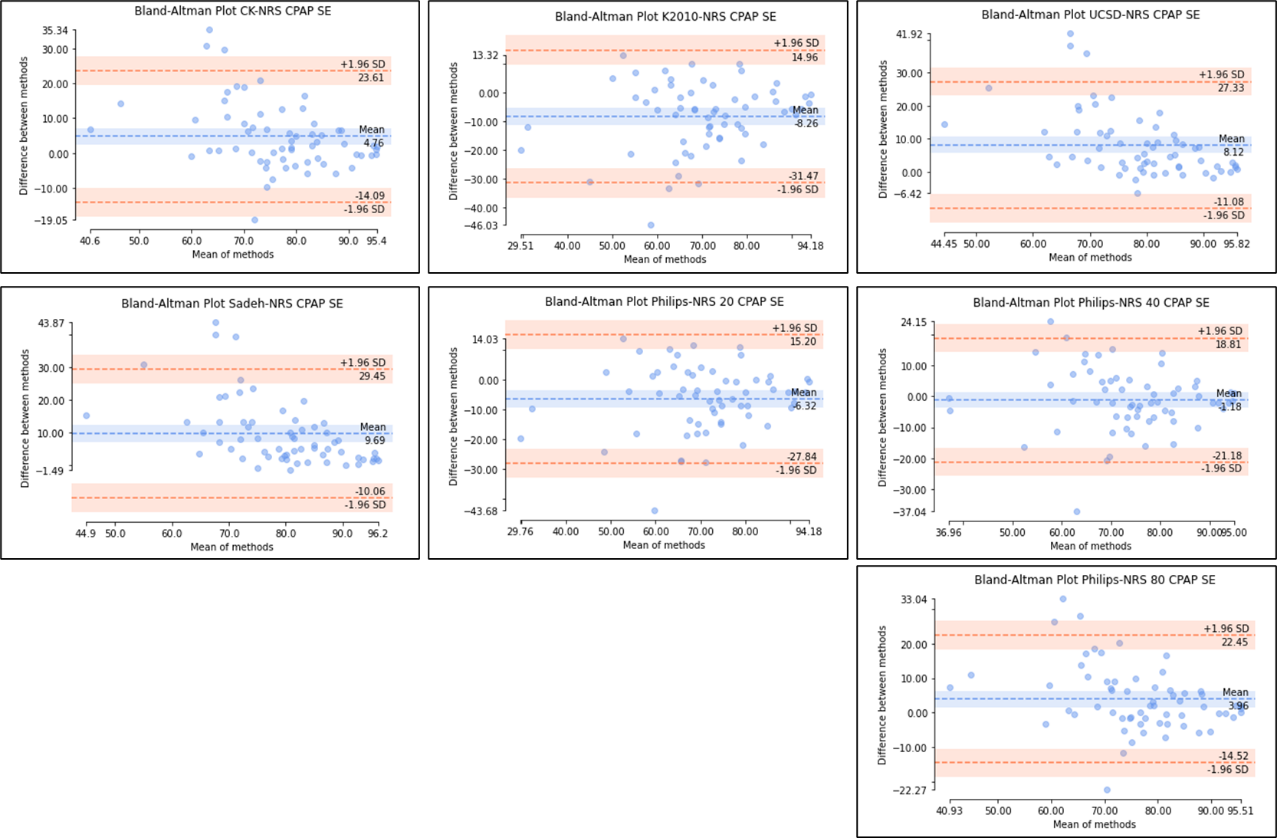


^a. Plots represent the Bland Altman distributions of non-rescored actigraphy algorithms in comparison to polysomnography (PSG; ground truth) for Sleep Efficiency (SE) for the CPAP subgroup. The y-axis represents the difference between methods while the x-axis represents the mean of methods. The mean difference, standard deviation of the difference, lower and upper limits of agreement (95%) are listed below each respective graph. Each graph showcases the limits in the orange dashed lights while the blue dashed line represents the mean difference. Abbreviated names for each algorithm are as follows: CK = Cole Kripke, K2010 = Kripke 2010, UCSD, Sadeh, and Philips^

**Figure S20**

Mean difference statistics sleep efficacy (SE) for CPAP subgroup rescored algorithms.^a^


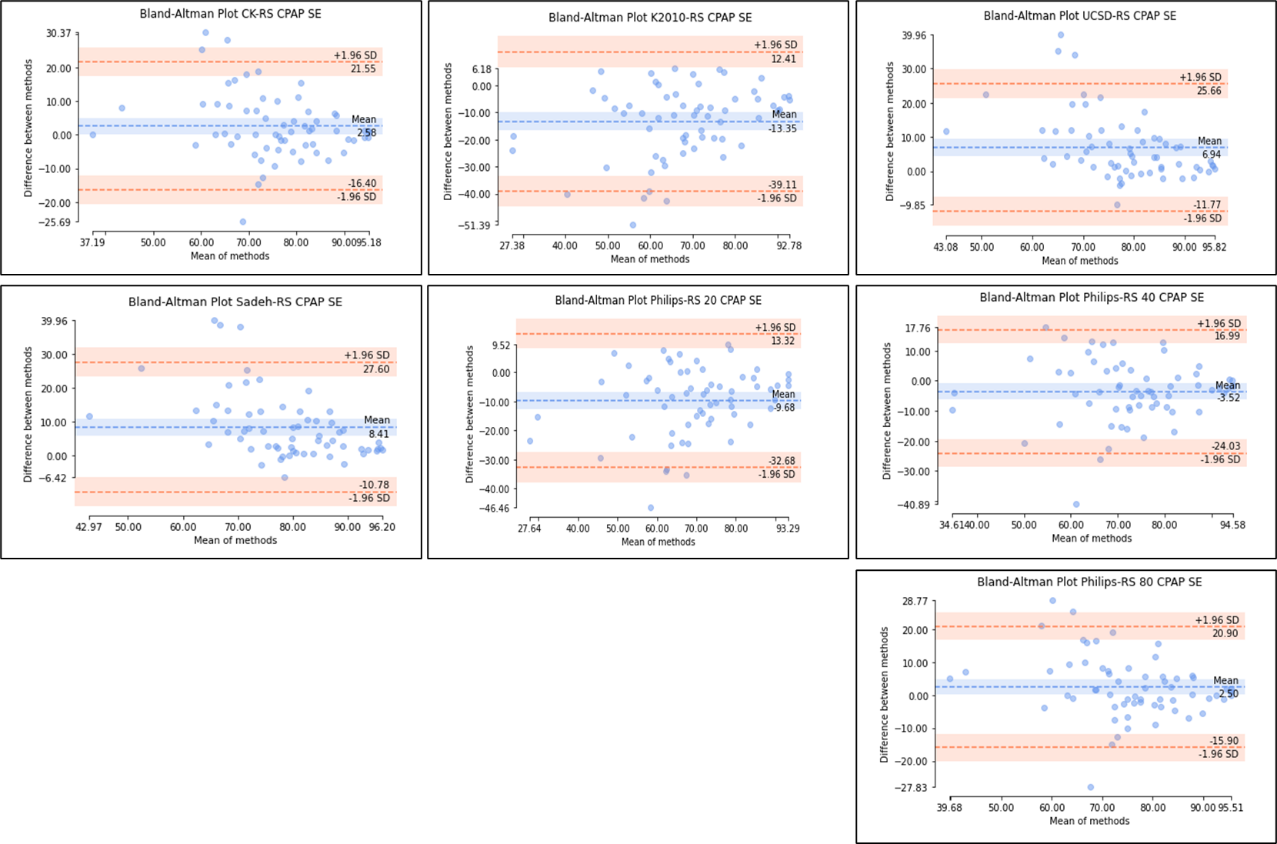


^a. Plots represent the Bland Altman distributions of rescored actigraphy algorithms in comparison to polysomnography (PSG; ground truth) for Sleep Efficiency (SE) for the CPAP subgroup. The y-axis represents the difference between methods while the x-axis represents the mean of methods. The mean difference, standard deviation of the difference, lower and upper limits of agreement (95%) are listed below each respective graph. Each graph showcases the limits in the orange dashed lights while the blue dashed line represents the mean difference. Abbreviated names for each algorithm are as follows: CK = Cole Kripke, K2010 = Kripke 2010, UCSD, Sadeh, and Philips.^

**Table S19**

Mean difference statistics sleep efficacy (SE) for CPAP subgroup non-rescored algorithms.


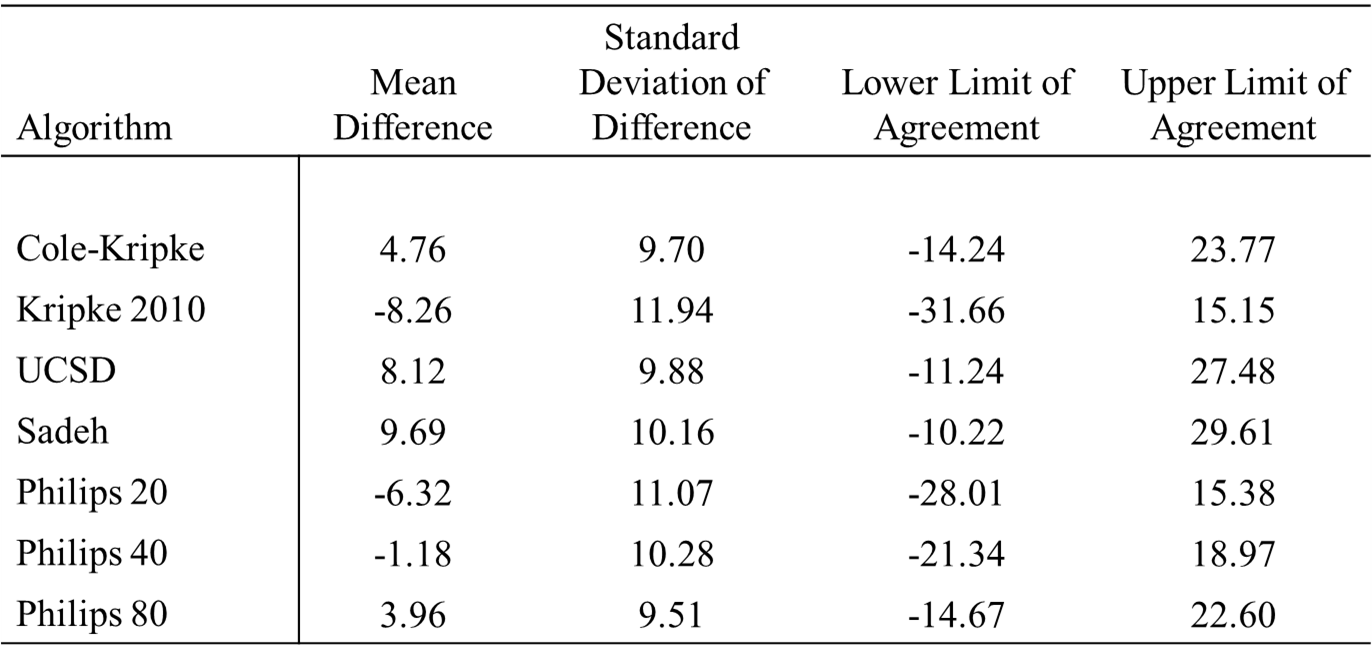


**Table S20**

Mean difference statistics sleep efficacy (SE) for CPAP subgroup rescored algorithms.


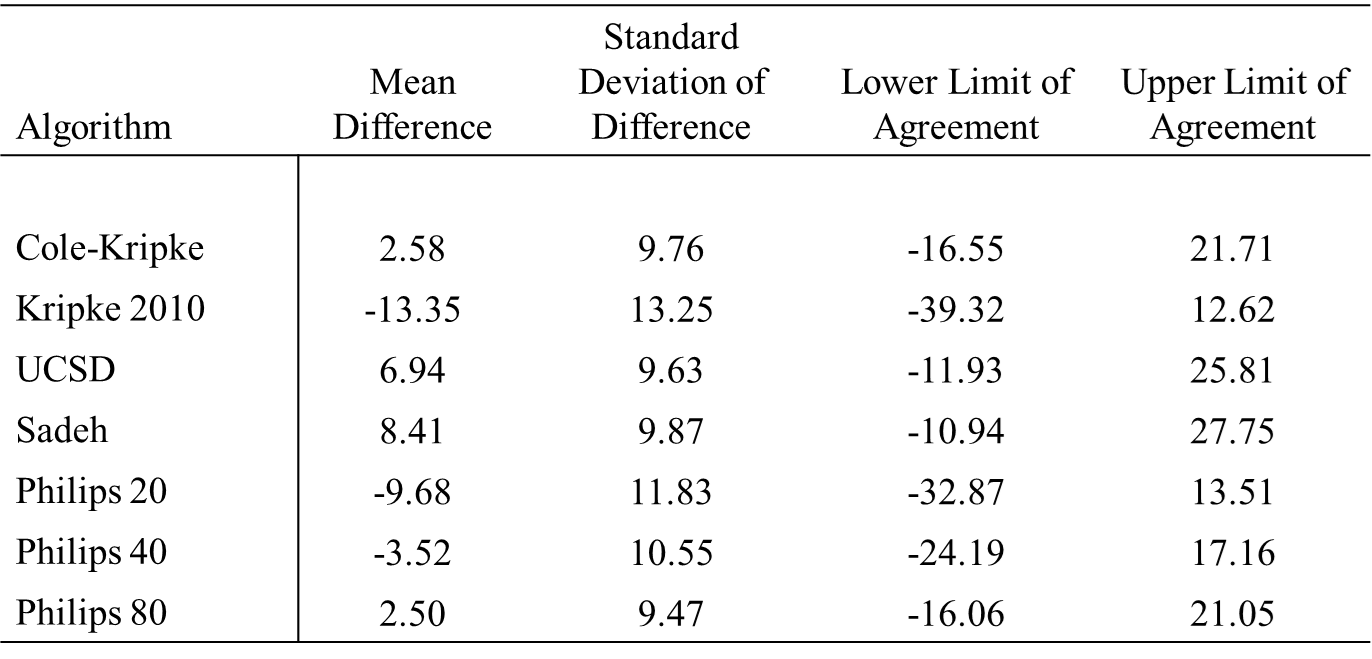


Bland-Altman Distributions Total Sleep Time

For the CPAP subpopulation, all algorithms demonstrate some mean difference between the algorithm estimates and PSG measures of total sleep time. The Philips Algorithm with a threshold of 40 had minimal difference. The Bland Altman distribution demonstrated some systematic bias. However, the points were relatively randomly distributed i.e., did not show a specific distribution pattern. There were only a few outliers, and we expect these would not significantly impact results. The estimates do not appear to get worse as total sleep time decreased or increased. With respect to specific algorithms, the Philips threshold 80, Cole-Kripke, UCSD, and Sadeh all overestimated total sleep time. While Philips threshold 40, Philips threshold 20, and Kripke 2010 underestimate total sleep time.

Rescoring resulted similar results with minor, no improvements, or larger discrepancies in mean difference and distributions. All algorithms demonstrate some mean difference between the algorithm estimates and PSG measures of total sleep time. The Philips Algorithm with a threshold of 80 and Cole-Kripke had the smallest difference. The Bland Altman distribution demonstrated some systematic bias. However, the points were relatively randomly distributed i.e., did not show a specific distribution pattern. There were only a few outliers, and we expect these would not significantly impact results. The estimates do not appear to get worse as total sleep time decreased or increased. With respect to specific algorithms, the Philips threshold 80, Cole-Kripke, UCSD, and Sadeh all overestimated total sleep time. While Philips threshold 40, Philips threshold 20, and Kripke 2010 underestimate total sleep time.

**Figure S21**

Mean difference statistics total sleep time (TST) for CPAP subgroup non-rescored algorithms.^a^


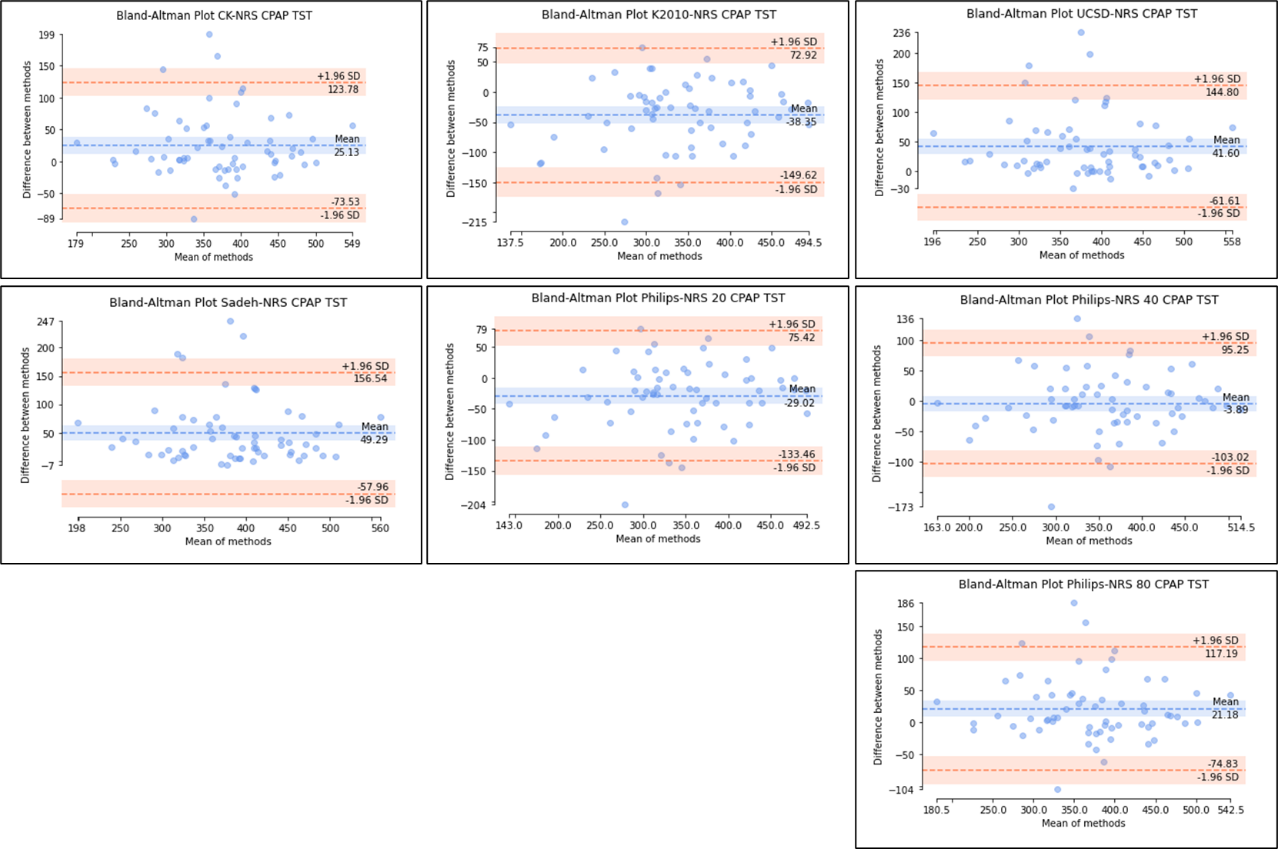


^a. Plots represent the Bland Altman distributions of non-rescored actigraphy algorithms in comparison to polysomnography (PSG; ground truth) for Total Sleep Time (TST) for the CPAP subgroup. The y-axis represents the difference between methods while the x-axis represents the mean of methods. The mean difference, standard deviation of the difference, lower and upper limits of agreement (95%) are listed below each respective graph. Each graph showcases the limits in the orange dashed lights while the blue dashed line represents the mean difference. Abbreviated names for each algorithm are as follows: CK = Cole Kripke, K2010 = Kripke 2010, UCSD, Sadeh, and Philips.^

**Figure S22**

Mean difference statistics total sleep time (TST) for CPAP subgroup rescored algorithms.^a^


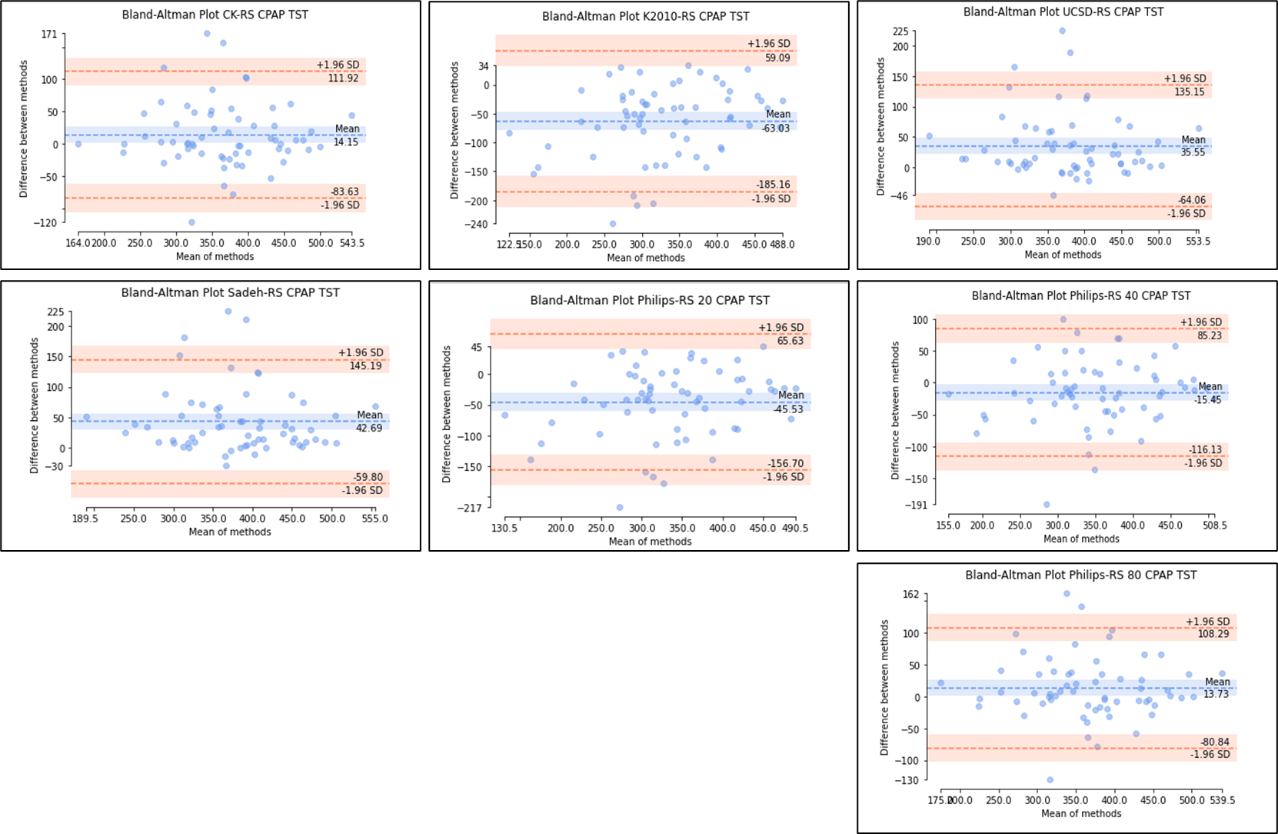


^a. Plots represent the Bland Altman distributions of rescored actigraphy algorithms in comparison to polysomnography (PSG; ground truth) for Total Sleep Time (TST) for the CPAP subgroup. The y-axis represents the difference between methods while the x-axis represents the mean of methods. The mean difference, standard deviation of the difference, lower and upper limits of agreement (95%) are listed below each respective graph. Each graph showcases the limits in the orange dashed lights while the blue dashed line represents the mean difference. Abbreviated names for each algorithm are as follows: CK = Cole Kripke, K2010 = Kripke 2010, UCSD, Sadeh, and Philips.^

**Table S21**

Mean difference statistics total sleep time (TST) for CPAP subgroup non-rescored algorithms.


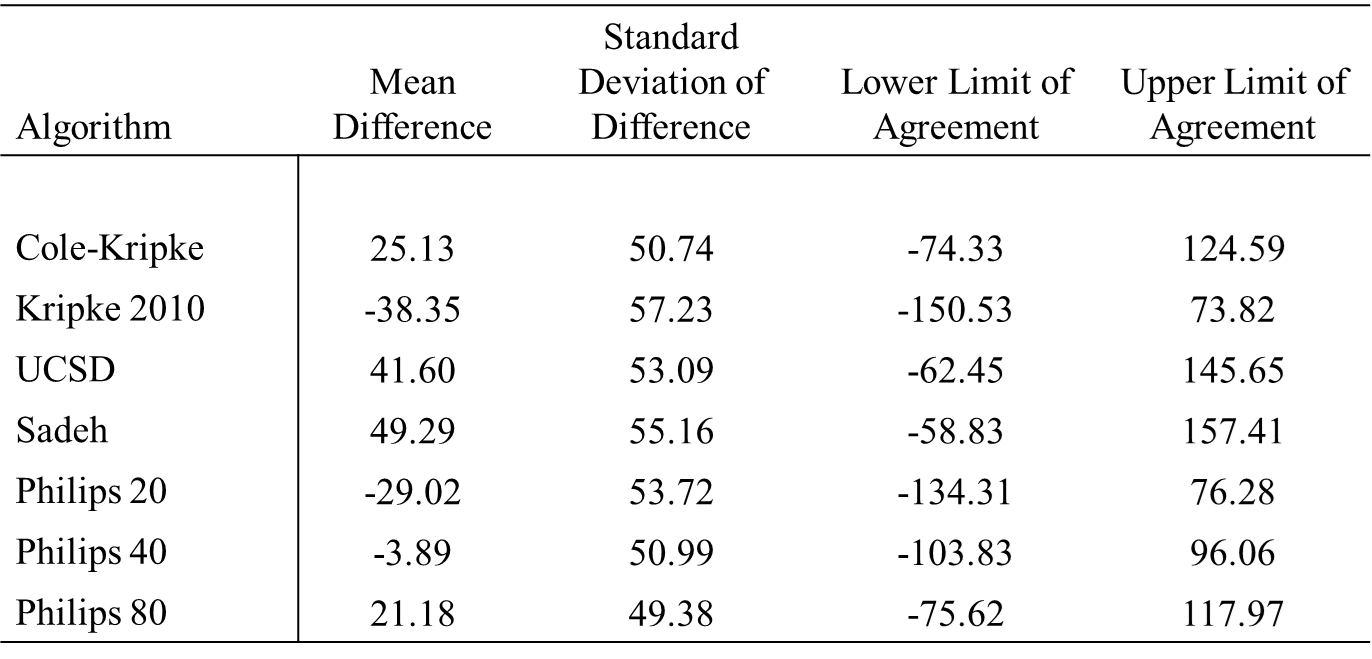


**Table S22**

Mean difference statistics total sleep time (TST) for CPAP subgroup rescored algorithms.


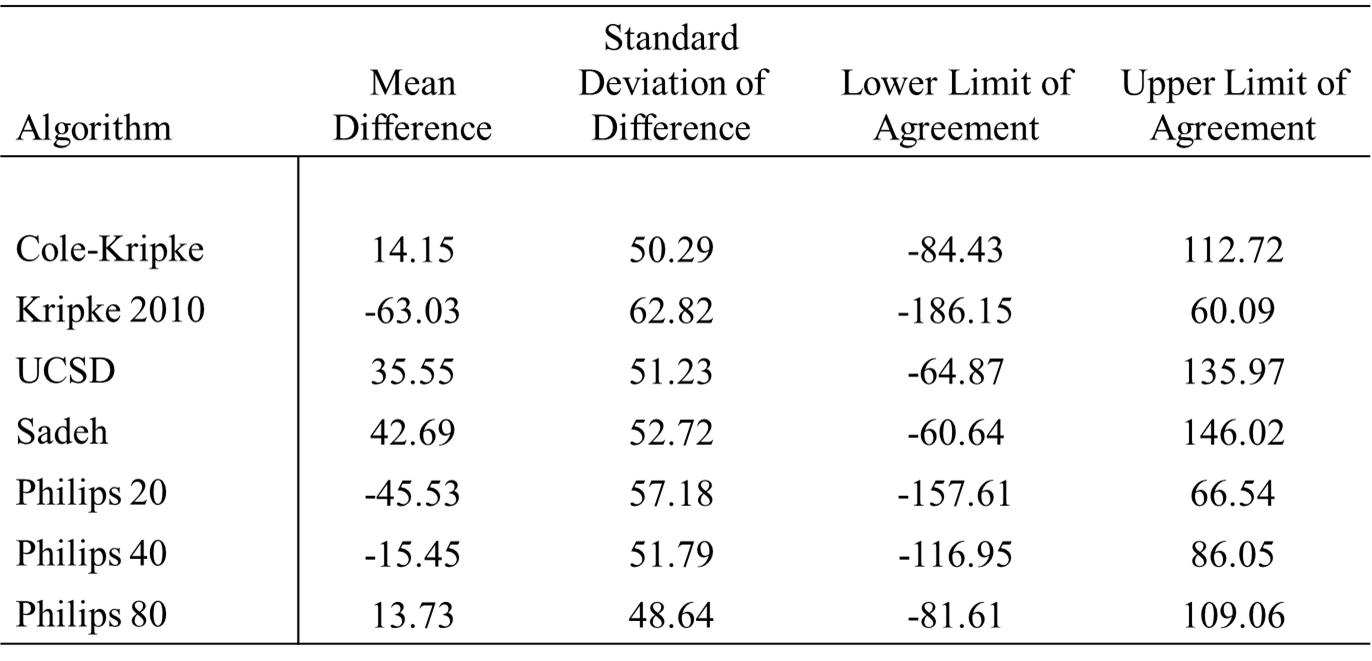


### Bland-Altman Distributions Wake After Sleep Onset

For the RLS subpopulation, all algorithms demonstrate some mean difference between the algorithm estimates and PSG measures of WASO. The Philips Algorithm with a threshold of 20 had minimal difference. The Bland Altman distribution demonstrate clear systematic bias. The Cole-Kripke , UCSD, Sadeh, and Philips Threshold 80 demonstrate clear heteroscedasticity. While the Kripke 2010, Philips threshold 20 and 40 demonstrated some indication of heteroscedasticity as the spread of points increased relative to WASO. That is, WASO estimates were better at lower WASO averages and become considerably worse as WASO increased. There were only a few outliers, and we expect these would not significantly impact results. The Kripke 2010 algorithm overestimated total sleep WASO. While the Philips algorithms, Cole-Kripke, UCSD, and Sadeh all underestimated WASO.

Rescoring resulted similar results with minor, no improvements, or larger discrepancies in mean difference and distributions. All algorithms demonstrate some mean difference between the algorithm estimates and PSG measures of WASO. The Philips Algorithm with a threshold of 40 and 20 had the smallest difference. The Bland Altman distribution demonstrate clear systematic bias. The all algorithms demonstrate clear heteroscedasticity. That is, WASO estimates were better at lower WASO averages and become considerably worse as WASO increased. There were only a few outliers, and we expect these would not significantly impact results. The Philips threshold 40, Philips threshold 80, Cole-Kripke, UCSD, and Sadeh all underestimated WASO. While Philips threshold 20 and Kripke 2010 overestimated total sleep WASO.

**Figure S23**

Mean difference statistics wake after sleep onset (WASO) for CPAP subgroup non-rescored algorithms.^a^


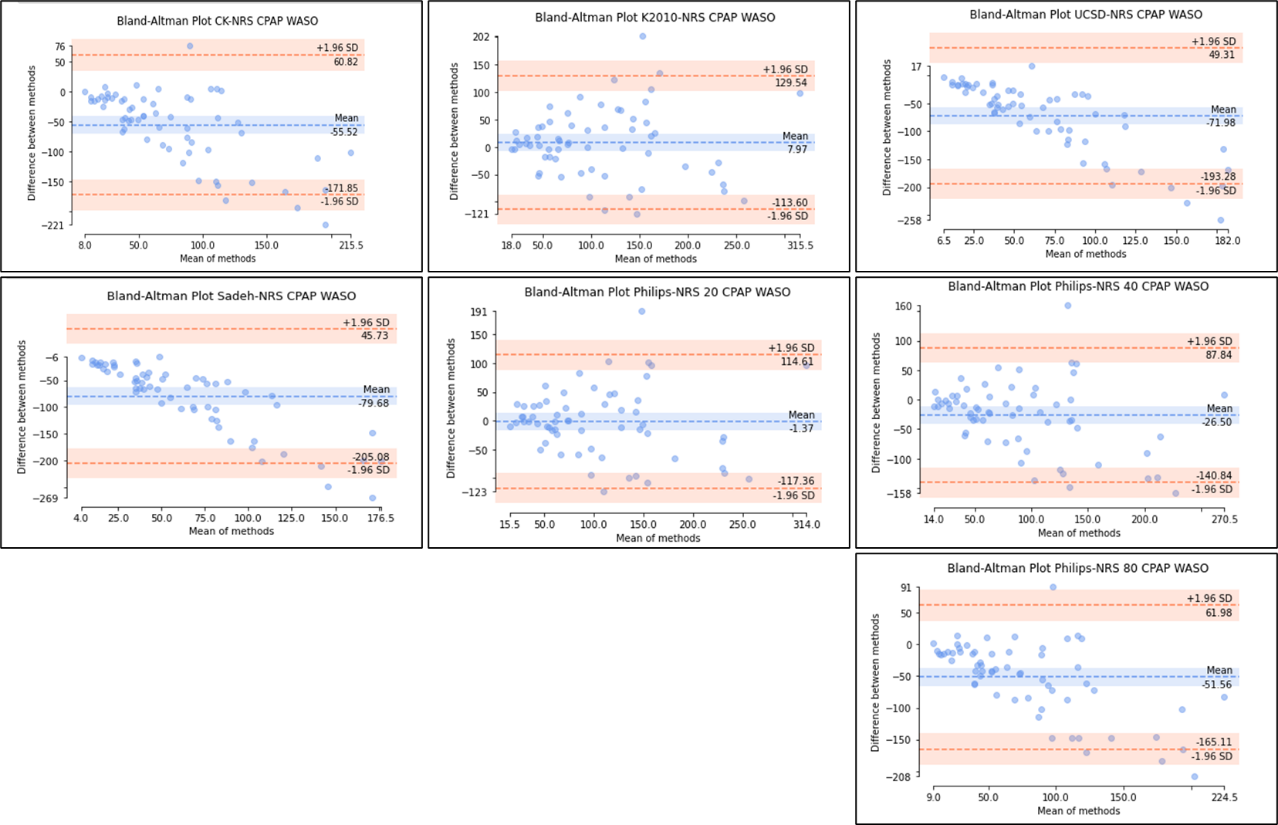


^a. Plots represent the Bland Altman distributions of non-rescored actigraphy algorithms in comparison to polysomnography (PSG; ground truth) for Wake After Sleep Onset (WASO) for the CPAP Subgroup. The y-axis represents the difference between methods while the x-axis represents the mean of methods. The mean difference, standard deviation of the difference, lower and upper limits of agreement (95%) are listed below each respective graph. Each graph showcases the limits in the orange dashed lights while the blue dashed line represents the mean difference. Abbreviated names for each algorithm are as follows: CK = Cole Kripke, K2010 = Kripke 2010, UCSD, Sadeh, and Philips.^

**Figure S24**

Mean difference statistics wake after sleep onset (WASO) for CPAP subgroup rescored algorithms.^a^


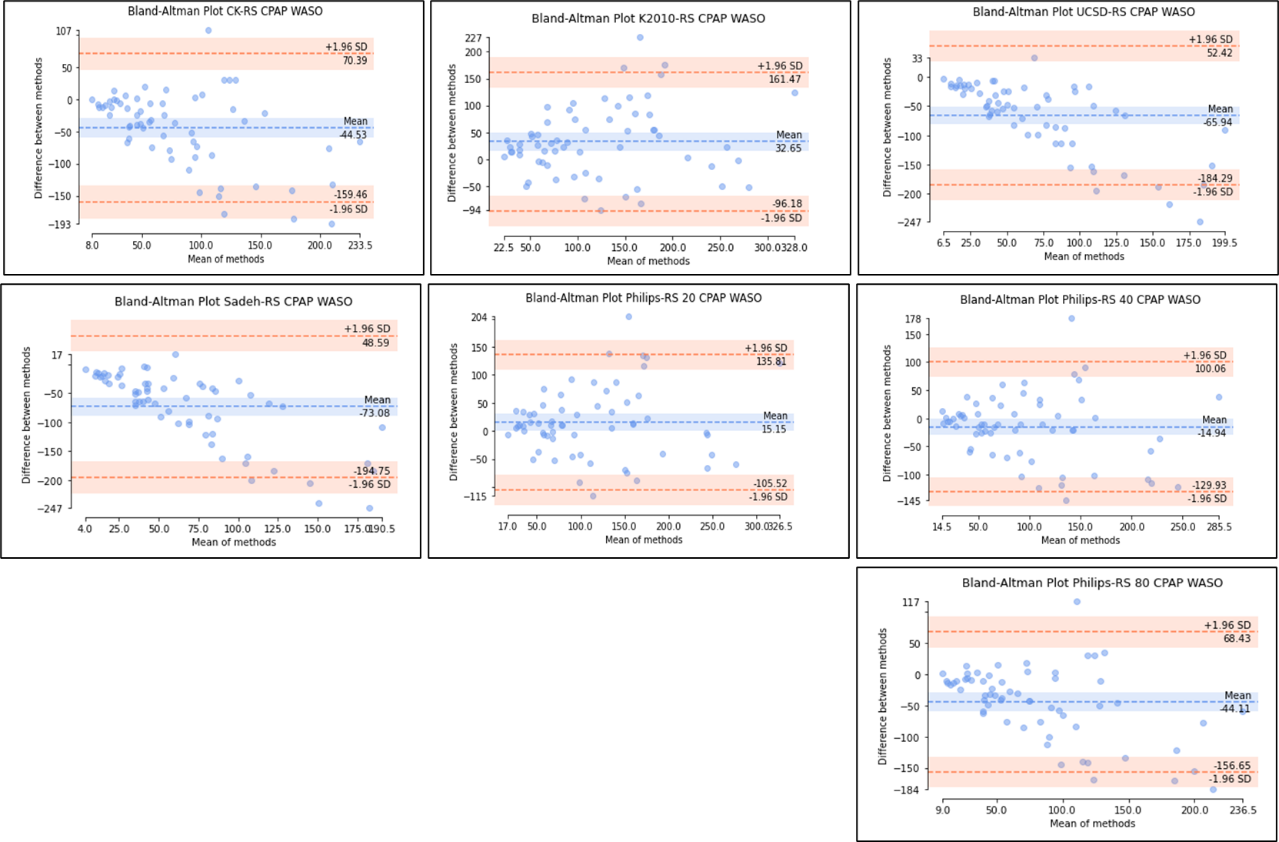


^a. Plots represent the Bland Altman distributions of rescored actigraphy algorithms in comparison to polysomnography (PSG; ground truth) for Wake After Sleep Onset (WASO) for the CPAP Subgroup. The y-axis represents the difference between methods while the x-axis represents the mean of methods. The mean difference, standard deviation of the difference, lower and upper limits of agreement (95%) are listed below each respective graph. Each graph showcases the limits in the orange dashed lights while the blue dashed line represents the mean difference. Abbreviated names for each algorithm are as follows: CK = Cole Kripke, K2010 = Kripke 2010, UCSD, Sadeh, and Philips.^

**Table S23**

Mean difference statistics wake after sleep onset (WASO) for CPAP subgroup non-rescored algorithms.


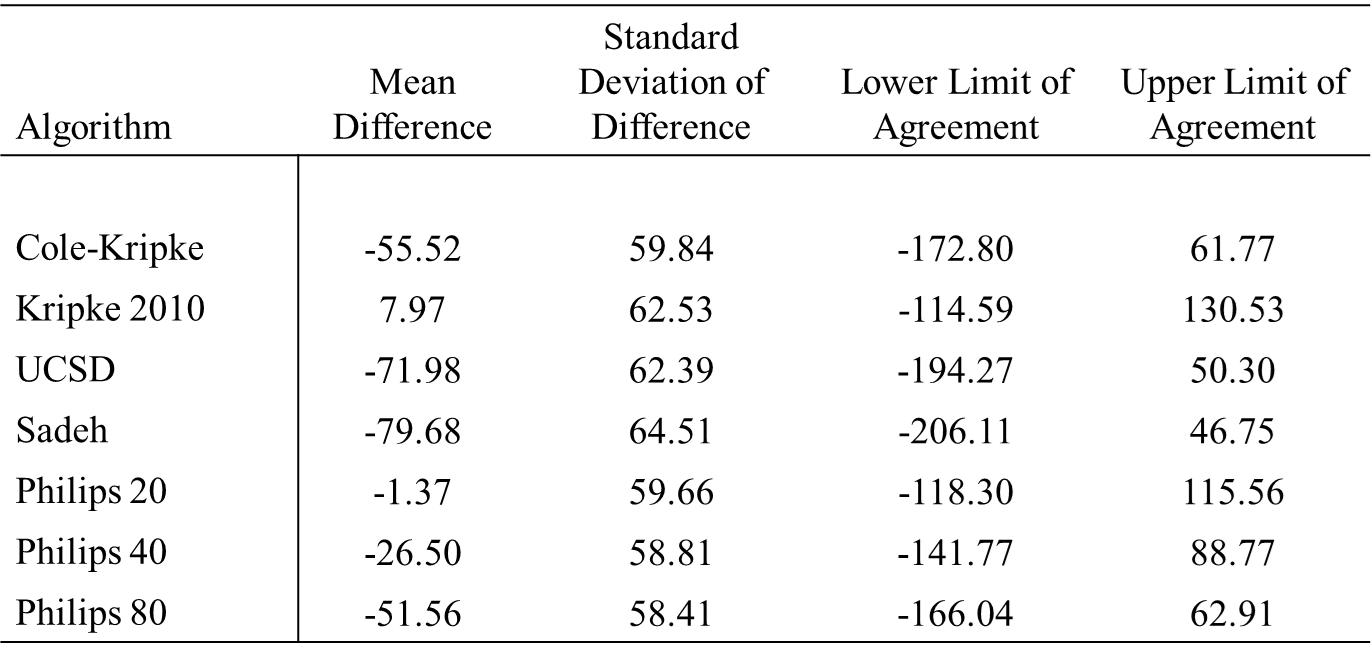


**Figure S24**

Mean difference statistics wake after sleep onset (WASO) for CPAP subgroup rescored algorithms.


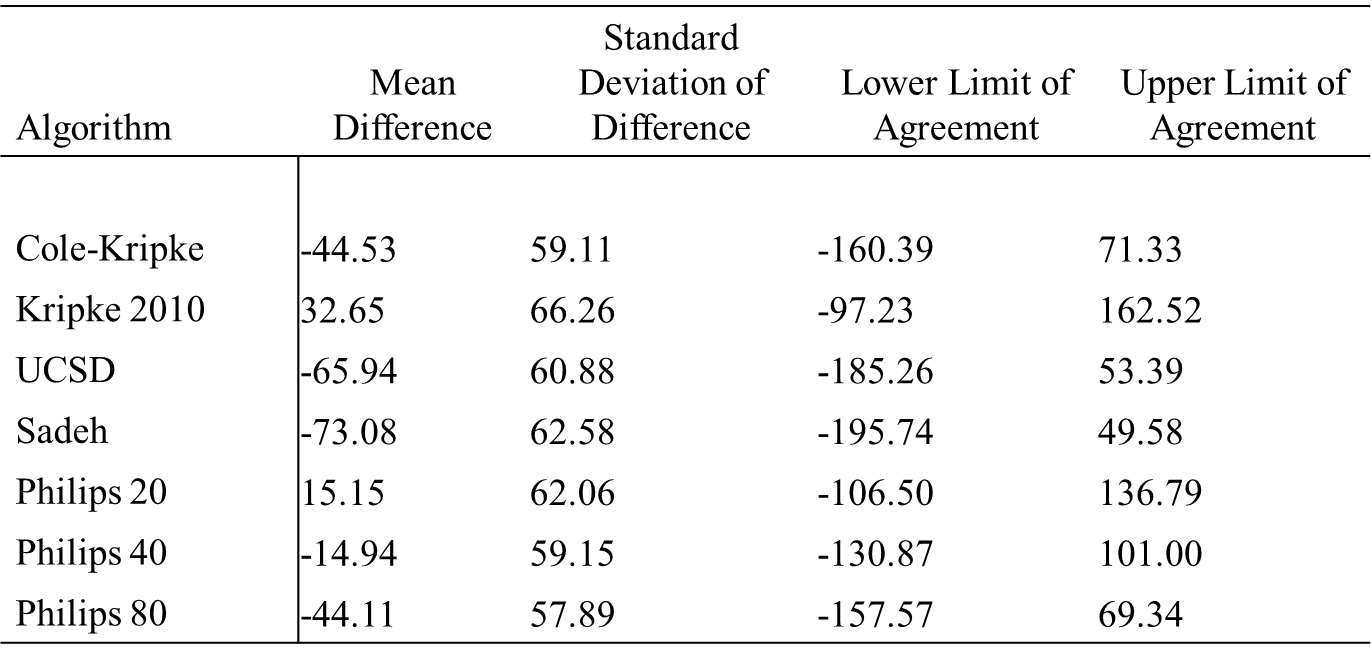

Supplement: Multimedia Appendix 11 [file formative-v9-e70778-s011.docx]
